# Supplementary material for: Engineering Cathepsin S Selective Chemical Probes and Antibody−Drug Conjugates through Substrate Profiling with Unnatural Amino Acids
Source: J Med Chem. 2026 Jul 1;69(14):16550–69. doi: 10.1021/acs.jmedchem.6c00202 (PMC13403234; doi:10.1021/acs.jmedchem.6c00202)
Supplement: Supplementary file 1 [file jm6c00202_si_001.pdf]

Supporting information

Engineering cathepsin S selective chemical probes and antibody-drug conjugates through substrate profiling with unnatural amino acids

Maria Łęcka[1,#,*], Oliwia Gorzeń[1,#], Natalia Ćwilichowska-Puślecka[1], Julia Nguyen[1], Martyna Majchrzak[1], Vanessa Pippa[1], Piotr Jakimowicz[1], Jerzy Wiśniewski[1], Bartosz Dołęga-Kozierowski[2], Piotr Kasprzak[2], Boris Turk[3,4], Marcin Drąg[1,5], Rafał Matkowski[2,6], Marcin Poręba[1,7,*]

[1]Faculty of Chemistry, Wroclaw University of Science and Technology, 50-370 Wroclaw, Poland; [2]Lower Silesian Oncology, Pulmonology and Hematology Center, 53-413 Wroclaw, Poland; [3]Jozef Stefan Institute, Ljubljana, 1000, Slovenia; [4]Faculty of Chemistry and Chemical Engineering, University of Ljubljana, 1000 Ljubljana, Slovenia; [5]Centre for Chemical Biology, Institute of Physical Chemistry, Polish Academy of Sciences, 01-224 Warsaw, Poland; [6]Wrocław Medical University, Faculty of Medicine, Department of Oncology, 50-367, [7]Lead contact; [#]equal contribution, *correspondence: maria.lecka@pwr.edu.pl, marcin.poreba@pwr.edu.pl

Supporting information

Figure S1. Substrate specificity of cysteine cathepsins at the P1 position……………………….3

Figure S2. Substrate specificity of cathepsin S at the P4-P2 positions (P1-Arg HyCoSuL)…….4

Figure S3. Substrate specificity of cathepsin S at the P4-P2 positions (P1-Gln HyCoSuL)…….5

Table S1. Chemical structures of first-generation substrates for cathepsin S…………………..6

Table S2. Chemical structures of second-generation substrates for cathepsin S………………8

Table S3. Chemical structures of third-generation substrates for cathepsin S…………………9

Table S4. Kinetic parameters (k_{cat}/K_M) of third-generation cathepsin S substrates……………11

Table S5. Michaelis-Menten constant (K_M) for third-generation cathepsin S substrates………11

Table S6. k_{cat} values for third-generation cathepsin S substrates………………………………12

Table S7. Structures of cathepsin S-selective inhibitors…………………………………………12

Table S8. Second-order rate constants of cathepsin S inhibitors (k_{obs}/[I])……………………..13

Table S9. Structures of cathepsin S-selective activity-based probes (ABPs)…………………14

Table S10. Chemical structures of cathepsin S-selective peptide prodrugs…………………..15

Figure S4 Cathepsin S-cleavable antibody-drug conjugates ……………………..…………..…16

Figure S5 ADCs stability in serum ……………………………………………………………...16

Table S11. Metal-conjugated antibody panel for mass cytometry profiling of breast tumor architecture ..………………………………………………………………………………………17

Table S12. Metal-conjugated antibody panel for detection of tumor-associated biomarkers and cathepsin S by mass cytometry. ………………………………………………………………17

Figure S6. Single-cell mass cytometry profiling of the breast tumor microenvironment …………………………………..…………………………………………………...……18

Table S13. The LC-MS analysis of substrates, inhibitors, activtiy-based probes and prodrugs for cathepsin S…………………………………………………………………………………..19

Table S14. The LC-MS spectra of substrates, inhibitors, activtiy-based probes and prodrugs for cathepsin S ……………………………………………………………………………………22

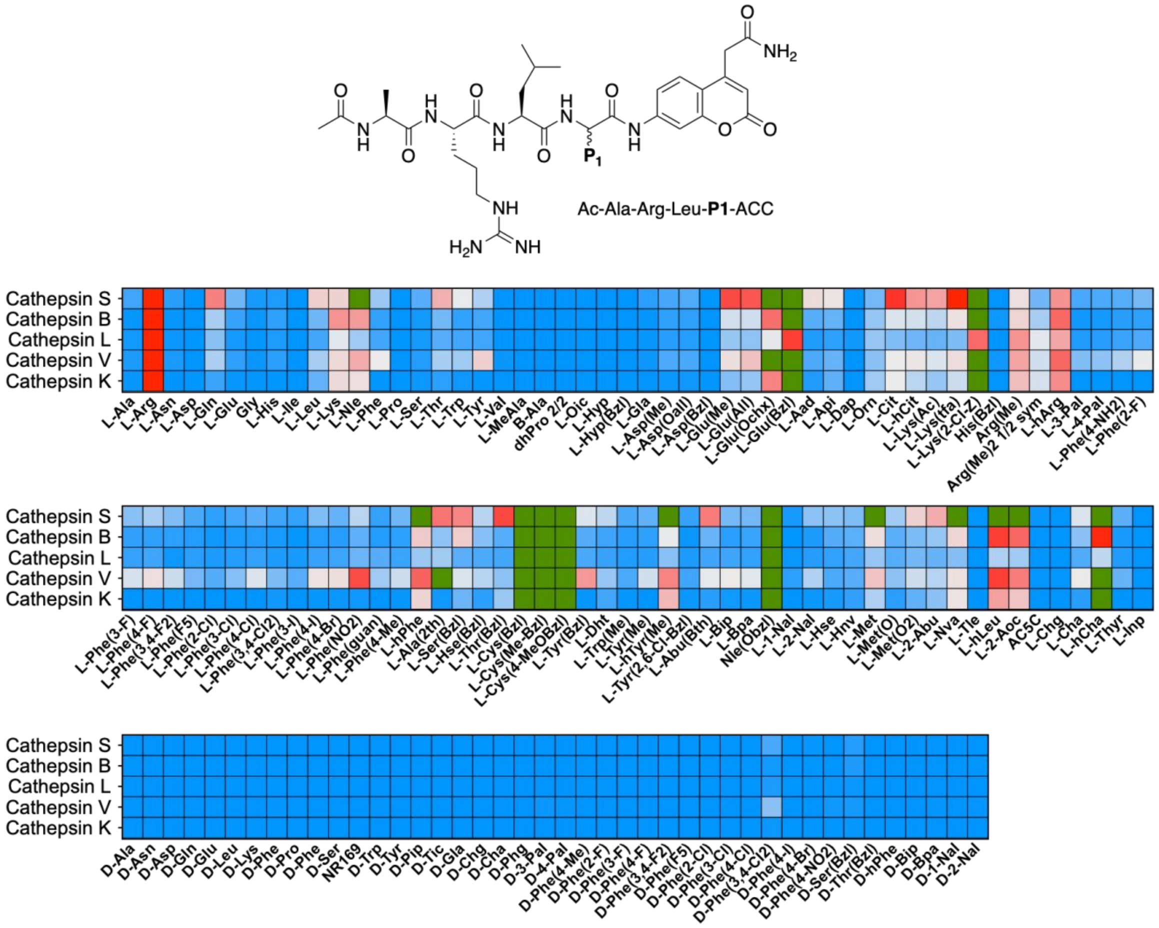

Figure S1. Substrate specificity of cysteine cathepsins at the P1 position. The P1 substrate preferences of human cysteine cathepsins were determined using a fluorogenic substrate library based on the Ac-Ala-Arg-Leu-P1-ACC scaffold, comprising 19 natural and over 100 unnatural amino acids. Specificity profiling was performed in triplicate. Substrate hydrolysis rates (RFU/s) are presented as average values, with standard deviations (SD) below 10% for each substrate. To facilitate comparison, data were normalized to the activity against arginine (set to 100%), and a red-to-blue heatmap was used to visualize relative preferences. Unnatural amino acids that were better recognized than arginine (>100%) are highlighted in green. P1 specificity data for cathepsins B, L, V, and K were taken from our previously published studies.

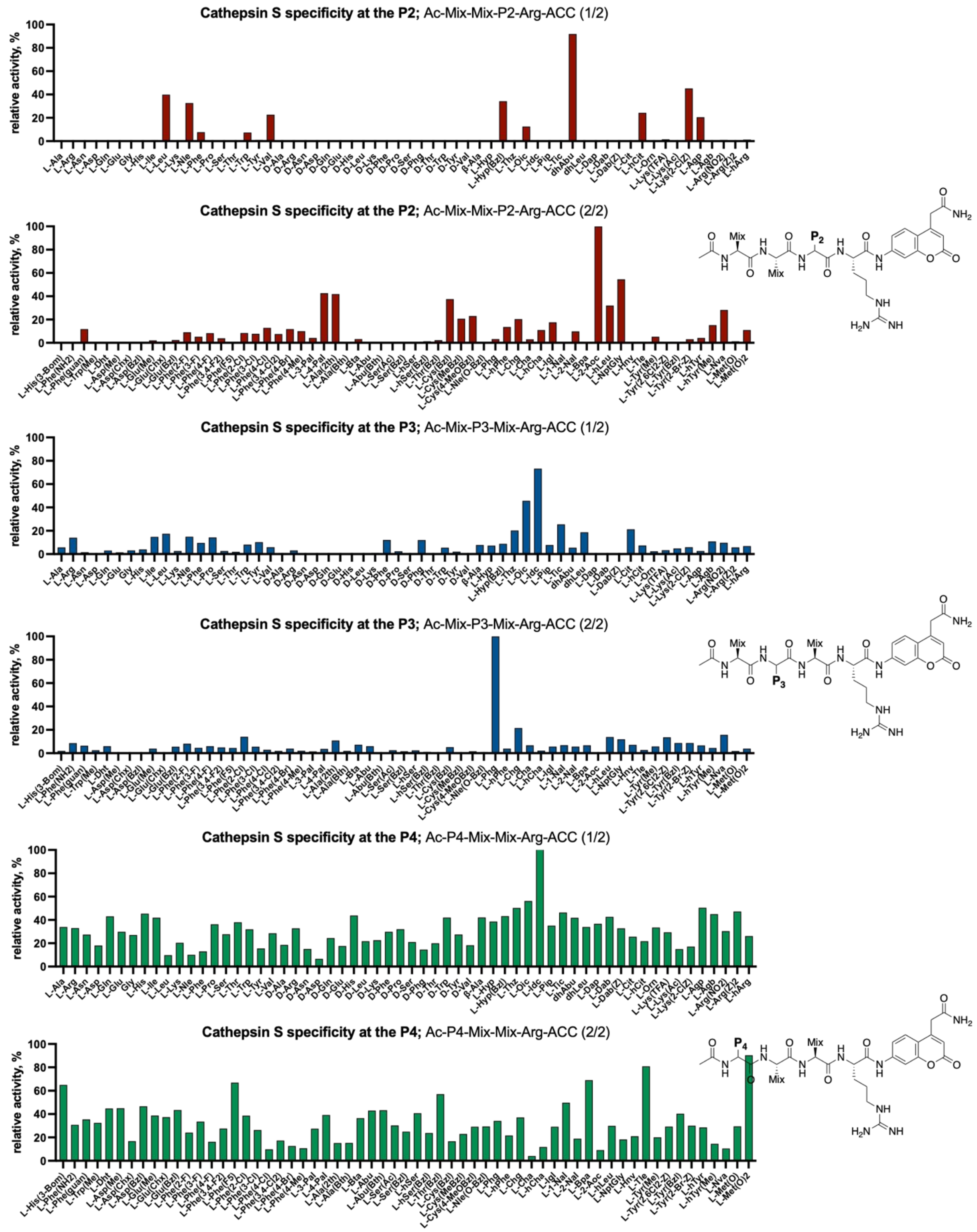

Figure S2. Substrate specificity of cathepsin S at the P4-P2 positions (P1-Arg HyCoSuL). The x-axis shows the abbreviated names of natural and unnatural amino acids, while the y-axis represents the relative enzymatic activity of cathepsin S for each substrate. The most efficiently cleaved amino acid at each position was set to 100%, and the activities of other amino acids were normalized accordingly. The structures of the P4, P3, and P2 sublibraries are presented on the right side.

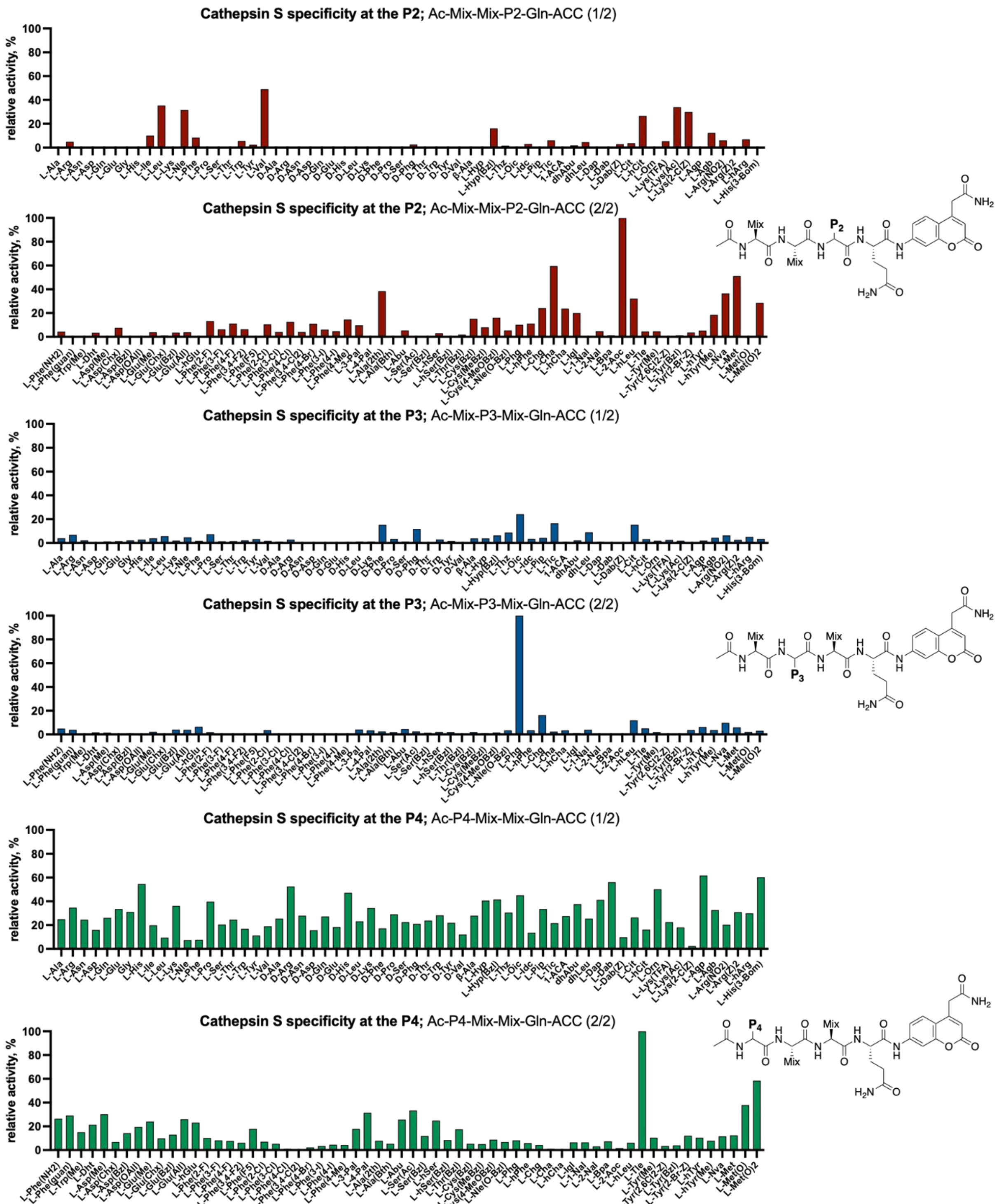

Figure S3. **Substrate specificity of cathepsin S at the P4-P2 positions (P1-Gln HyCoSuL).** The x-axis shows the abbreviated names of natural and unnatural amino acids, while the y-axis represents the relative enzymatic activity of cathepsin S for each substrate. The most efficiently cleaved amino acid at each position was set to 100%, and the activities of other amino acids were normalized accordingly. The structures of the P4, P3, and P2 sublibraries are presented on the right side.

Table S1. **Chemical structures of first-generation substrates for cathepsin S.** All substrates follow the general structure Ac-P4-P3-P2-Arg-ACC. The table lists the sequences and corresponding chemical structures designed to explore substrate preferences at the P4-P2 positions, with a fixed Arg residue at P1.

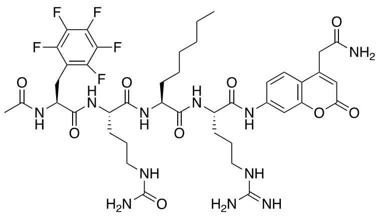<br>**OG-64**<br>Ac-Phe(F$_5$)-Cit-2Aoc-Arg-ACC	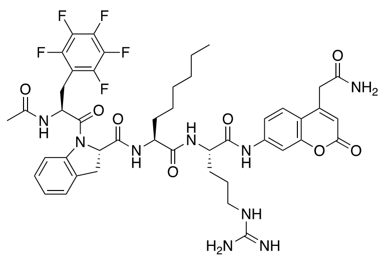<br>**OG-65**<br>Ac-Phe(F$_5$)-Idc-2Aoc-Arg-ACC	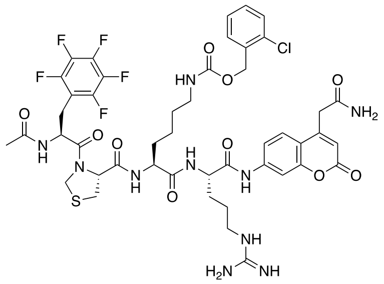<br>**OG-66**<br>Ac-Phe(F$_5$)-Thz-Lys(2ClZ)-Arg-ACC
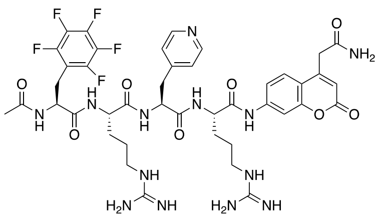<br>**OG-67**<br>Ac-Phe(F$_5$)-Cit-3Pal-Arg-ACC	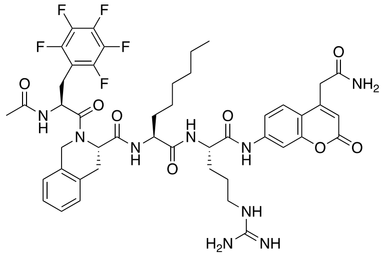<br>**OG-68**<br>Ac-Phe(F$_5$)-Tic-2Aoc-Arg-ACC	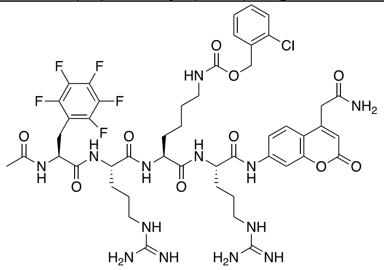<br>**OG-69**<br>Ac-Phe(F$_5$)-Arg-Lys(2ClZ)-Arg-ACC
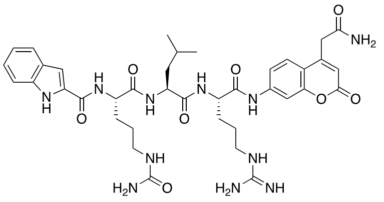<br>**OG-70**<br>HN-Idc-Cit-Leu-Arg-ACC	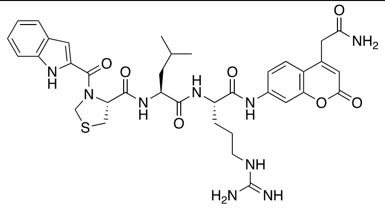<br>**OG-71**<br>HN-Idc-Thz-Leu-Arg-ACC	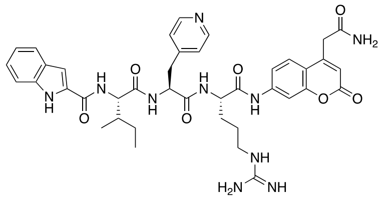<br>**OG-72**<br>HN-Idc-Ile-3Pal-Arg-ACC
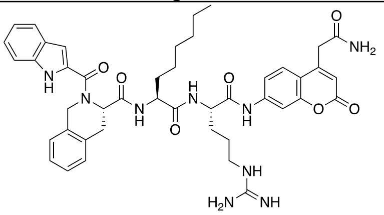<br>**OG-73**<br>HN-Idc-Tic-2Aoc-Arg-ACC	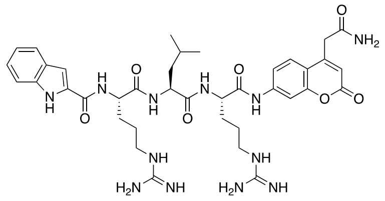<br>**OG-74**<br>HN-Idc-Arg-Leu-Arg-ACC	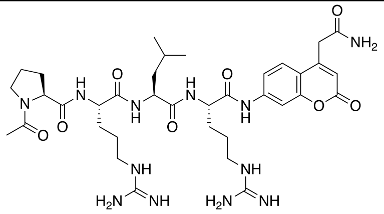<br>**OG-75**<br>Ac-Pro-Arg-Leu-Arg-ACC
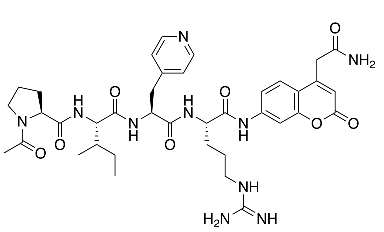<br>**OG-76**<br>Ac-Pro-Ile-3Pal-Arg-ACC	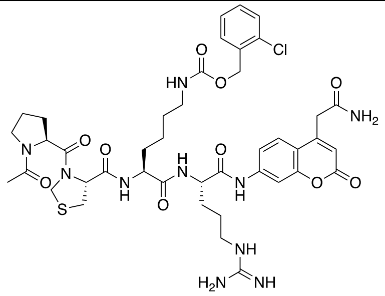<br>**OG-77**<br>Ac-Pro-Thz-Lys(2ClZ)-Arg-ACC	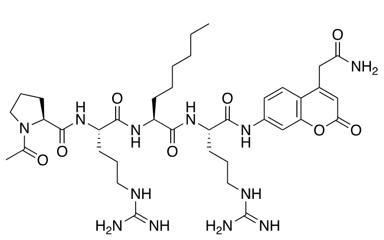<br>**OG-79**<br>Ac-Pro-Arg-2Aoc-Arg-ACC

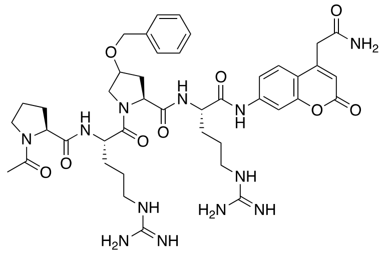

OG-80
Ac-Pro-Arg-Hyp(Bzl)-Arg-ACC

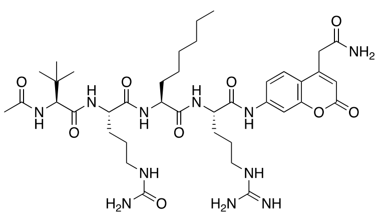

OG-81
Ac-Tle-Cit-2Aoc-Arg-ACC

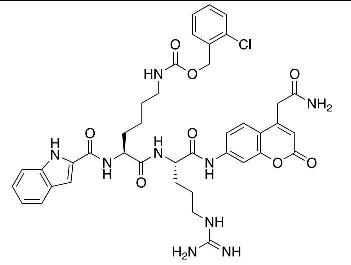

OG-82
Ac-Tle-Idc-Lys(2ClZ)-Arg-ACC

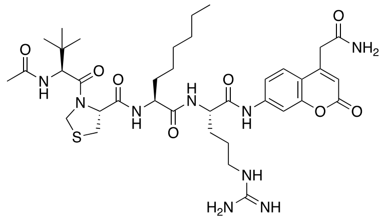

OG-83
Ac-Tle-Thz-2Aoc-Arg-ACC

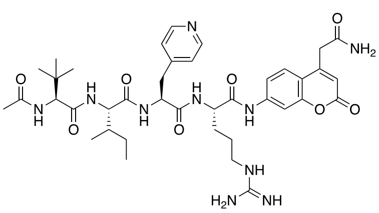

OG-84
Ac-Tle-Ile-3Pal-Arg-ACC

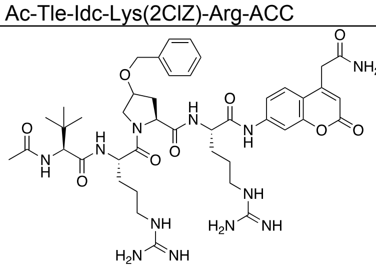

OG-85
Ac-Tle-Arg-Hyp(Bzl)-Arg-ACC

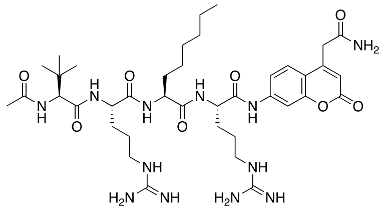

OG-86
Ac-Tle-Arg-2Aoc-Arg-ACC

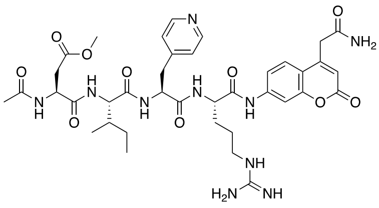

OG-87
Ac-Asp(Me)-Ile-3Pal-Arg-ACC

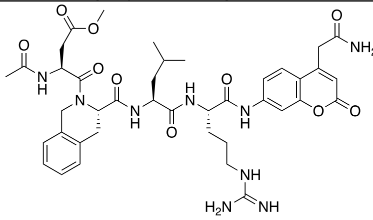

OG-88
Ac-Asp(Me)-Tic-Leu-Arg-ACC

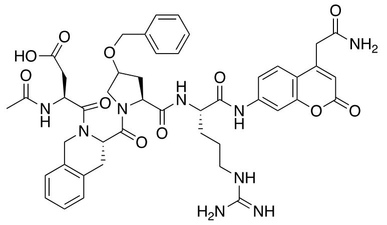

OG-89
Ac-Asp(Bzl)-Tic-Hyp(Bzl)-Arg-ACC

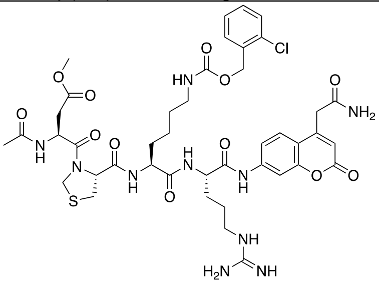

OG-90
Ac-Asp(Bzl)-Thz-Lys(2ClZ)-Arg-ACC

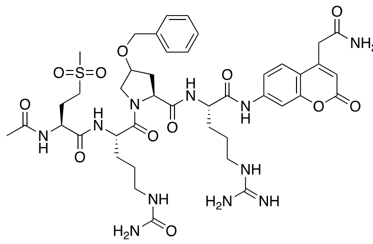

OG-91
Ac-Met(O_2)-Cit-Hyp(Bzl)-Arg-ACC

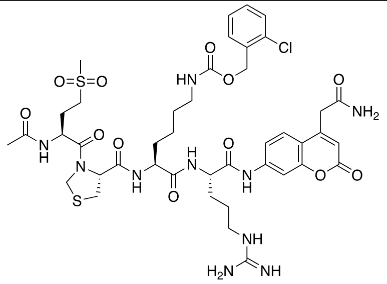

OG-93
Ac-Met(O_2)-Thz-Lys(2ClZ)-Arg-ACC

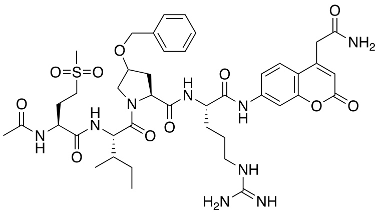

OG-94
Ac-Met(O_2)-Ile-Hyp(Bzl)-Arg-ACC

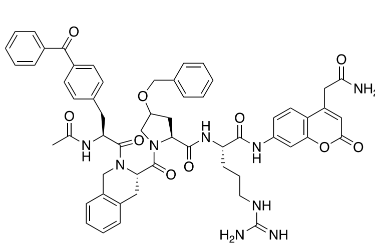

OG-95
Ac-Bpa-Tic-Hyp(Bzl)-Arg-ACC

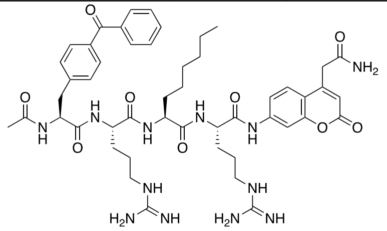

OG-96
Ac-Bpa-Arg-2Aoc-Arg-ACC

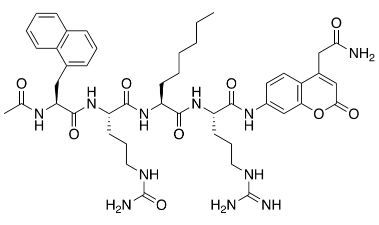

OG-115
Ac-1Nal-Cit-2Aoc-Arg-ACC

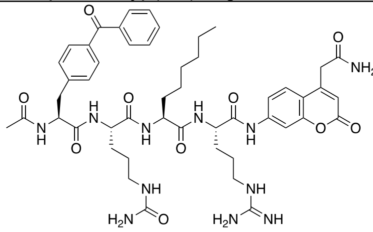

OG-116
Ac-Bpa-Cit-2Aoc-Arg-ACC

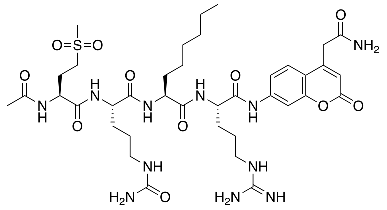

OG-117
Ac-Met(O$_2$)-Cit-2Aoc-Arg-ACC

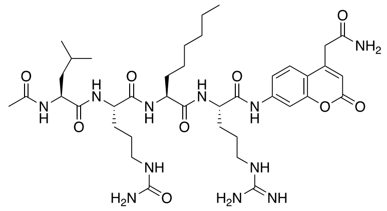

OG-118
Ac-Leu-Cit-2Aoc-Arg-ACC

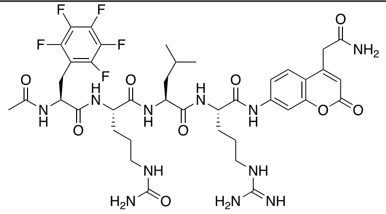

OG-119
Ac-Phe(F$_5$)-Cit-Leu-Arg-ACC

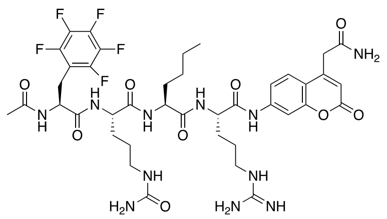

OG-120
Ac-Phe(F$_5$)-Cit-Nle-Arg-ACC

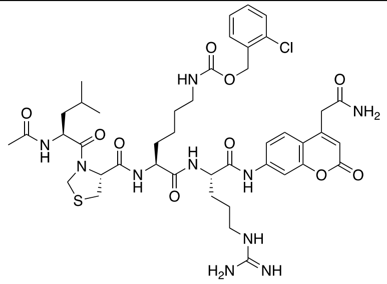

OG-121
Ac-Leu-Thz-Lys(2ClZ)-Arg-ACC

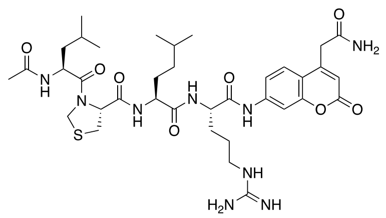

OG-122
Ac-Leu-Thz-hLeu-Arg-ACC

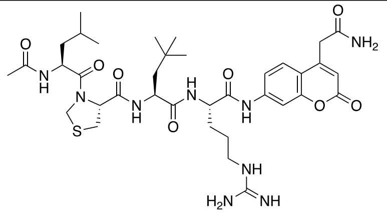

OG-123
Ac-Leu-Thz-NptGly-Arg-ACC

Table S2. Chemical structures of second-generation substrates for cathepsin S. All substrates follow the general structure Ac-Phe(F$_5$)-Cit-2Aoc-P1-ACC. This library was designed to optimize the P1 position using the fixed P4-P2 motif derived from the most selective first-generation substrate.

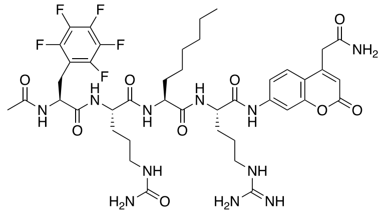

OG-64
Ac-Phe(F$_5$)-Cit-2Aoc-Arg-ACC

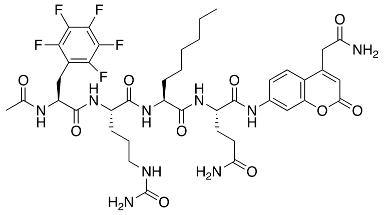

OG-133
Ac-Phe(F$_5$)-Cit-2Aoc-Gln-ACC

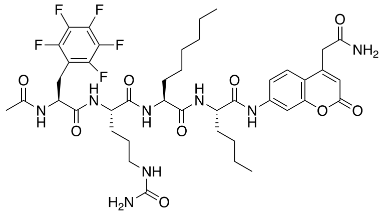

OG-134
Ac-Phe(F$_5$)-Cit-2Aoc-Nle-ACC

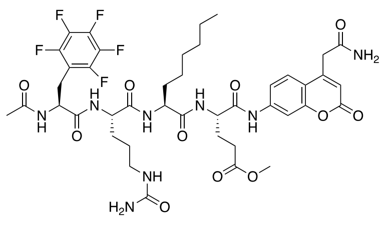

OG-135
Ac-Phe(F$_5$)-Cit-2Aoc-Glu(Me)-ACC

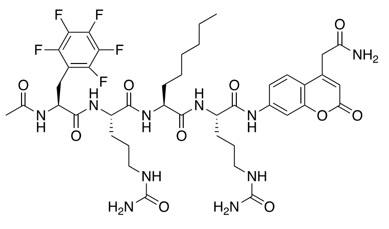

OG-136
Ac-Phe(F$_5$)-Cit-2Aoc-Cit-ACC

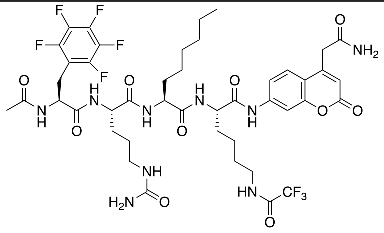

OG-137
Ac-Phe(F$_5$)-Cit-2Aoc-Lys(TFA)-ACC

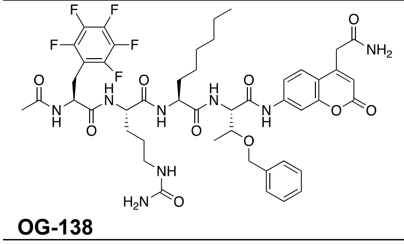

OG-138

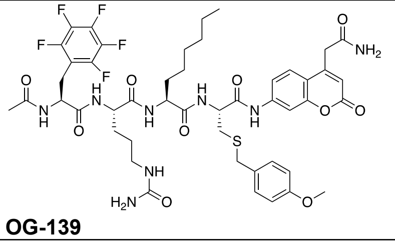

OG-139

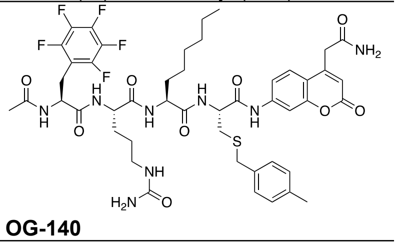

OG-140

Ac-Phe(F5)-Cit-2Aoc-Thr(Bzl)-ACC	Ac-Phe(F5)-Cit-2Aoc-Cys(MeOBzl)-ACC	Ac-Phe(F5)-Cit-2Aoc-Cys(MeBzl)-ACC
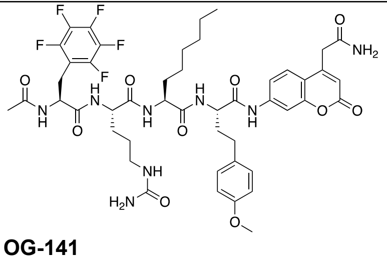 **OG-141** Ac-Phe(F5)-Cit-2Aoc-hTyr(Me)-ACC	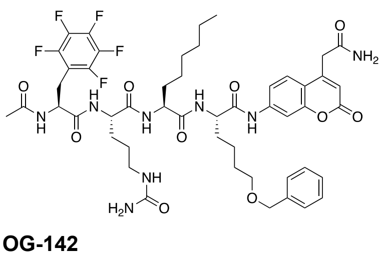 **OG-142** Ac-Phe(F5)-Cit-2Aoc-Nle(OBzl)-ACC	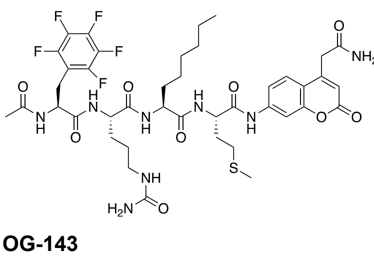 **OG-143** Ac-Phe(F5)-Cit-2Aoc-Met-ACC
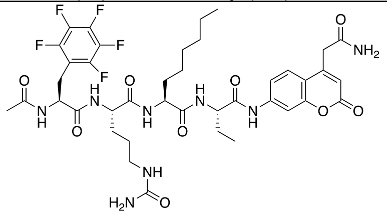 **OG-144** Ac-Phe(F5)-Cit-2Aoc-Abu-ACC	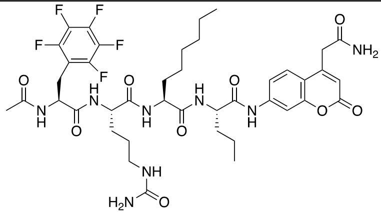 **OG-145** Ac-Phe(F5)-Cit-2Aoc-Nva-ACC	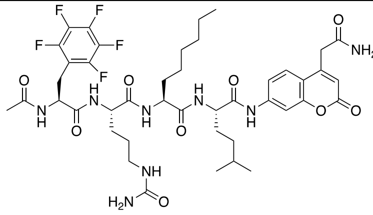 **OG-146** Ac-Phe(F5)-Cit-2Aoc-hLeu-ACC
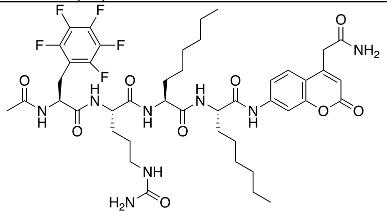 **OG-147** Ac-Phe(F5)-Cit-2Aoc-2Aoc-ACC		

Table S3. Chemical structures of third-generation substrates for cathepsin S. These substrates were developed by further refining the peptide sequence to enhance cathepsin S selectivity and activity. Variations in the P4-P1 positions were introduced based on previous substrate screening results, aiming to improve enzymatic cleavage efficiency and specificity.

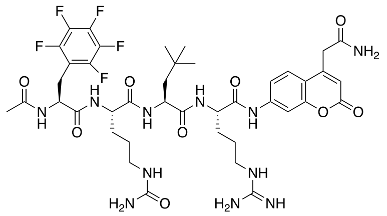 **OG-191** Ac-Phe(F5)-Cit-NptGly-Arg-ACC	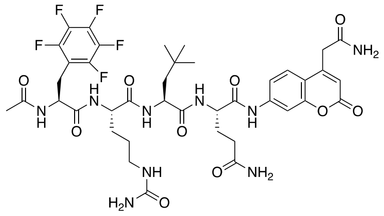 **OG-192** Ac-Phe(F5)-Cit-NptGly-Gln-ACC	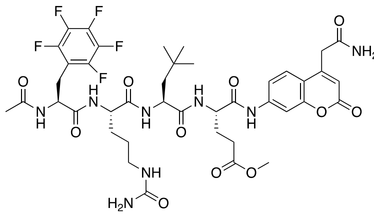 **OG-193** Ac-Phe(F5)-Cit-NptGly-Glu(Me)-ACC
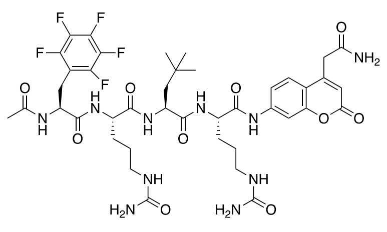 **OG-194** Ac-Phe(F5)-Cit-NptGly-Cit-ACC	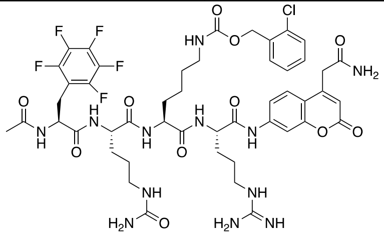 **OG-195** Ac-Phe(F5)-Cit-Lys(2ClZ)-Arg-ACC	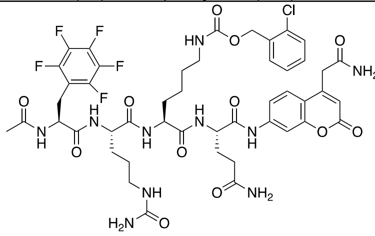 **OG-196** Ac-Phe(F5)-Cit-Lys(2ClZ)-Gln-ACC

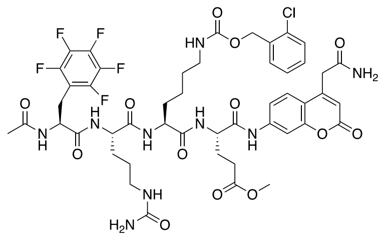

OG-197
Ac-Phe(F_5)-Cit-Lys(2ClZ)-Glu(Me)-ACC

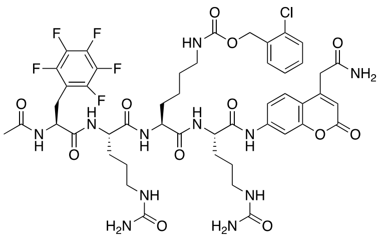

OG-198
Ac-Phe(F_5)-Cit-Lys(2ClZ)-Cit-ACC

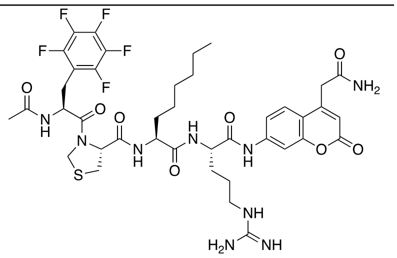

OG-199
Ac-Phe(F_5)-Thz-2Aoc-Arg-ACC

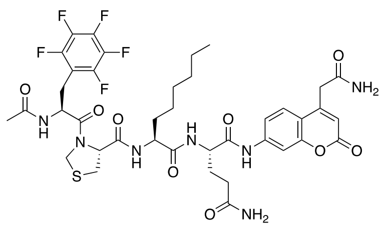

OG-200
Ac-Phe(F_5)-Thz-2Aoc-Gln-ACC

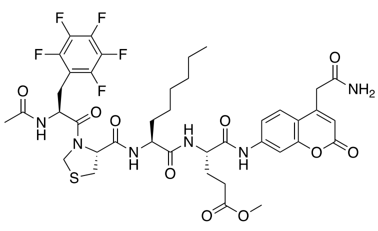

OG-201
Ac-Phe(F_5)-Thz-2Aoc-Glu(Me)-ACC

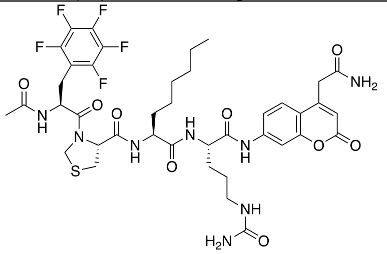

OG-202
Ac-Phe(F_5)-Thz-2Aoc-Cit-ACC

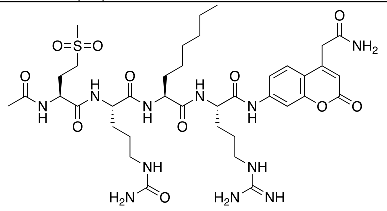

OG-203
Ac-Met(O_2)-Cit-2Aoc-Arg-ACC

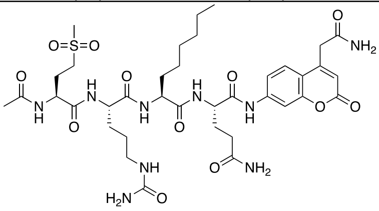

OG-204
Ac-Met(O_2)-Cit-2Aoc-Gln-ACC

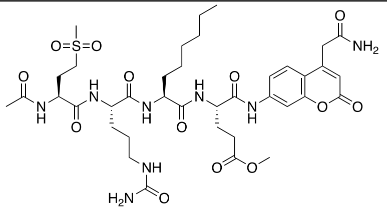

OG-205
Ac-Met(O_2)-Cit-2Aoc-Glu(Me)-ACC

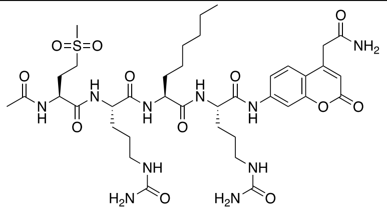

OG-206
Ac-Met(O_2)-Cit-2Aoc-Cit-ACC

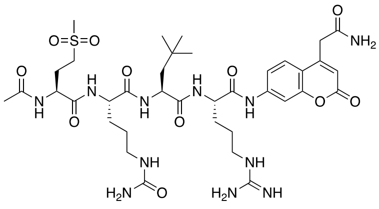

OG-207
Ac-Met(O_2)-Cit-NptGly-Arg-ACC

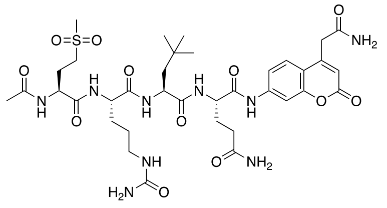

OG-208
Ac-Met(O_2)-Cit-NptGly-Gln-ACC

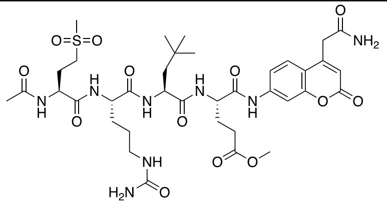

OG-209
Ac-Met(O_2)-Cit-NptGly-Glu(Me)-ACC

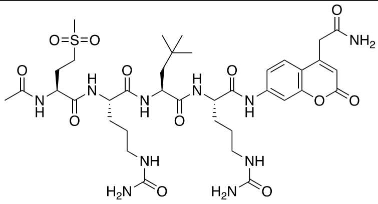

OG-210
Ac-Met(O_2)-Cit-NptGly-Cit-ACC

Table S4. Kinetic parameters (k_{cat}/K_M) of third-generation cathepsin S substrates. Catalytic efficiency (k_{cat}/K_M) values were determined for each substrate to evaluate the impact of sequence modifications on cathepsin S cleavage. Measurements were performed under steady-state conditions, and the data reflect the enzyme's substrate preference and turnover efficiency.

P4-P2	P1	Code	k_{cat}/K_M, $M^{-1}s^{-1}$ Cathepsin S	Cathepsin B	Cathepsin L	Cathepsin V
Phe(F_5)-Cit-2Aoc	Arg	**OG-64**	574,000	144,000	13,800	25,400
Phe(F_5)-Cit-NptGly	Arg	**OG-191**	382,000	32,200	5,000	92,800
	Gln	**OG-192**	119,000	6,400	6,100	28,100
	Glu(Me)	**OG-193**	133,000	7,900	7,800	22,700
	Cit	**OG-194**	115,000	11,200	6,700	38,600
Phe(F_5)-Cit-Lys(2ClZ)	Arg	**OG-195**	364,000	73,200	10,200	14,200
	Gln	**OG-196**	688,000	45,400	5,200	10,400
	Glu(Me)	**OG-197**	356,000	5,500	2,100	7,700
	Cit	**OG-198**	438,000	18,700	4,400	17,900
Phe(F_5)-Thz-2Aoc	Arg	**OG-199**	498,000	119,000	12,400	38,500
	Gln	**OG-200**	93,600	14,800	1,200	46,400
	Glu(Me)	**OG-201**	214,000	29,200	2,500	25,900
	Cit	**OG-202**	247,000	54,500	2,900	56,700
Met(O_2)-Cit-2Aoc	Arg	**OG-203**	1,097,000	232,000	102,000	93,900
	Gln	**OG-204**	268,000	186,000	24,700	32,300
	Glu(Me)	**OG-205**	867,000	127,000	9,600	22,100
	Cit	**OG-206**	166,000	96,500	12,000	17,000
Met(O_2)-Cit-NptGly	Arg	**OG-207**	529,000	21,900	50,700	83,800
	Gln	**OG-208**	91,400	3,700	8,900	36,800
	Glu(Me)	**OG-209**	564,000	5,100	8,300	33,100
	Cit	**OG-210**	219,000	7,500	9,800	37,200

Table S5. **Michaelis-Menten constant (K_M) values for third-generation cathepsin S substrates.** K_M values were determined to assess the substrate binding affinity of cathepsin S for each third-generation peptide sequence. Measurements were carried out under steady-state conditions, and the data represent the average of at least two independent experiments.

P4-P2	P1	Code	K_M, μM Cathepsin S	Cathepsin B	Cathepsin L	Cathepsin V
Phe(F_5)-Cit-2Aoc	Arg	**OG-64**	8,53	8,52	10,9	16,5
Phe(F_5)-Cit-NptGly	Arg	**OG-191**	62,2	62,2	169	59,9
	Gln	**OG-192**	21,2	20,4	63,7	27,1
	Glu(Me)	**OG-193**	15,2	12,6	41,0	22,5
	Cit	**OG-194**	9,43	24,9	72,9	22,1
Phe(F_5)-Cit-Lys(2ClZ)	Arg	**OG-195**	4,39	5,33	19,5	18,3
	Gln	**OG-196**	0,27	0,88	23,0	13,4
	Glu(Me)	**OG-197**	0,74	3,63	42,8	19,4
	Cit	**OG-198**	0,62	2,67	36,5	12,8
Phe(F_5)-Thz-2Aoc	Arg	**OG-199**	8,70	74,7	19,3	11,4
	Gln	**OG-200**	1,63	20,3	15,9	4,31
	Glu(Me)	**OG-201**	3,04	15,8	12,1	11,2
	Cit	**OG-202**	8,37	21,3	24,4	4,94
Met(O_2)-Cit-2Aoc	Arg	**OG-203**	11,3	10,1	15,8	12,9
	Gln	**OG-204**	7,24	10,0	10,1	8,35
	Glu(Me)	**OG-205**	6,54	16,9	58,6	25,8
	Cit	**OG-206**	9,52	10,9	11,7	7,65
Met(O_2)-Cit-NptGly	Arg	**OG-207**	42,5	201	133	118
	Gln	**OG-208**	48,1	294	105	102
	Glu(Me)	**OG-209**	39,2	369	236	176
	Cit	**OG-210**	32,9	322	242	54,4

Table S6. k_{cat} values for third-generation cathepsin S substrates. k_{cat} values were determined to assess the cathepsin S turnover toward 3rd generation fluorescent substrates. Measurements were carried out under steady-state conditions, and the data represent the average of at least two independent experiments.

P4-P2	P1	Code	k_{cat}, M^{-1}			
			Cathepsin S	Cathepsin B	Cathepsin L	Cathepsin V
Phe(F_5)-Cit-2Aoc	Arg	**OG-64**	4,90	1,23	0,15	0,42
Phe(F_5)-Cit-NptGly	Arg	**OG-191**	23,76	2,00	0,85	5,56
	Gln	**OG-192**	2,52	0,13	0,39	0,76
	Glu(Me)	**OG-193**	2,02	0,10	0,32	0,51
	Cit	**OG-194**	1,08	0,28	0,49	0,85
Phe(F_5)-Cit-Lys(2ClZ)	Arg	**OG-195**	1,60	0,39	0,20	0,26
	Gln	**OG-196**	0,19	0,04	0,12	0,14
	Glu(Me)	**OG-197**	0,26	0,02	0,09	0,15
	Cit	**OG-198**	0,27	0,05	0,16	0,23
Phe(F_5)-Thz-2Aoc	Arg	**OG-199**	4,33	8,90	0,24	0,44
	Gln	**OG-200**	0,15	0,30	0,02	0,20
	Glu(Me)	**OG-201**	0,65	0,46	0,03	0,29
	Cit	**OG-202**	2,07	1,16	0,07	0,28
Met(O_2)-Cit-2Aoc	Arg	**OG-203**	12,40	2,34	1,61	0,18
	Gln	**OG-204**	1,94	1,86	0,25	0,27
	Glu(Me)	**OG-205**	5,67	2,15	0,56	0,57
	Cit	**OG-206**	1,58	1,05	0,14	0,13
Met(O_2)-Cit-NptGly	Arg	**OG-207**	22,48	4,40	6,74	9,89
	Gln	**OG-208**	4,40	1,09	0,94	3,74
	Glu(Me)	**OG-209**	22,11	1,88	1,96	5,83
	Cit	**OG-210**	7,21	2,41	2,37	2,02

Table S7. Structures of cathepsin S-selective inhibitors with the general formula Ac-Met(O_2)-Cit-NptGly-P1-AOMK (JN1-JN6) or Ac-Phe(F_5)-Cit-Lys(2ClZ)-P1-AOMK (JN7-JN12). Each inhibitor is based on a selective tetrapeptide scaffold and contains a reactive AOMK warhead at the C-terminus. Variations at the P1 position were introduced to fine-tune potency and selectivity toward cathepsin S.

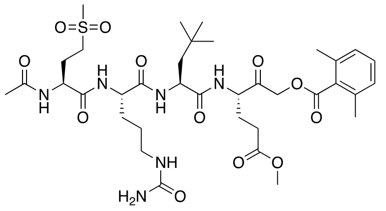

JN-1
Ac-Met(O_2)-Cit-NptGly-Glu(Me)-AOMK

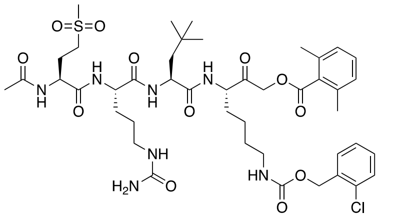

JN-2
Ac-Met(O_2)-Cit-NptGly-Lys(2ClZ)-AOMK

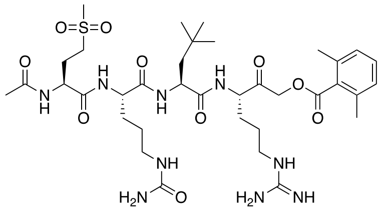

JN-3
Ac-Met(O_2)-Cit-NptGly-Arg-AOMK

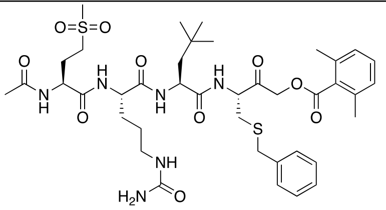

JN-4
Ac-Met(O_2)-Cit-NptGly-Cys(Bzl)-AOMK

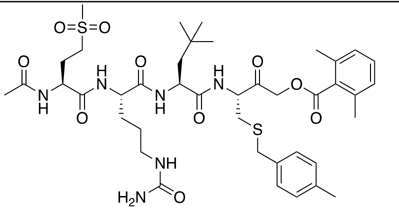

JN-5
Ac-Met(O_2)-Cit-NptGly-Cys(MeBzl)-AOMK

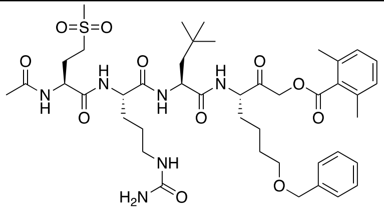

JN-6
Ac-Met(O_2)-Cit-NptGly-Nle(OBzl)-AOMK

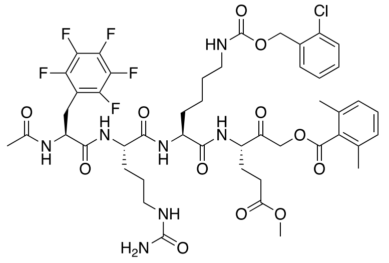

JN-7
Ac-Phe(F5)-Cit-Lys(2ClZ)-Glu(Me)-AOMK

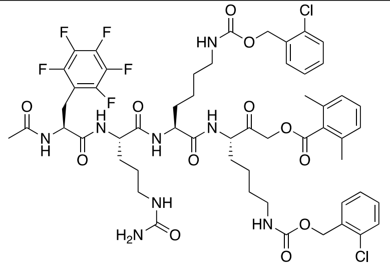

JN-8
Ac-Phe(F5)-Cit-Lys(2ClZ)-Lys(2ClZ)-AOMK

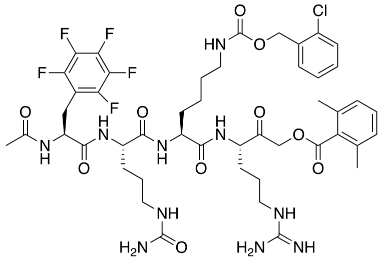

JN-9
Ac-Phe(F5)-Cit-Lys(2ClZ)-Arg-AOMK

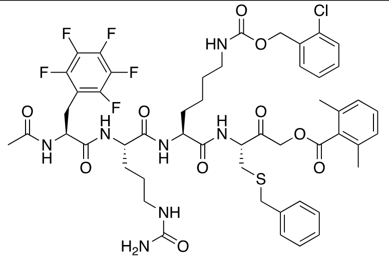

JN-10
Ac-Phe(F5)-Cit-Lys(2ClZ)-Cys(Bzl)-AOMK

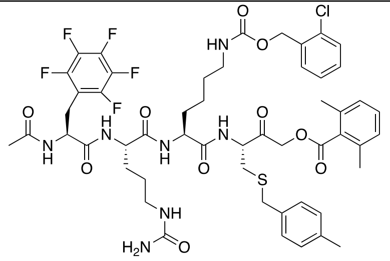

JN-11
Ac-Phe(F5)-Cit-Lys(2ClZ)-Cys(MeBzl)-AOMK

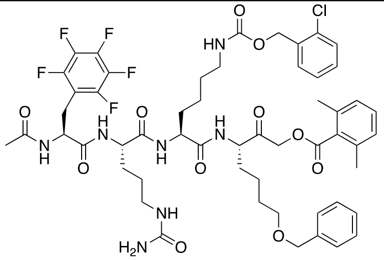

JN-12
Ac-Phe(F5)-Cit-Lys(2ClZ)-Nle(OBzl)-AOMK

Table S8. Second-order rate constants of cathepsin S inhibitors (k_{obs}/[I]). Kinetic analysis of cathepsin S-selective inhibitors was performed to determine their second-order rate constants of inhibition (k_{obs}/[I]). Values were obtained under pseudo-first-order conditions by monitoring the time-dependent decrease in enzyme activity. Higher kobs/[I] values indicate more efficient inhibition.

P4-P2	P1	Code	**k_{obs}/[I], $M^{-1}s^{-1}$**		
			Cathepsin S	Cathepsin L	Cathepsin B
Met(O2)-Cit-NptGly	Glu(Me)	**JN-1**	392,000	560	7,440
	Lys(2ClZ)	**JN-2**	269,000	220	5,100
	Arg	**JN-3**	236,000	4,840	41,500
	Cys(Bzl)	**JN-4**	443,000	450	22,400
	Cys(MeBzl)	**JN-5**	344,000	610	10,500
	Nle(OBzl)	**JN-6**	246,000	650	16,900
Phe(F5)-Cit-Lys(2ClZ)	Glu(Me)	**JN-7**	609,999	870	17,000
	Lys(2ClZ)	**JN-8**	225,000	350	1,600
	Arg	**JN-9**	371,000	4,270	148,000
	Cys(Bzl)	**JN-10**	545,000	620	16,100
	Cys(MeBzl)	**JN-11**	261,000	320	1,900
	Nle(OBzl)	**JN-12**	392,000	490	5,160
		**E-64**	132,000	65,100	81,200

Table S9. Structures of cathepsin S-selective activity-based probes (ABPs) based on optimized substrate sequences. Table presents the chemical structures of fluorescent ABPs designed using the most selective substrate motifs for cathepsin S. Each probe consists of a peptide recognition sequence, an irreversible AOMK warhead, and a fluorescent dye (Cy5 or BODIPY) conjugated to the N-terminus. The design preserves enzymatic selectivity while enabling probe visualization in biochemical and cellular assays.

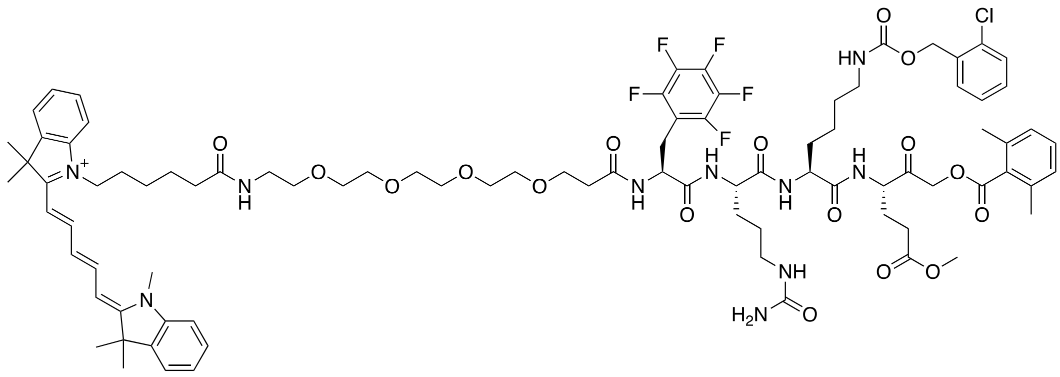

OG-233
Cy5-PEG(4)-Phe(F_5)-Cit-Lys(2ClZ)-Glu(Me)-AOMK

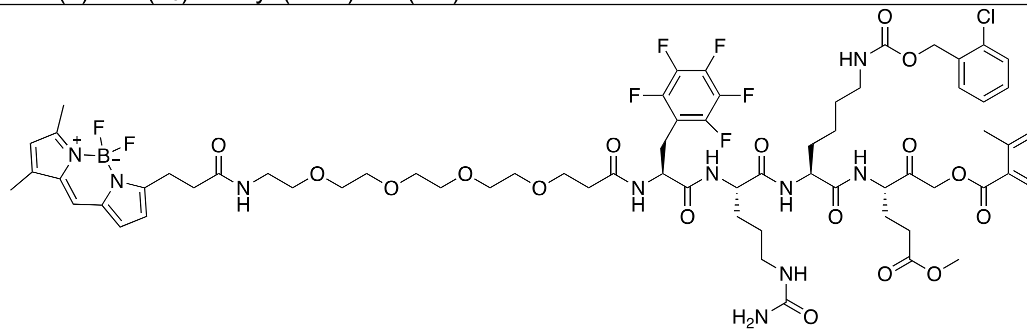

OG-235
BODIPY-PEG(4)-Phe(F_5)-Cit-Lys(2ClZ)-Glu(Me)-AOMK

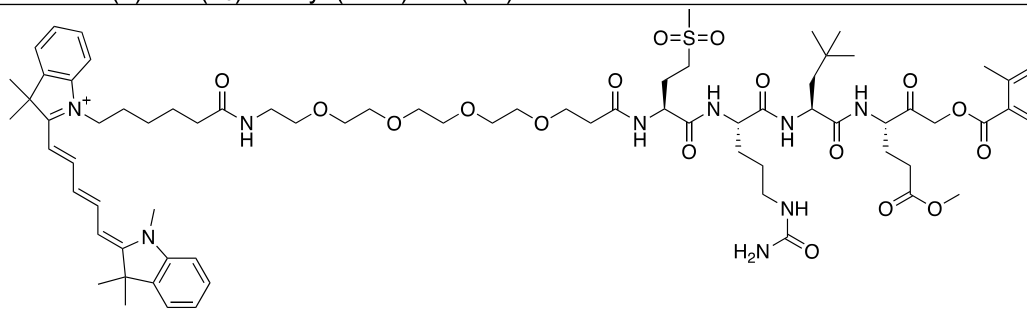

OG-234
Cy5-PEG(4)-Met(O_2)-Cit-NptGly-Glu(Me)-AOMK

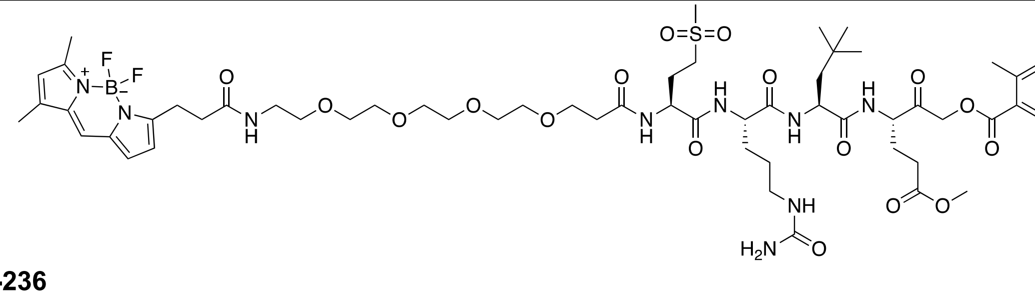

OG-236
BODIPY-PEG(4)-Met(O_2)-Cit-NptGly-Glu(Me)-AOMK

Table S10. Chemical structures of cathepsin S-selective peptide prodrugs (OG-313, OG-314), non-cleavable control (OG-352), and pan-cathepsin prodrug (Z-Val-Cit-PABC-MMAE). This table displays the structures of MMAE-based prodrugs incorporating protease-cleavable peptide linkers. OG-313 and OG-314 contain cathepsin S-selective sequences identified through substrate optimization. OG-352 includes a non-cleavable D-amino acid at the P1 position, serving as a negative control. The widely used Z-Val-Cit-PABC-MMAE is included as a pan-cathepsin-cleavable reference.

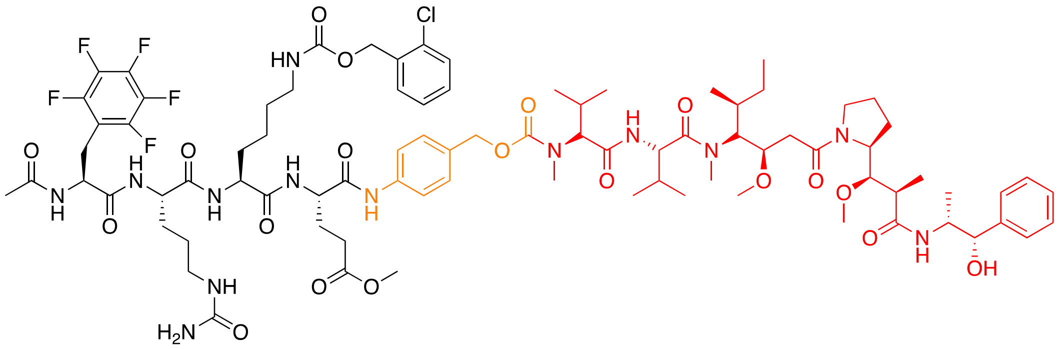

OG-313
Ac-Phe(F_5)-Cit-Lys(2ClZ)-Glu(Me)-PABC-MMAE

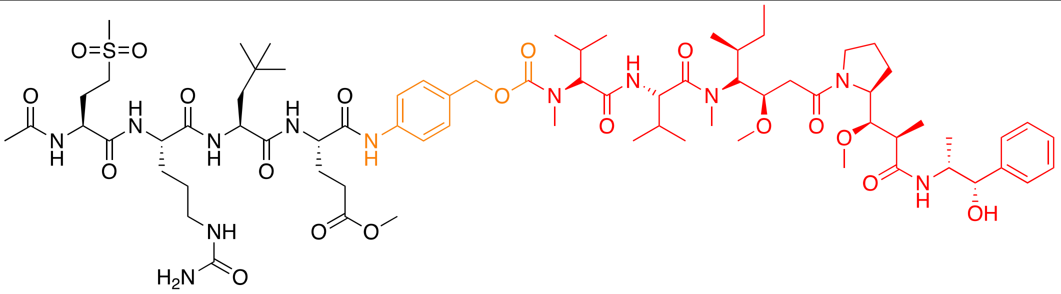

OG-314
Ac-Met(O_2)-Cit-NptGly-Glu(Me)-PABC-MMAE

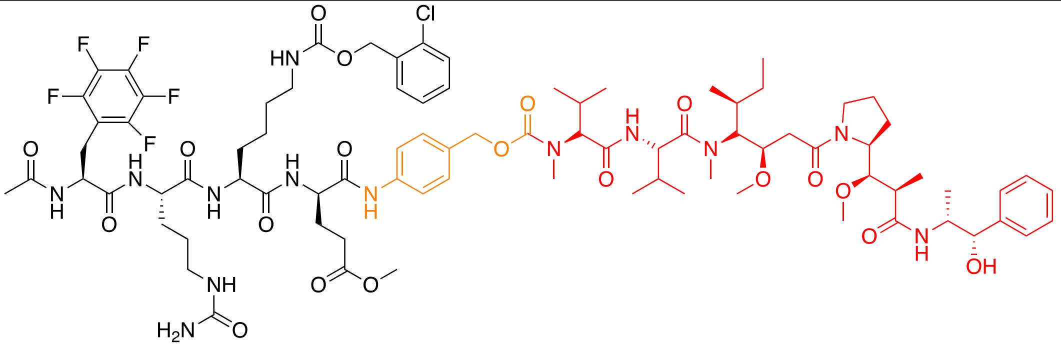

OG-352
Ac-Phe(F_5)-Cit-Lys(2ClZ)-DGlu(Me)-PABC-MMAE

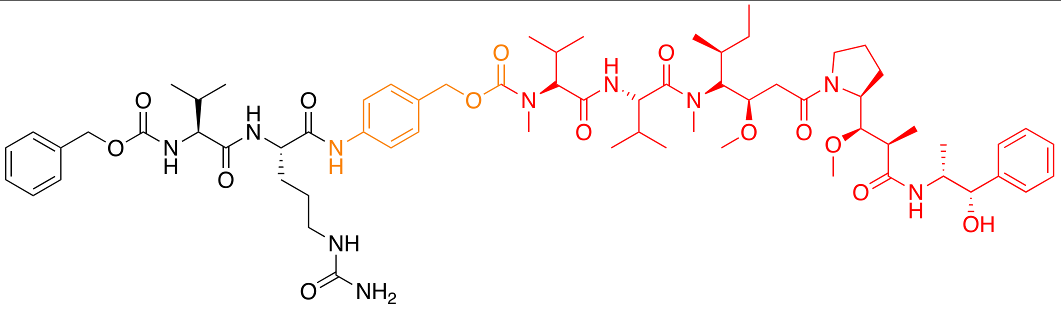

Z-Val-Cit
Cbz-Val-Cit-PABC-MMAE

Trastuzumab-PEG(4)-peptide-PABC-MMAE

ADC	peptide	DAR
**300**	Val-Cit	7.6 *
**300D**	Val-**D**Cit	6.0 *
**313**	Phe(F$_5$)-Cit-Lys(2ClZ)-Glu(Me)	4.6
**313D**	Phe(F$_5$)-Cit-Lys(2ClZ)-**D**Glu(Me)	5,2

Sacituzumab-PEG(4)-peptide-PABC-MMAE

ADC	peptide	DAR
**300**	Val-Cit	2.2
**300D**	Val-**D**Cit	3.0
**313**	Phe(F$_5$)-Cit-Lys(2ClZ)-Glu(Me)	0.9
**313D**	Phe(F$_5$)-Cit-Lys(2ClZ)-**D**Glu(Me)	2.8
**314**	Met(O$_2$)-Cit-NptGly-Glu(Me)	3.1
**314D**	Met(O$_2$)-Cit-NptGly-**D**Glu(Me)	2.7

Figure S4. Cathepsin S-cleavable antibody-drug conjugates. Drug-to-antibody ratio for four anti-HER-2 ADCs and six anti-TROP-2 ADCs, examined by LC-MS/MS. * ADCs are from Gorzeń et al. Journal od Controlled Release, 2025, doi: 10.1016/j.jconrel.2025.114269.

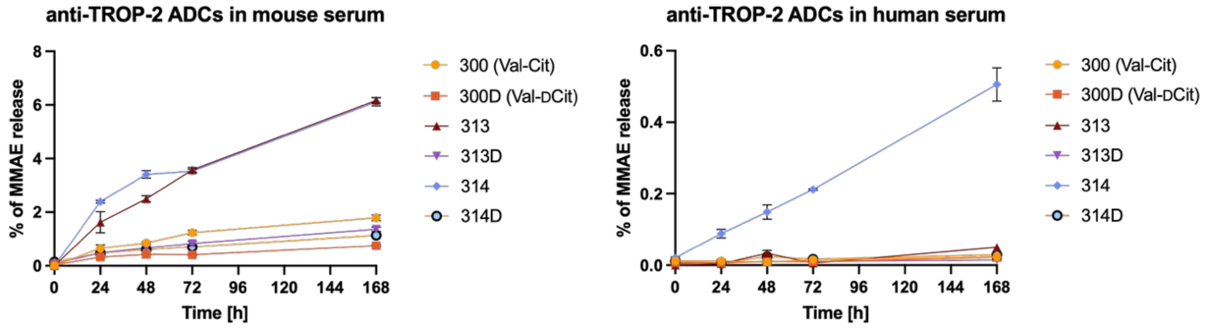

Figure S5. ADC stability in serum. Stability of anti-TROP-2 sacituzumab-based ADCs in mouse and human serum. The y-axis represents the percentage of free MMAE released into the serum, and the x-axis shows the time of incubation (24, 48, 72, and 168 h).

Table S11. Metal-conjugated antibody panel for mass cytometry profiling of breast tumor architecture. This panel includes antibodies targeting markers for major cell populations within the tumor microenvironment, including apoptotic cells, immune cells, epithelial cells, fibroblasts, and endothelial cells. The table also outlines the gating strategies used to define each cell type, providing a framework for high-resolution single-cell identification and population profiling by CyTOF.

**Cell type**	**antibody**	**metal**
Apoptotic cells (**cPARP+, cCasp3+)**	Anti-cPARP	143Nd
	Anti-cl. Casp-3	142Ce
Granulocytes (**CD66b+**)	Anti-CD66b	113Cd
Lymphocytes (**CD45+**, CD66b-)	Anti-CD45	89Y
B cells (CD45+, **CD19+**)	Anti-CD19	165Ho
T cells (CD45+, **CD3+**)	Anti-CD3	154Sm
CD4+ T cells (CD45+, CD3+, **CD4+**, CD8-)	Anti-CD4	110Cd
CD8+ T cells (CD45+, CD3+, CD4-, **CD8+**)	Anti-CD8	114Cd
Macrophages (CD45+, CD3-, CD19-, **CD11b+**)	Anti-CD11b	209Bi
NK cells (CD45+, CD3-, CD19-, **CD56+**)	Anti-CD56	176Yb
Other lymphocytes (CD45+, CD3-, CD19-, CD11b-, CD56-)	-	-
Epithelial cells (CD45-, CD66b-, **EpCAM+** or **Cadh3+**)	Anti-EpCAM	141Pr
	Anti-Cadherin-3	172Yb
Fibroblasts (CD45-, CD66b-, EpCAM-, Cadh3-, **FAP+ or SMA+**)	Anti-FAP	164Dy
	Anti-SMA	161Dy
Endothelial (CD45-, CD66b-, EpCAM-, Cadh3-, FAP+, SMA-, **CD31+**)	Anti-CD31	144Nd
Other non-immune (all markers negative)	-	-

Table S12. Metal-conjugated antibody panel for detection of tumor-associated biomarkers and cathepsin S by mass cytometry. This panel includes antibodies specific for key breast cancer markers such as HER2, estrogen receptor (ER), and progesterone receptor (PR), enabling molecular subtyping of tumor cells. Additionally, the panel features a metal-conjugated antibody against cathepsin S, allowing single-cell resolution profiling of protease expression within the tumor microenvironment.

**Protein/hormone target**	**antibody**	**metal**
HER-2	Anti-HER-2	113Cd
Progesterone (PR)	Anti-PR	145Nd
Estrogen (ER)	Anti-ER	163Dy
Cathepsin S	Anti-Cat S	155Gd
Cathepsin B	Anti-Cat B	162Dy

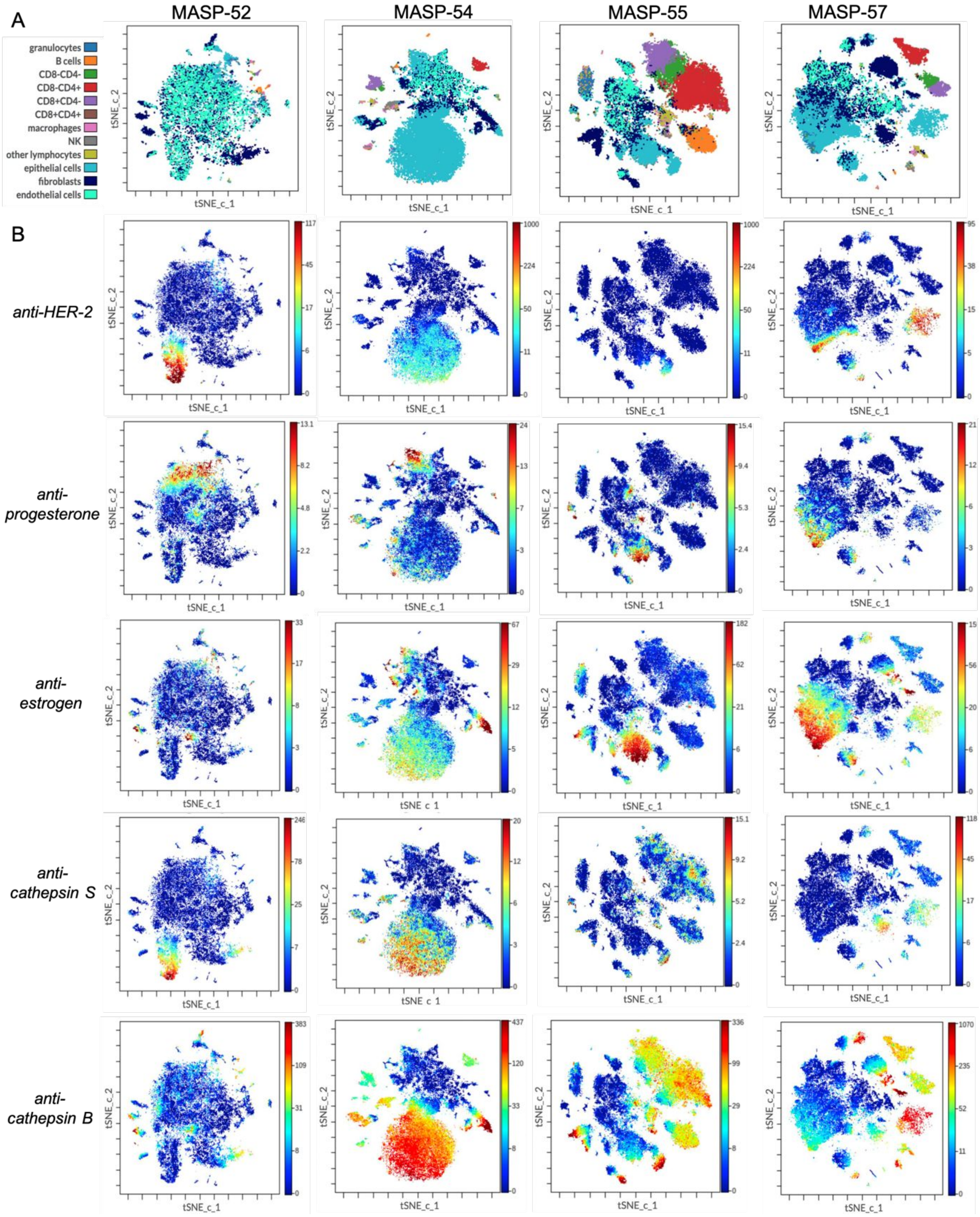

Figure S6. Single-cell mass cytometry profiling of the breast tumor microenvironment. Freshly resected breast tumors were enzymatically dissociated into single-cell suspensions and analyzed by mass cytometry (CyTOF) to generate high-dimensional cellular landscapes. **(A)** viSNE analysis revealed marked intratumoral heterogeneity among patient samples. While epithelial and endothelial cells represented the dominant populations across all tumors, the abundance of tumor-infiltrating immune cells varied significantly, with some patients exhibiting substantial immune infiltration. **(B)** viSNE-based marker distribution maps showed high co-expression of HER2 with cathepsin S, whereas correlations with PR and ER were only moderate. HER2 expression was primarily restricted to epithelial cells, while cathepsin S was detected in both epithelial cells and infiltrating immune cells. These findings support the rationale for developing protease-selective ADCs that exploit cathepsin S activity in HER2-positive tumors or enable extracellular activation in immune cell-rich tumor microenvironments.

Table S13. The LC-MS analysis of substrates, inhibitors, activtiy-based probes and prodrugs for cathepsin S.

Code	Compound structure	$[M+H]^+$ calculated	$[M+H]^+$ measured
**OG-64**	Ac-Phe(F_5)-Cit-2Aoc-Arg-ACC	952,41	952,48
**OG-65**	Ac-Phe(F_5)-Idc-2Aoc-Arg-ACC	940,38	940,44
**OG-66**	Ac-Phe(F_5)-Thz-Lys(2ClZ)-Arg-ACC	1065,31	1065,44
**OG-67**	Ac-Phe(F_5)-Cit-3Pal-Arg-ACC	958,37	958,41
**OG-68**	Ac-Phe(F_5)-Tic-2Aoc-Arg-ACC	954,39	954,45
**OG-69**	Ac-Phe(F_5)-Arg-Lys(2ClZ)-Arg-ACC	1106,40	1106,56
**OG-70**	HN-Idc-Cit-Leu-Arg-ACC	788,38	788,46
**OG-71**	HN-Idc-Thz-Leu-Arg-ACC	744,30	744,36
**OG-72**	HN-Idc-Ile-3Pal-Arg-ACC	779,36	779,45
**OG-73**	HN-Idc-Tic-2Aoc-Arg-ACC	818,40	818,48
**OG-74**	HN-Idc-Arg-Leu-Arg-ACC	787,39	787,48
**OG-75**	Ac-Pro-Arg-Leu-Arg-ACC	784,42	784,50
**OG-76**	Ac-Pro-Ile-3Pal-Arg-ACC	775,38	775,45
**OG-77**	Ac-Pro-Thz-Lys(2ClZ)-Arg-ACC	925,34	925,42
**OG-79**	Ac-Pro-Arg-2Aoc-Arg-ACC	811,45	811,53
**OG-80**	Ac-Pro-Arg-Hyp(Bzl)-Arg-ACC	873,43	873,52
**OG-81**	Ac-Tle-Cit-2Aoc-Arg-ACC	828,47	828,54
**OG-82**	HN-Idc-Lys(2ClZ)-Arg-ACC	814,30	814,36
**OG-83**	Ac-Tle-Thz-2Aoc-Arg-ACC	786,39	786,47
**OG-84**	Ac-Tle-Ile-3Pal-Arg-ACC	791,41	791,50
**OG-85**	Ac-Tle-Arg-Hyp(Bzl)-Arg-ACC	889,46	889,53
**OG-86**	Ac-Tle-Arg-2Aoc-Arg-ACC	827,48	827,58
**OG-87**	Ac-Asp(Me)-Ile-3Pal-Arg-ACC	807,37	807,47
**OG-88**	Ac-Asp(Me)-Tic-Leu-Arg-ACC	818,38	818,45
**OG-89**	Ac-Asp-Tic-Hyp(Bzl)-Arg-ACC	894,37	894,44
**OG-90**	Ac-Asp(Me)-Thz-Lys(2ClZ)-Arg-ACC	957,33	957,38
**OG-91**	Ac-Met(O_2)-Cit-Hyp(Bzl)-Arg-ACC	940,39	940,43
**OG-93**	Ac-Met(O_2)-Thz-Lys(2ClZ)-Arg-ACC	991,31	991,40
**OG-94**	Ac-Met(O_2)-Ile-Hyp(Bzl)-Arg-ACC	896,39	896,46
**OG-95**	Ac-Bpa-Tic-Hyp(Bzl)-Arg-ACC	1030,44	1030,55
**OG-96**	Ac-Bpa-Arg-2Aoc-Arg-ACC	965,49	965,54

**OG-115**	Ac-1Nal-Cit-2Aoc-Arg-ACC	912,47	912,55
**OG-116**	Ac-Bpa-Cit-2Aoc-Arg-ACC	966,48	966,58
**OG-117**	Ac-Met(O_2)-Cit-2Aoc-Arg-ACC	878,41	878,48
**OG-118**	Ac-Leu-Cit-2Aoc-Arg-ACC	828,47	828,57
**OG-119**	Ac-Phe(F_5)-Cit-Leu-Arg-ACC	924,37	924,47
**OG-120**	Ac-Phe(F_5)-Cit-Nle-Arg-ACC	924,37	924,45
**OG-121**	Ac-Leu-Thz-Lys(2ClZ)-Arg-ACC	941,37	941,46
**OG-122**	Ac-Leu-Thz-hLeu-Arg-ACC	772,37	772,47
**OG-123**	Ac-Leu-Thz-NptGly-Arg-ACC	772,37	772,48
**OG-133**	Ac-Phe(F_5)-Cit-2Aoc-Gln-ACC	924,36	924,44
**OG-134**	Ac-Phe(F_5)-Cit-2Aoc-Nle-ACC	909,39	909,49
**OG-135**	Ac-Phe(F_5)-Cit-2Aoc-Glu(Me)-ACC	939,36	939,44
**OG-136**	Ac-Phe(F_5)-Cit-2Aoc-Cit-ACC	953,39	953,48
**OG-137**	Ac-Phe(F_5)-Cit-2Aoc-Lys(TFA)-ACC	1020,38	1020,51
**OG-138**	Ac-Phe(F_5)-Cit-2Aoc-Thr(Bzl)-ACC	987,40	987,50
**OG-139**	Ac-Phe(F_5)-Cit-2Aoc-Cys(MeOBzl)-ACC	1019,37	1019,50
**OG-140**	Ac-Phe(F_5)-Cit-2Aoc-Cys(MeBzl)-ACC	1003,37	1003,46
**OG-141**	Ac-Phe(F_5)-Cit-2Aoc-hTyr(Me)-ACC	987,40	987,48
**OG-142**	Ac-Phe(F_5)-Cit-2Aoc-Nle(OBzl)-ACC	1015,43	1015,56
**OG-143**	Ac-Phe(F_5)-Cit-2Aoc-Met-ACC	927,34	927,43
**OG-144**	Ac-Phe(F_5)-Cit-2Aoc-Abu-ACC	881,35	881,42
**OG-145**	Ac-Phe(F_5)-Cit-2Aoc-Nva-ACC	895,37	895,46
**OG-146**	Ac-Phe(F_5)-Cit-2Aoc-hLeu-ACC	923,40	923,52
**OG-147**	Ac-Phe(F_5)-Cit-2Aoc-2Aoc-ACC	937,42	937,50
**OG-191**	Ac-Phe(F_5)-Cit-NptGly-Arg-ACC	938,39	938,43
**OG-192**	Ac-Phe(F_5)-Cit-NptGly-Gln-ACC	910,34	910,39
**OG-193**	Ac-Phe(F_5)-Cit-NptGly-Glu(Me)-ACC	925,34	925,41
**OG-194**	Ac-Phe(F_5)-Cit-NptGly-Cit-ACC	939,37	939,41
**OG-195**	Ac-Phe(F_5)-Cit-Lys(2ClZ)-Arg-ACC	1107,38	1107,54
**OG-196**	Ac-Phe(F_5)-Cit-Lys(2ClZ)-Gln-ACC	1079,34	1079,44
**OG-197**	Ac-Phe(F_5)-Cit-Lys(2ClZ)-Glu(Me)-ACC	1094,34	1094,51
**OG-198**	Ac-Phe(F_5)-Cit-Lys(2ClZ)-Cit-ACC	1108,36	1108,51
**OG-199**	Ac-Phe(F_5)-Thz-2Aoc-Arg-ACC	910,33	910,39
**OG-200**	Ac-Phe(F_5)-Thz-2Aoc-Glu-ACC	883,27	883,33

**OG-201**	Ac-Phe(F_5)-Thz-2Aoc-Gln-ACC	882,28	882,34
**OG-202**	Ac-Phe(F_5)-Thz-2Aoc-Cit-ACC	911,31	911,37
**OG-203**	Ac-Met(O_2)-Cit-2Aoc-Arg-ACC	878,41	878,47
**OG-204**	Ac-Met(O_2)-Cit-2Aoc-Gln-ACC	850,37	850,47
**OG-205**	Ac-Met(O_2)-Cit-2Aoc-Glu(Me)-ACC	865,37	865,44
**OG-206**	Ac-Met(O_2)-Cit-2Aoc-Cit-ACC	879,40	879,46
**OG-207**	Ac-Met(O_2)-Cit-NptGly-Arg-ACC	864,40	864,49
**OG-208**	Ac-Met(O_2)-Cit-NptGly-GlnA-CC	836,35	836,45
**OG-209**	Ac-Met(O_2)-Cit-NptGly-Glu(Me)-ACC	851,35	851,42
**OG-210**	Ac-Met(O_2)-Cit-NptGly-Cit-ACC	865,35	865,46
**JN-1**	Ac-Met(O_2)-Cit-NptGly-Glu(Me)-AOMK	797,37	797,46
**JN-2**	Ac-Met(O_2)-Cit-NptGly-Lys(2ClZ)-AOMK	950,40	950,47
**JN-3**	Ac-Met(O_2)-Cit-NptGly-Arg-AOMK	810,41	810,47
**JN-4**	Ac-Met(O_2)-Cit-NptGly-Cys(Bzl)-AOMK	847,37	847,40
**JN-5**	Ac-Met(O_2)-Cit-NptGly-Cys(MeBzl)-AOMK	861,38	861,39
**JN-6**	Ac-Met(O_2)-Cit-NptGly-Nle(OBzl)-AOMK	873,44	873,42
**JN-7**	Ac-Phe(F_5)-Cit-Lys(2ClZ)-Glu(Me)-AOMK	1040,35	1040,39
**JN-8**	Ac-Phe(F_5)-Cit-Lys(2ClZ)-Lys(2ClZ)-AOMK	1193,39	1193,47
**JN-9**	Ac-Phe(F_5)-Cit-Lys(2ClZ)-Arg-AOMK	1053,39	1053,45
**JN-10**	Ac-Phe(F_5)-Cit-Lys(2ClZ)-Cys(Bzl)-AOMK	1090,35	1090,44
**JN-11**	Ac-Phe(F_5)-Cit-Lys(2ClZ)-Cys(MeBzl)-AOMK	1104,37	1104,53
**JN-12**	Ac-Phe(F_5)-Cit-Lys(2ClZ)-Nle(OBzl)-AOMK	1116,42	1116,54
**OG-233**	Cy5-PEG(4)-Phe(F_5)-Cit-Lys(2ClZ)-Glu(Me)-AOMK	1709,7<br>855,39 $[M+H]^{2+}$	855,72 $[M+H]^{2+}$
**OG-235**	BODIPY-PEG(4)-Phe(F_5)-Cit-Lys(2ClZ)-Glu(Me)-AOMK	1518,59<br>760,30 $[M+H]^{2+}$	760,58 $[M+H]^{2+}$
**OG-234**	Cy5-PEG(4)-Met(O_2)-Cit-NptGly-Glu(Me)-AOMK	1466,79<br>733,90 $[M+H]^{2+}$	733,35 $[M+H]^{2+}$
**OG-236**	BODIPY-PEG(4)-Met(O_2)-Cit-NptGly-Glu(Me)-AOMK	1275,61<br>638,81 $[M+H]^{2+}$	638,00 $[M+H]^{2+}$
**OG-313**	Ac-Phe(F_5)-Cit-Lys(2ClZ)-Glu(Me)-PABC-MMAE	1742,83<br>871,92 $[M+H]^{2+}$	872,33 $[M+H]^{2+}$
**OG-314**	Ac-Met(O_2)-Cit-NptGly-Glu(Me)-PABC-MMAE	1499,84<br>750,42 $[M+H]^{2+}$	750,83 $[M+H]^{2+}$
**OG-352**	Ac-Phe(F_5)-Cit-Lys(2ClZ)-DGlu(Me)-PABC-MMAE	1742,83<br>871,92 $[M+H]^{2+}$	872,12 $[M+H]^{2+}$
**Z-Val-Cit**	Cbz-Val-Cit-PABC-MMAE	1257,74<br>629,38 $[M+H]^{2+}$	629,77 $[M+H]^{2+}$

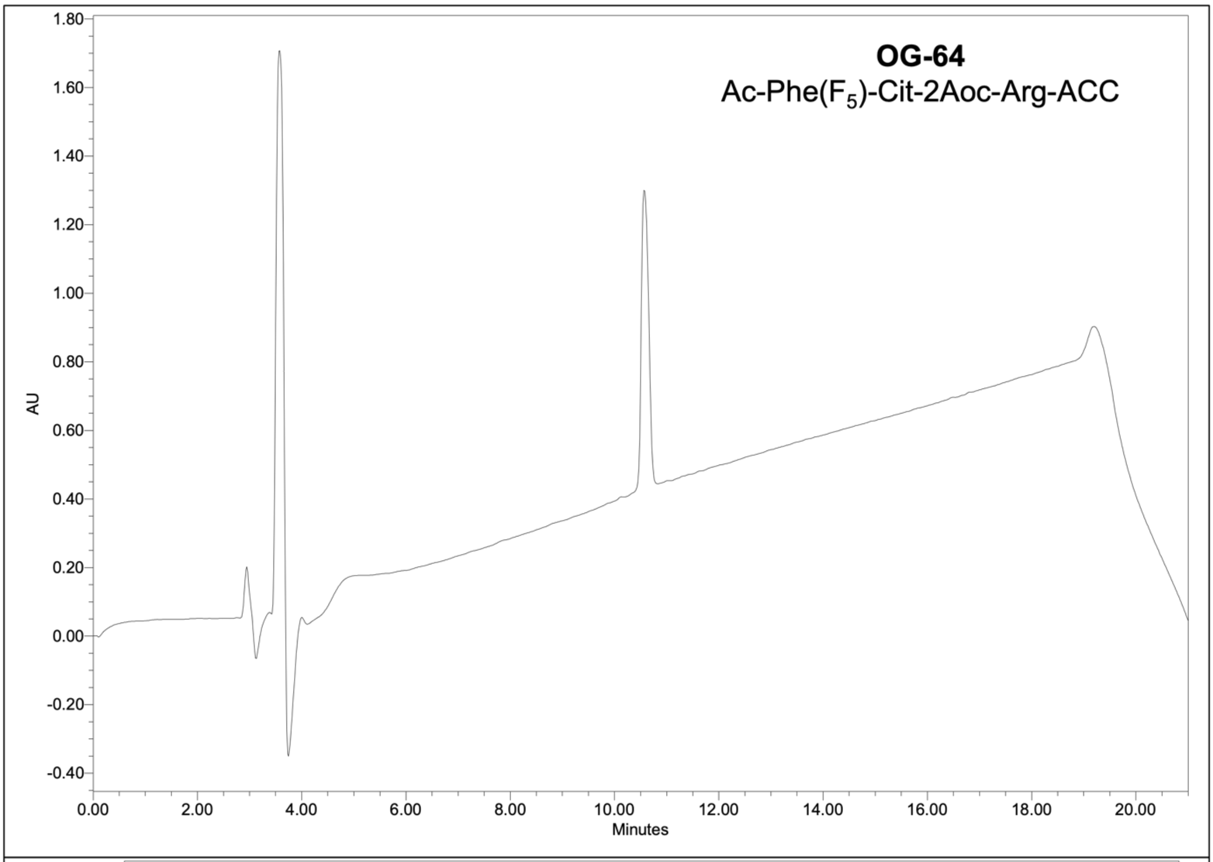
OG-64
Ac-Phe(F5)-Cit-2Aoc-Arg-ACC
AU
Minutes

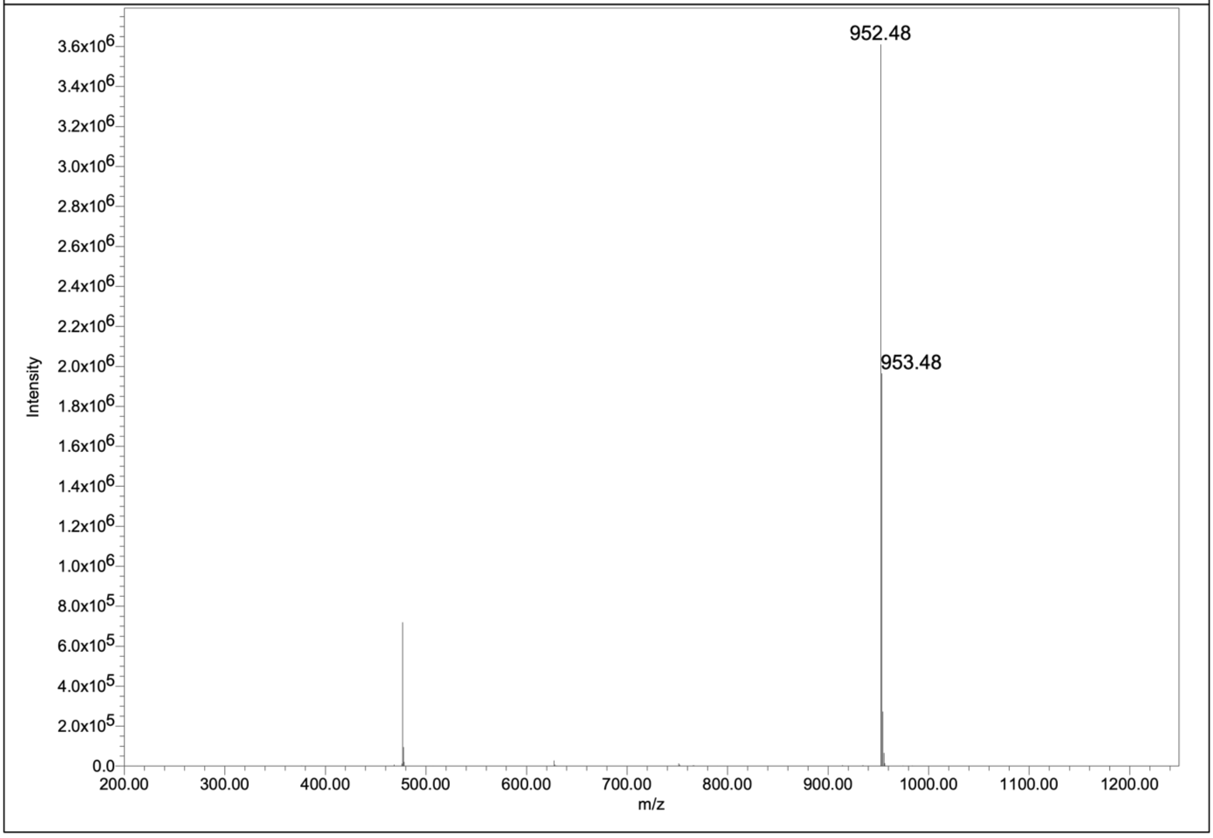
952.48
953.48
Intensity
m/z

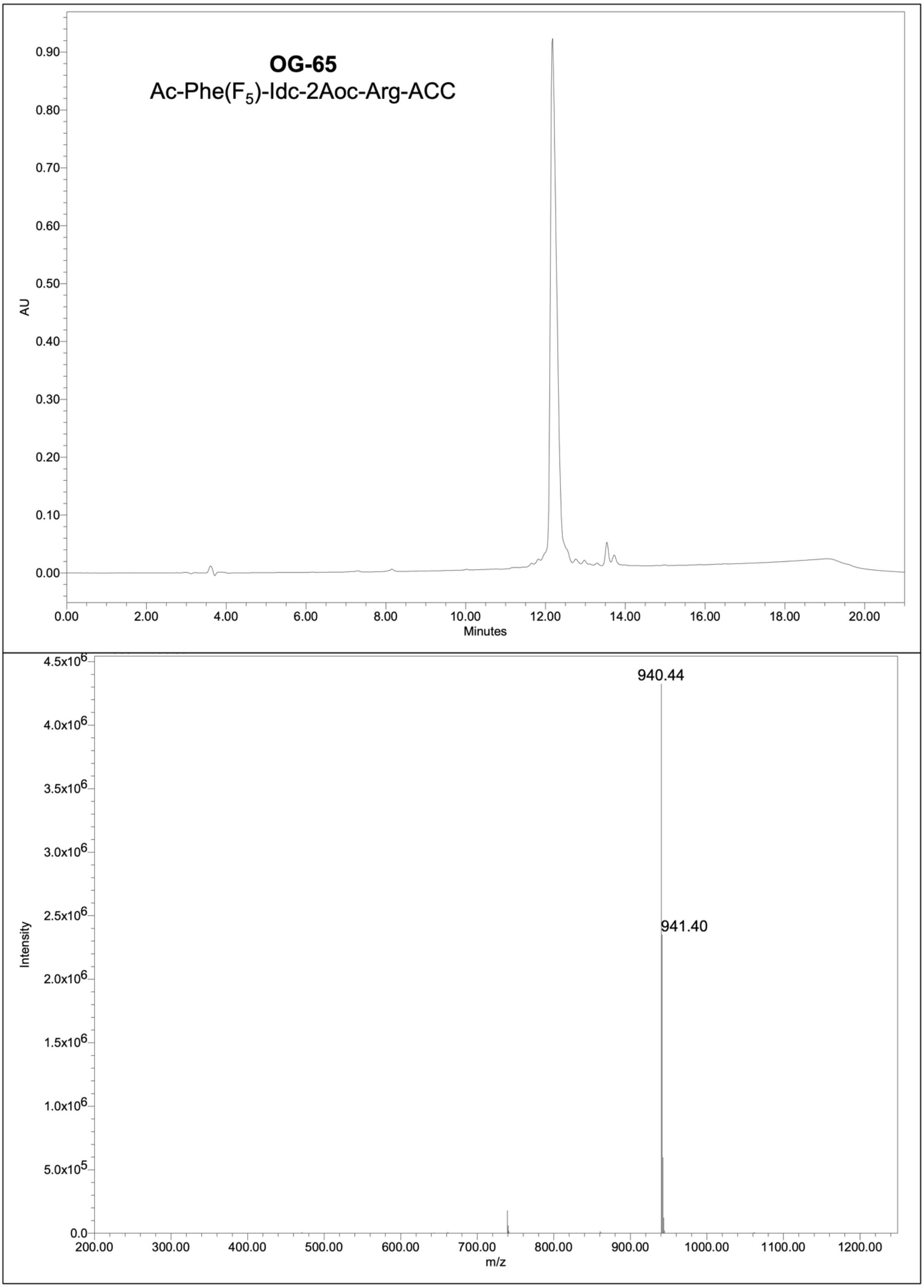
OG-65
Ac-Phe(F5)-Idc-2Aoc-Arg-ACC
AU
Minutes
940.44
941.40
Intensity
m/z

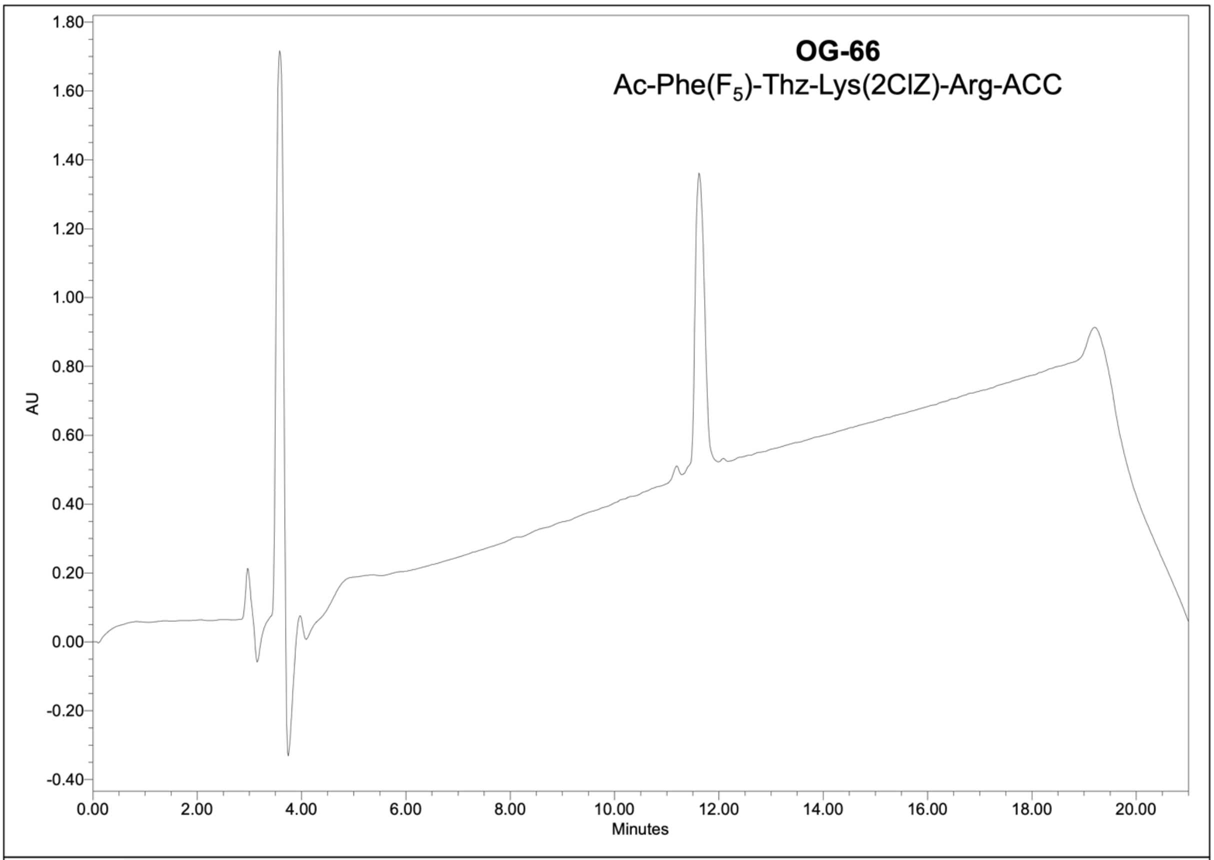
OG-66
Ac-Phe(F5)-Thz-Lys(2ClZ)-Arg-ACC
AU
1.80
1.60
1.40
1.20
1.00
0.80
0.60
0.40
0.20
0.00
-0.20
-0.40
0.00
2.00
4.00
6.00
8.00
10.00
12.00
14.00
16.00
18.00
20.00
Minutes

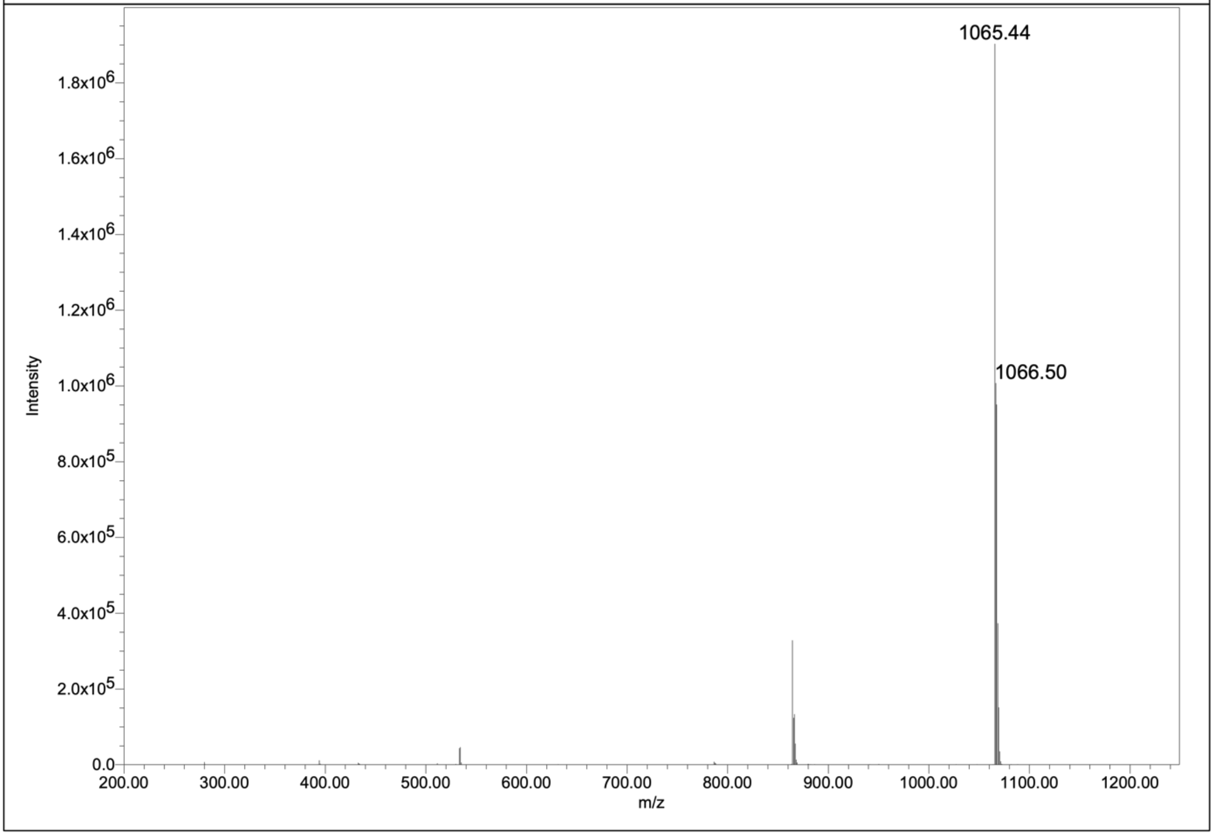
1065.44
1066.50
Intensity
1.8x10^6
1.6x10^6
1.4x10^6
1.2x10^6
1.0x10^6
8.0x10^5
6.0x10^5
4.0x10^5
2.0x10^5
0.0
200.00
300.00
400.00
500.00
600.00
700.00
800.00
900.00
1000.00
1100.00
1200.00
m/z

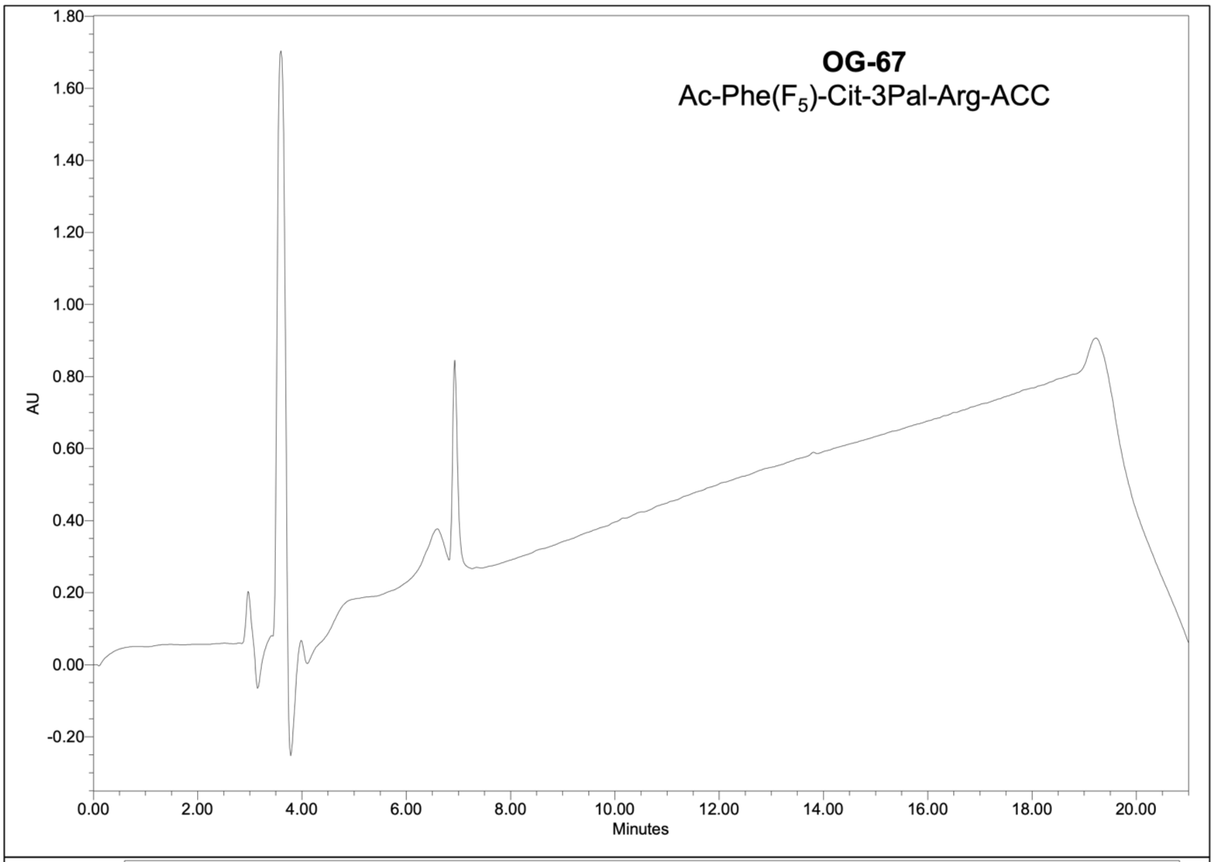

OG-67
Ac-Phe(F5)-Cit-3Pal-Arg-ACC
AU
Minutes

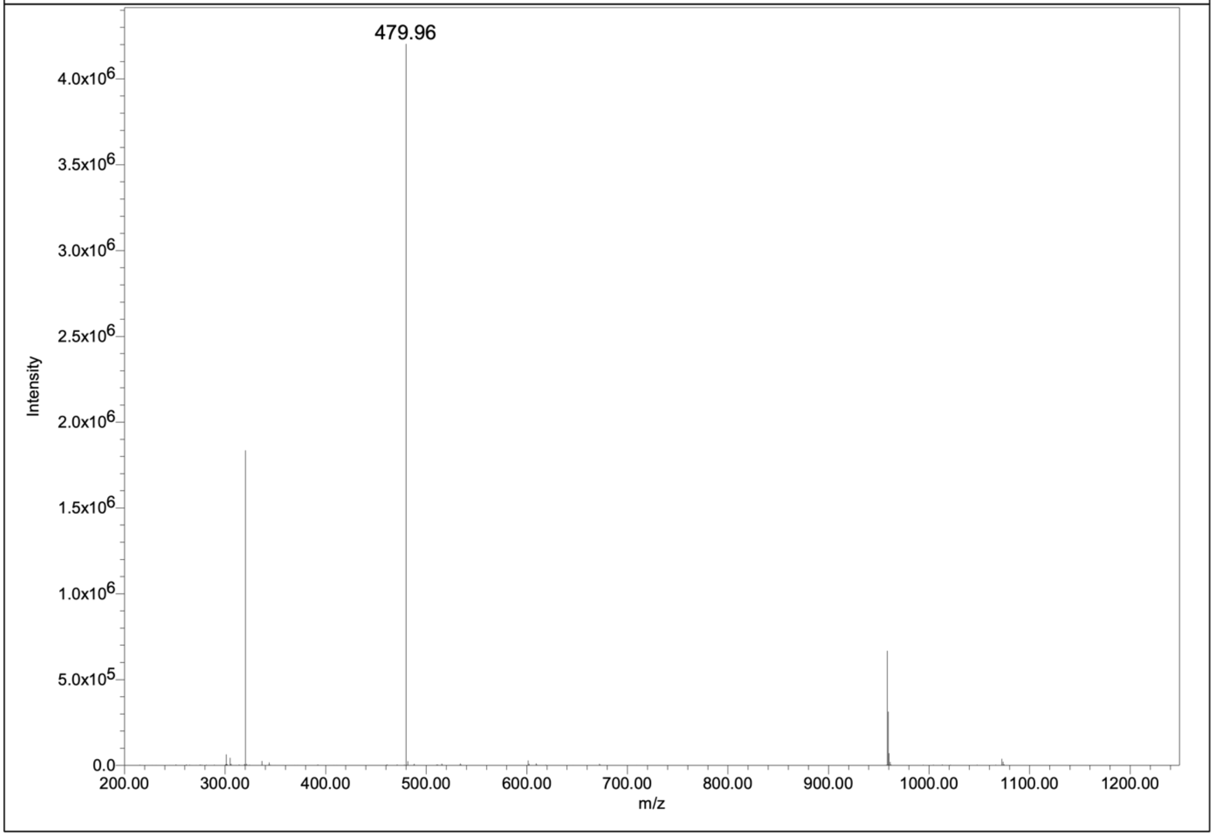

479.96
Intensity
m/z

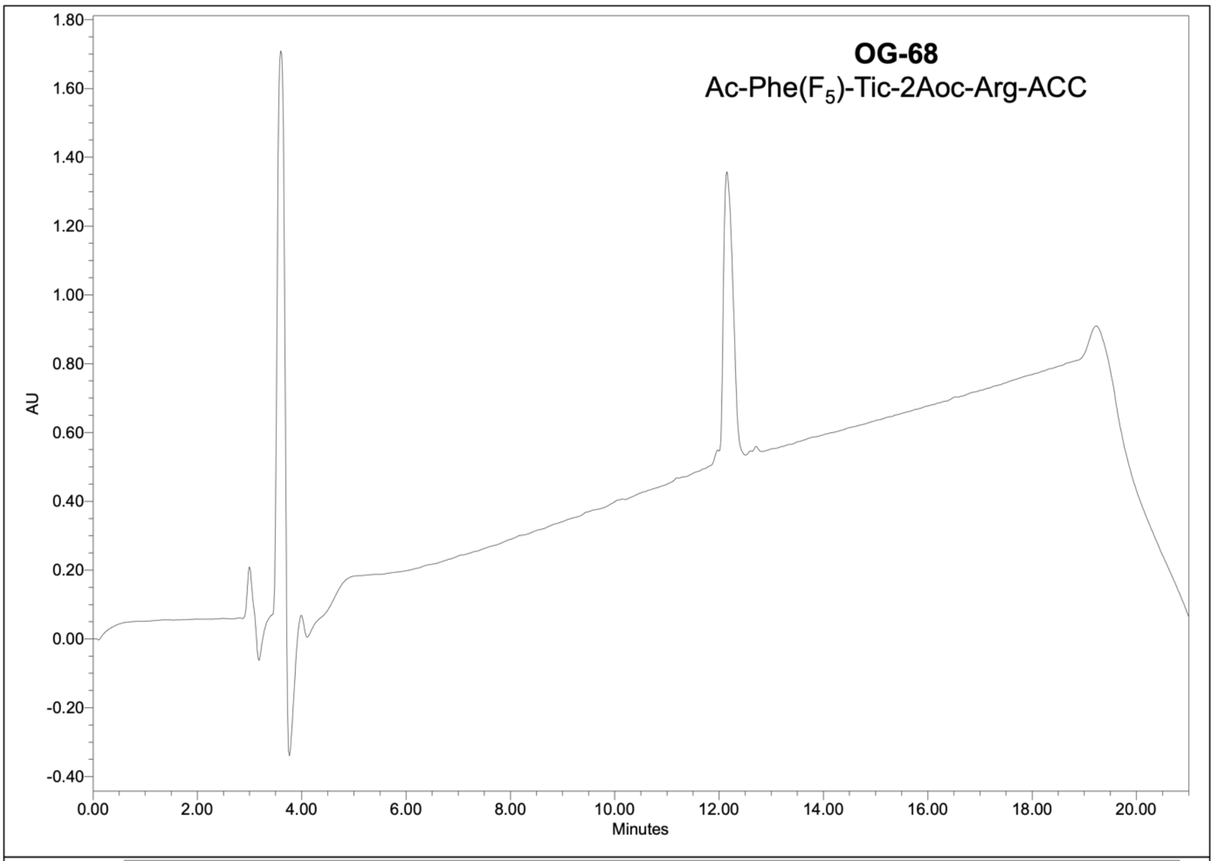

OG-68
Ac-Phe(F$_5$)-Tic-2Aoc-Arg-ACC
AU
Minutes

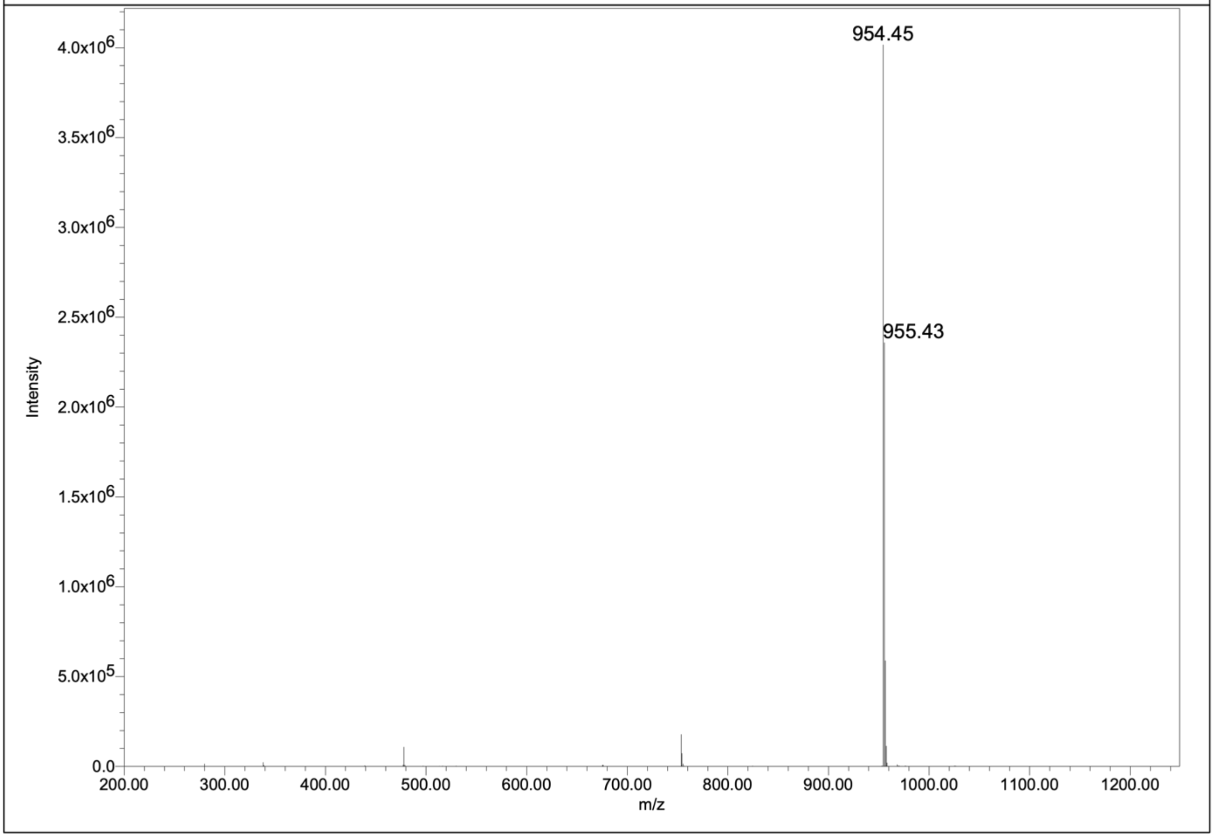

954.45
955.43
Intensity
m/z

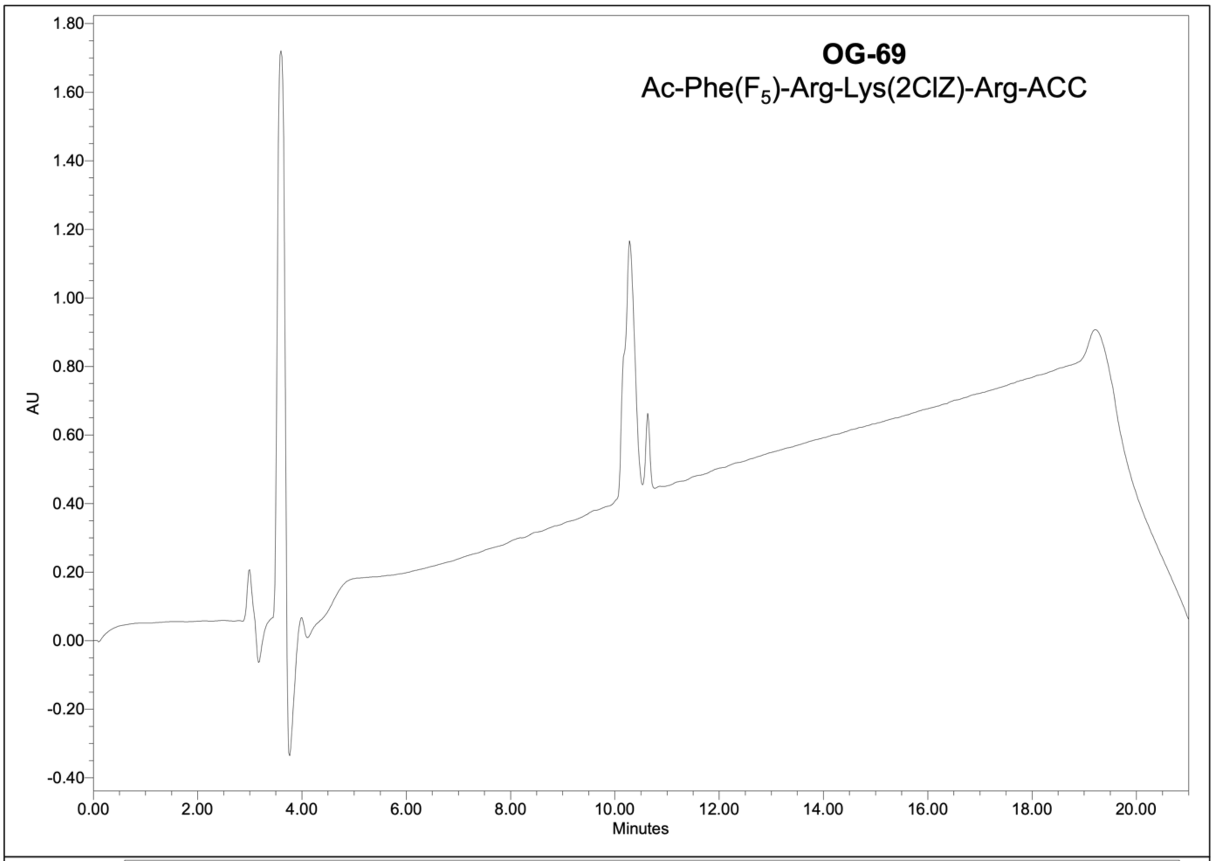
OG-69
Ac-Phe(F5)-Arg-Lys(2ClZ)-Arg-ACC
AU
Minutes

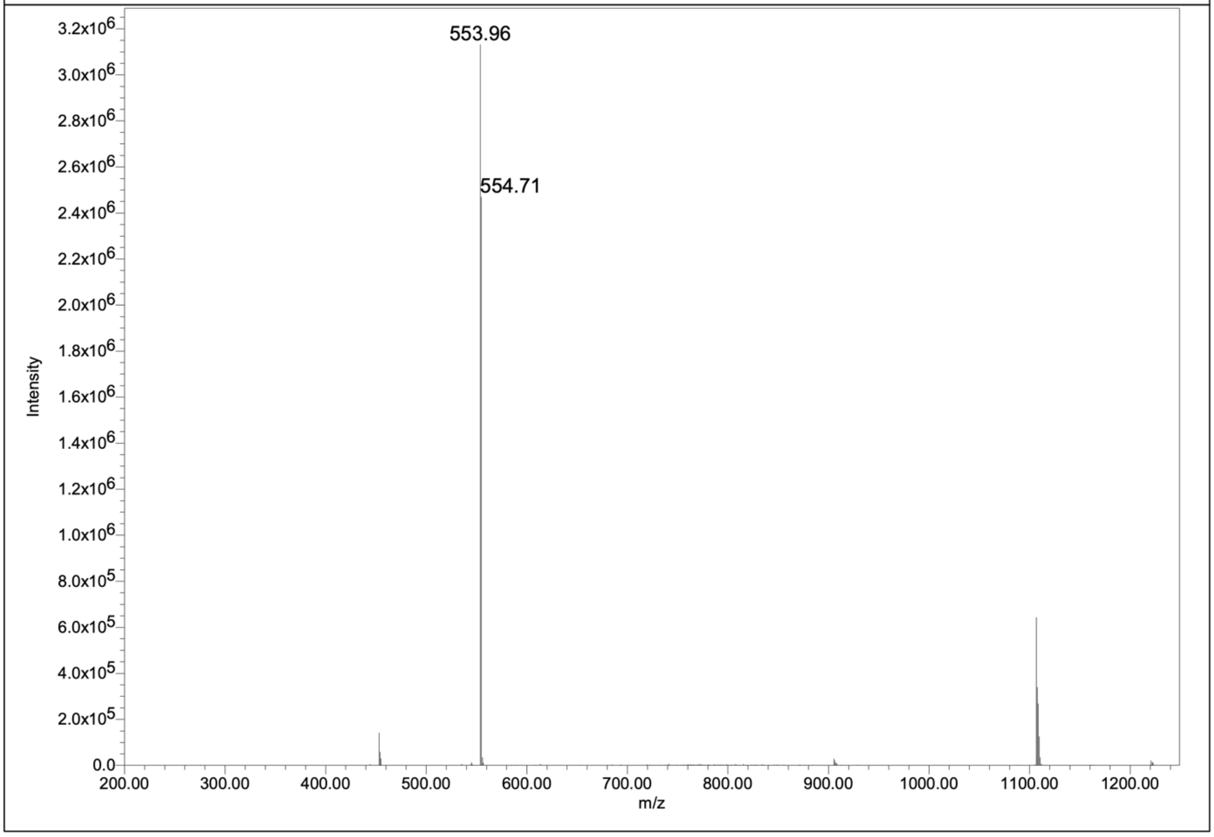
553.96
554.71
Intensity
m/z

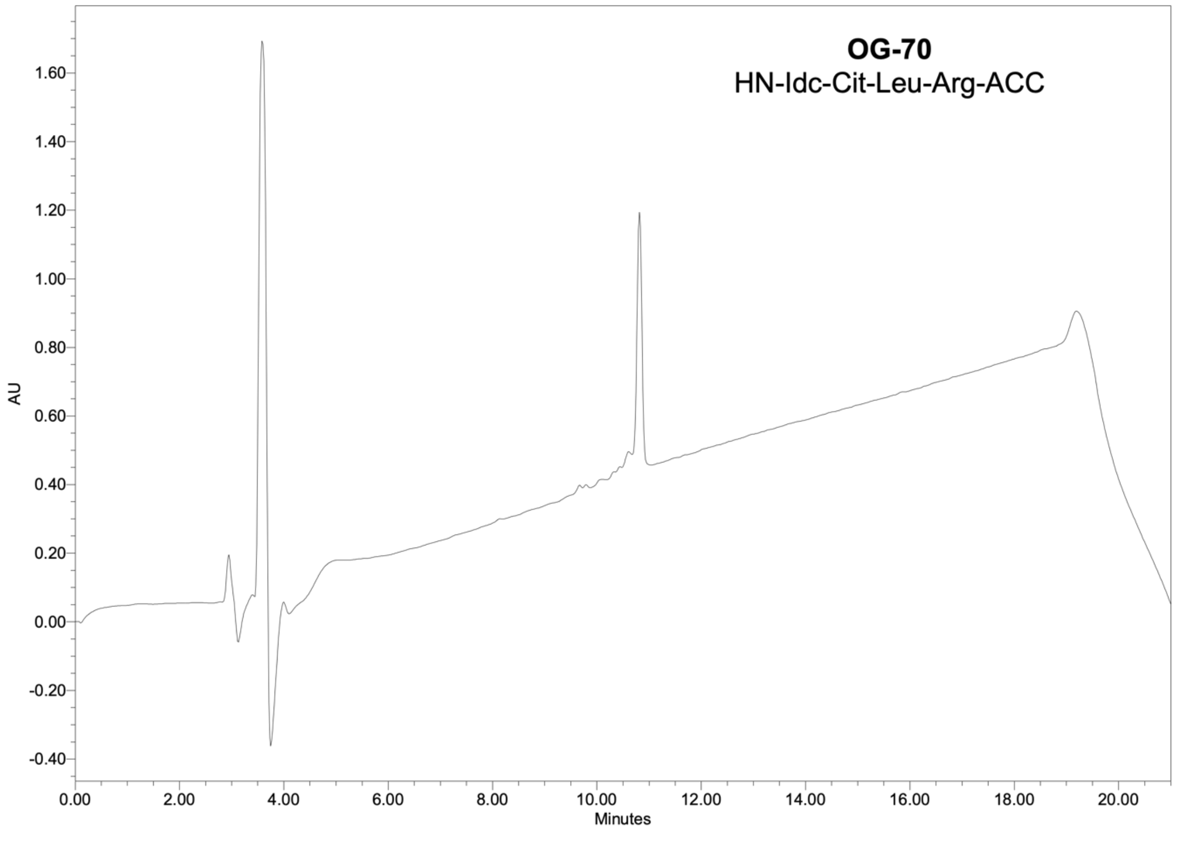
OG-70
HN-Idc-Cit-Leu-Arg-ACC
AU
Minutes
1.60
1.40
1.20
1.00
0.80
0.60
0.40
0.20
0.00
-0.20
-0.40
0.00
2.00
4.00
6.00
8.00
10.00
12.00
14.00
16.00
18.00
20.00

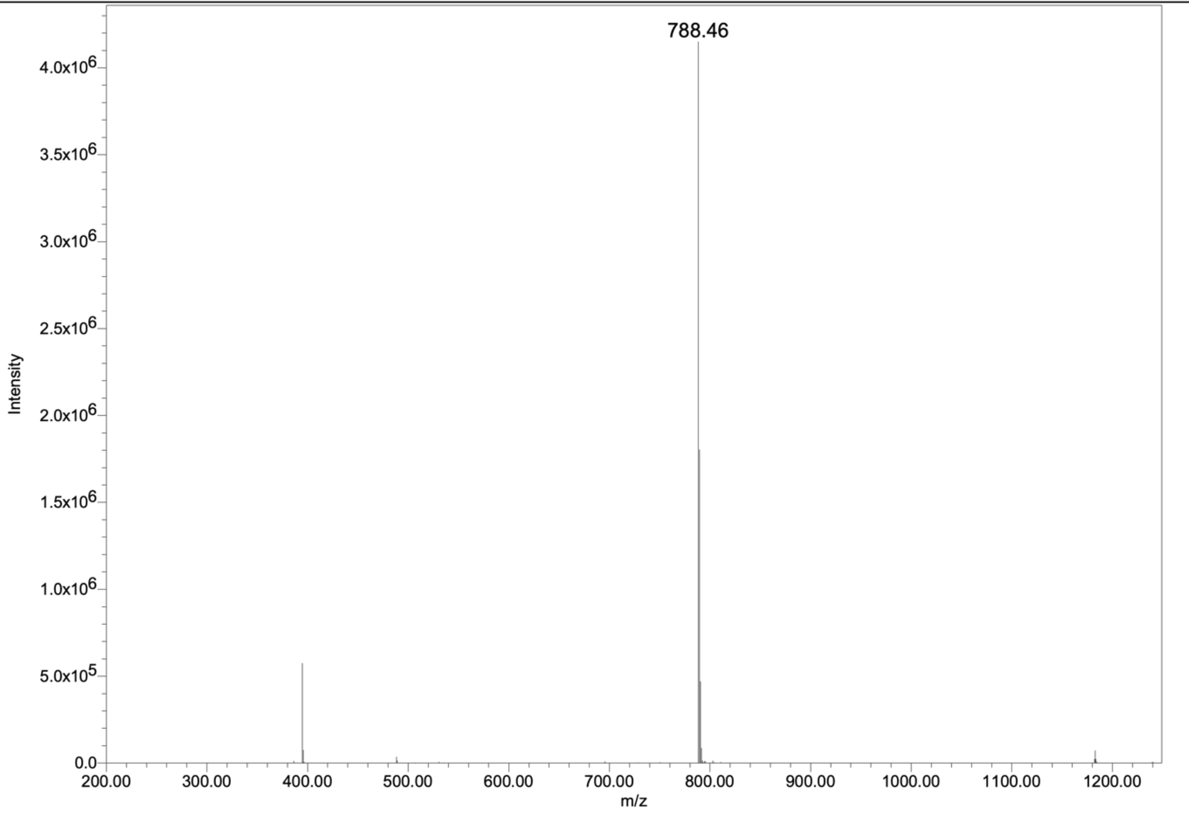
788.46
Intensity
m/z
4.0x10^6
3.5x10^6
3.0x10^6
2.5x10^6
2.0x10^6
1.5x10^6
1.0x10^6
5.0x10^5
0.0
200.00
300.00
400.00
500.00
600.00
700.00
800.00
900.00
1000.00
1100.00
1200.00

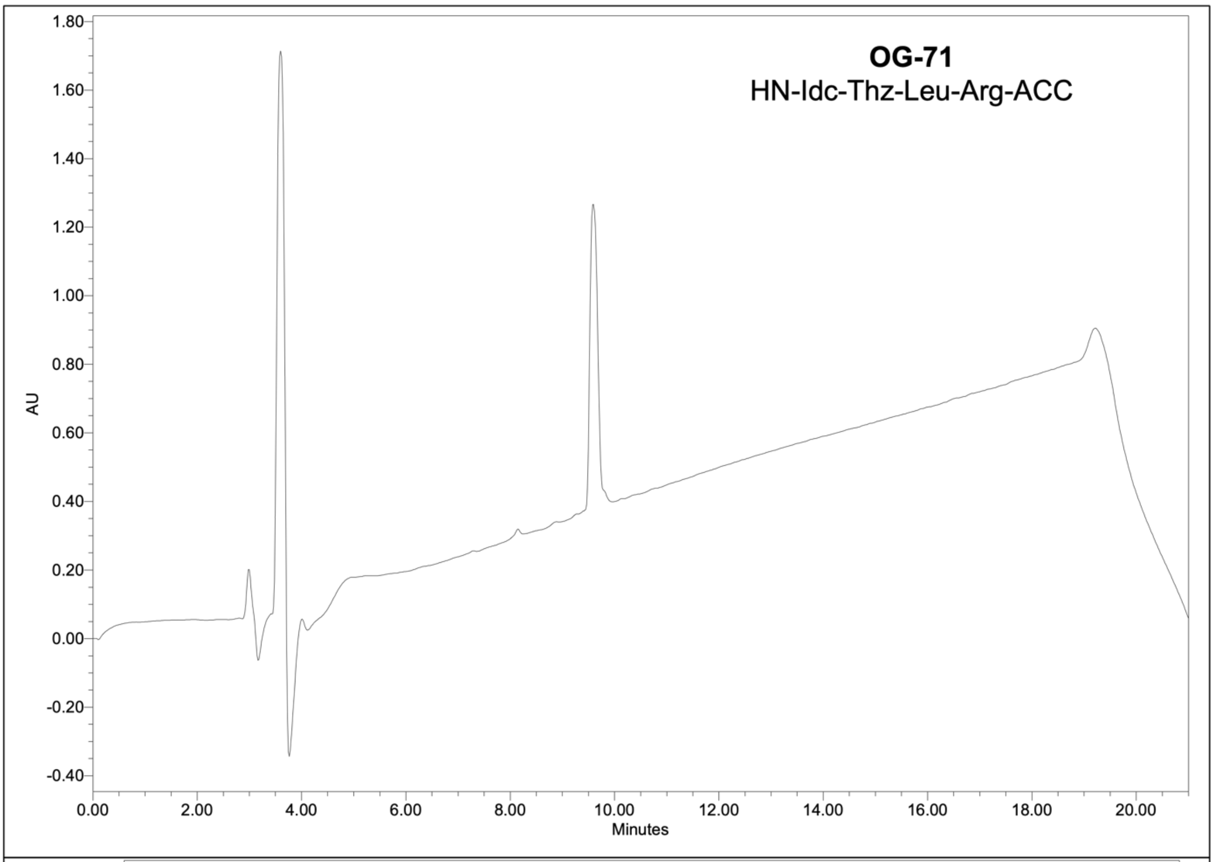

OG-71
HN-Idc-Thz-Leu-Arg-ACC
AU
Minutes

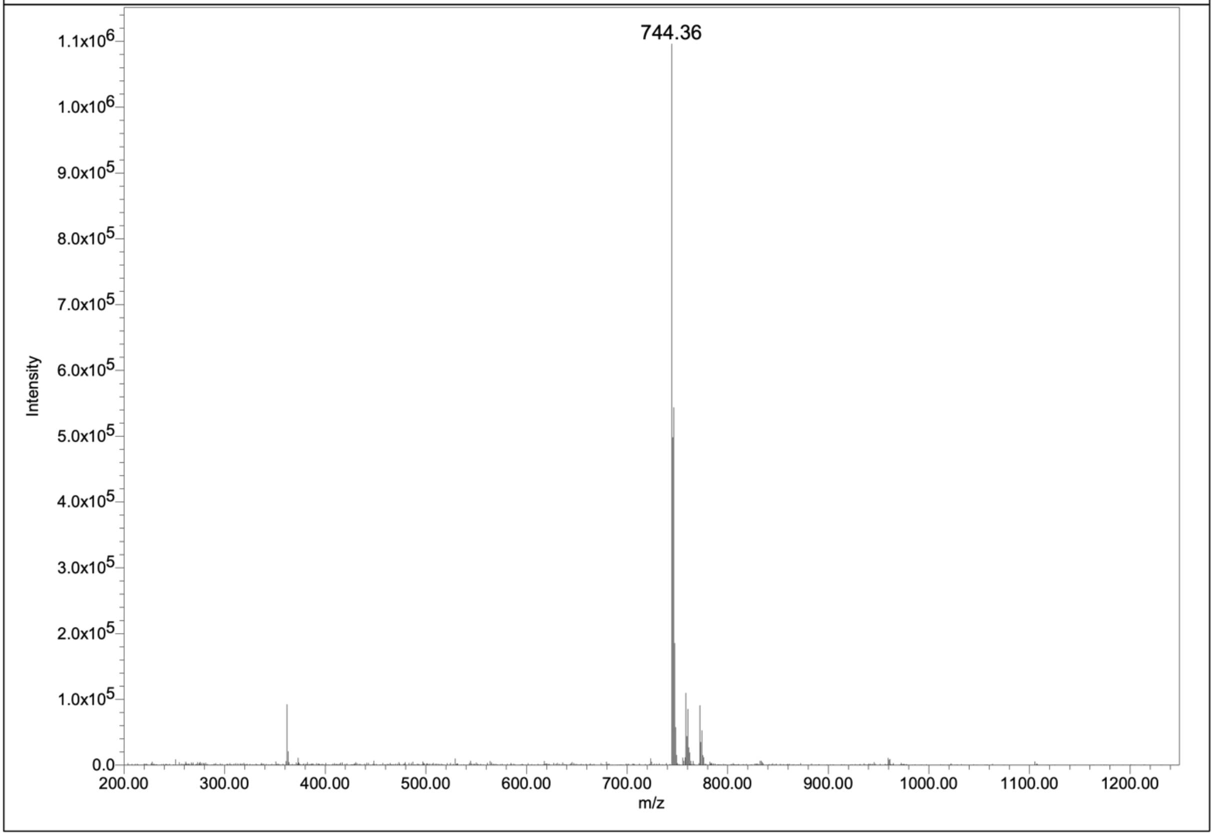

744.36
Intensity
m/z

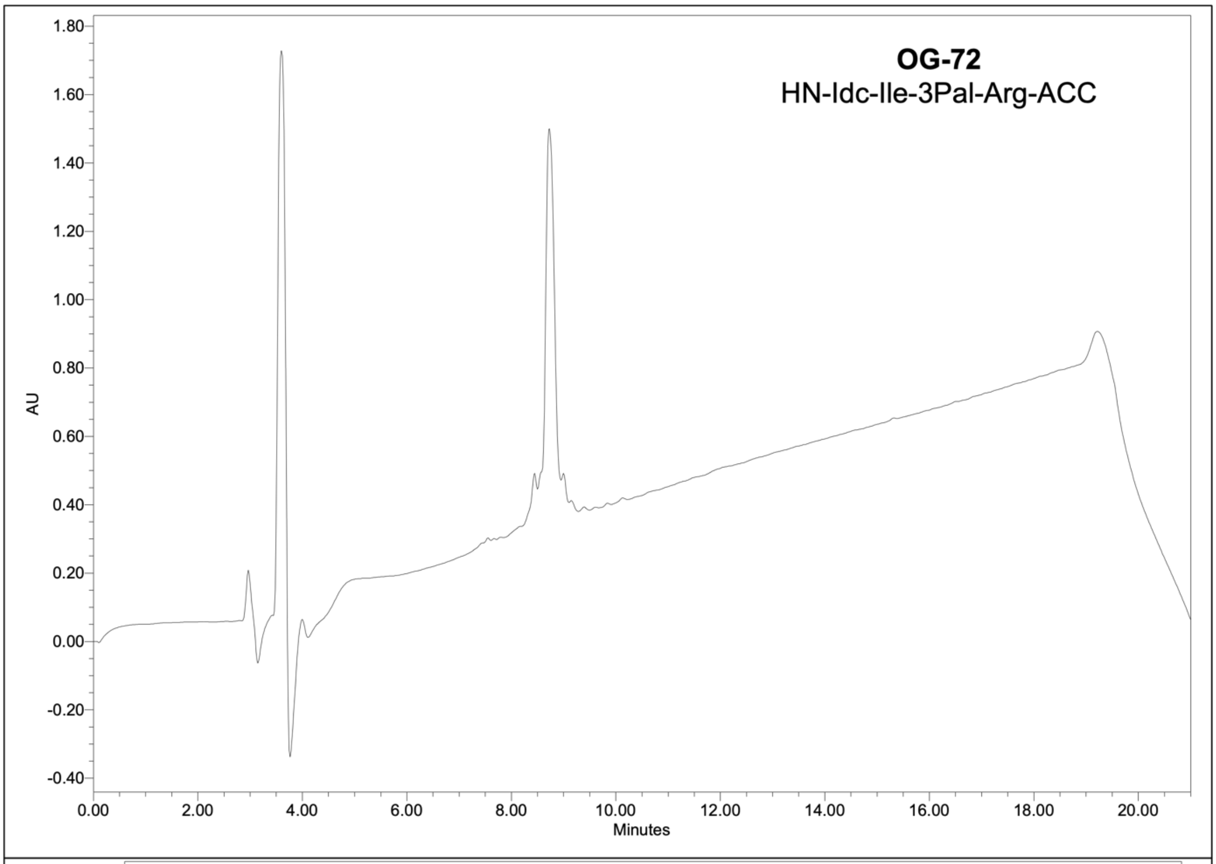
OG-72
HN-Idc-Ile-3Pal-Arg-ACC
AU
Minutes

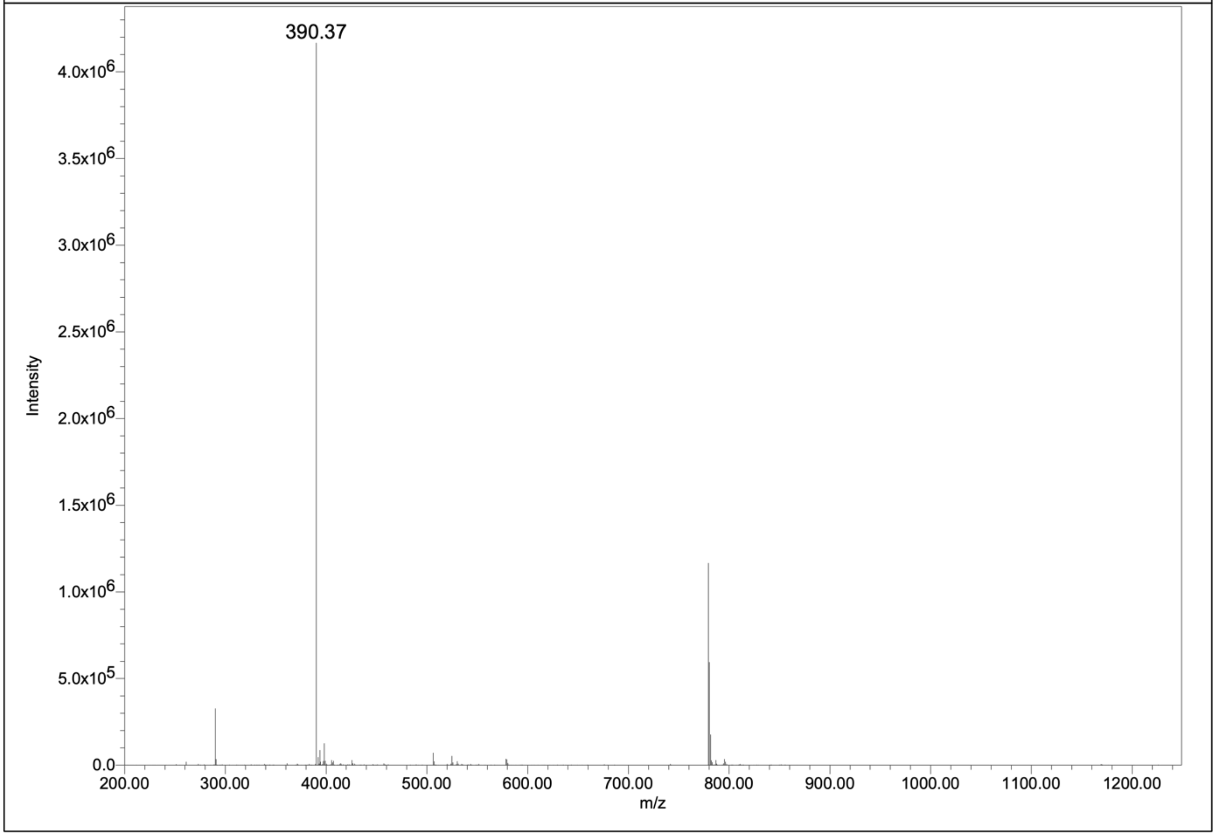
390.37
Intensity
m/z

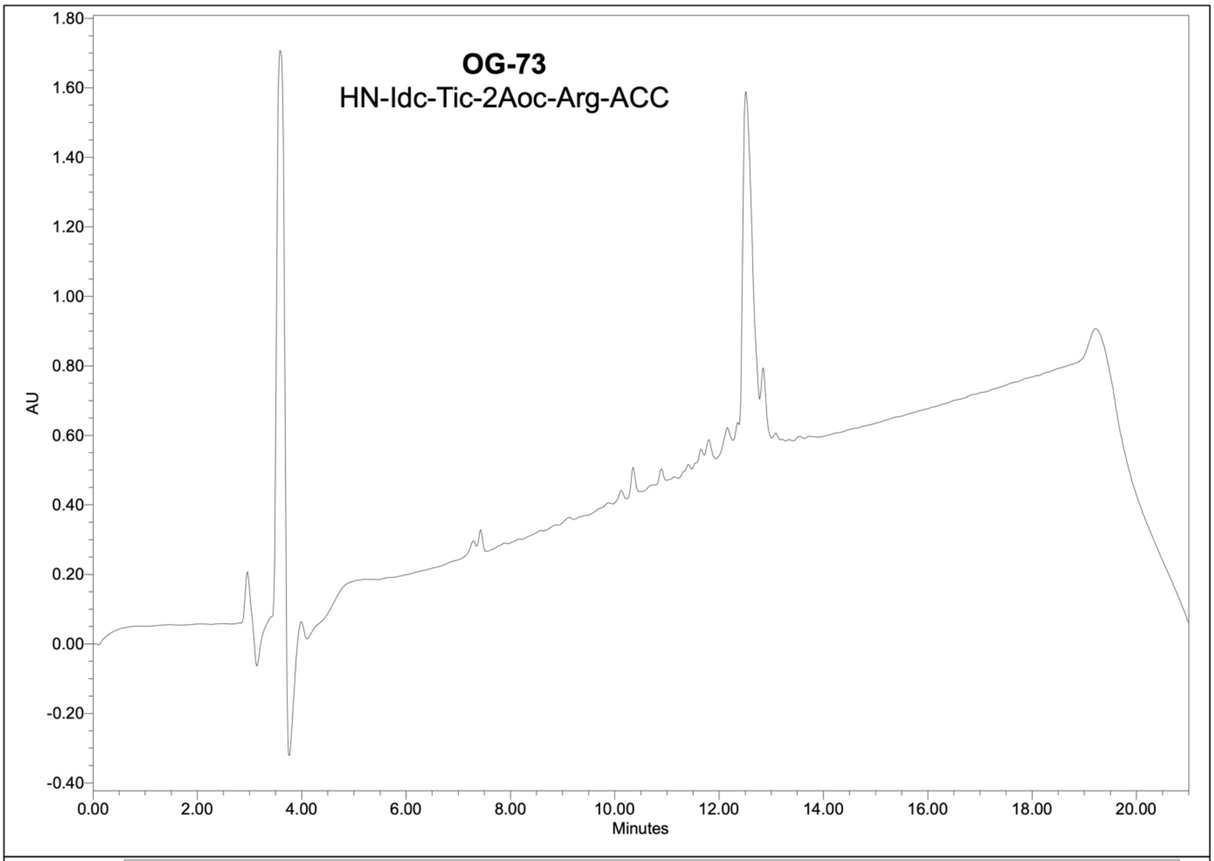
OG-73
HN-Idc-Tic-2Aoc-Arg-ACC
AU
Minutes

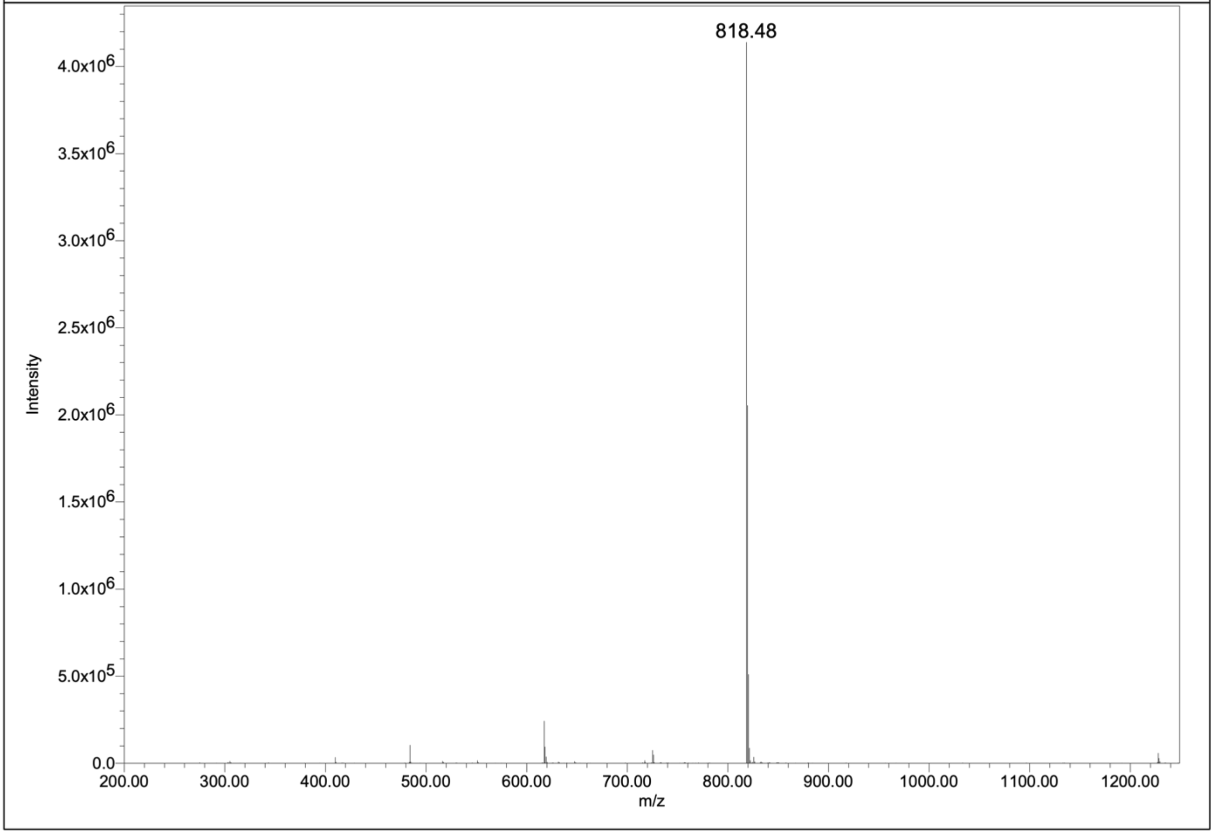
818.48
Intensity
m/z

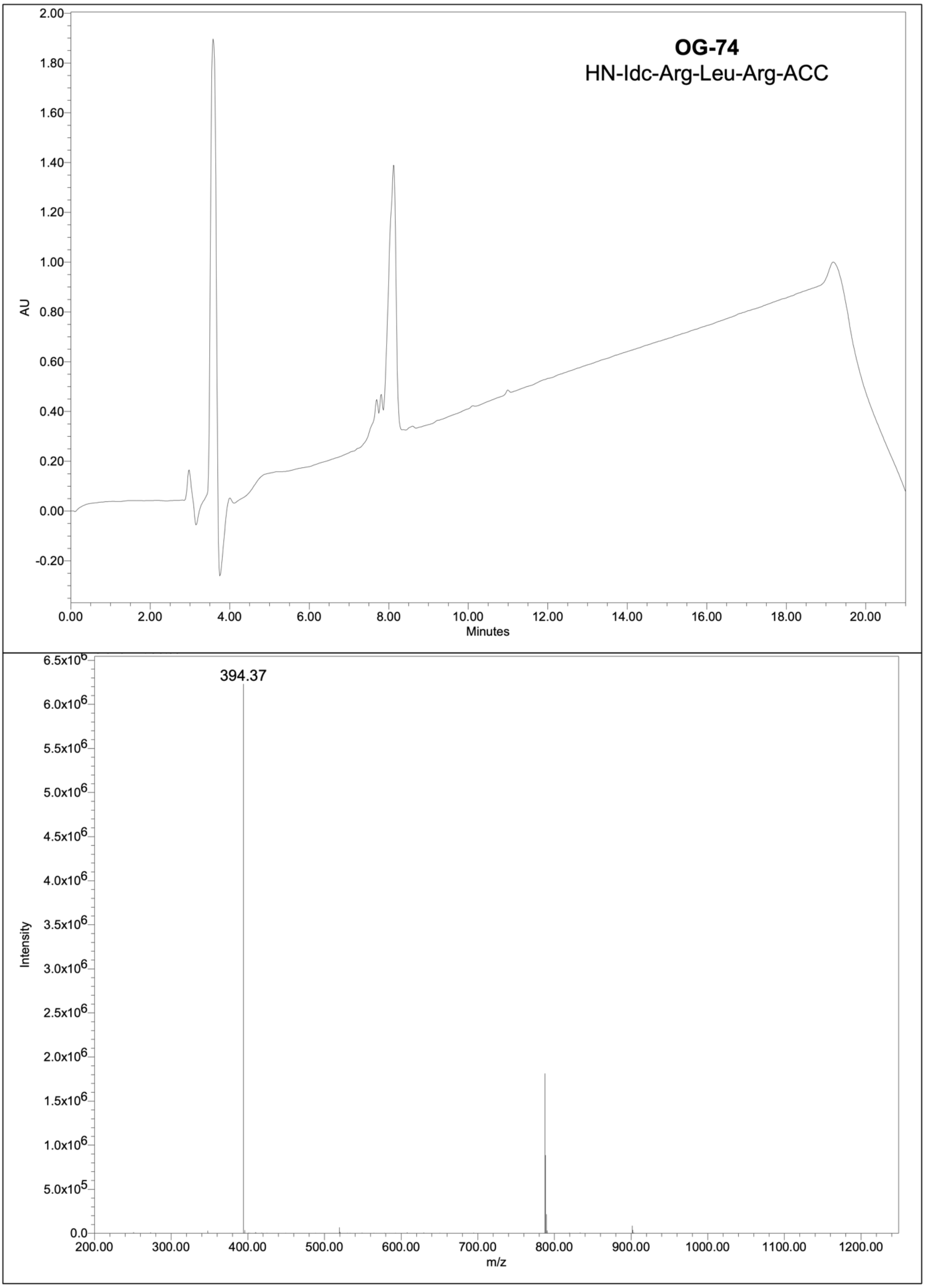

OG-74
HN-Idc-Arg-Leu-Arg-ACC
AU
Minutes
394.37
Intensity
m/z

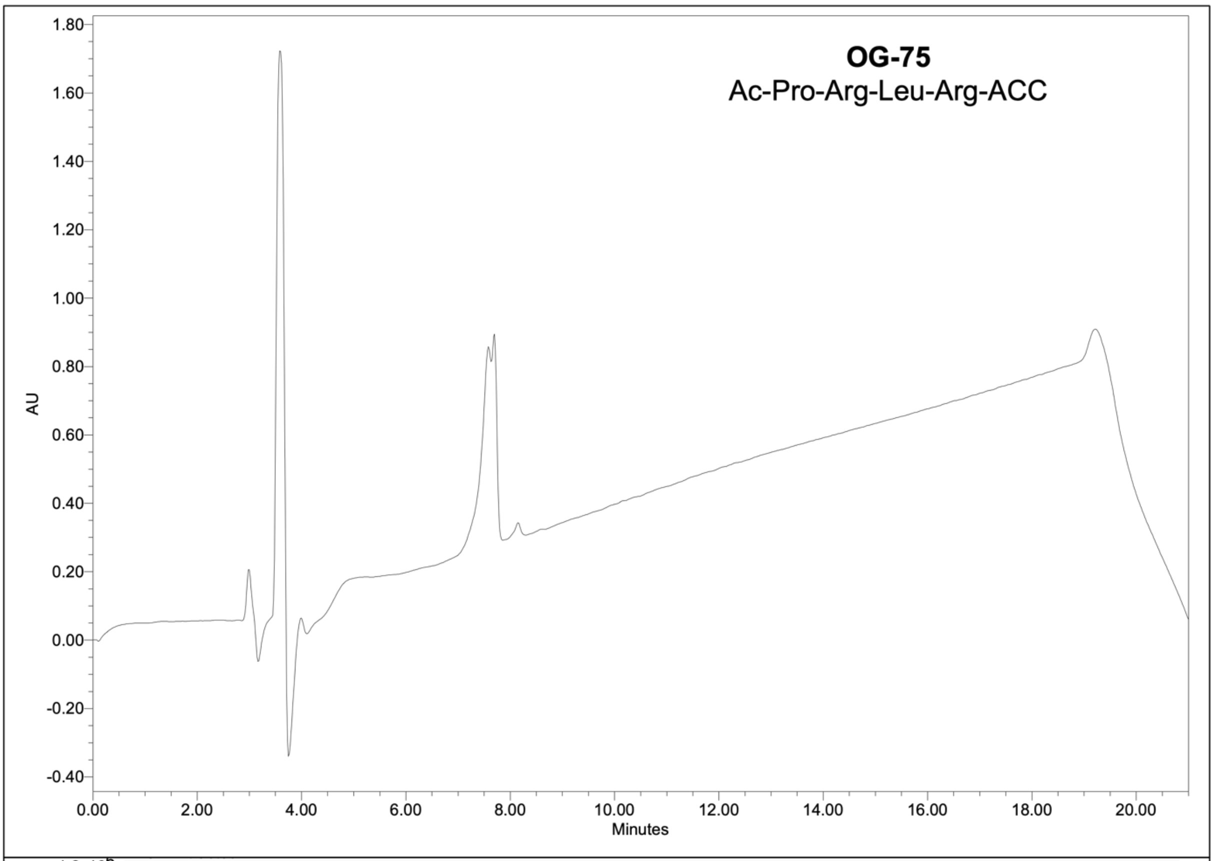
OG-75
Ac-Pro-Arg-Leu-Arg-ACC
AU
Minutes

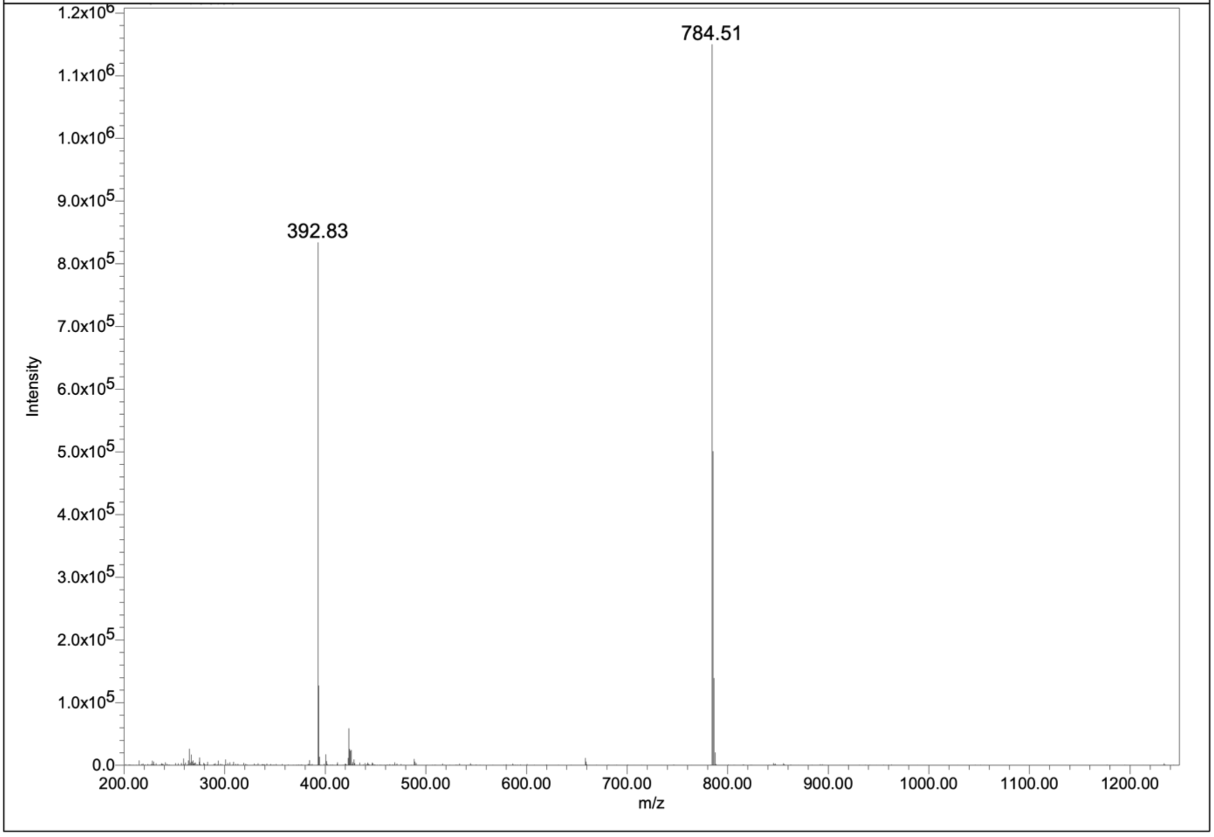
784.51
392.83
Intensity
m/z

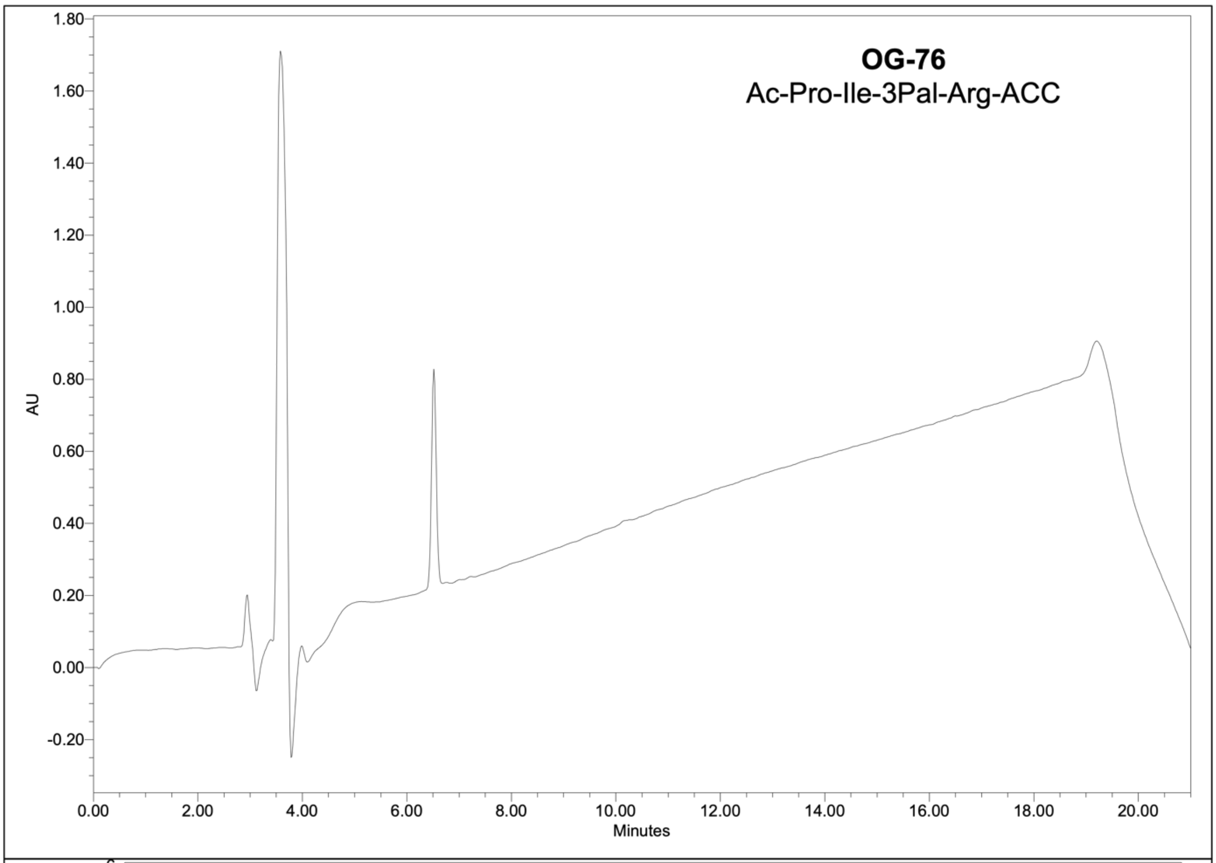
OG-76
Ac-Pro-Ile-3Pal-Arg-ACC
AU
Minutes

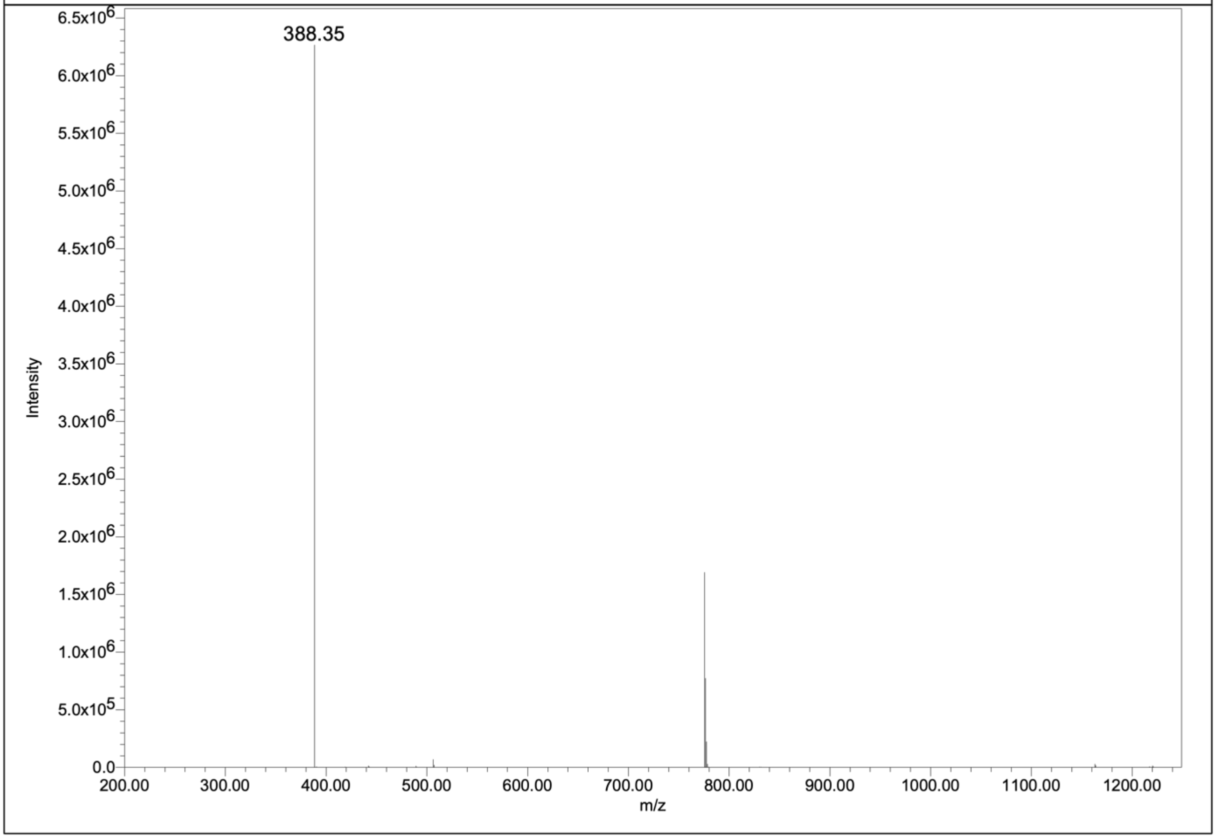
388.35
Intensity
m/z

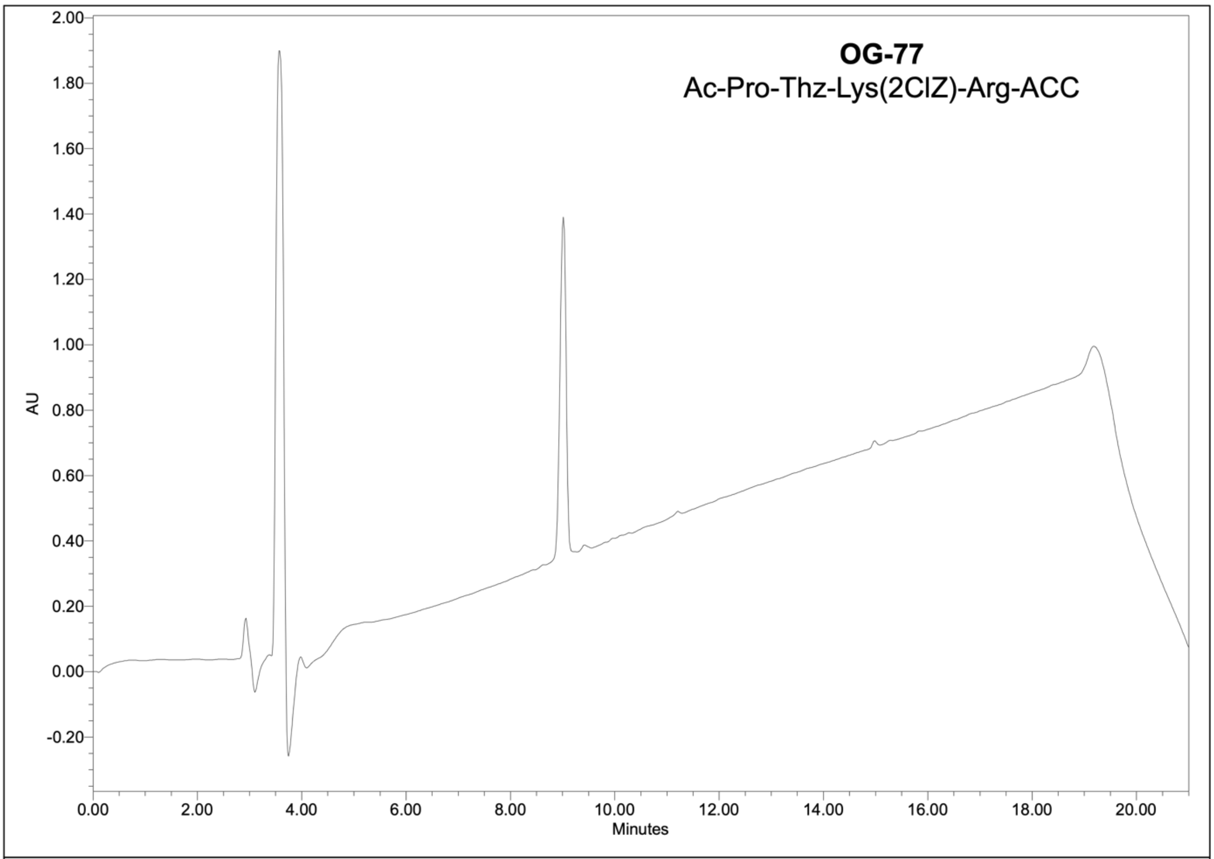
OG-77
Ac-Pro-Thz-Lys(2ClZ)-Arg-ACC
AU
Minutes
2.00
1.80
1.60
1.40
1.20
1.00
0.80
0.60
0.40
0.20
0.00
-0.20
0.00
2.00
4.00
6.00
8.00
10.00
12.00
14.00
16.00
18.00
20.00

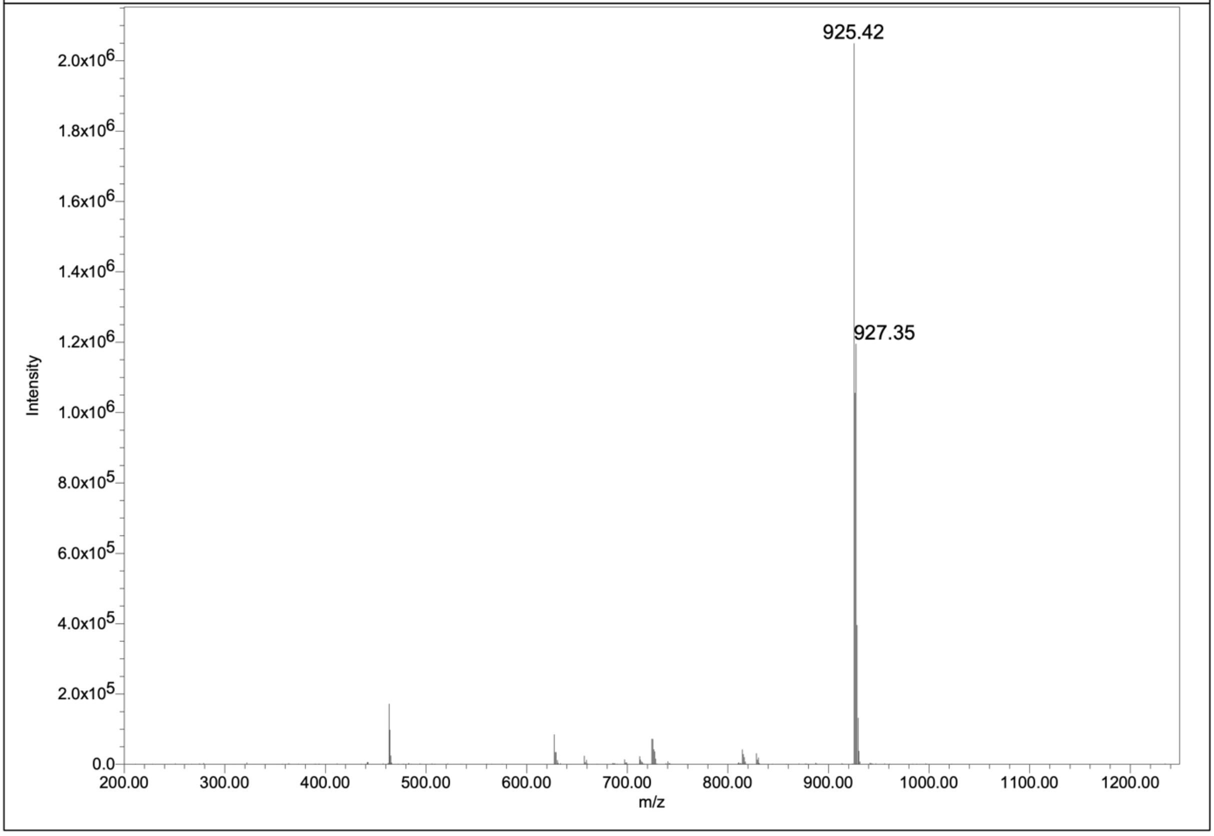
925.42
927.35
Intensity
m/z
2.0x10^6
1.8x10^6
1.6x10^6
1.4x10^6
1.2x10^6
1.0x10^6
8.0x10^5
6.0x10^5
4.0x10^5
2.0x10^5
0.0
200.00
300.00
400.00
500.00
600.00
700.00
800.00
900.00
1000.00
1100.00
1200.00

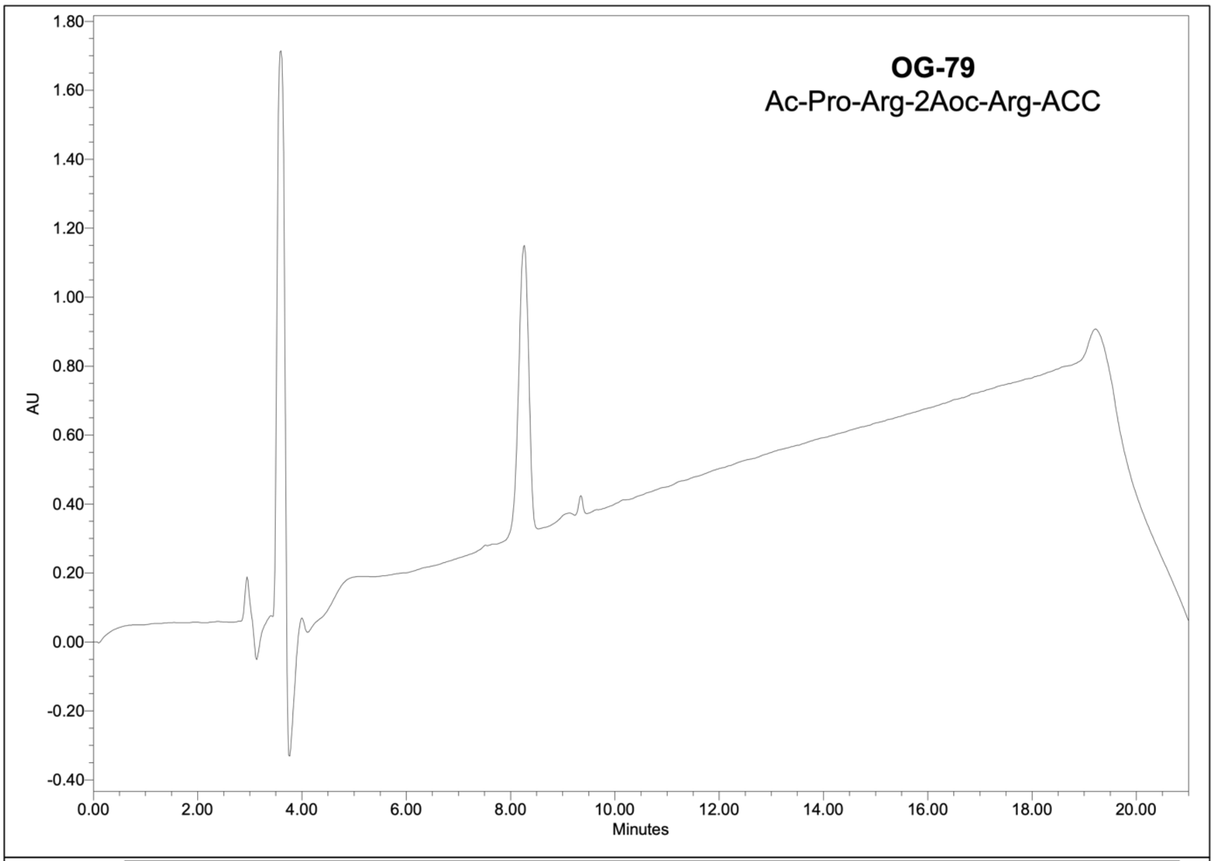
OG-79
Ac-Pro-Arg-2Aoc-Arg-ACC
AU
Minutes
1.80
1.60
1.40
1.20
1.00
0.80
0.60
0.40
0.20
0.00
-0.20
-0.40
0.00
2.00
4.00
6.00
8.00
10.00
12.00
14.00
16.00
18.00
20.00

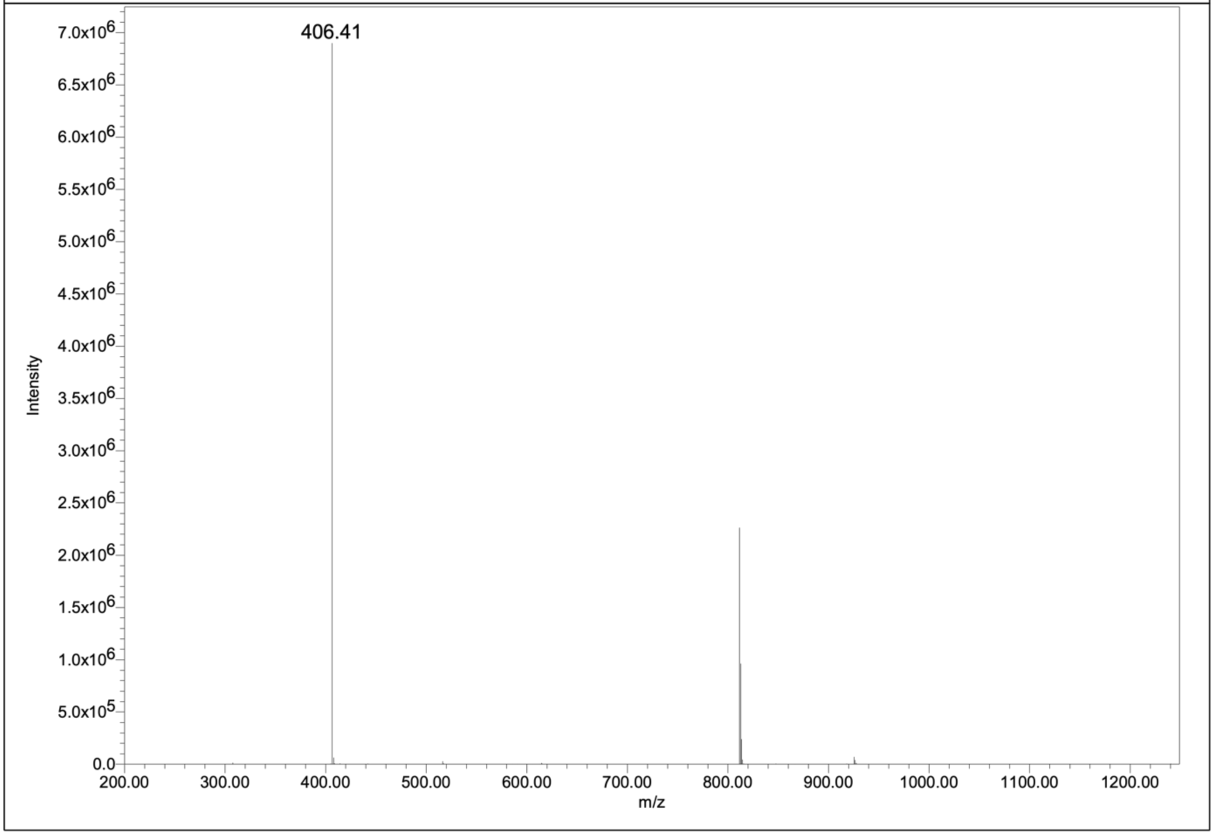
406.41
Intensity
m/z
7.0x10^6
6.5x10^6
6.0x10^6
5.5x10^6
5.0x10^6
4.5x10^6
4.0x10^6
3.5x10^6
3.0x10^6
2.5x10^6
2.0x10^6
1.5x10^6
1.0x10^6
5.0x10^5
0.0
200.00
300.00
400.00
500.00
600.00
700.00
800.00
900.00
1000.00
1100.00
1200.00

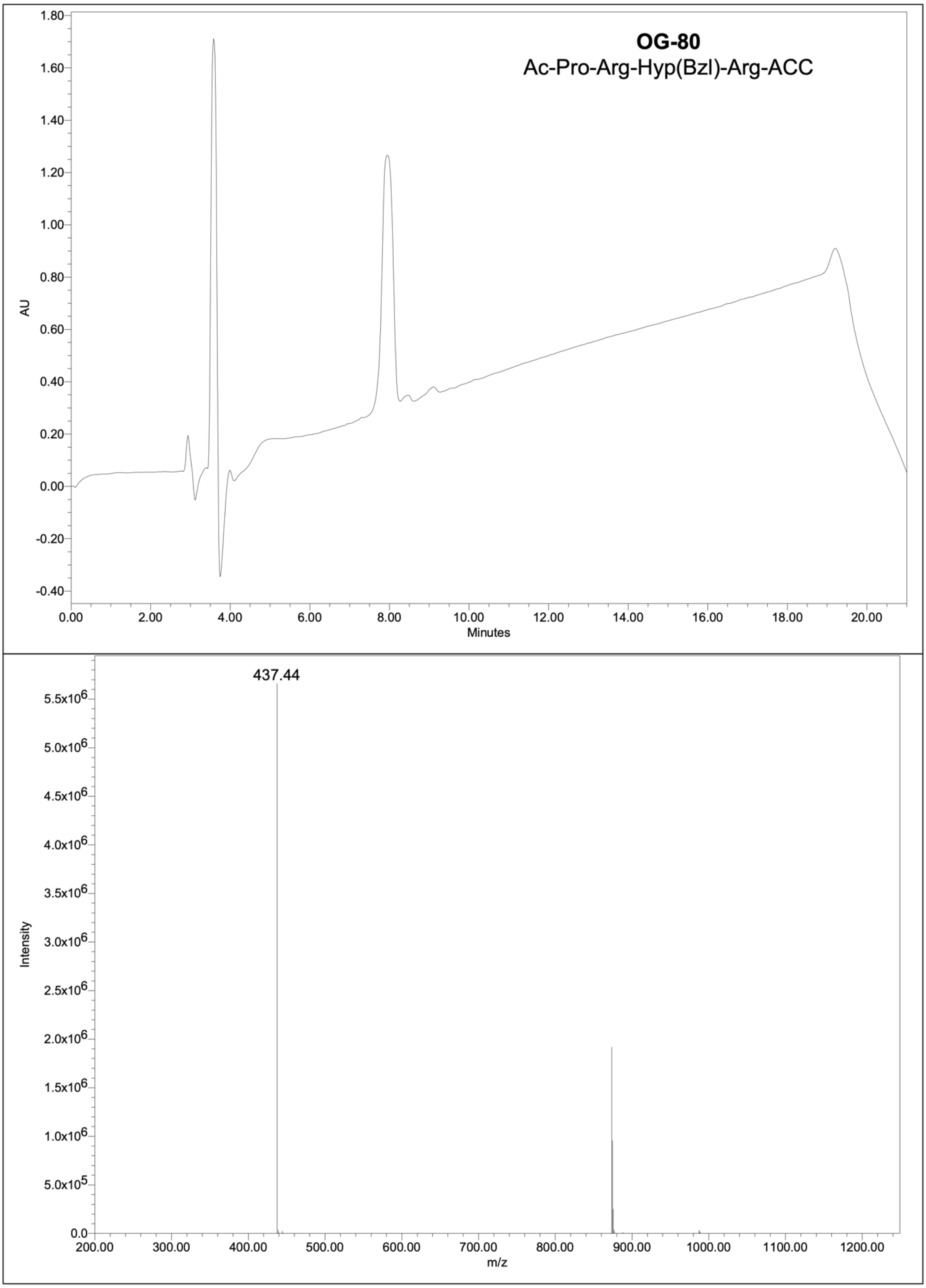
OG-80
Ac-Pro-Arg-Hyp(Bzl)-Arg-ACC
AU
Minutes
437.44
Intensity
m/z

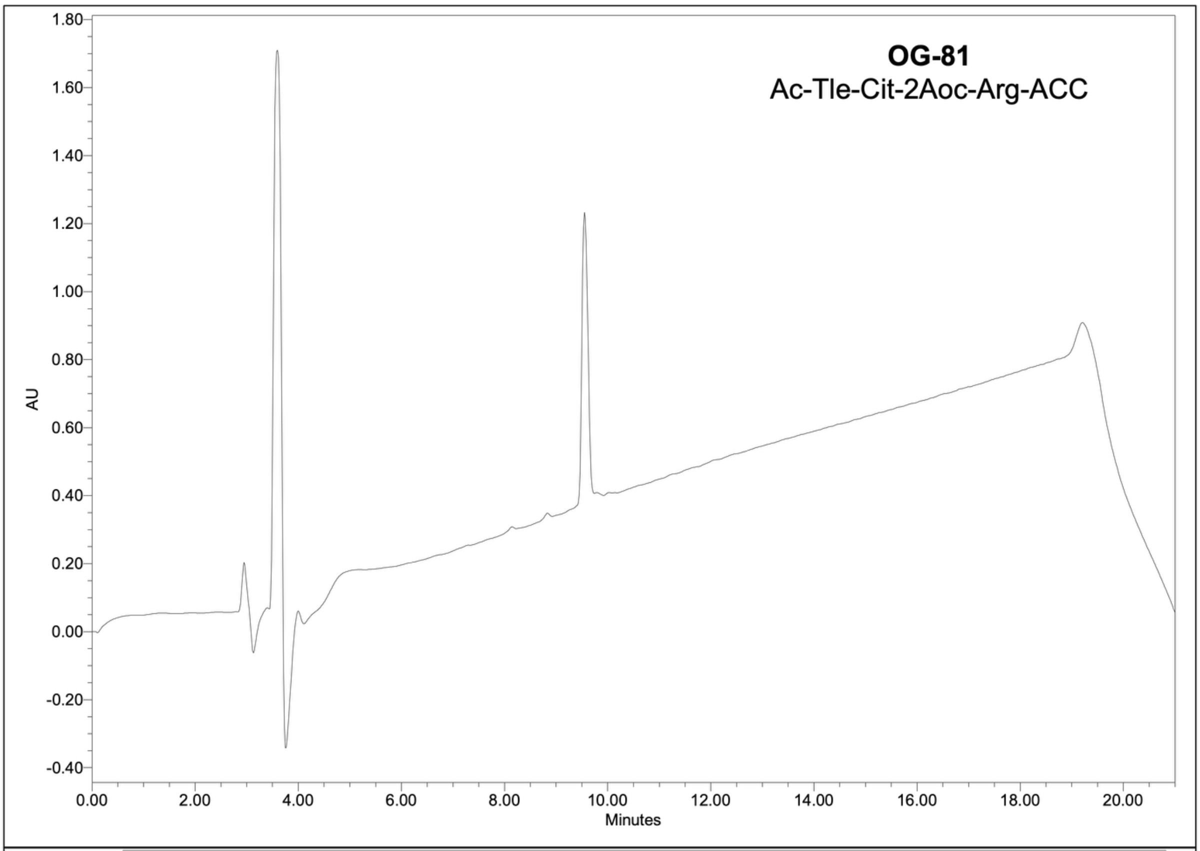
OG-81
Ac-Tle-Cit-2Aoc-Arg-ACC
AU
Minutes
1.80
1.60
1.40
1.20
1.00
0.80
0.60
0.40
0.20
0.00
-0.20
-0.40
0.00
2.00
4.00
6.00
8.00
10.00
12.00
14.00
16.00
18.00
20.00

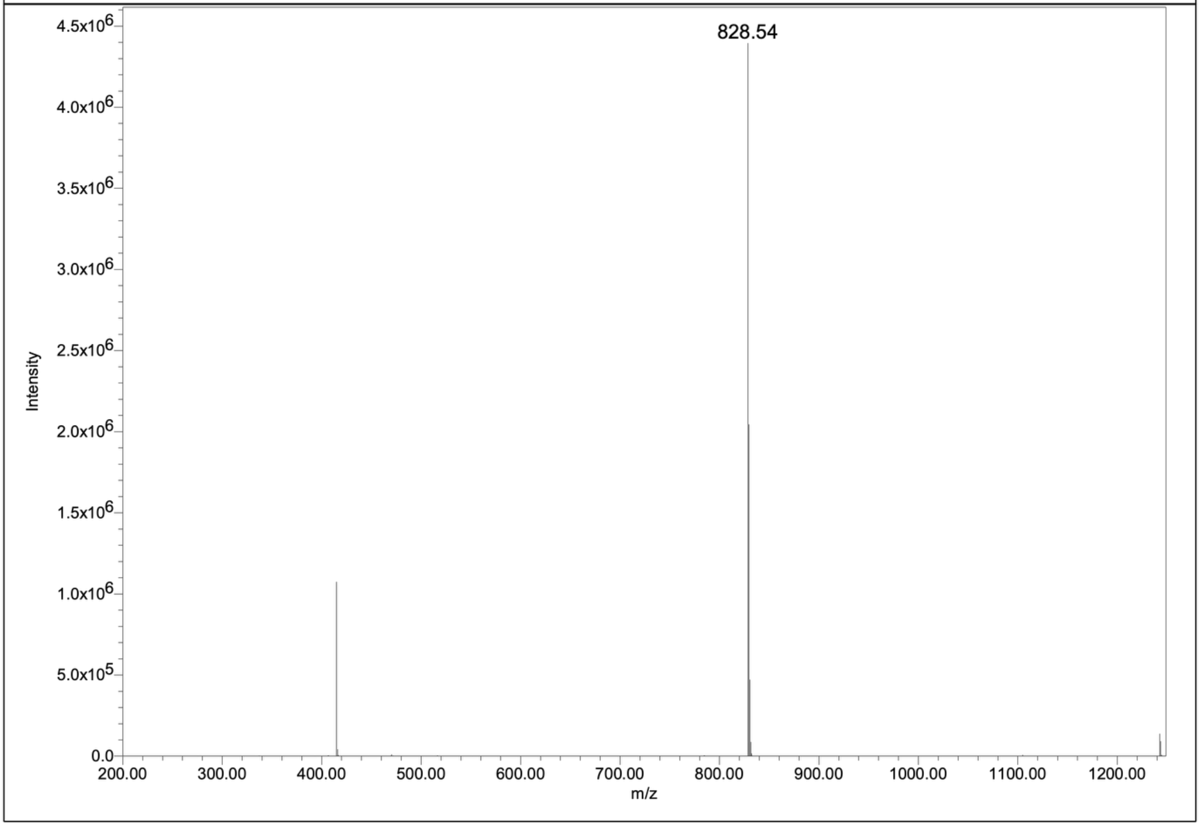
828.54
Intensity
m/z
4.5x10^6
4.0x10^6
3.5x10^6
3.0x10^6
2.5x10^6
2.0x10^6
1.5x10^6
1.0x10^6
5.0x10^5
0.0
200.00
300.00
400.00
500.00
600.00
700.00
800.00
900.00
1000.00
1100.00
1200.00

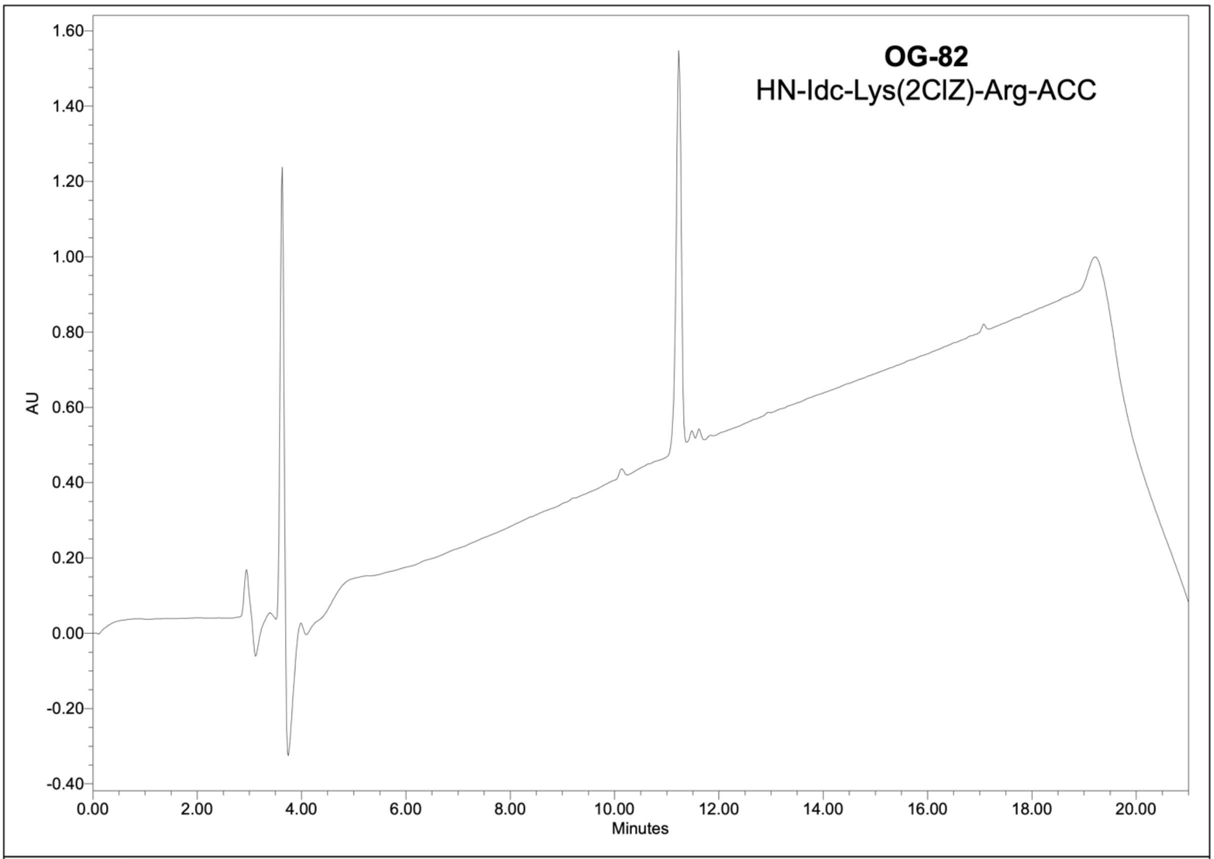

OG-82
HN-Idc-Lys(2ClZ)-Arg-ACC
AU
Minutes

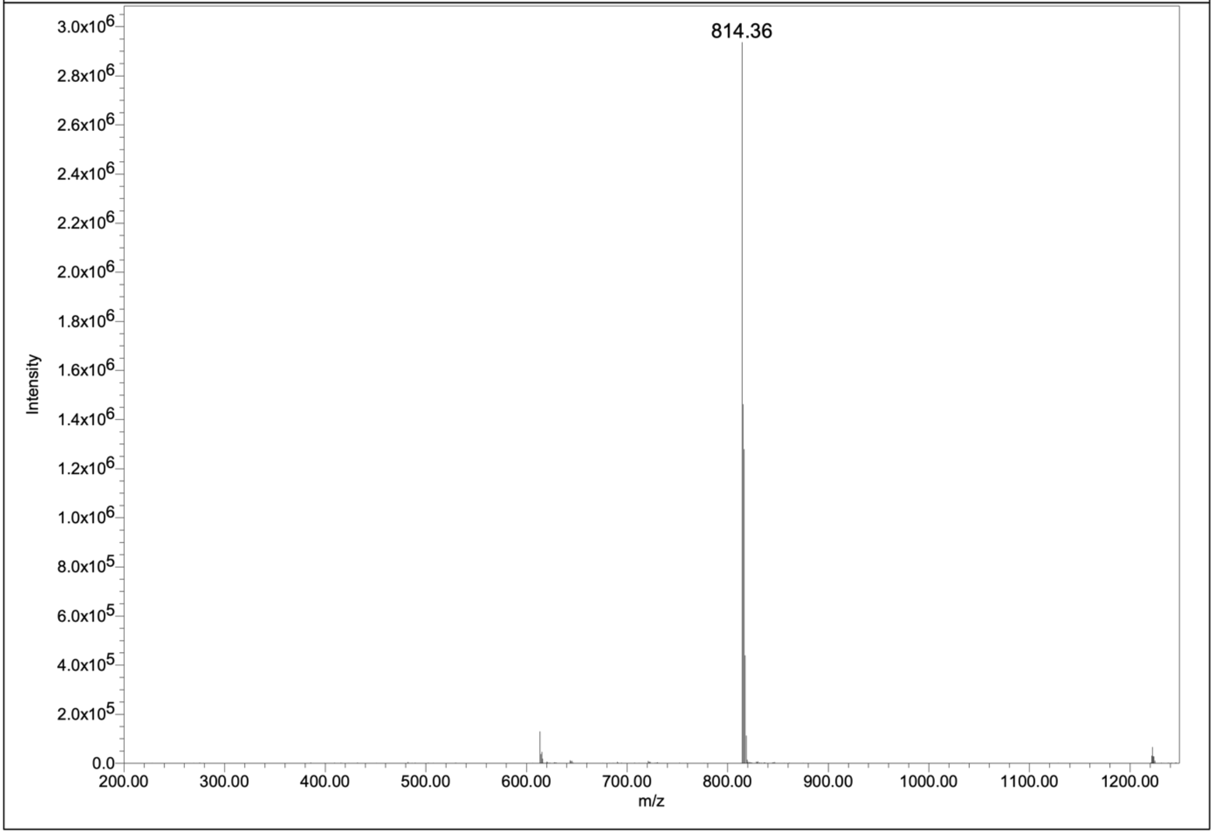

814.36
Intensity
m/z

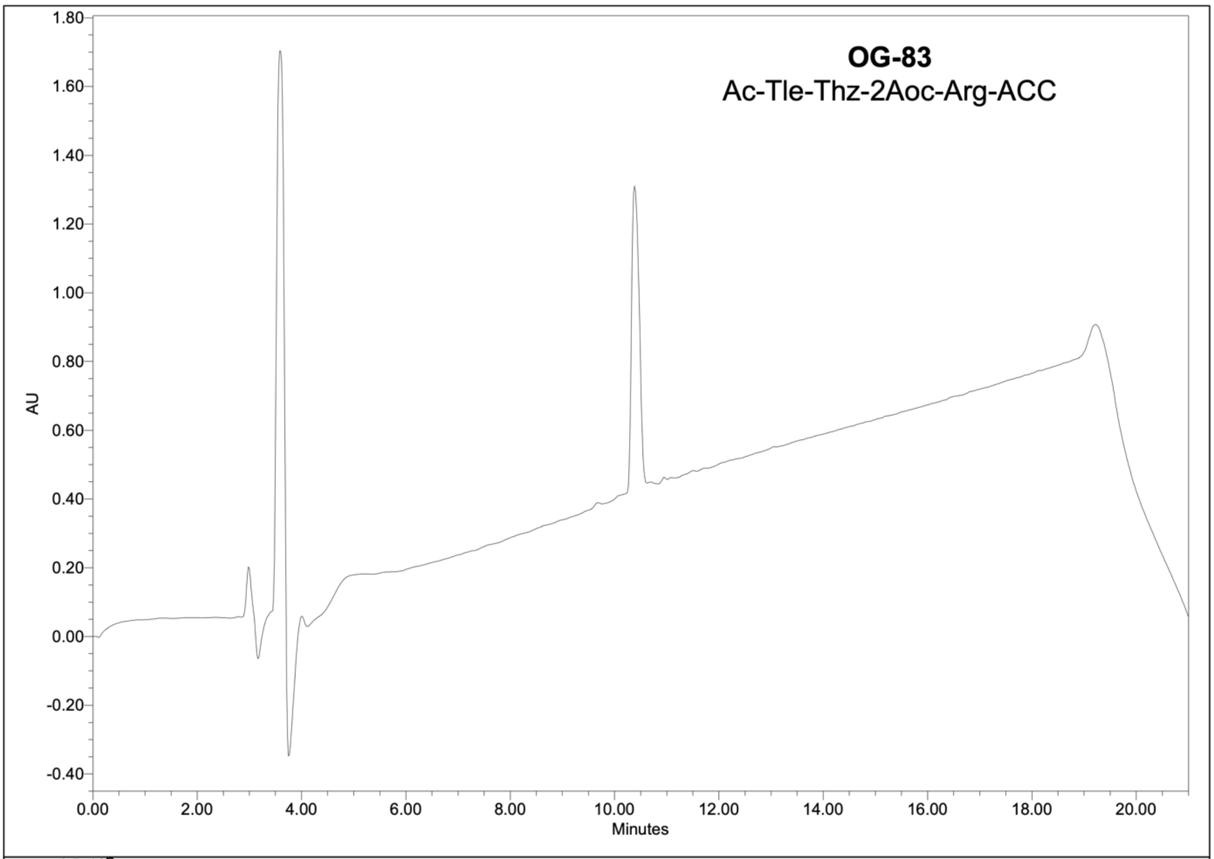
OG-83
Ac-Tle-Thz-2Aoc-Arg-ACC
AU
Minutes

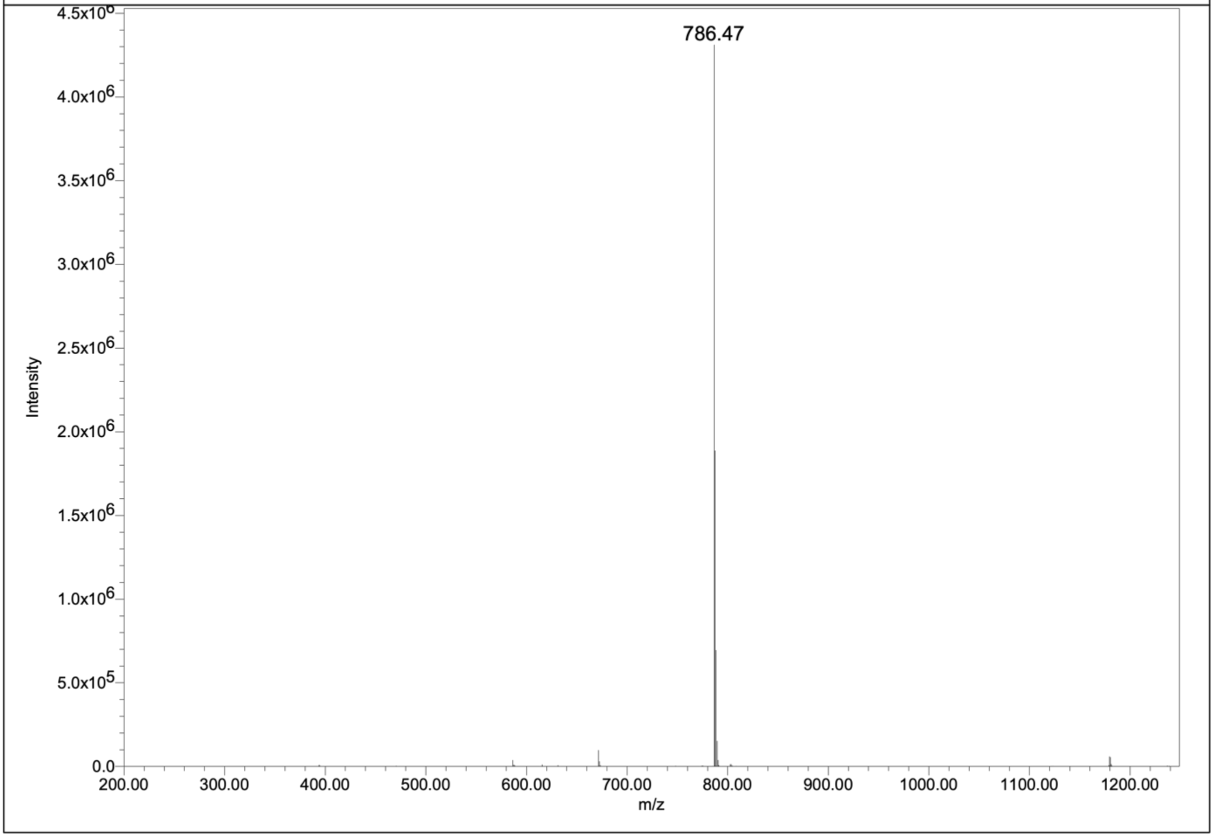
786.47
Intensity
m/z

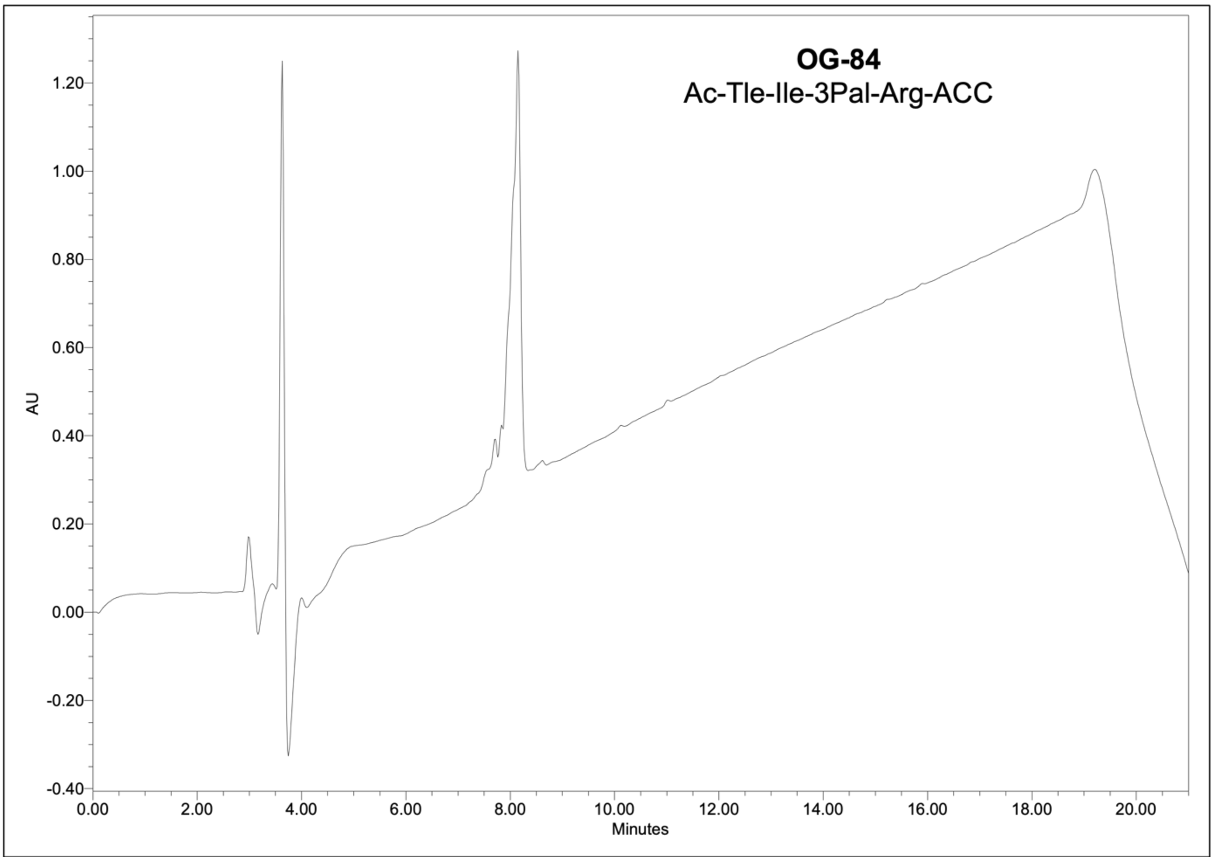
OG-84
Ac-Tle-Ile-3Pal-Arg-ACC
AU
1.20
1.00
0.80
0.60
0.40
0.20
0.00
-0.20
-0.40
0.00
2.00
4.00
6.00
8.00
10.00
12.00
14.00
16.00
18.00
20.00
Minutes

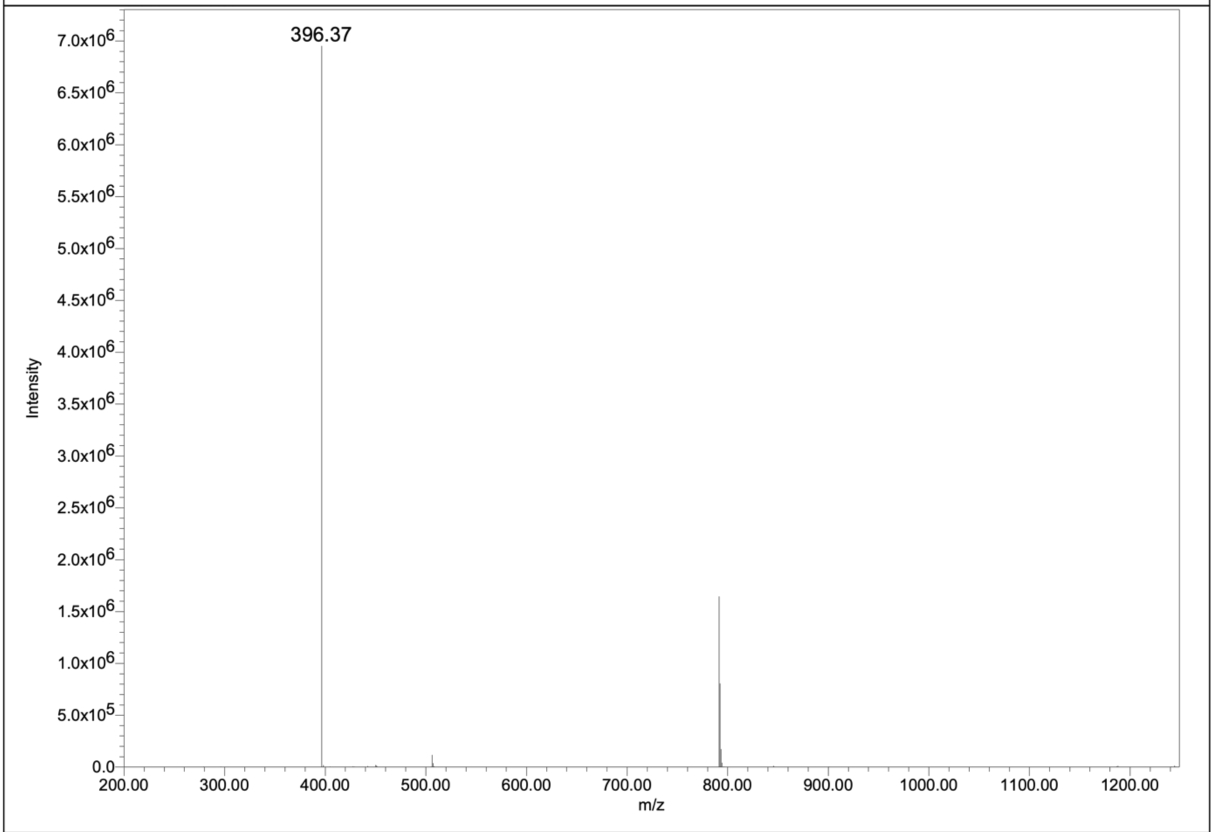
396.37
Intensity
7.0x10^6
6.5x10^6
6.0x10^6
5.5x10^6
5.0x10^6
4.5x10^6
4.0x10^6
3.5x10^6
3.0x10^6
2.5x10^6
2.0x10^6
1.5x10^6
1.0x10^6
5.0x10^5
0.0
200.00
300.00
400.00
500.00
600.00
700.00
800.00
900.00
1000.00
1100.00
1200.00
m/z

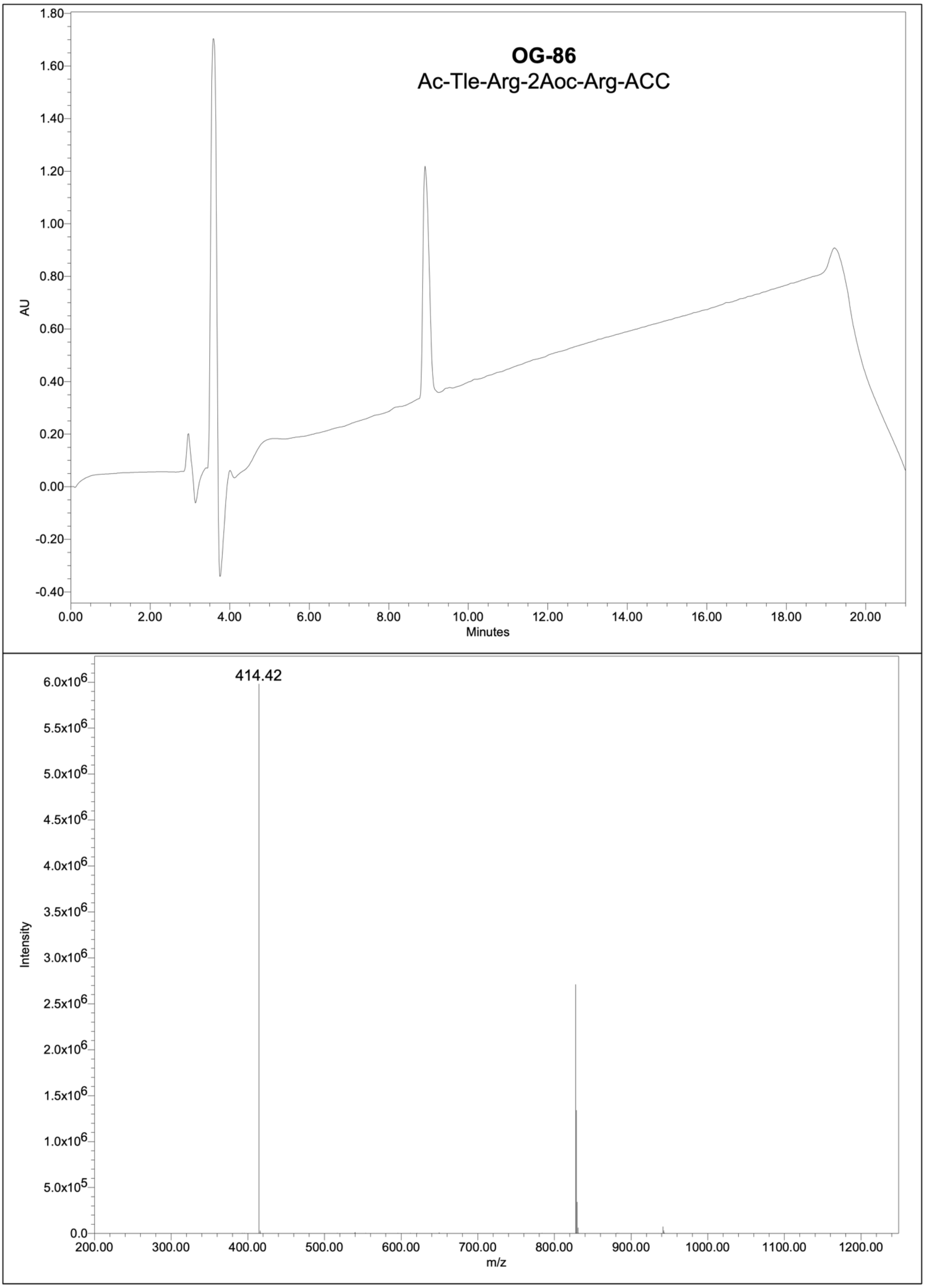
OG-86
Ac-Tle-Arg-2Aoc-Arg-ACC
AU
Minutes
414.42
Intensity
m/z

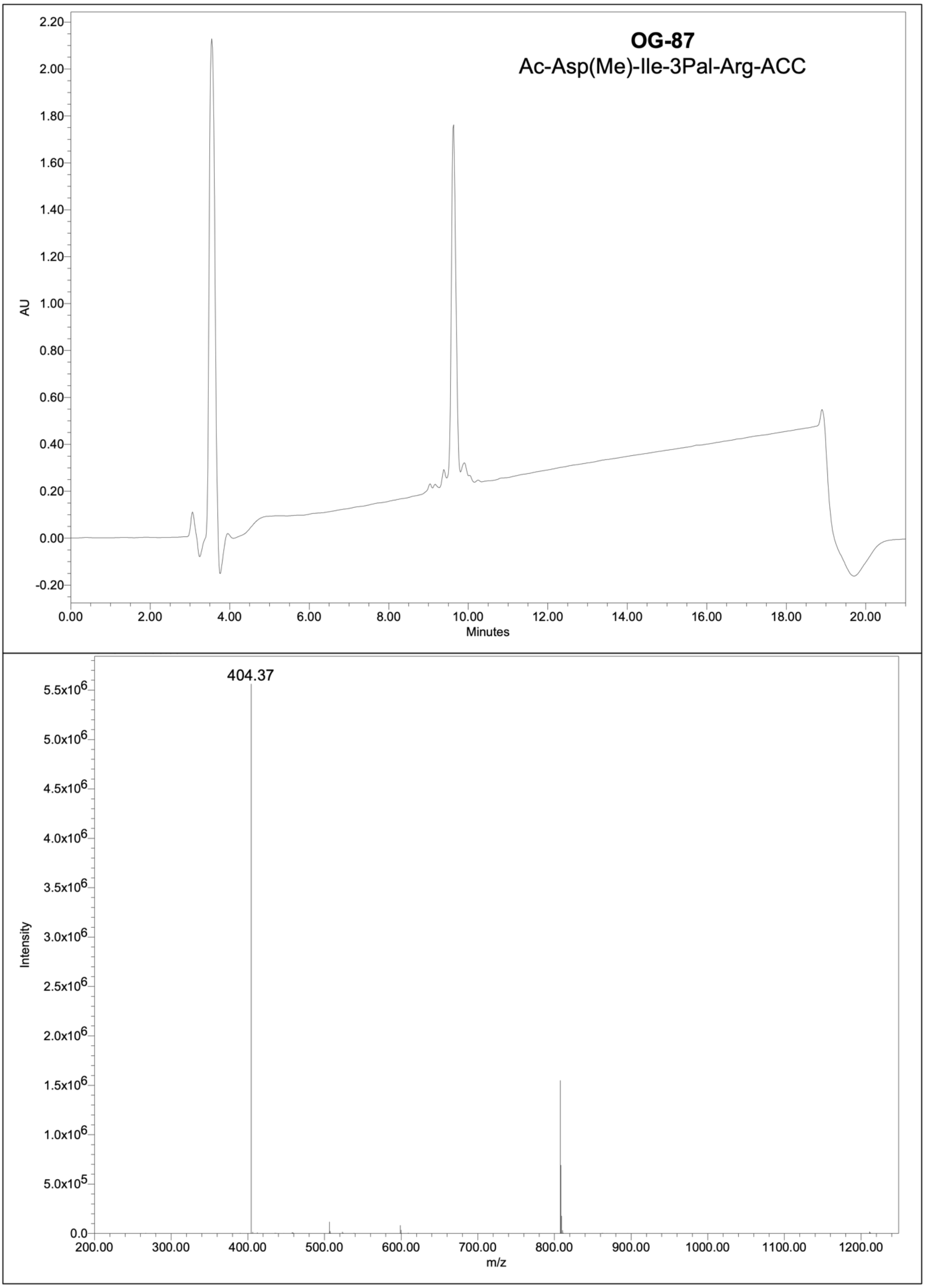
OG-87
Ac-Asp(Me)-Ile-3Pal-Arg-ACC
AU
Minutes
404.37
Intensity
m/z

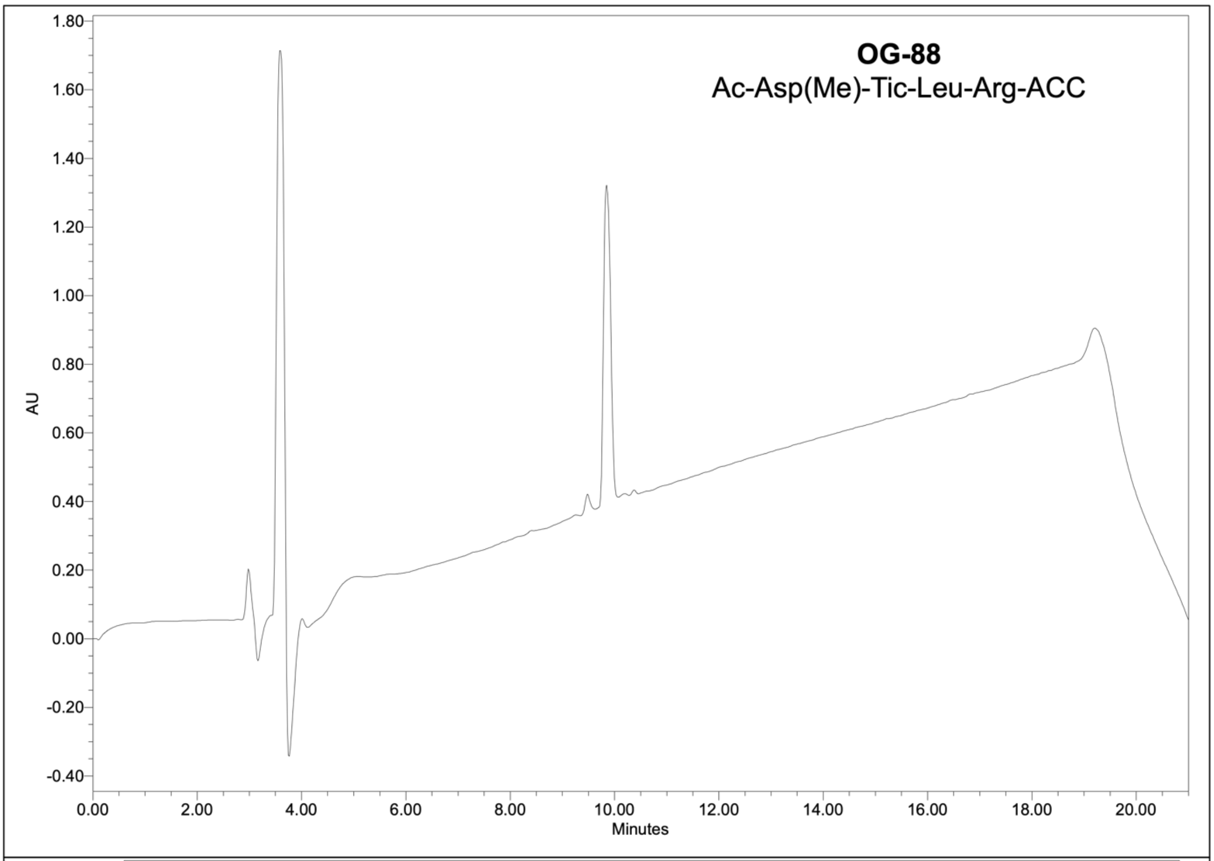
OG-88
Ac-Asp(Me)-Tic-Leu-Arg-ACC
AU
Minutes

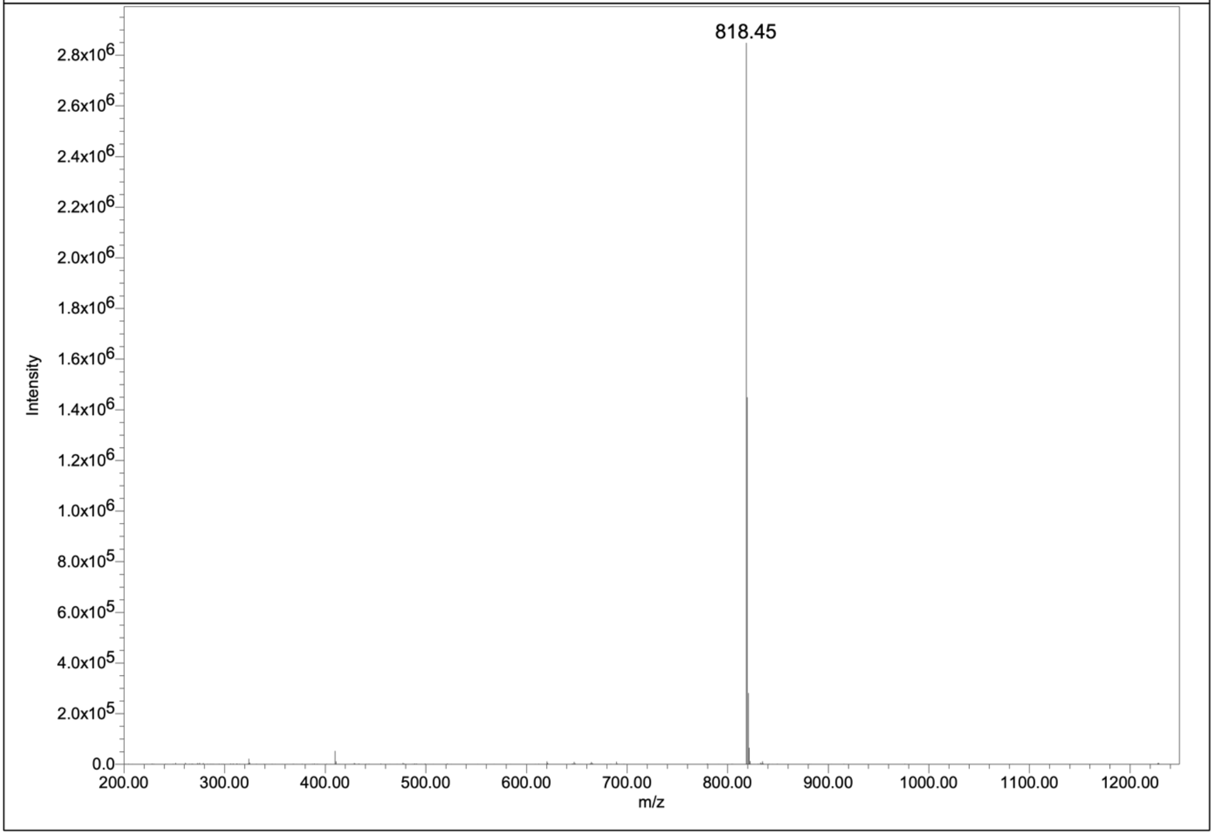
818.45
Intensity
m/z

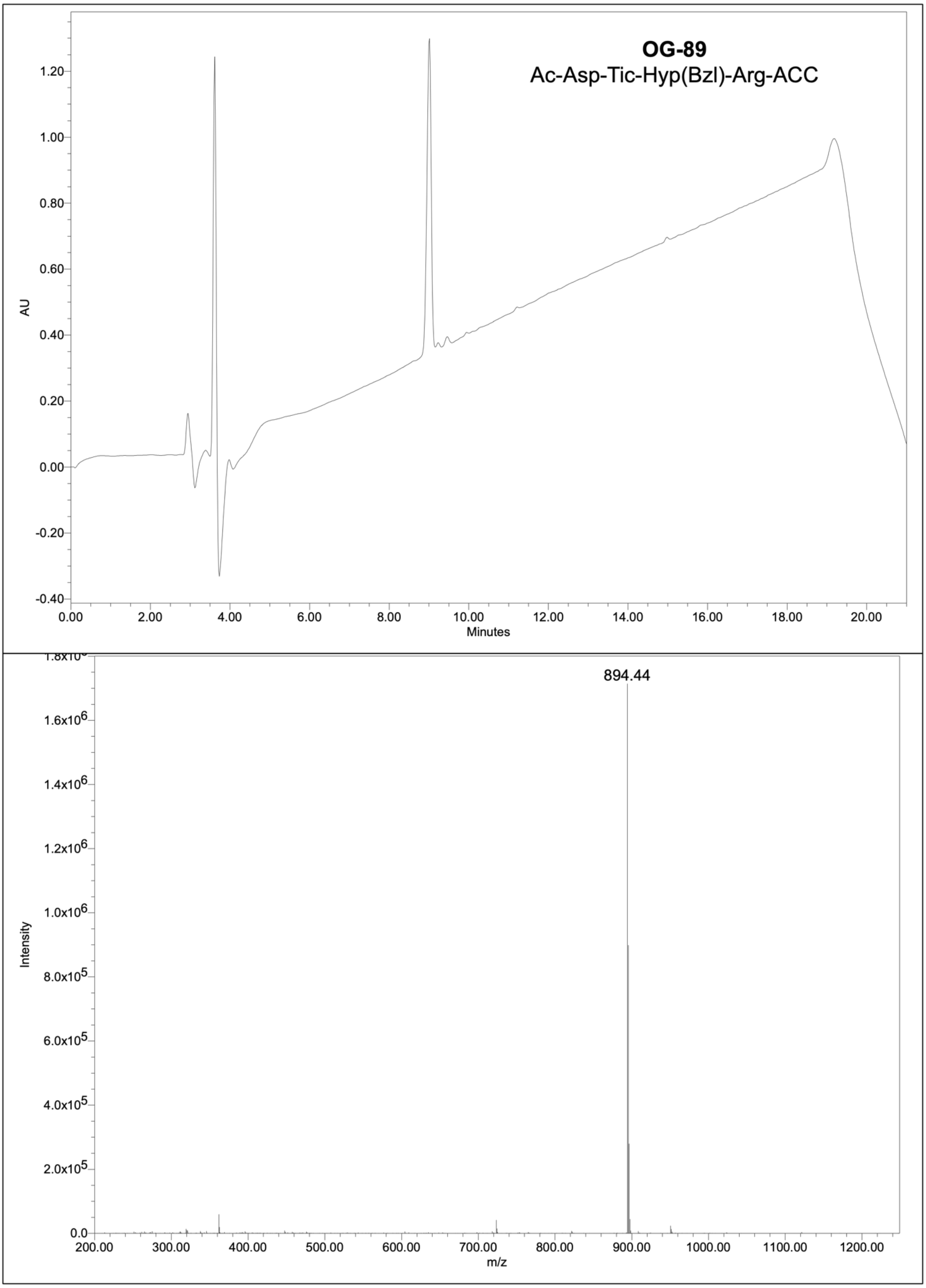
OG-89
Ac-Asp-Tic-Hyp(Bzl)-Arg-ACC
AU
Minutes
894.44
Intensity
m/z

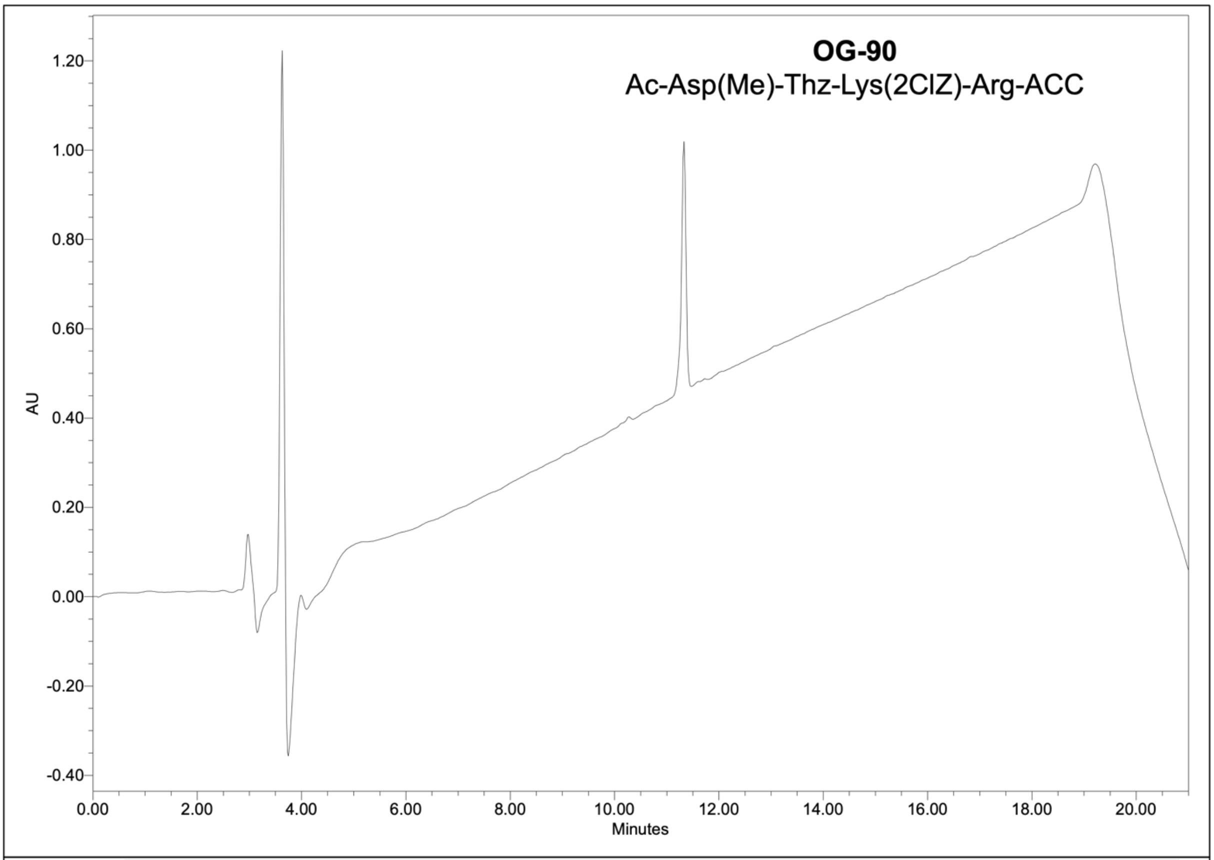
OG-90
Ac-Asp(Me)-Thz-Lys(2ClZ)-Arg-ACC
AU
Minutes
1.20
1.00
0.80
0.60
0.40
0.20
0.00
-0.20
-0.40
0.00
2.00
4.00
6.00
8.00
10.00
12.00
14.00
16.00
18.00
20.00

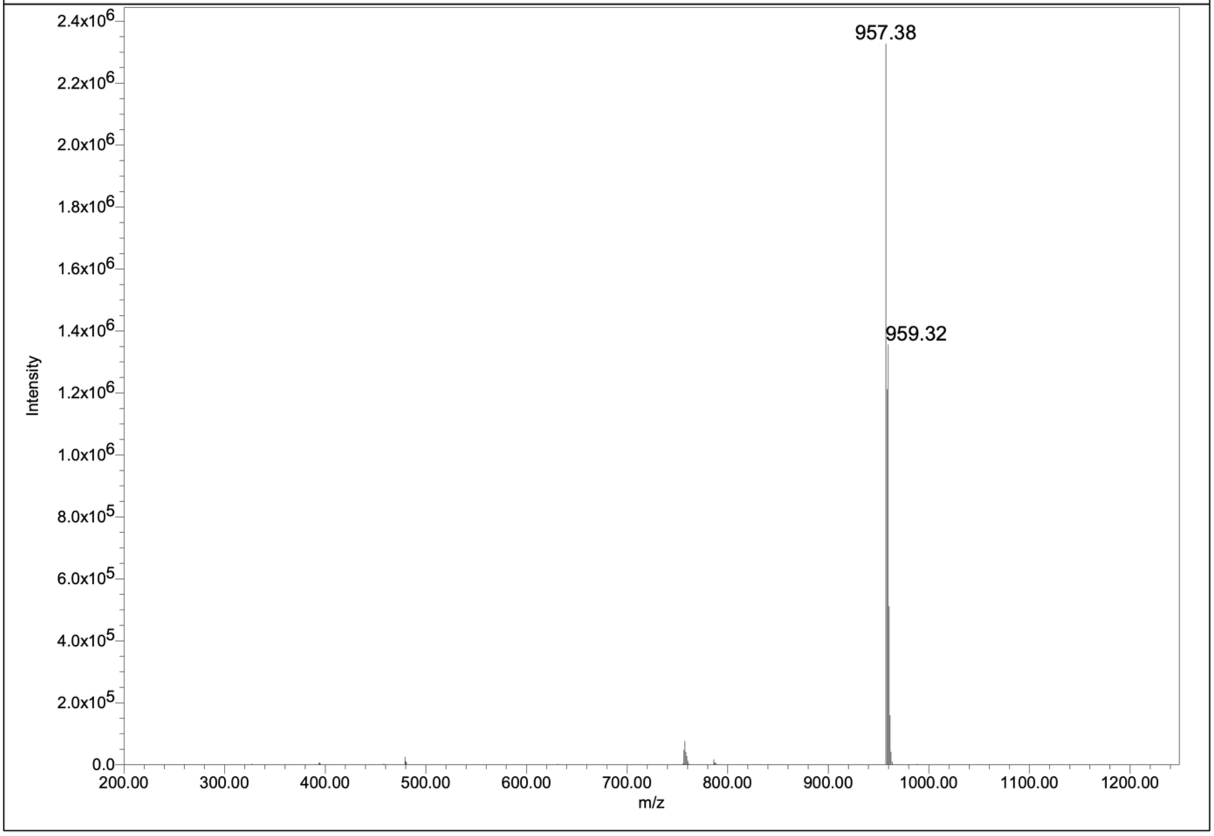
957.38
959.32
Intensity
m/z
2.4x10^6
2.2x10^6
2.0x10^6
1.8x10^6
1.6x10^6
1.4x10^6
1.2x10^6
1.0x10^6
8.0x10^5
6.0x10^5
4.0x10^5
2.0x10^5
0.0
200.00
300.00
400.00
500.00
600.00
700.00
800.00
900.00
1000.00
1100.00
1200.00

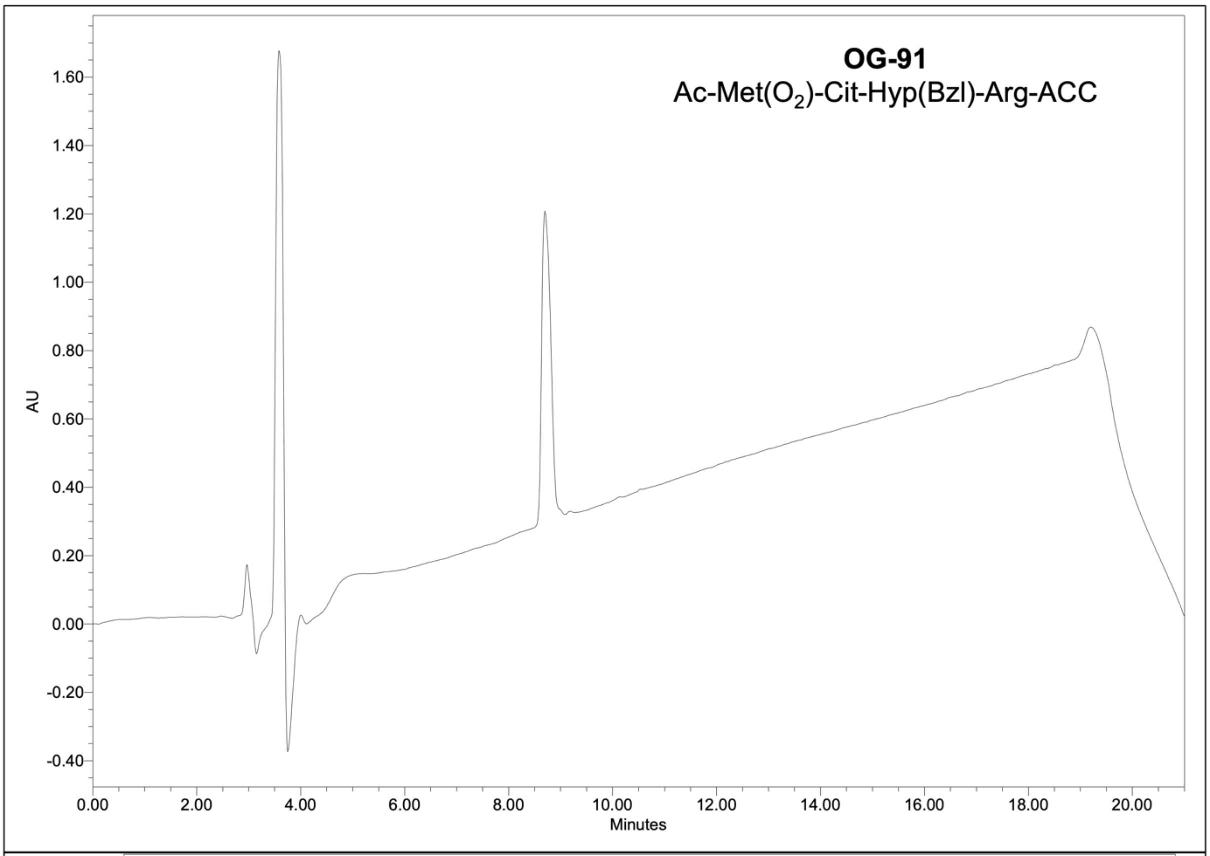

OG-91
Ac-Met(O_2)-Cit-Hyp(Bzl)-Arg-ACC
AU
1.60
1.40
1.20
1.00
0.80
0.60
0.40
0.20
0.00
-0.20
-0.40
0.00
2.00
4.00
6.00
8.00
10.00
12.00
14.00
16.00
18.00
20.00
Minutes

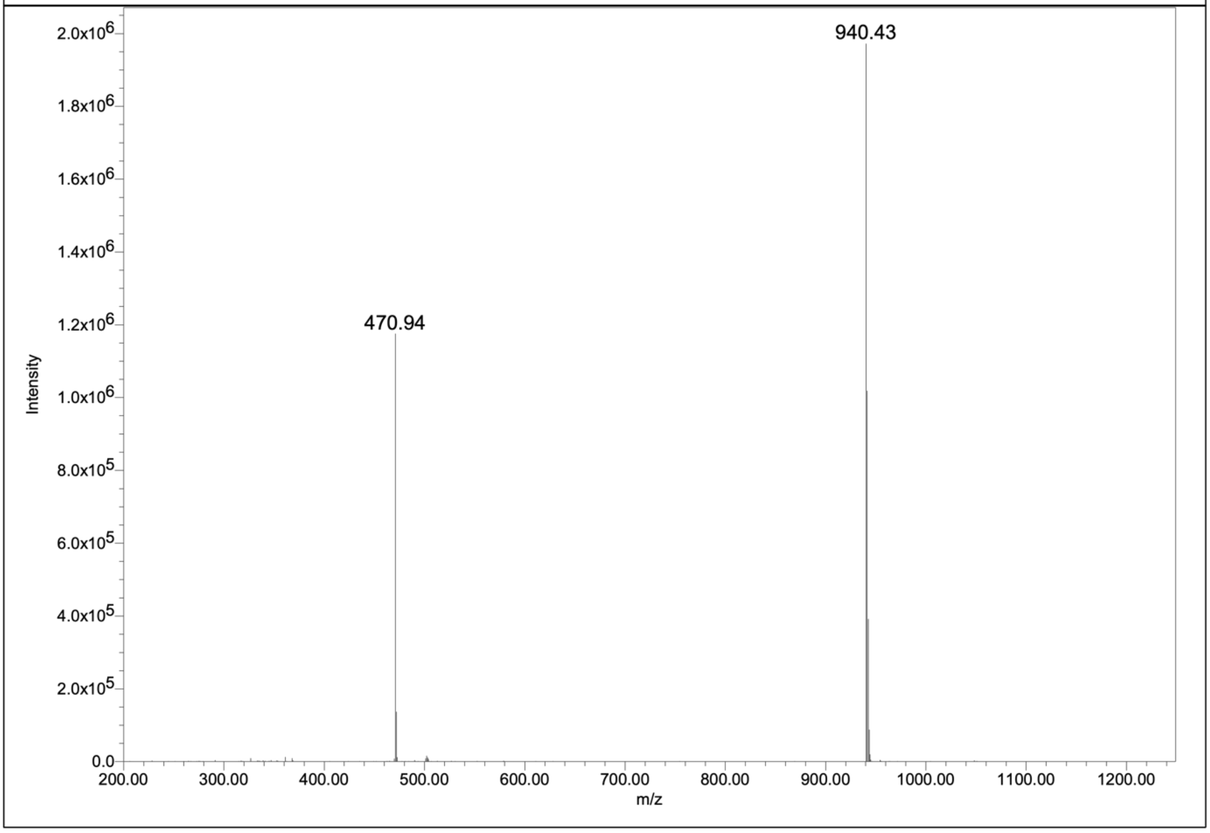

940.43
470.94
Intensity
2.0x10^6
1.8x10^6
1.6x10^6
1.4x10^6
1.2x10^6
1.0x10^6
8.0x10^5
6.0x10^5
4.0x10^5
2.0x10^5
0.0
200.00
300.00
400.00
500.00
600.00
700.00
800.00
900.00
1000.00
1100.00
1200.00
m/z

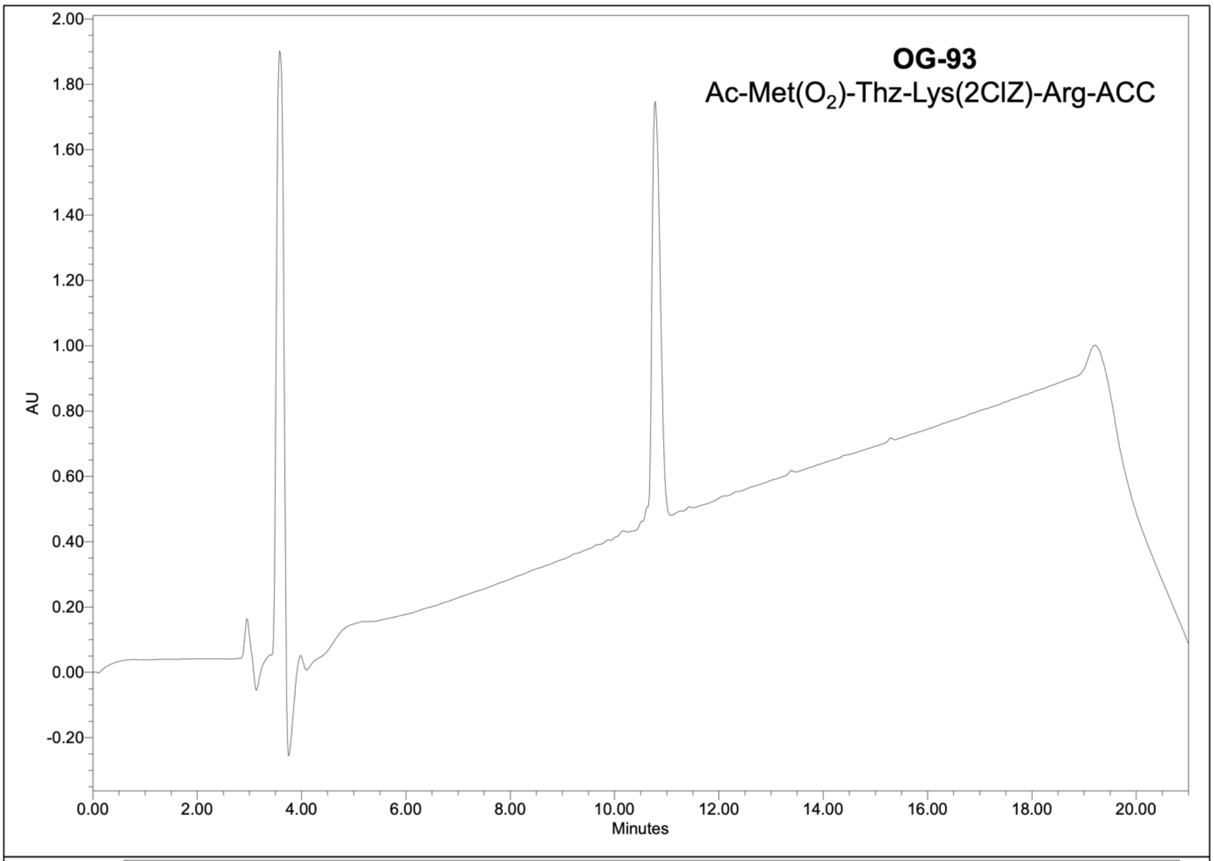
OG-93
Ac-Met(O_2)-Thz-Lys(2ClZ)-Arg-ACC
AU
Minutes
2.00
1.80
1.60
1.40
1.20
1.00
0.80
0.60
0.40
0.20
0.00
-0.20
0.00
2.00
4.00
6.00
8.00
10.00
12.00
14.00
16.00
18.00
20.00

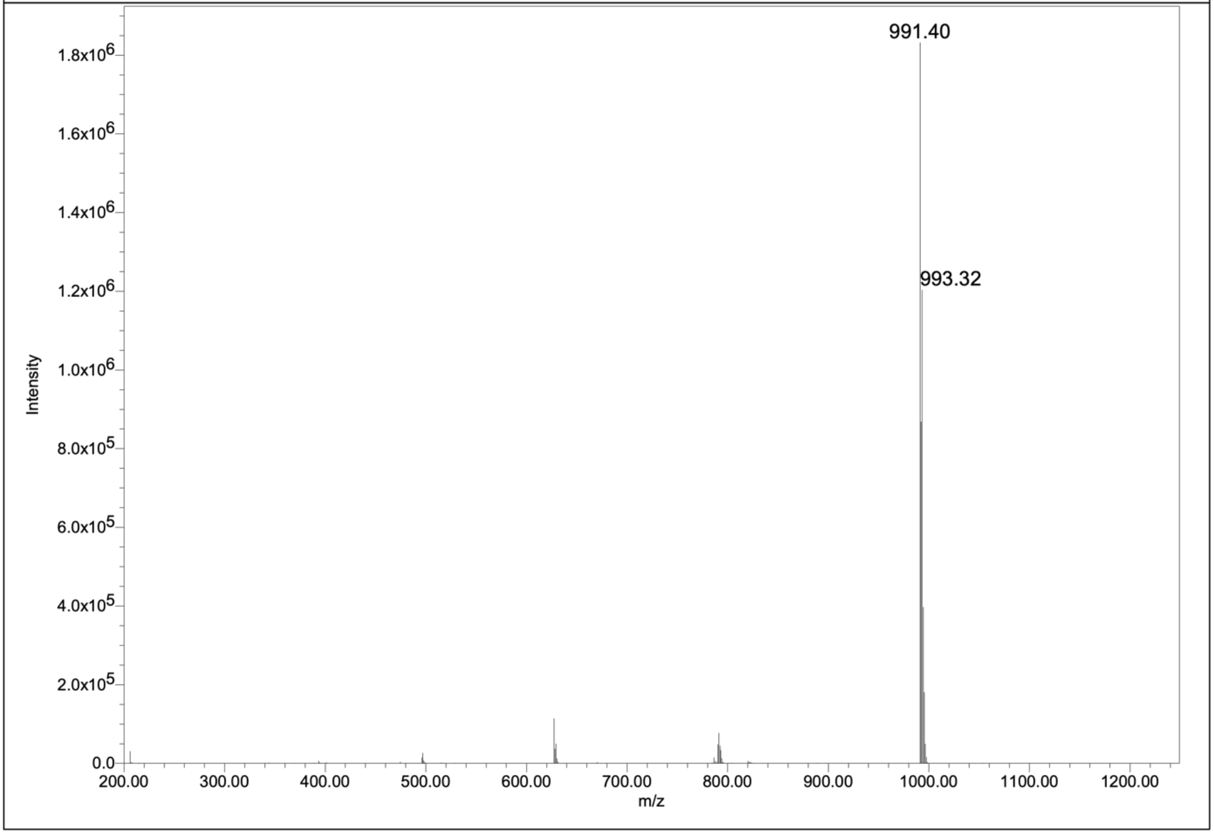
991.40
993.32
Intensity
m/z
1.8x10^6
1.6x10^6
1.4x10^6
1.2x10^6
1.0x10^6
8.0x10^5
6.0x10^5
4.0x10^5
2.0x10^5
0.0
200.00
300.00
400.00
500.00
600.00
700.00
800.00
900.00
1000.00
1100.00
1200.00

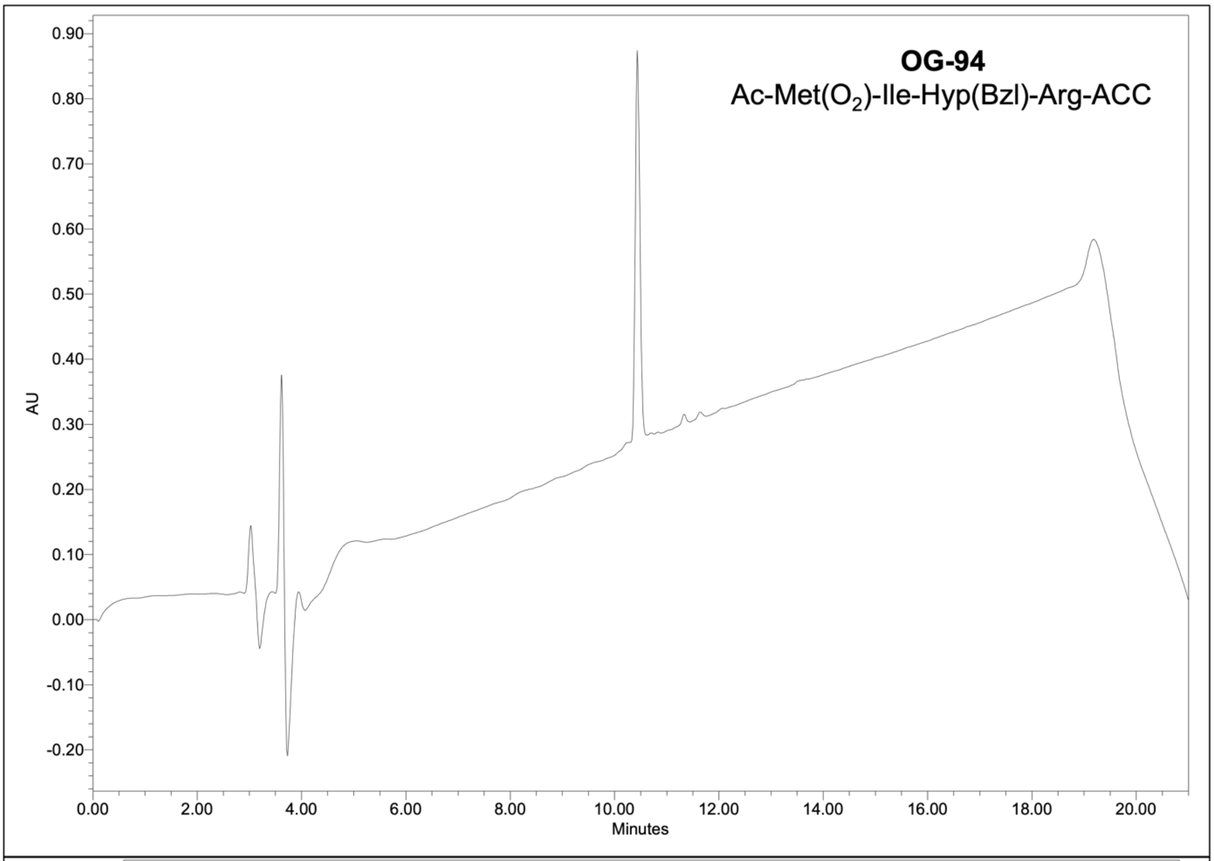

OG-94
Ac-Met(O_2)-Ile-Hyp(Bzl)-Arg-ACC
AU
Minutes

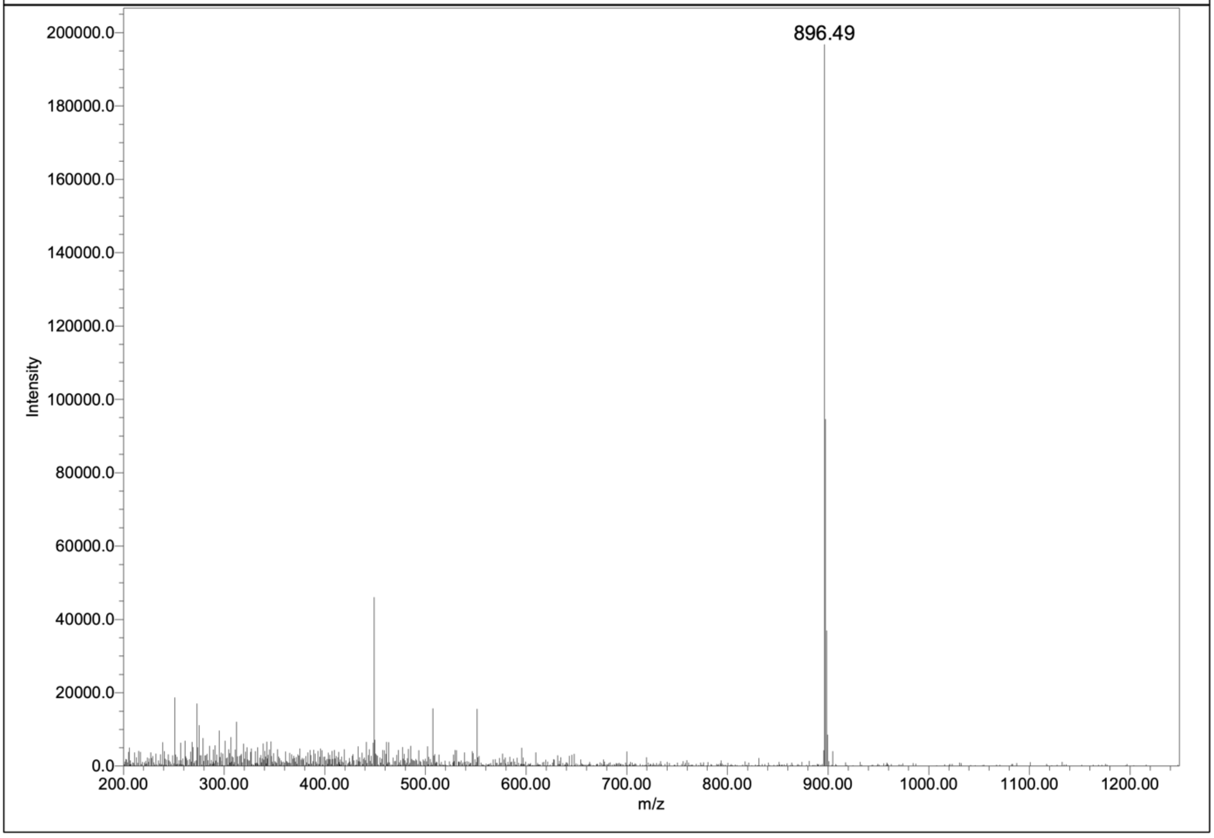

896.49
Intensity
m/z

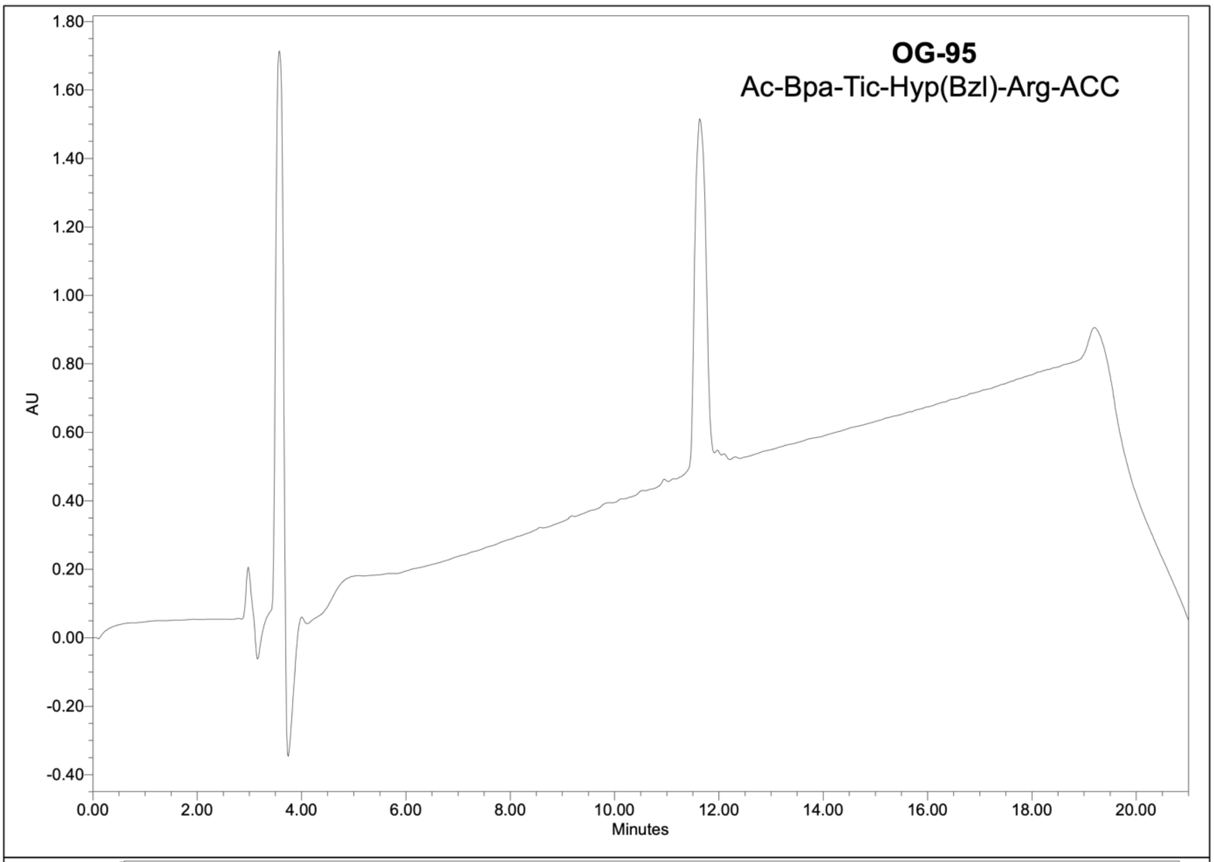

OG-95
Ac-Bpa-Tic-Hyp(Bzl)-Arg-ACC
AU
Minutes
1.80
1.60
1.40
1.20
1.00
0.80
0.60
0.40
0.20
0.00
-0.20
-0.40
0.00
2.00
4.00
6.00
8.00
10.00
12.00
14.00
16.00
18.00
20.00

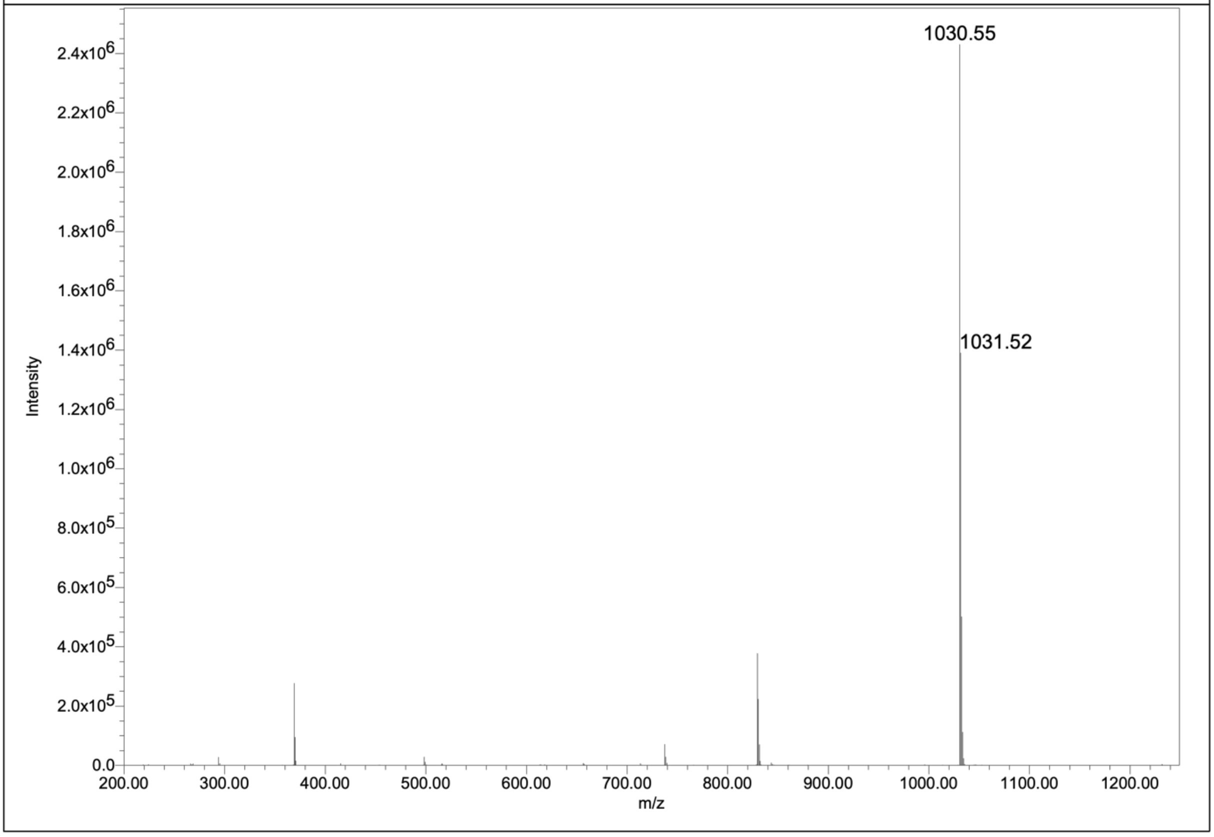

1030.55
1031.52
Intensity
m/z
2.4x10^6
2.2x10^6
2.0x10^6
1.8x10^6
1.6x10^6
1.4x10^6
1.2x10^6
1.0x10^6
8.0x10^5
6.0x10^5
4.0x10^5
2.0x10^5
0.0
200.00
300.00
400.00
500.00
600.00
700.00
800.00
900.00
1000.00
1100.00
1200.00

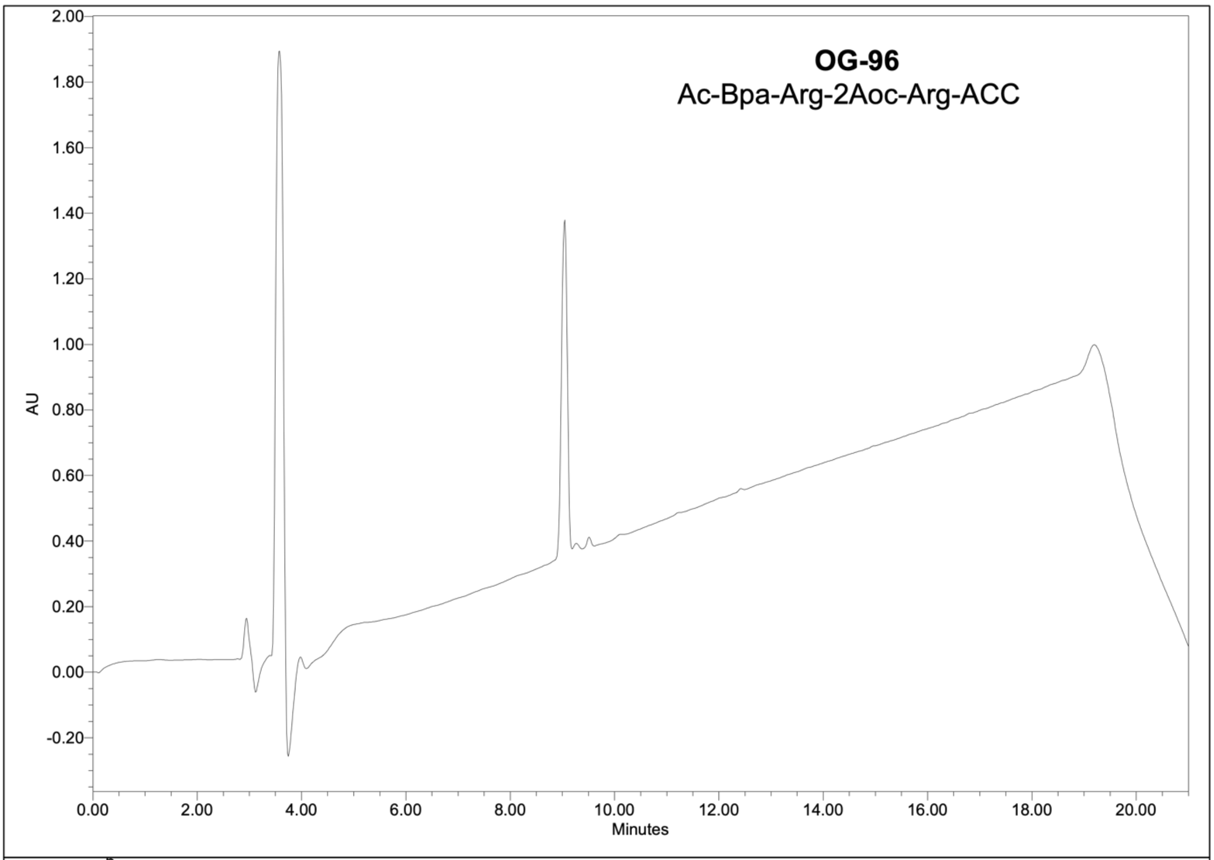
OG-96
Ac-Bpa-Arg-2Aoc-Arg-ACC
AU
Minutes
0.00
2.00
4.00
6.00
8.00
10.00
12.00
14.00
16.00
18.00
20.00
-0.20
0.00
0.20
0.40
0.60
0.80
1.00
1.20
1.40
1.60
1.80
2.00

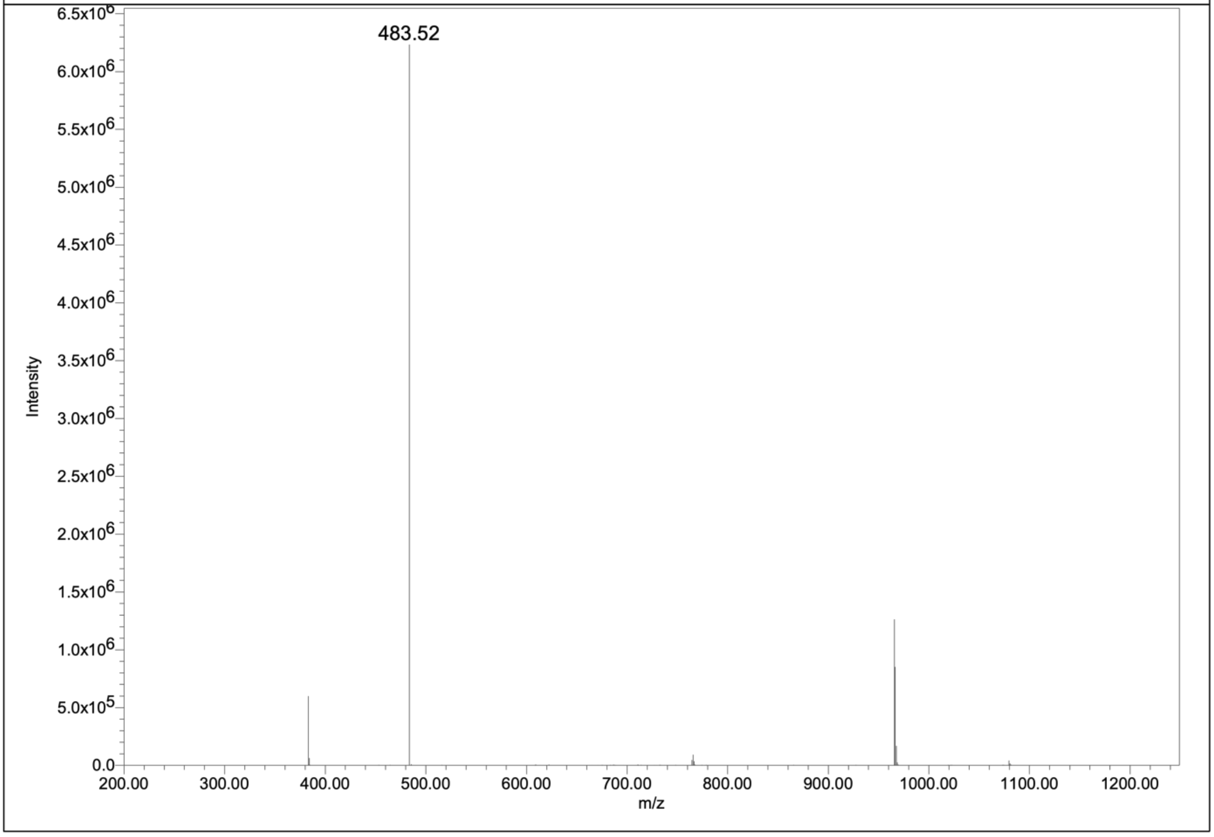
483.52
Intensity
m/z
200.00
300.00
400.00
500.00
600.00
700.00
800.00
900.00
1000.00
1100.00
1200.00
0.0
5.0x10^5
1.0x10^6
1.5x10^6
2.0x10^6
2.5x10^6
3.0x10^6
3.5x10^6
4.0x10^6
4.5x10^6
5.0x10^6
5.5x10^6
6.0x10^6
6.5x10^6

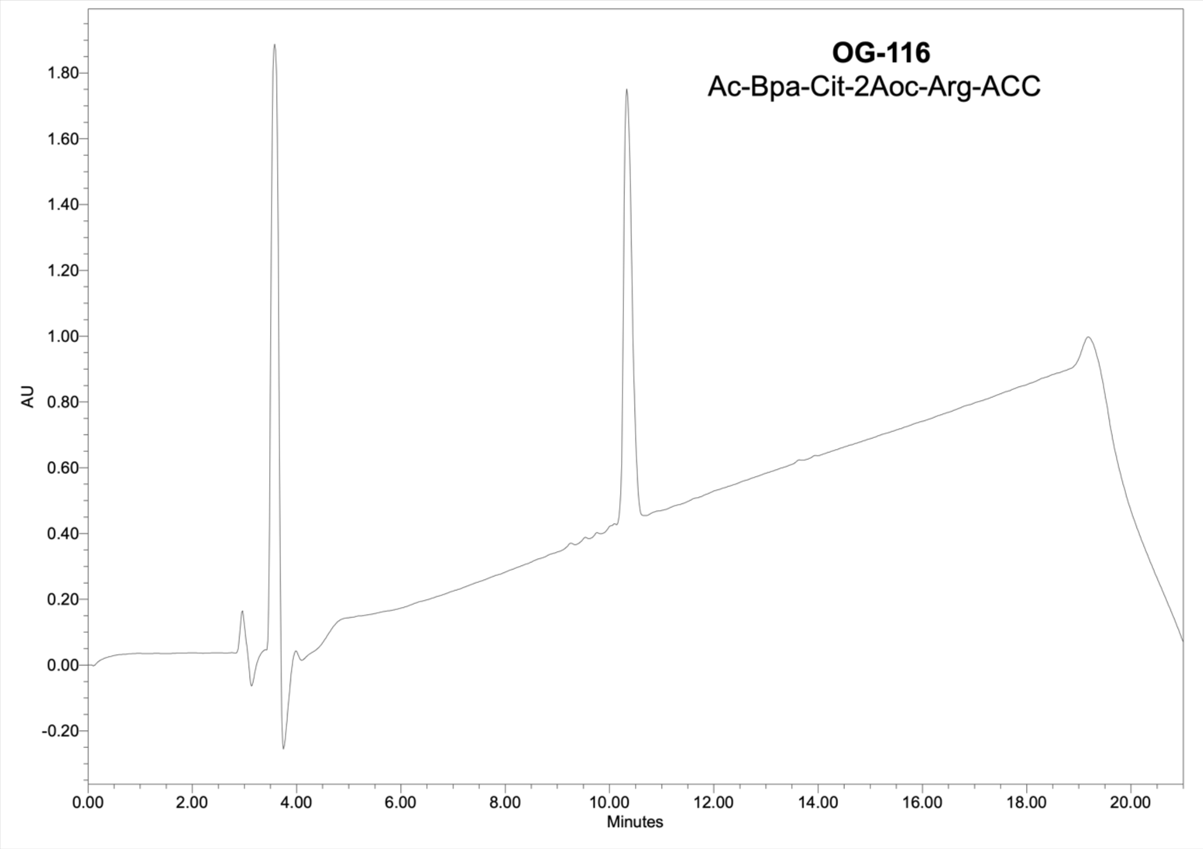
OG-116
Ac-Bpa-Cit-2Aoc-Arg-ACC
AU
Minutes
1.80
1.60
1.40
1.20
1.00
0.80
0.60
0.40
0.20
0.00
-0.20
0.00
2.00
4.00
6.00
8.00
10.00
12.00
14.00
16.00
18.00
20.00

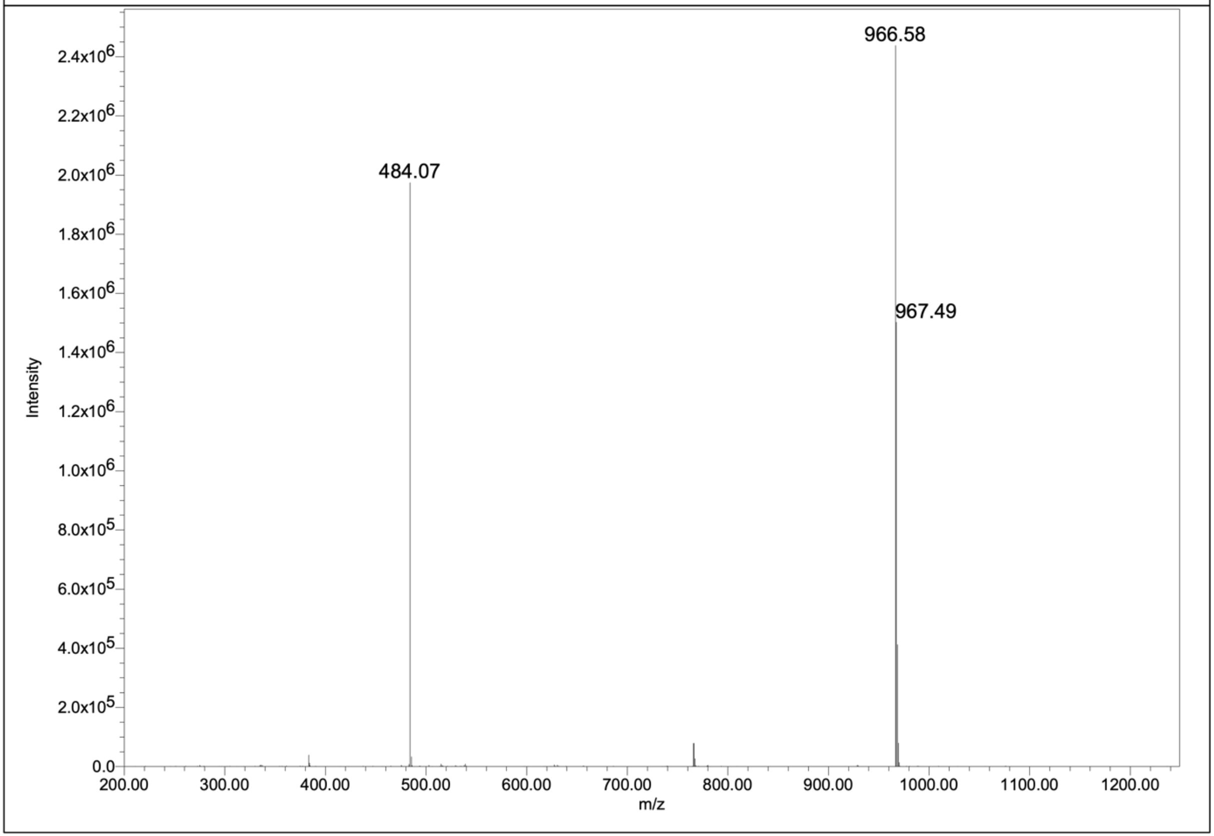
966.58
484.07
967.49
Intensity
m/z
2.4x10^6
2.2x10^6
2.0x10^6
1.8x10^6
1.6x10^6
1.4x10^6
1.2x10^6
1.0x10^6
8.0x10^5
6.0x10^5
4.0x10^5
2.0x10^5
0.0
200.00
300.00
400.00
500.00
600.00
700.00
800.00
900.00
1000.00
1100.00
1200.00

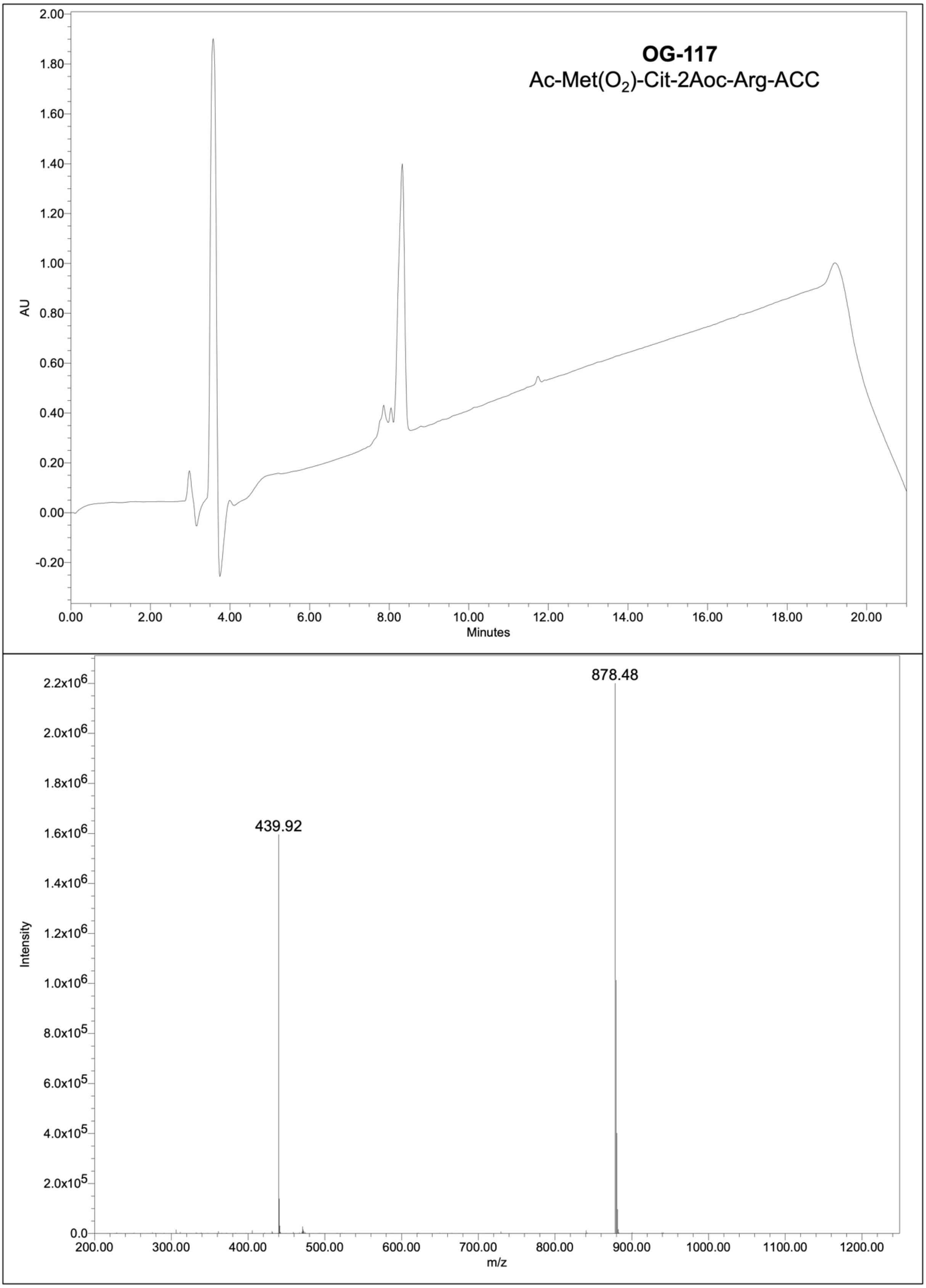
OG-117
Ac-Met(O2)-Cit-2Aoc-Arg-ACC
AU
Minutes
878.48
439.92
Intensity
m/z

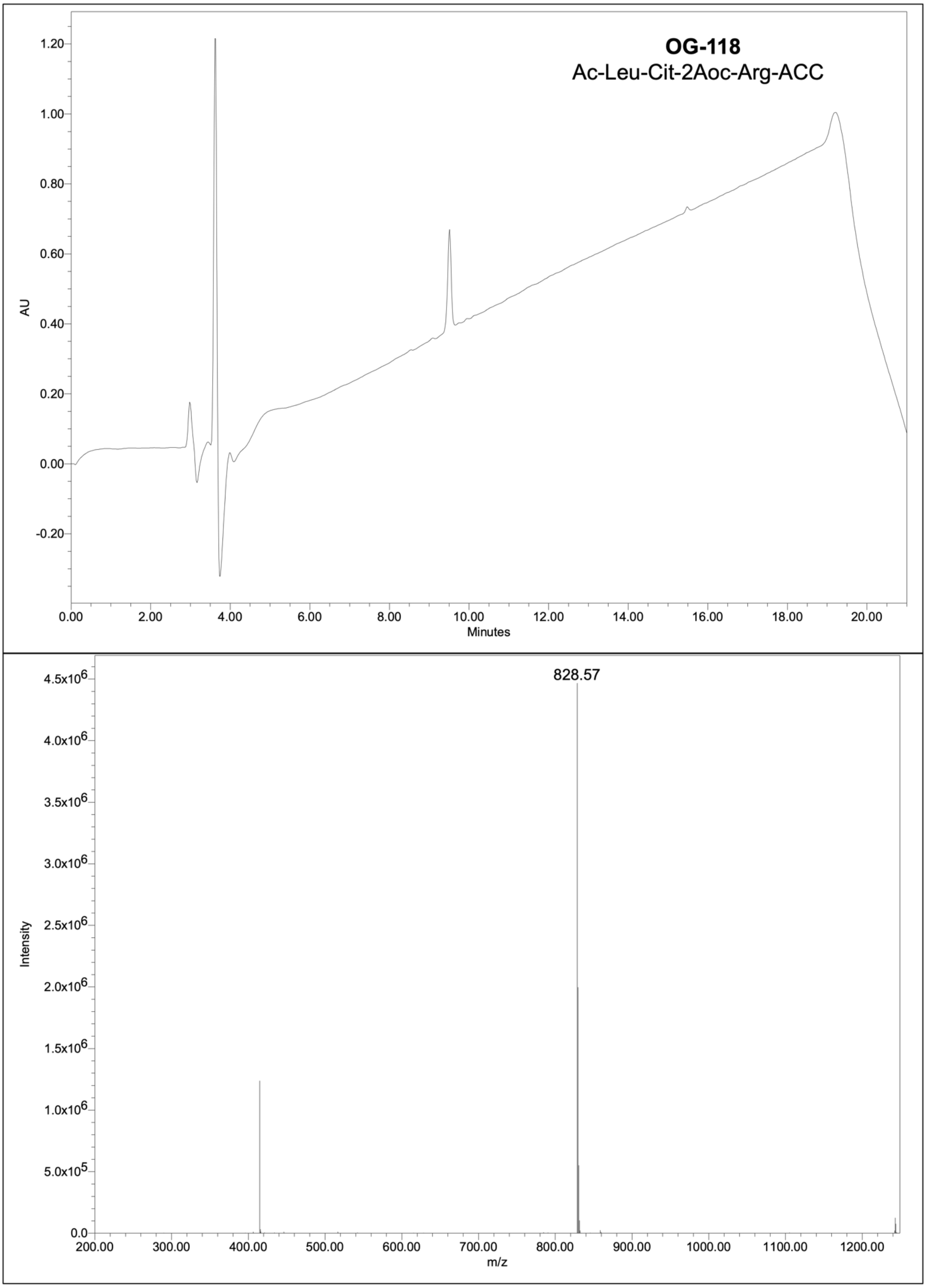
OG-118
Ac-Leu-Cit-2Aoc-Arg-ACC
AU
Minutes
828.57
Intensity
m/z

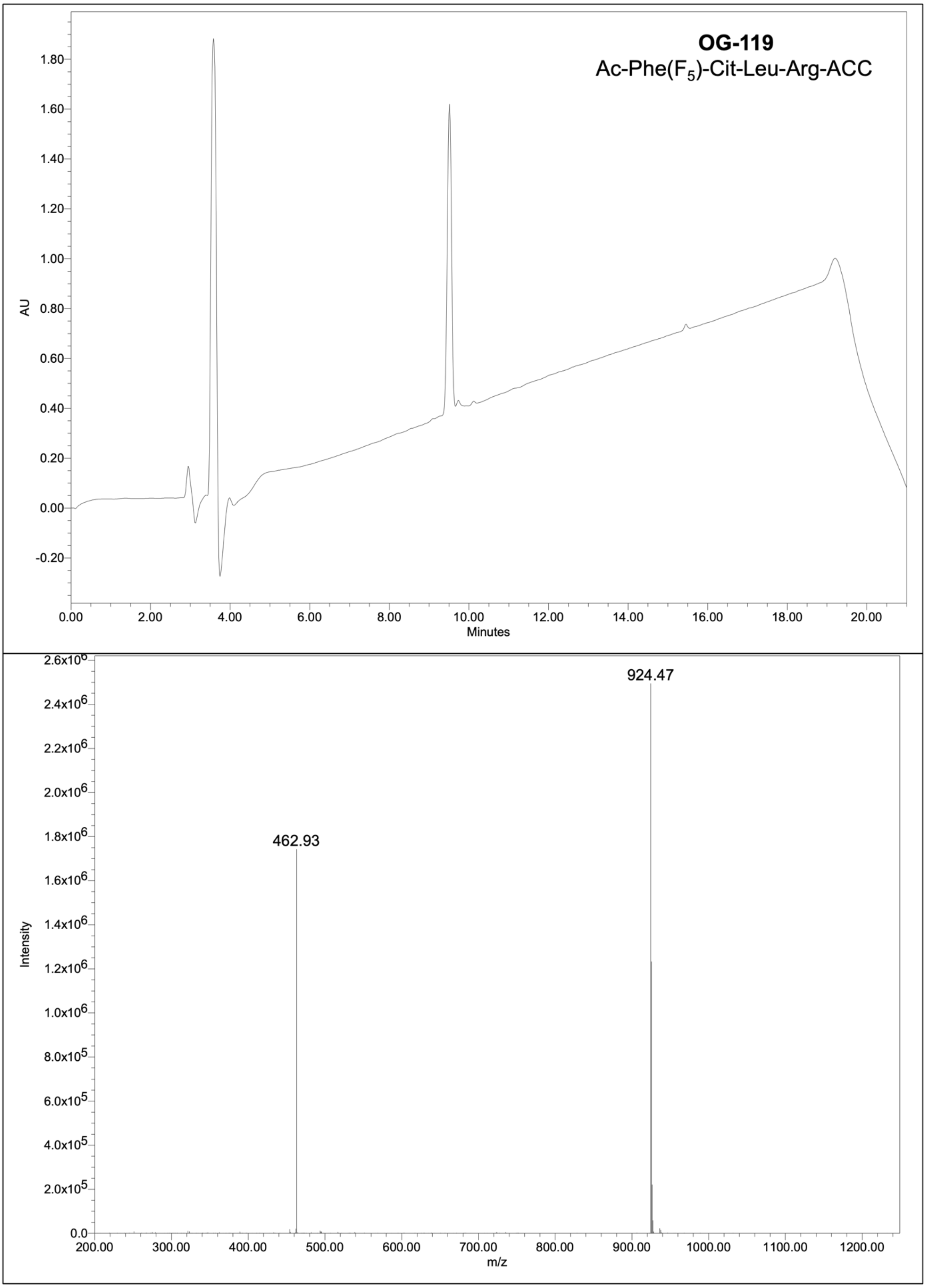
OG-119
Ac-Phe(F5)-Cit-Leu-Arg-ACC
AU
Minutes
924.47
462.93
Intensity
m/z

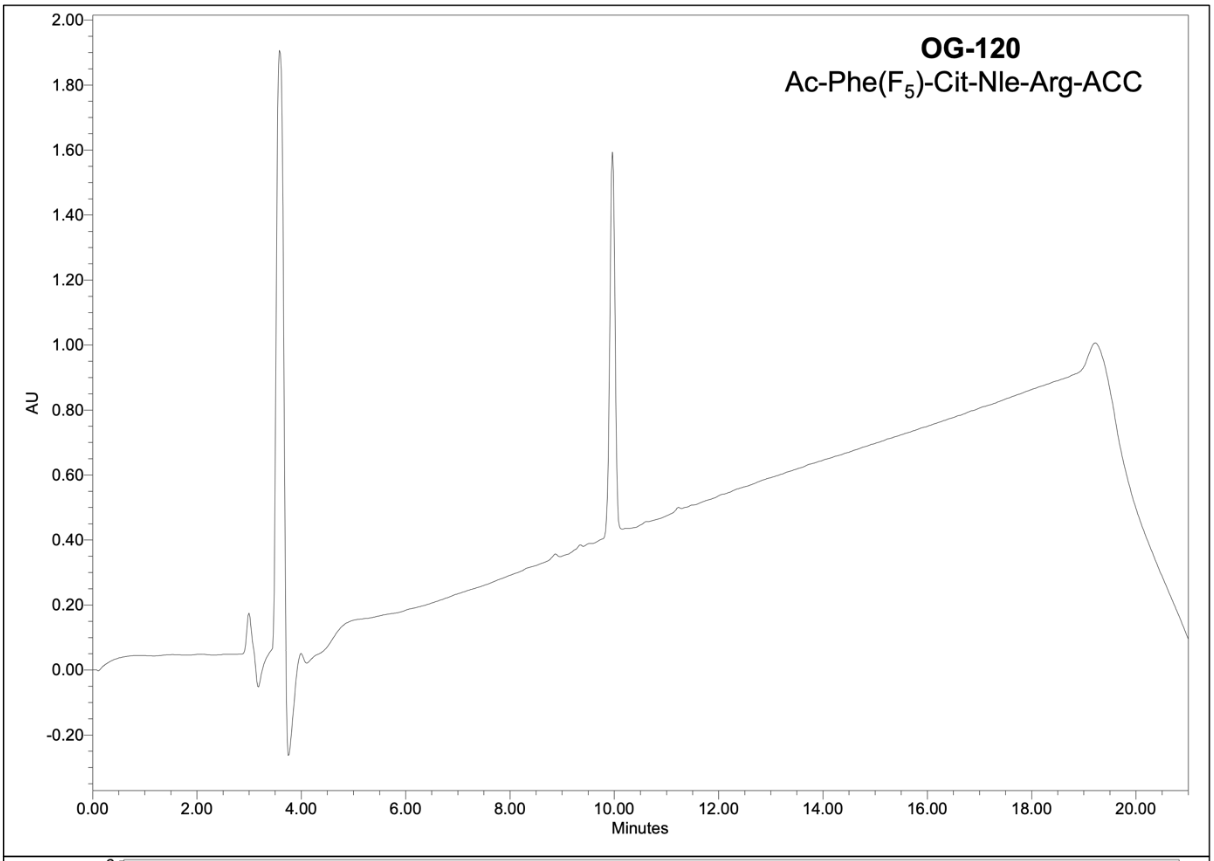
OG-120
Ac-Phe(F5)-Cit-Nle-Arg-ACC
AU
Minutes

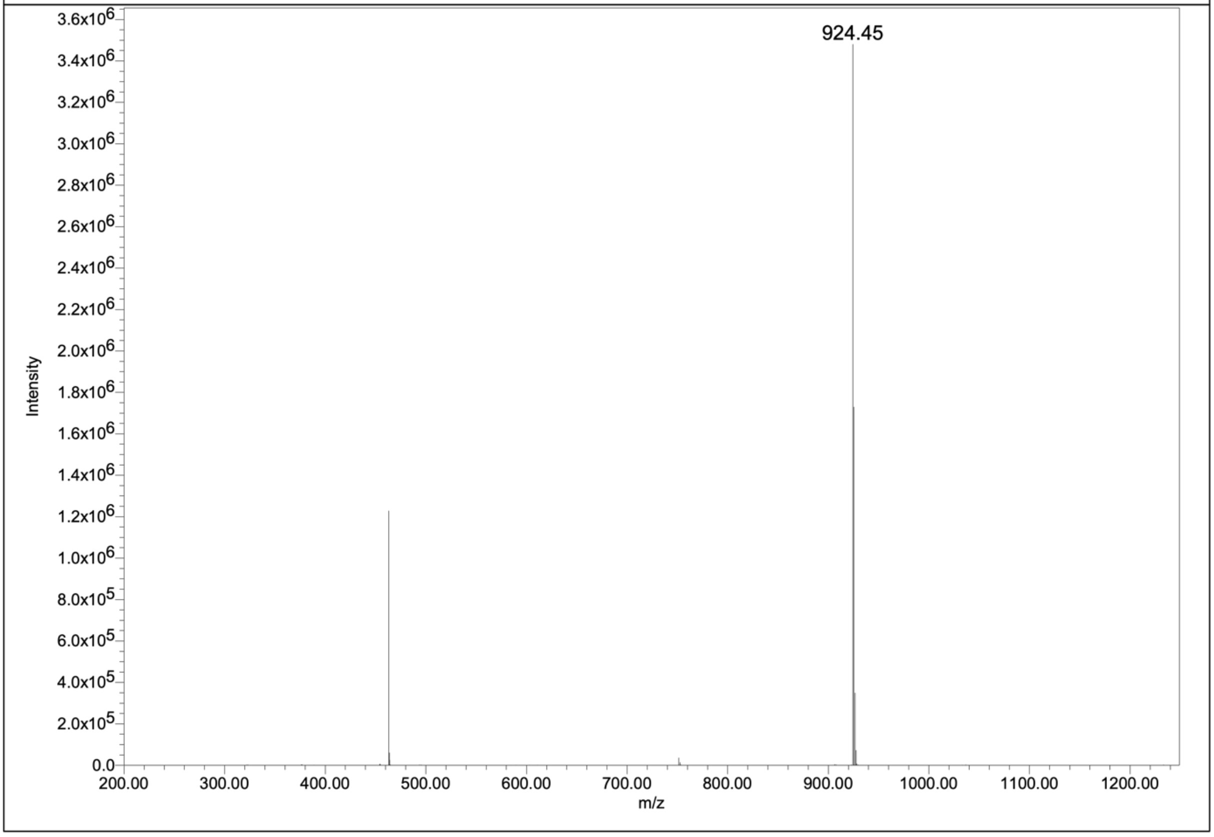
924.45
Intensity
m/z

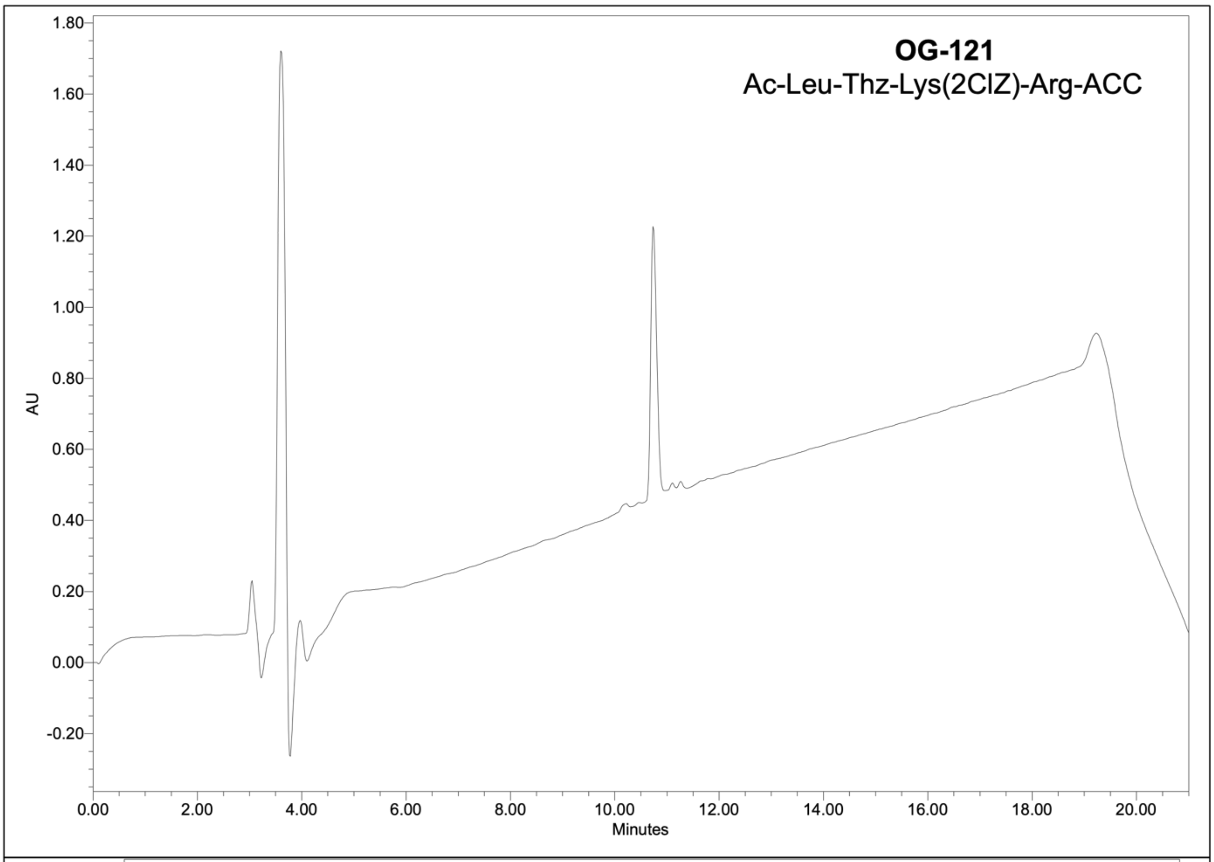

OG-121
Ac-Leu-Thz-Lys(2ClZ)-Arg-ACC
AU
Minutes

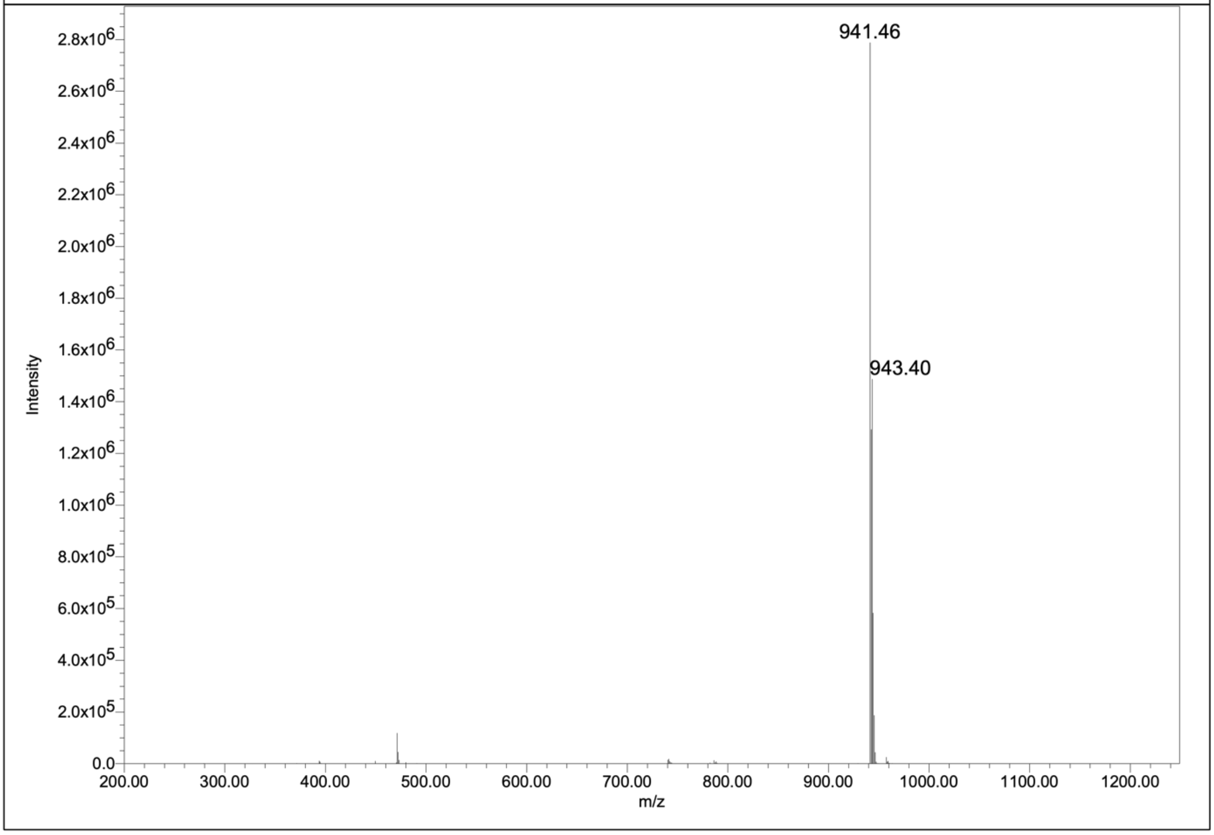

941.46
943.40
Intensity
m/z

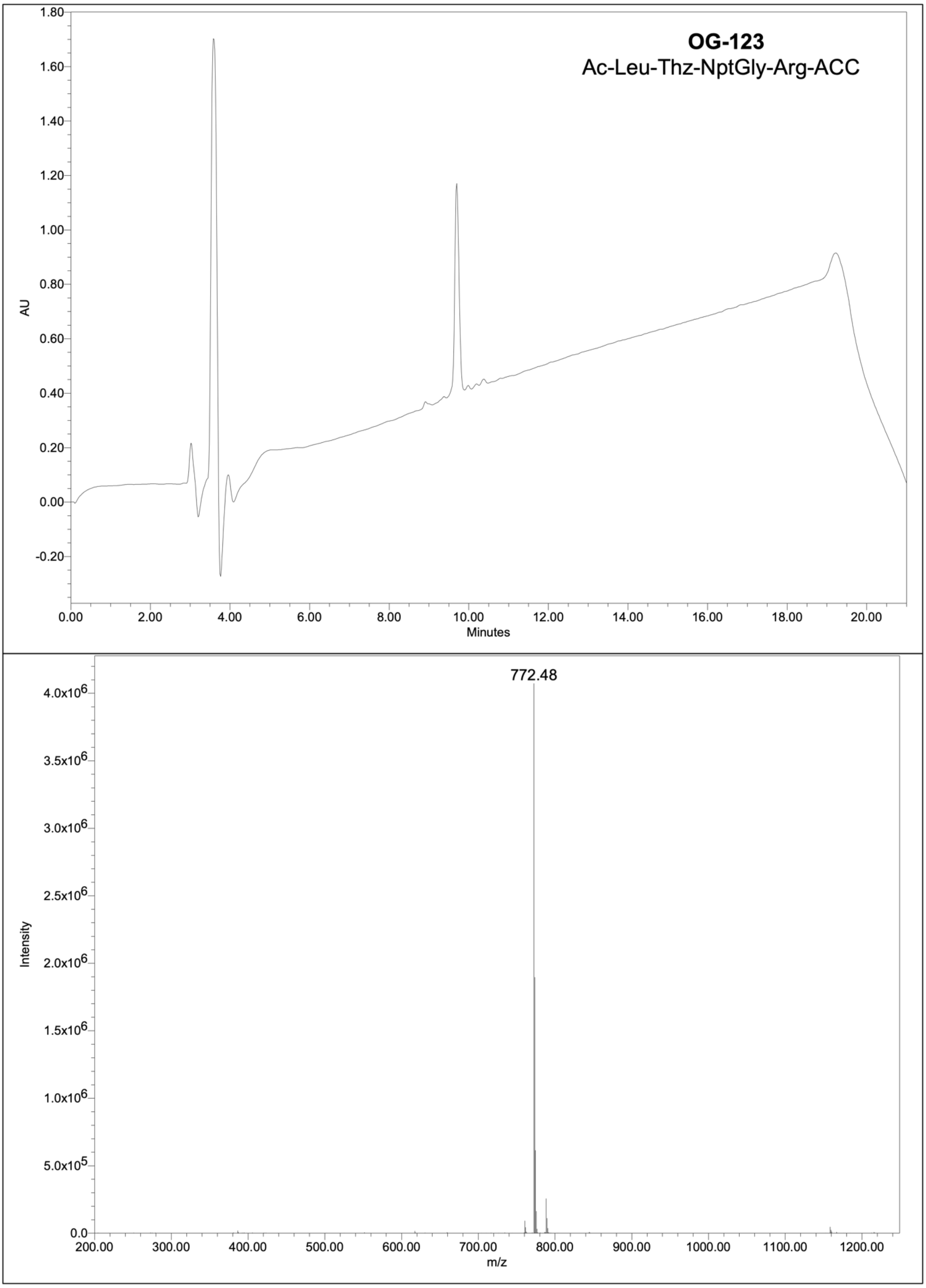

OG-123
Ac-Leu-Thz-NptGly-Arg-ACC
AU
Minutes
772.48
Intensity
m/z

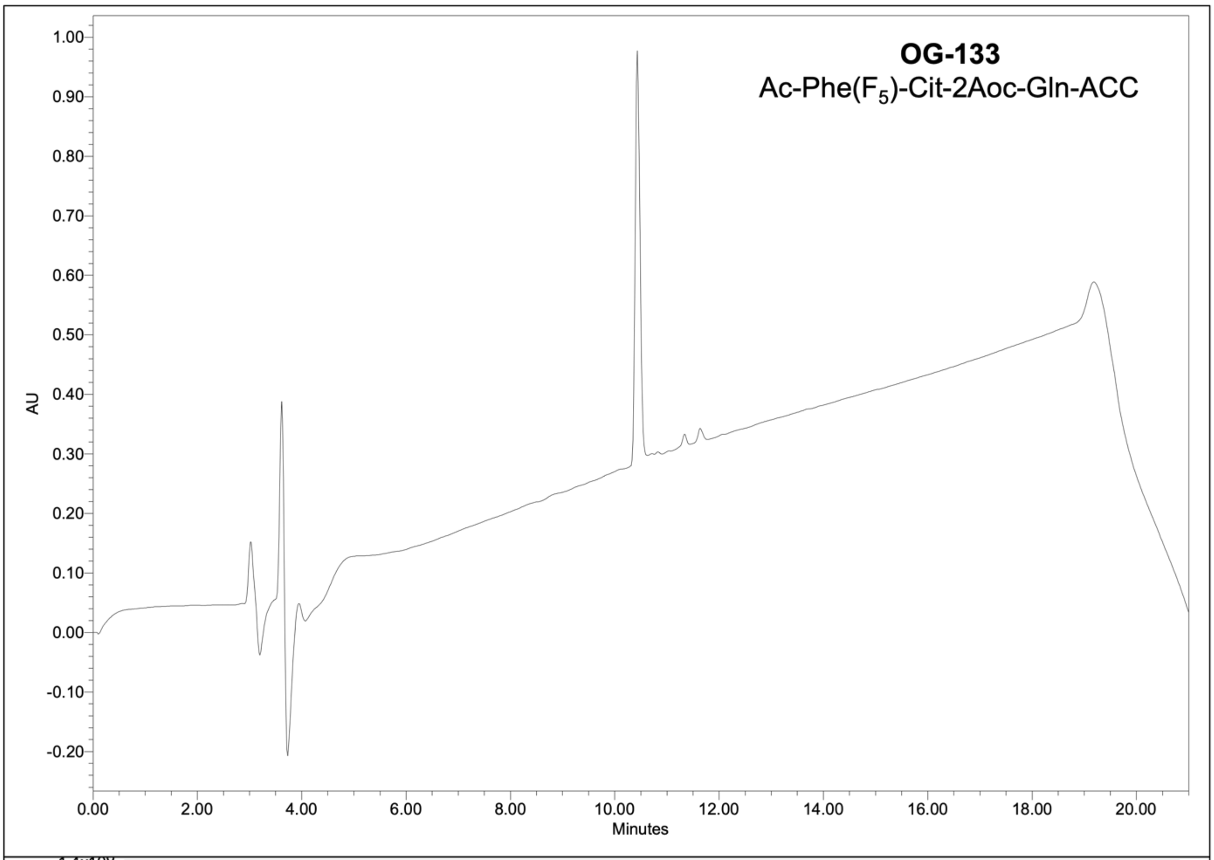
OG-133
Ac-Phe(F5)-Cit-2Aoc-Gln-ACC
AU
Minutes

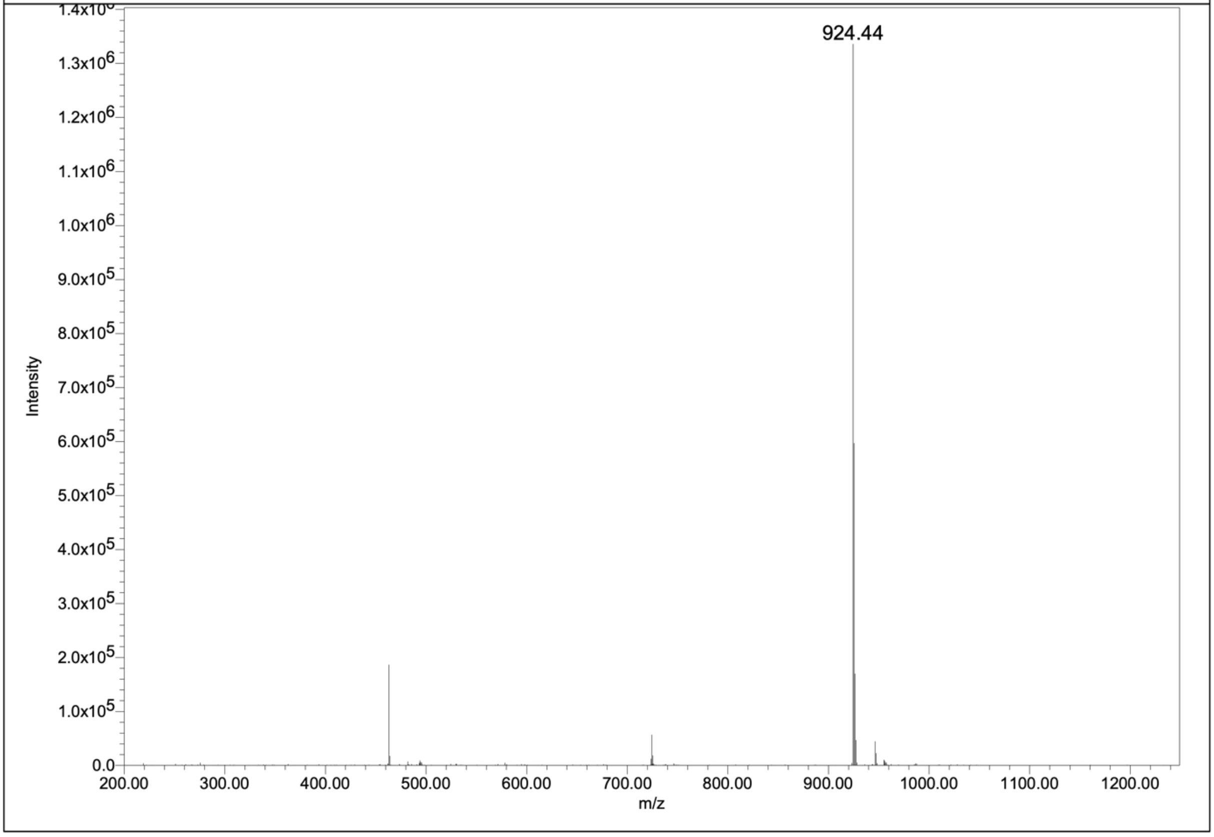
924.44
Intensity
m/z

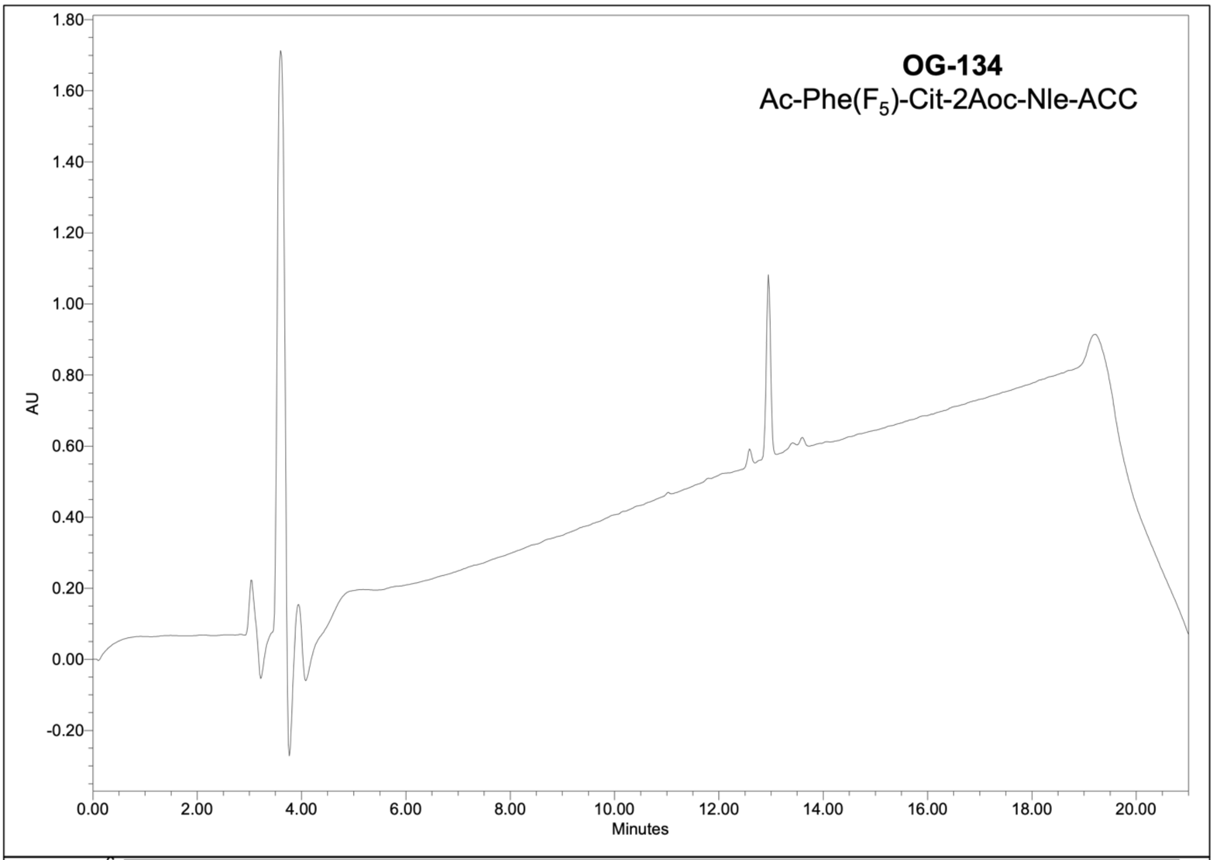

OG-134
Ac-Phe(F5)-Cit-2Aoc-Nle-ACC
AU
Minutes

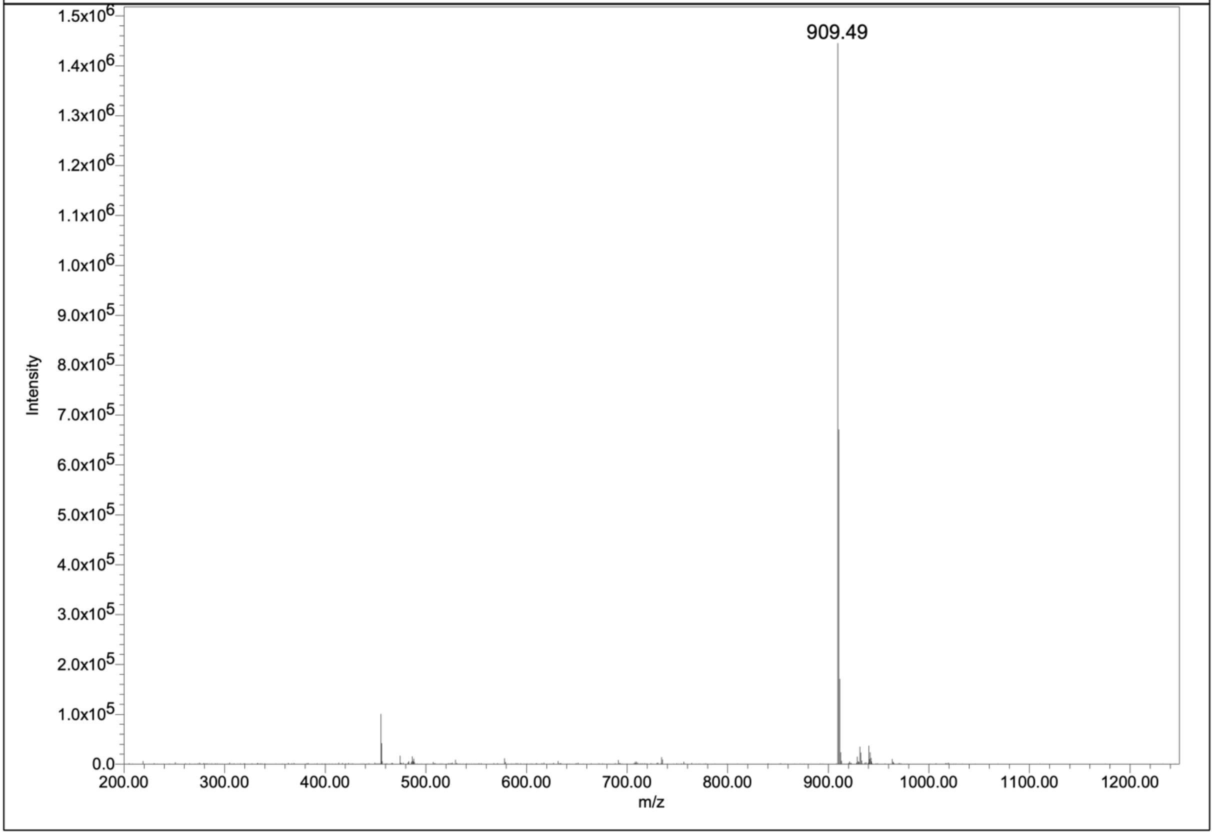

909.49
Intensity
m/z

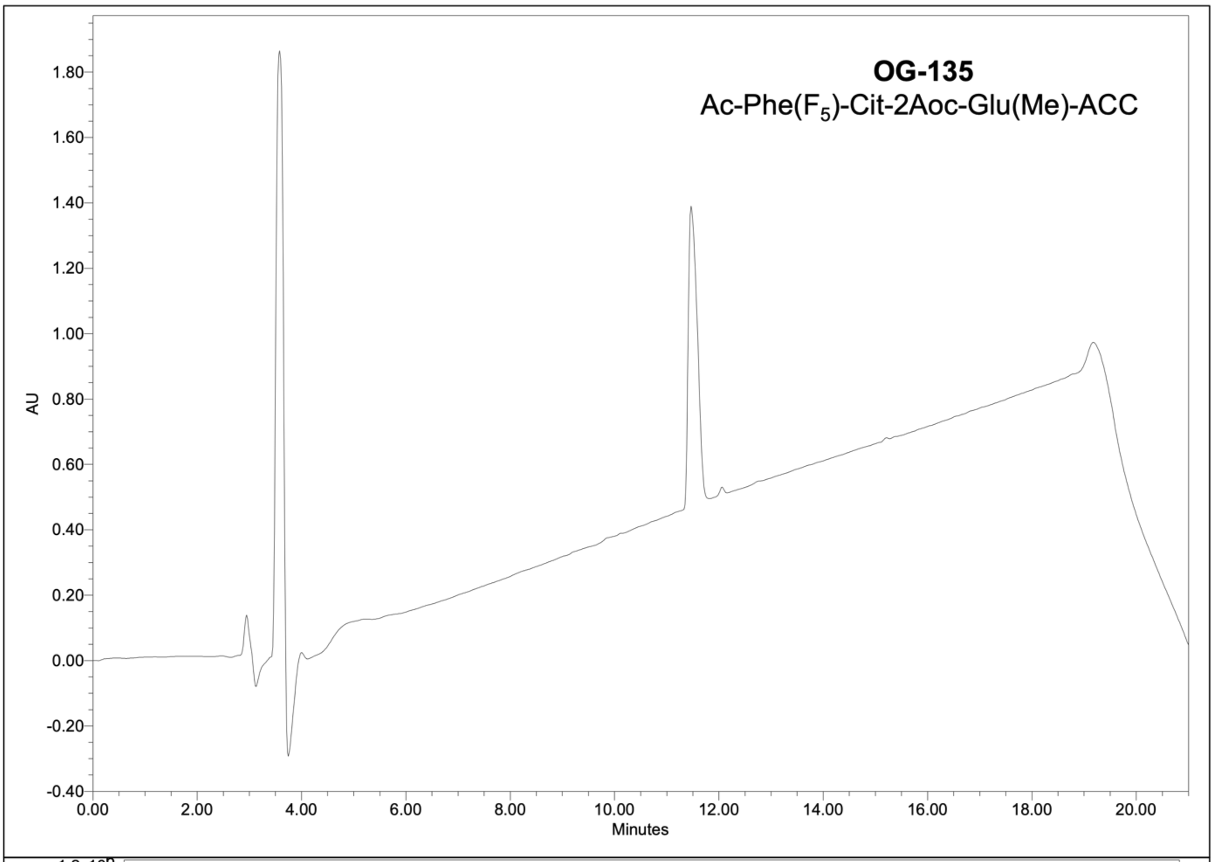

OG-135
Ac-Phe(F5)-Cit-2Aoc-Glu(Me)-ACC
AU
Minutes

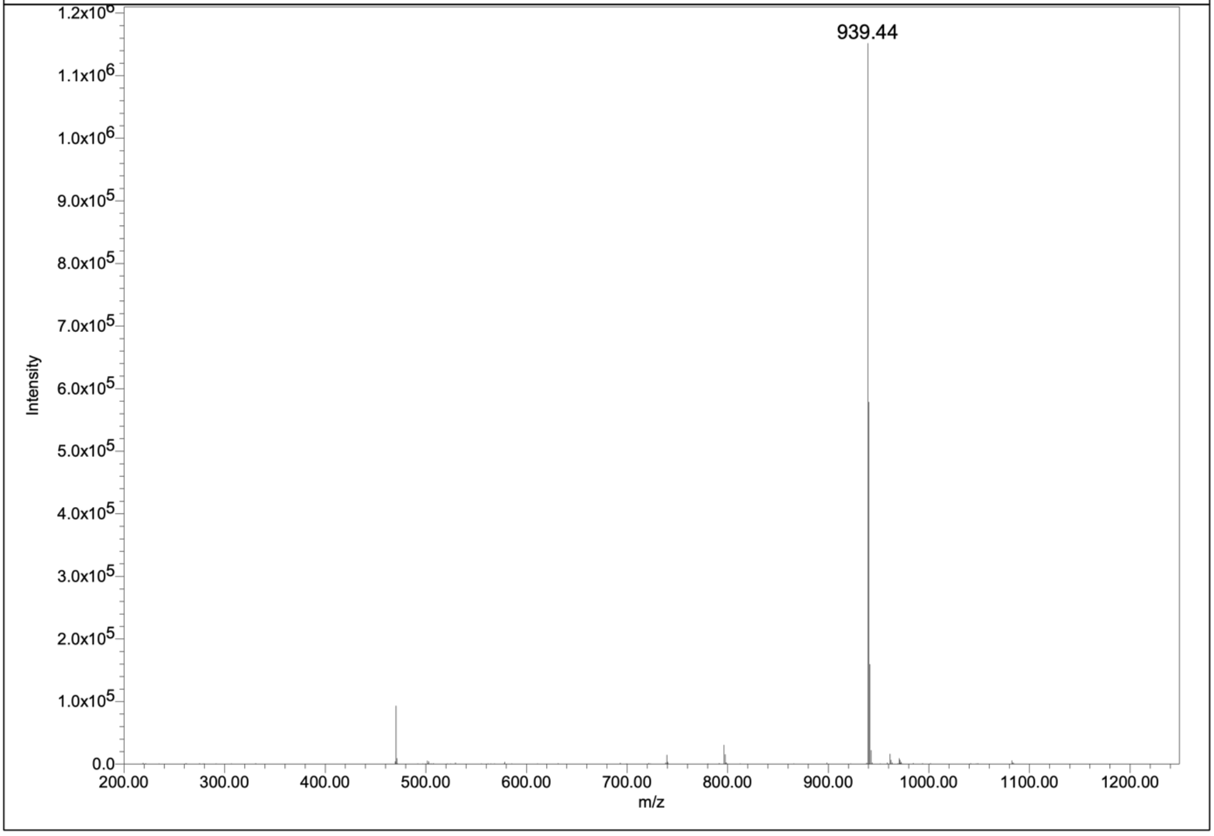

939.44
Intensity
m/z

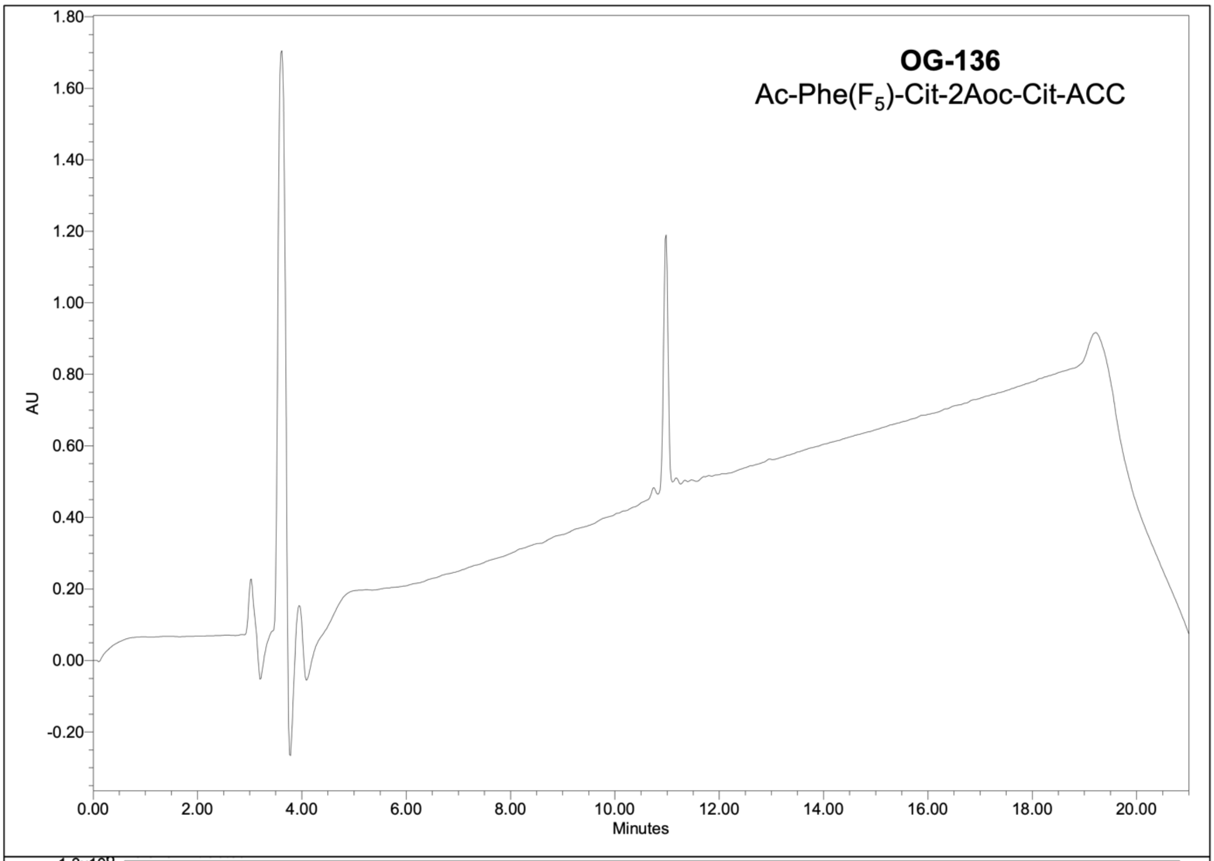
OG-136
Ac-Phe(F5)-Cit-2Aoc-Cit-ACC
AU
Minutes

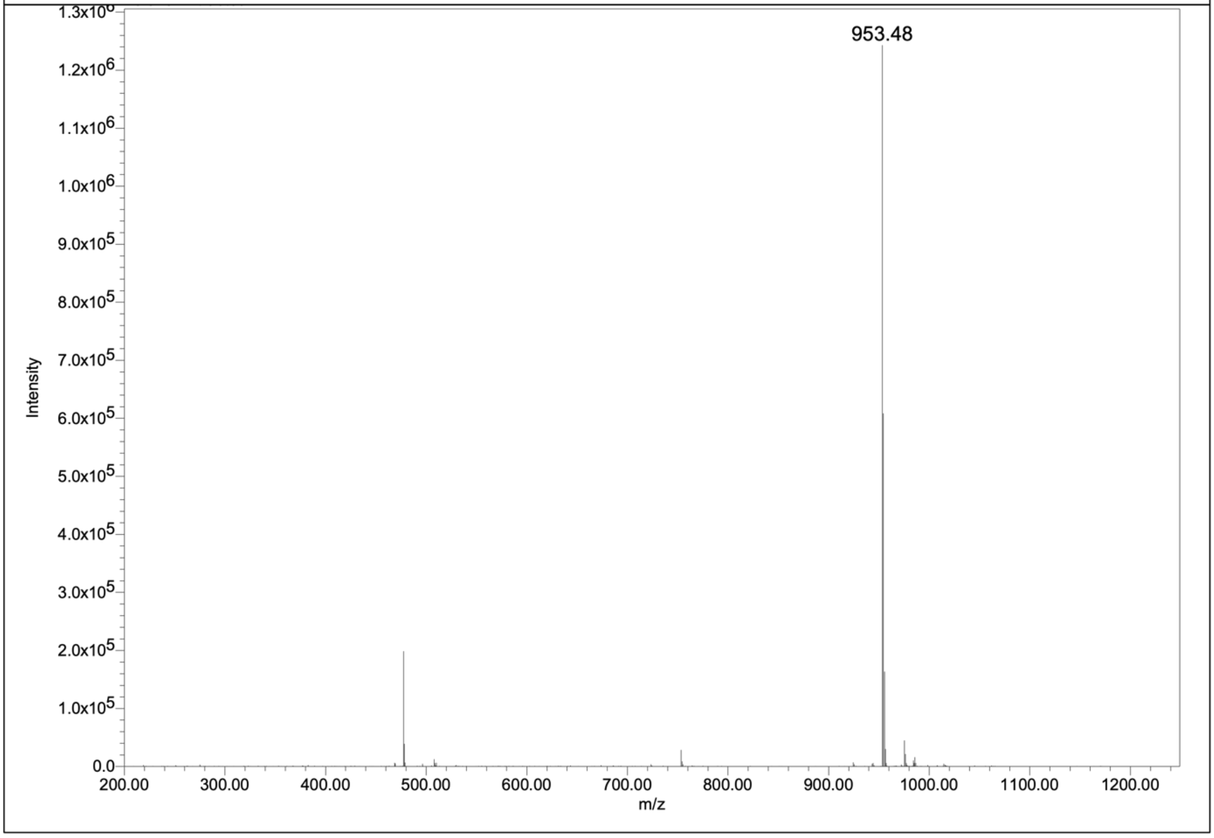
953.48
Intensity
m/z

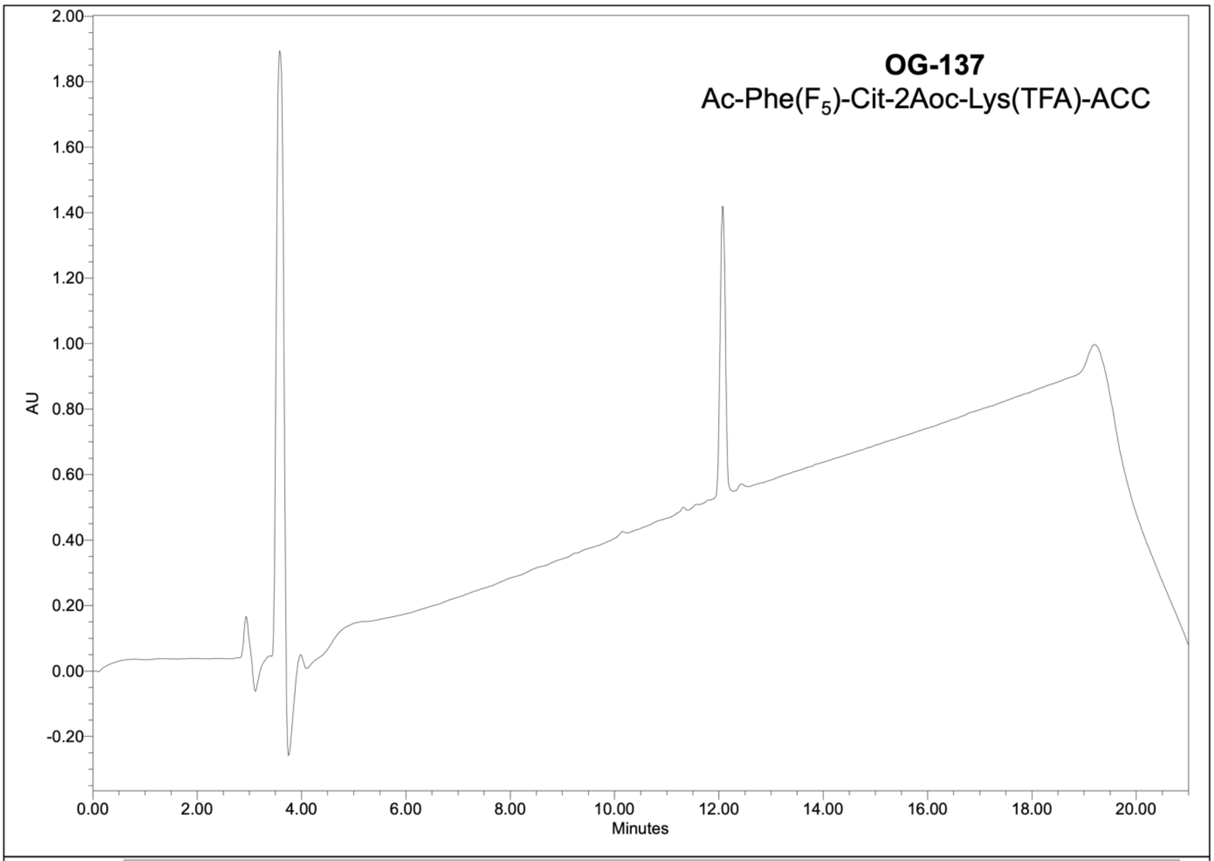
OG-137
Ac-Phe(F5)-Cit-2Aoc-Lys(TFA)-ACC
AU
Minutes
2.00
1.80
1.60
1.40
1.20
1.00
0.80
0.60
0.40
0.20
0.00
-0.20
0.00
2.00
4.00
6.00
8.00
10.00
12.00
14.00
16.00
18.00
20.00

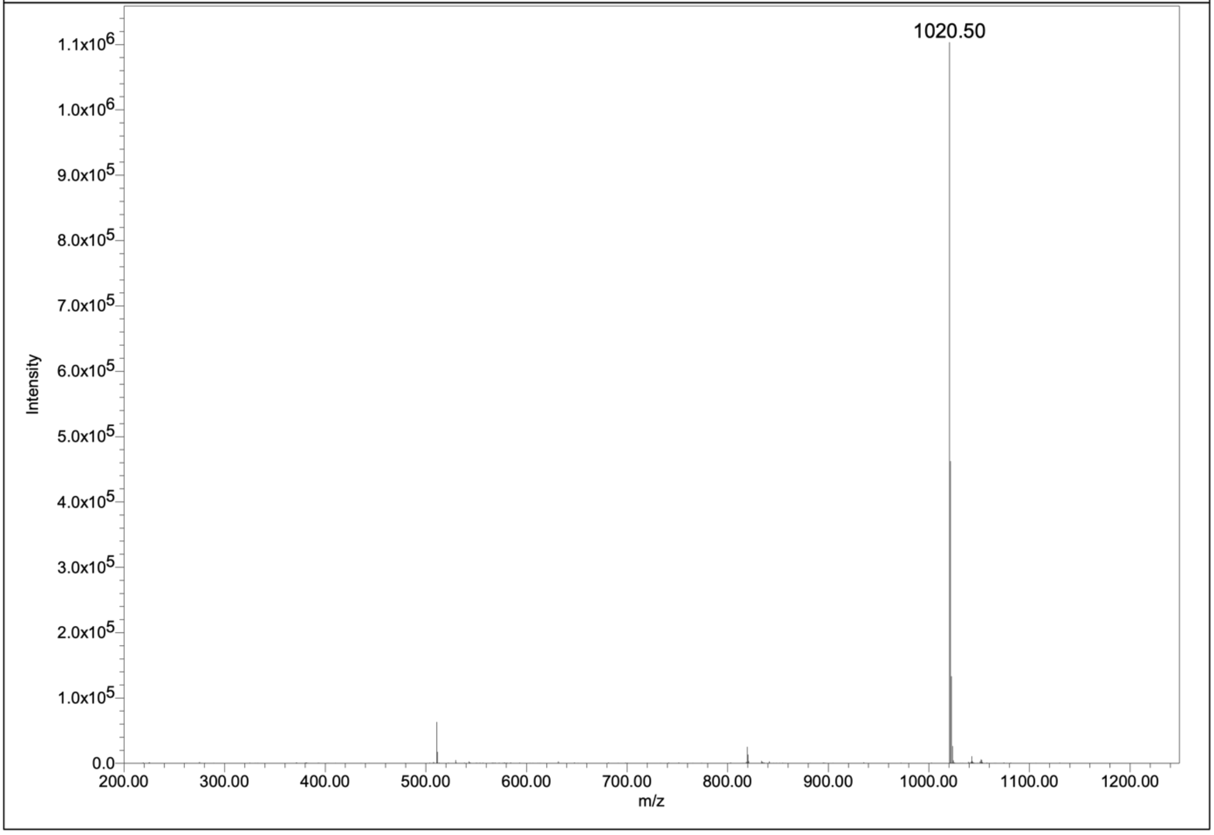
1020.50
Intensity
m/z
1.1x10^6
1.0x10^6
9.0x10^5
8.0x10^5
7.0x10^5
6.0x10^5
5.0x10^5
4.0x10^5
3.0x10^5
2.0x10^5
1.0x10^5
0.0
200.00
300.00
400.00
500.00
600.00
700.00
800.00
900.00
1000.00
1100.00
1200.00

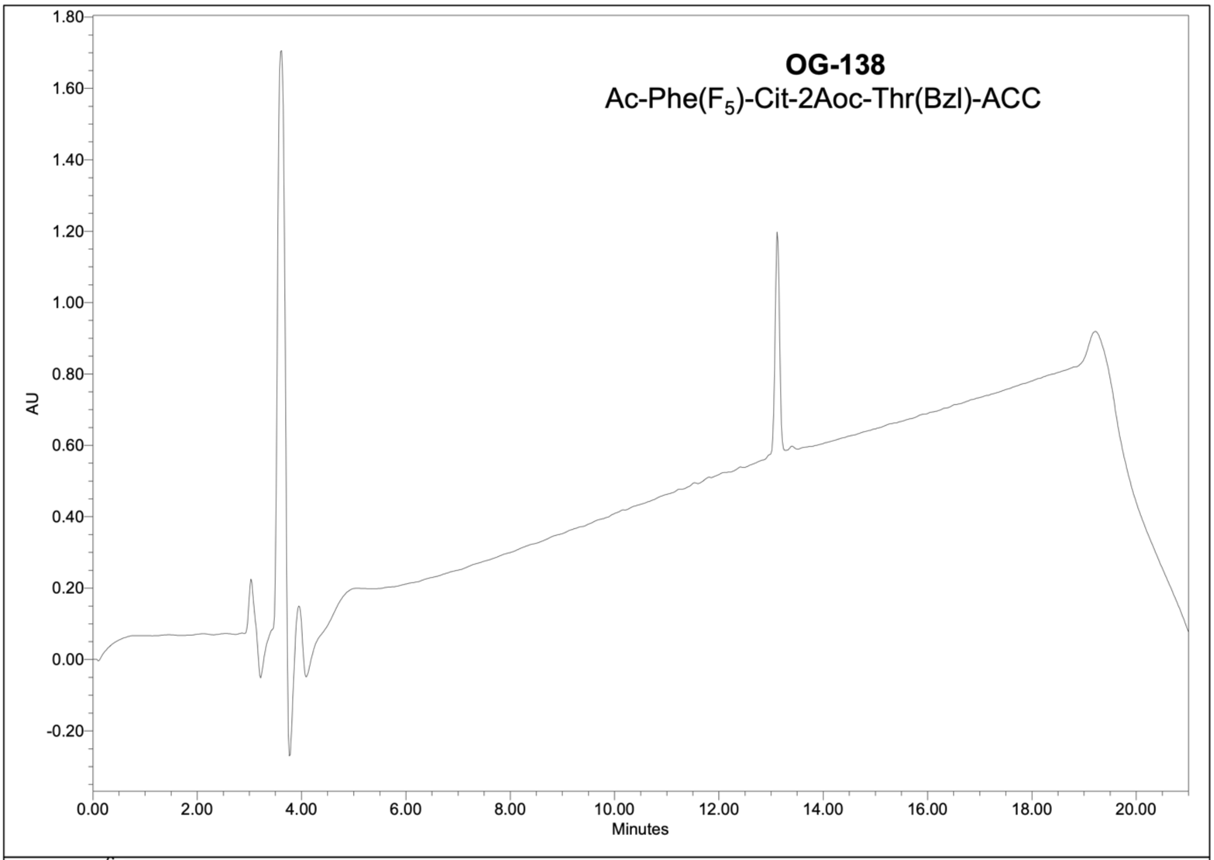
OG-138
Ac-Phe(F5)-Cit-2Aoc-Thr(Bzl)-ACC
AU
Minutes

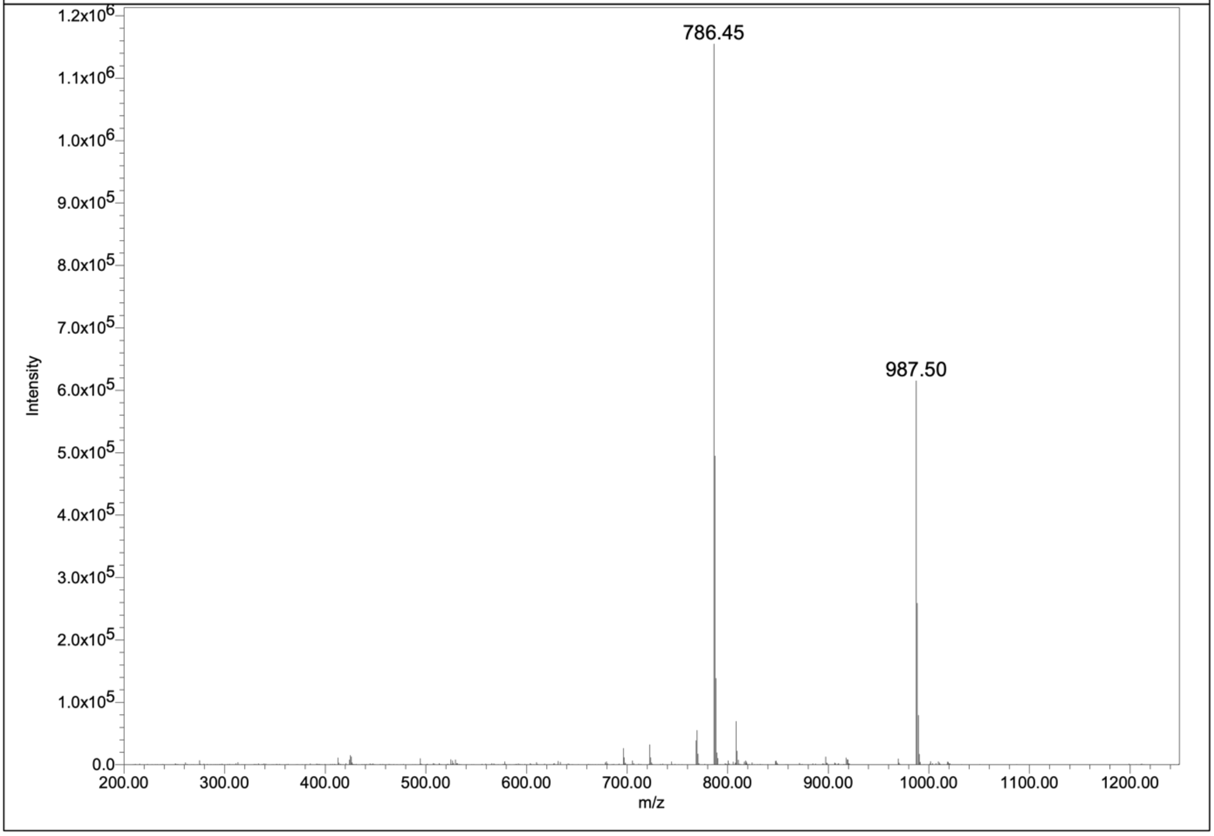
786.45
987.50
Intensity
m/z

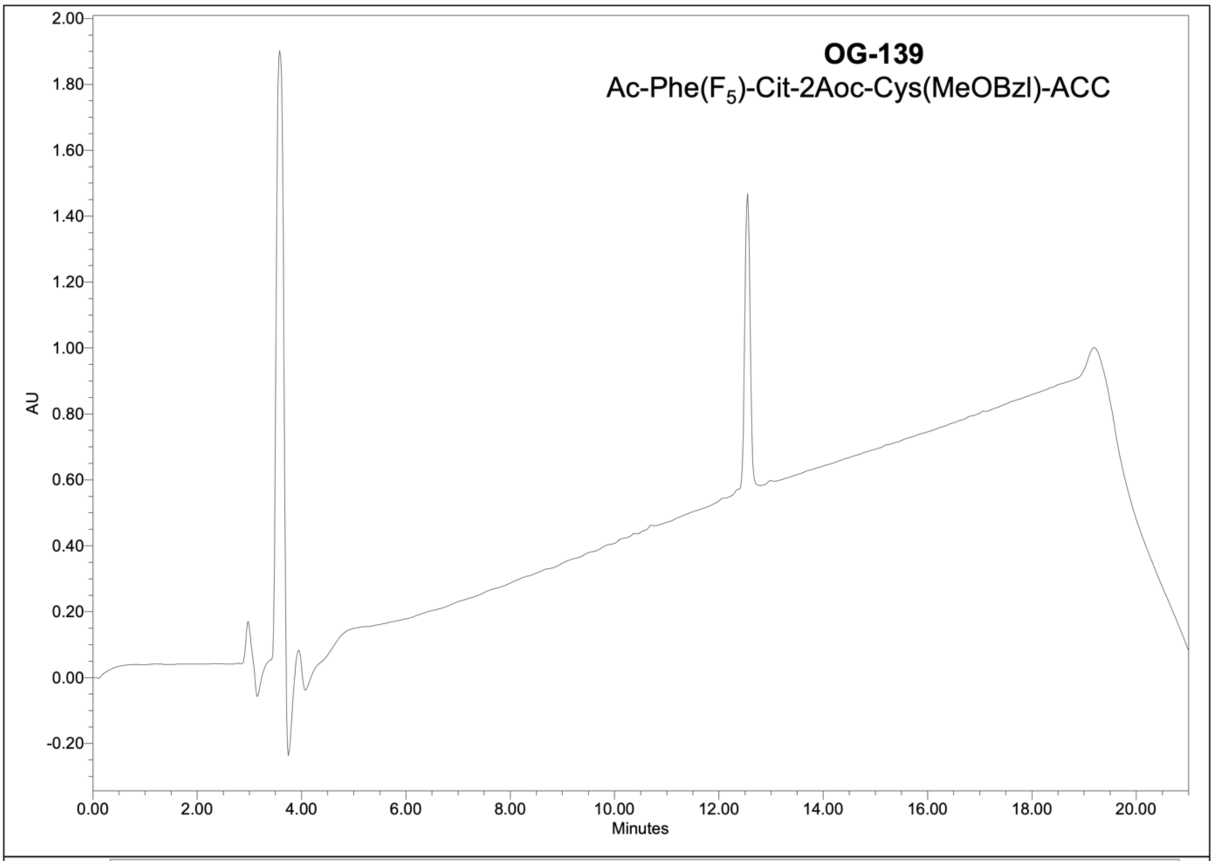
OG-139
Ac-Phe(F5)-Cit-2Aoc-Cys(MeOBzl)-ACC
AU
Minutes
2.00
1.80
1.60
1.40
1.20
1.00
0.80
0.60
0.40
0.20
0.00
-0.20
0.00
2.00
4.00
6.00
8.00
10.00
12.00
14.00
16.00
18.00
20.00

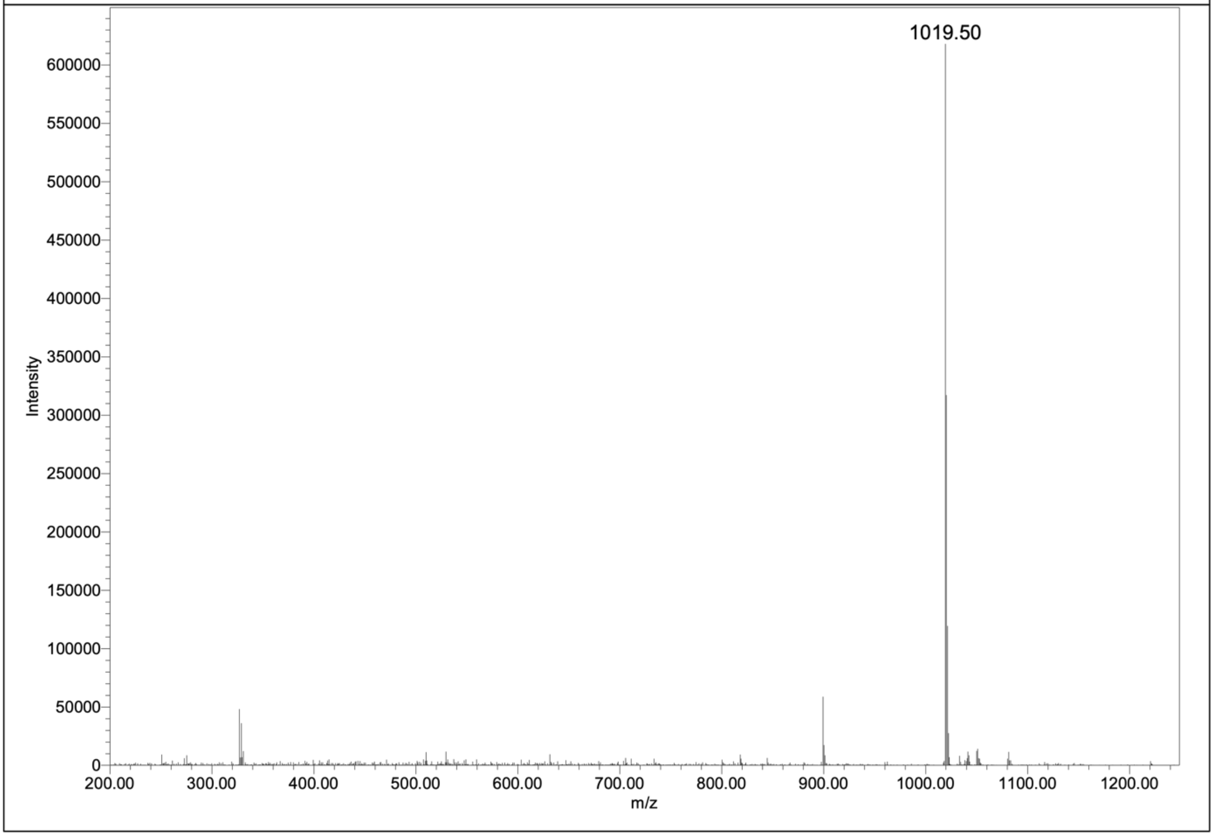
1019.50
Intensity
m/z
600000
550000
500000
450000
400000
350000
300000
250000
200000
150000
100000
50000
0
200.00
300.00
400.00
500.00
600.00
700.00
800.00
900.00
1000.00
1100.00
1200.00

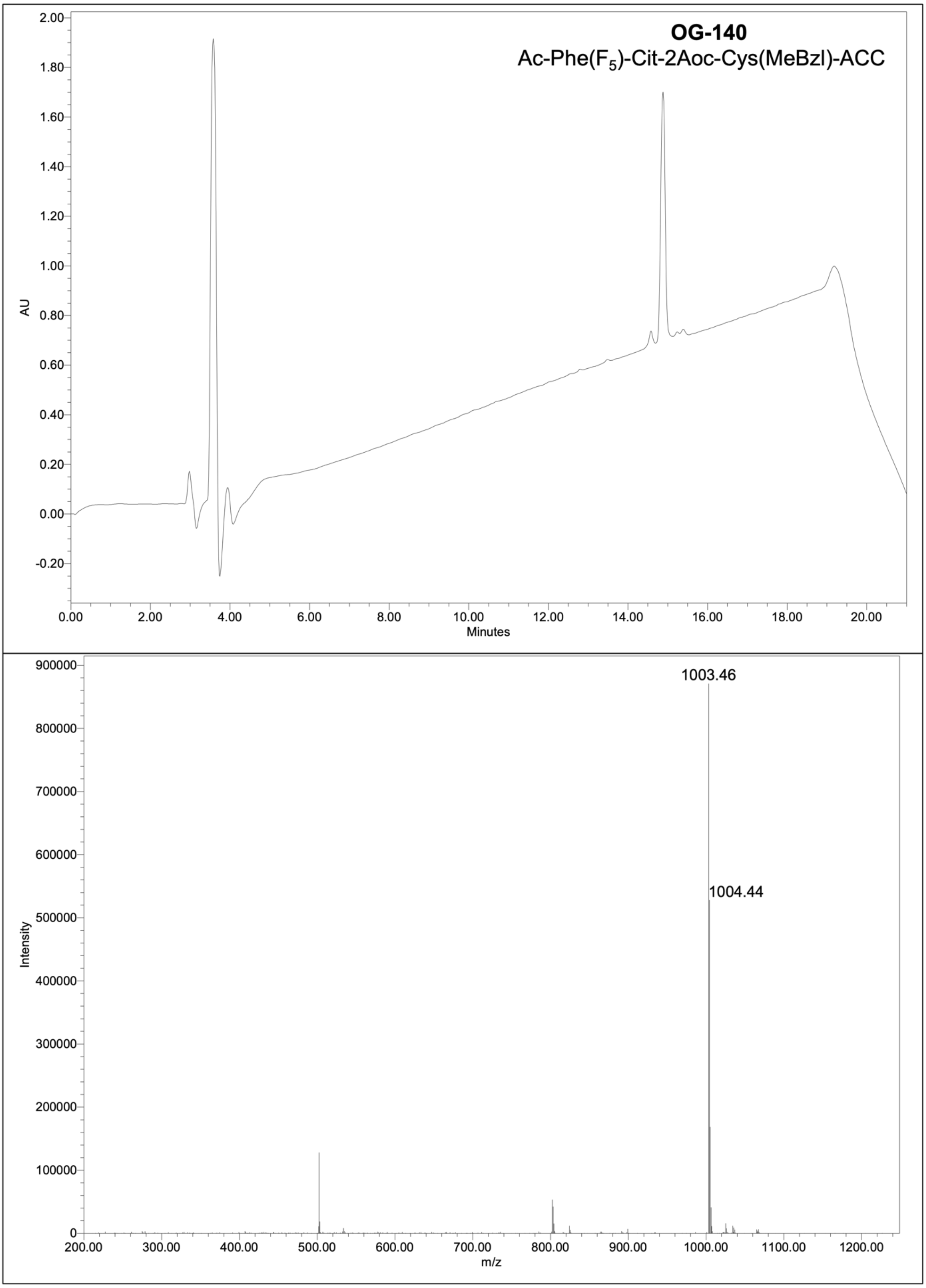

OG-140
Ac-Phe(F5)-Cit-2Aoc-Cys(MeBzl)-ACC
AU
Minutes
1003.46
1004.44
Intensity
m/z

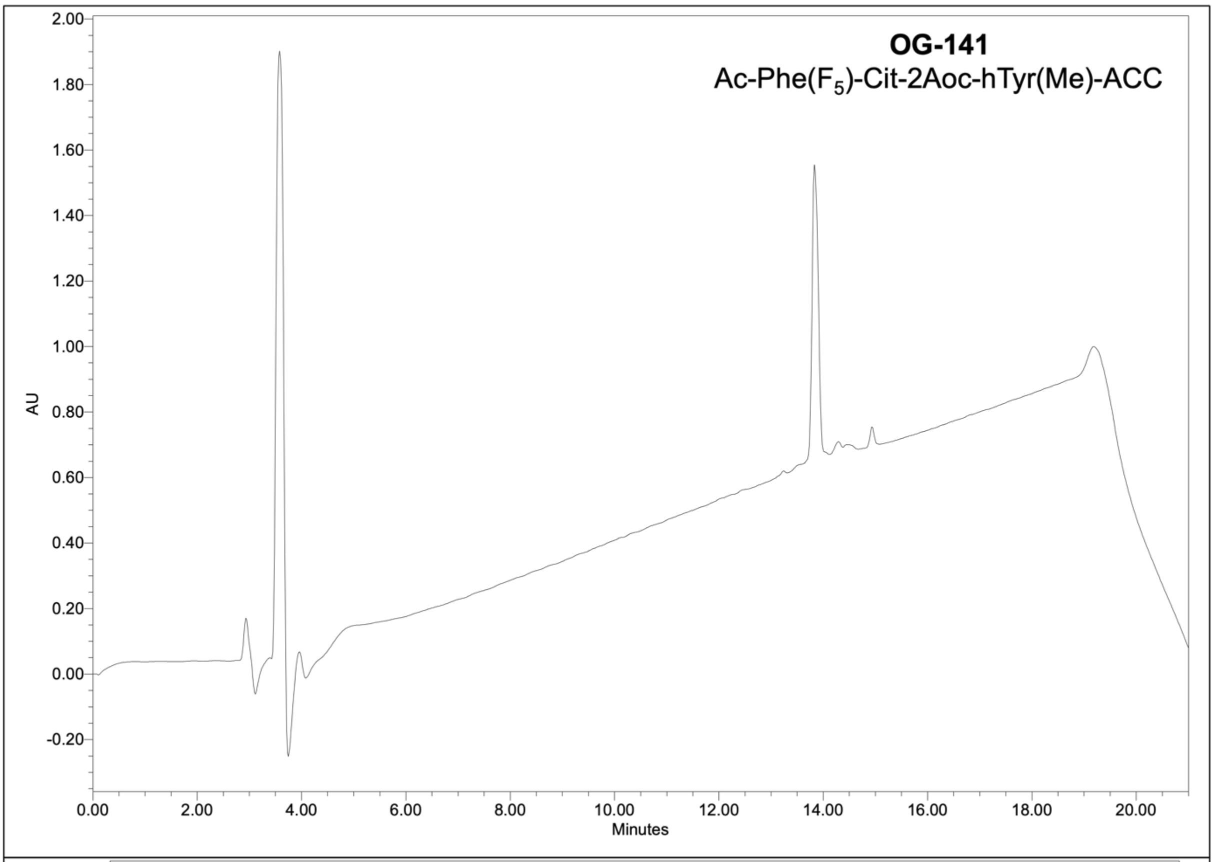

OG-141
Ac-Phe(F$_5$)-Cit-2Aoc-hTyr(Me)-ACC
AU
Minutes
2.00
1.80
1.60
1.40
1.20
1.00
0.80
0.60
0.40
0.20
0.00
-0.20
0.00
2.00
4.00
6.00
8.00
10.00
12.00
14.00
16.00
18.00
20.00

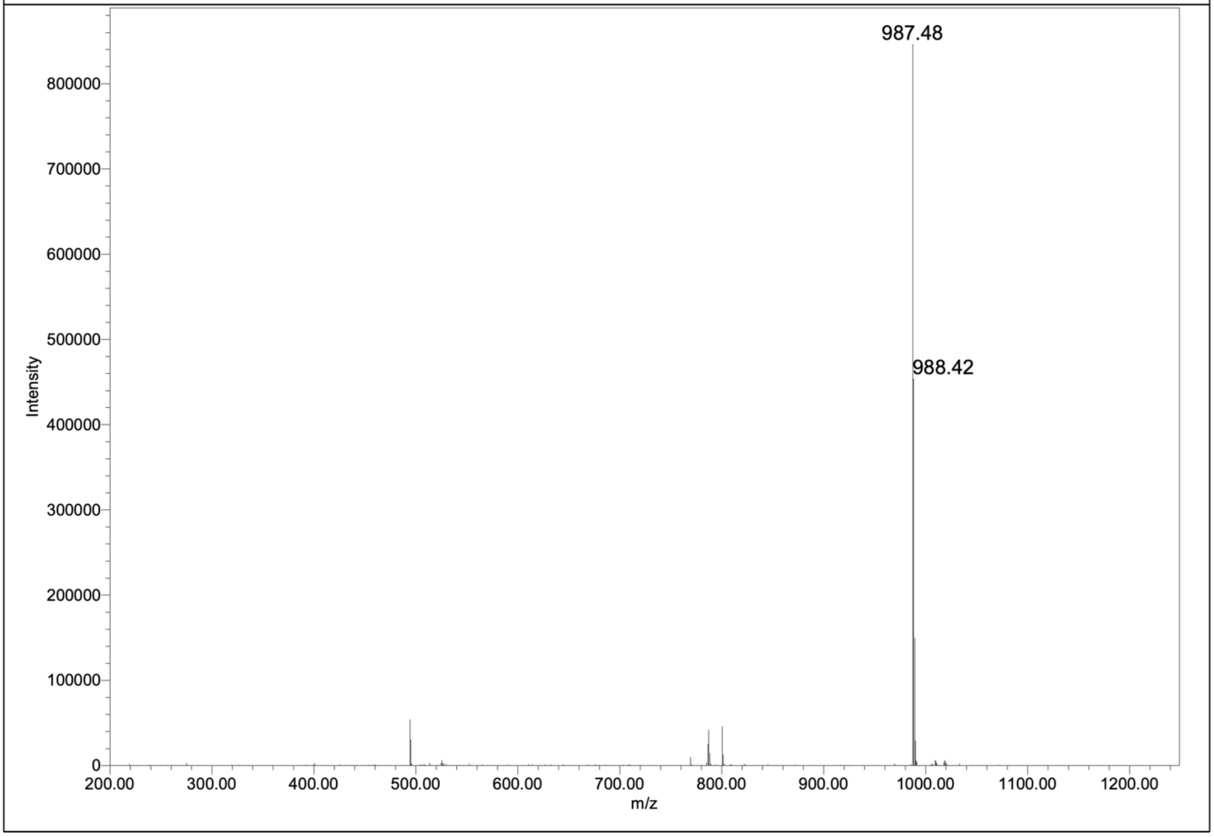

987.48
988.42
Intensity
m/z
800000
700000
600000
500000
400000
300000
200000
100000
0
200.00
300.00
400.00
500.00
600.00
700.00
800.00
900.00
1000.00
1100.00
1200.00

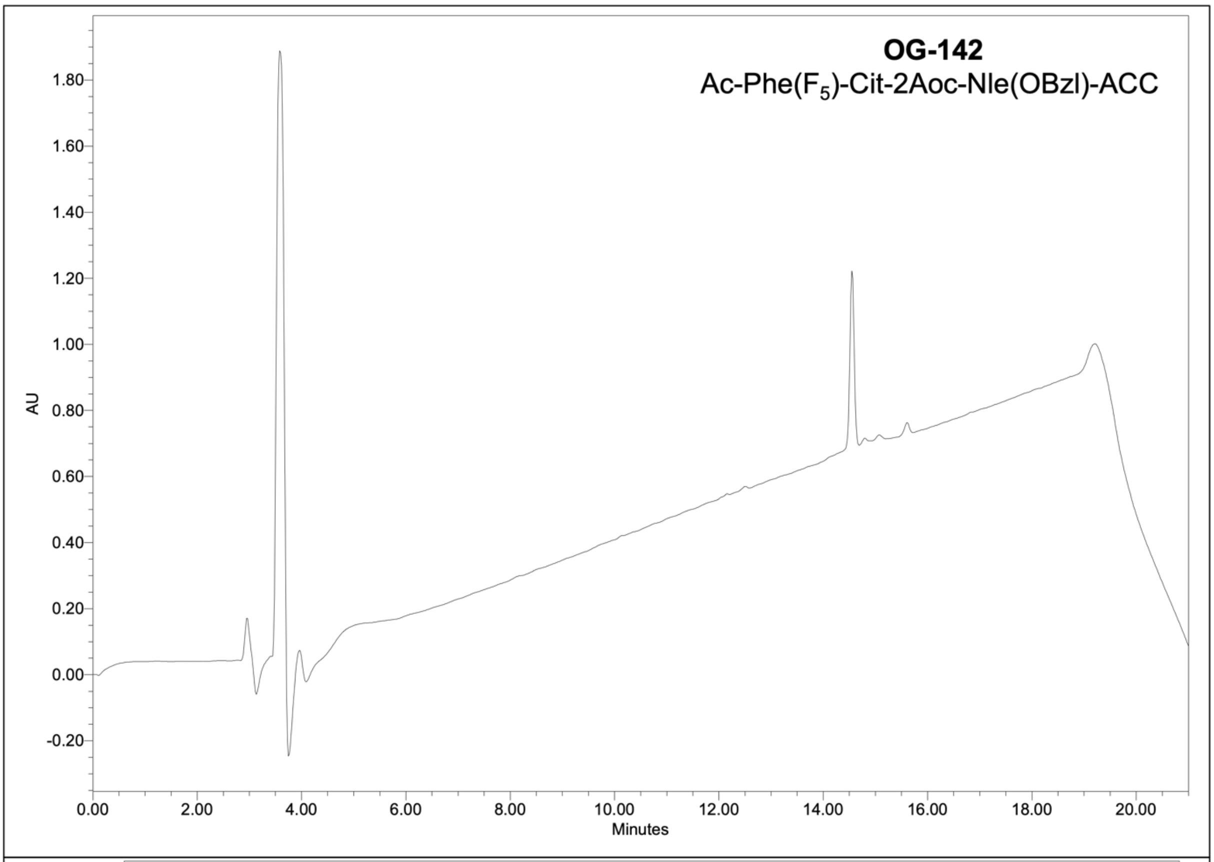
OG-142
Ac-Phe(F$_5$)-Cit-2Aoc-Nle(OBzl)-ACC
AU
Minutes

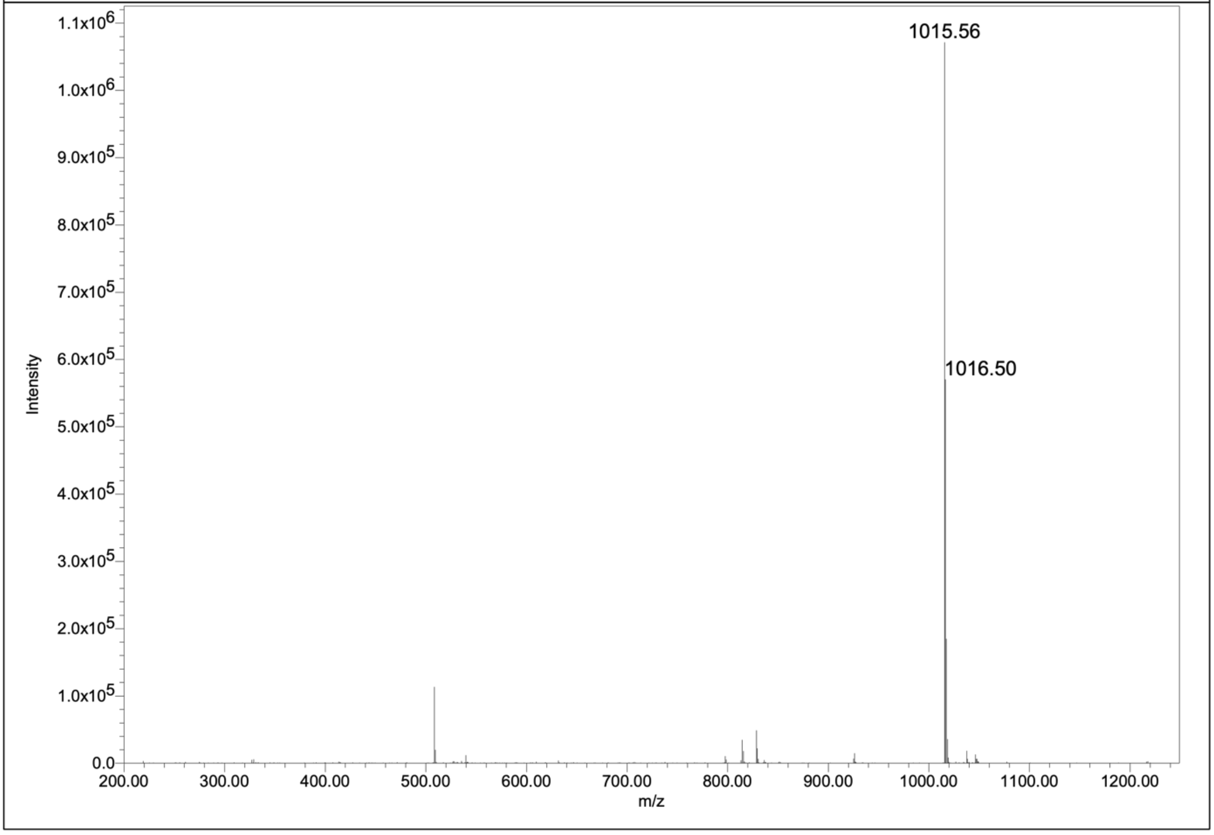
1015.56
1016.50
Intensity
m/z

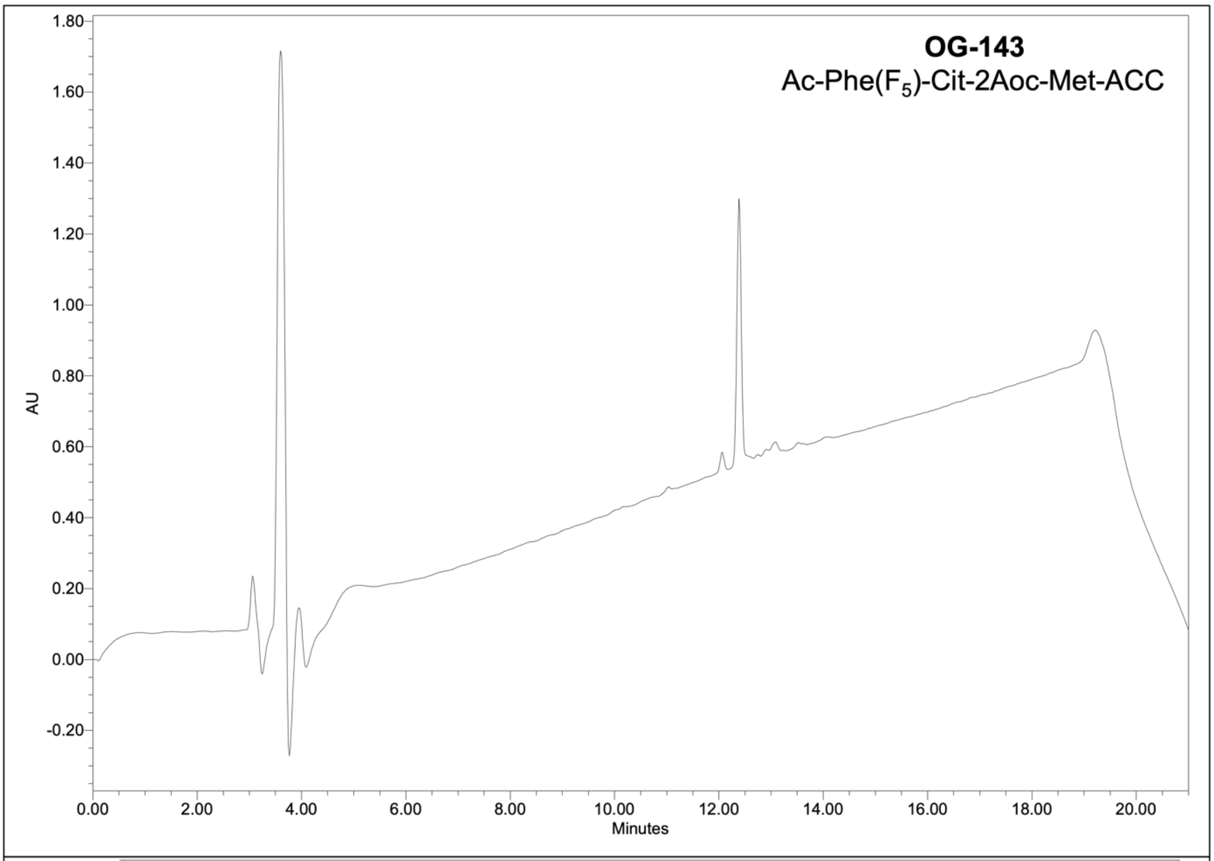

OG-143
Ac-Phe(F5)-Cit-2Aoc-Met-ACC
AU
Minutes

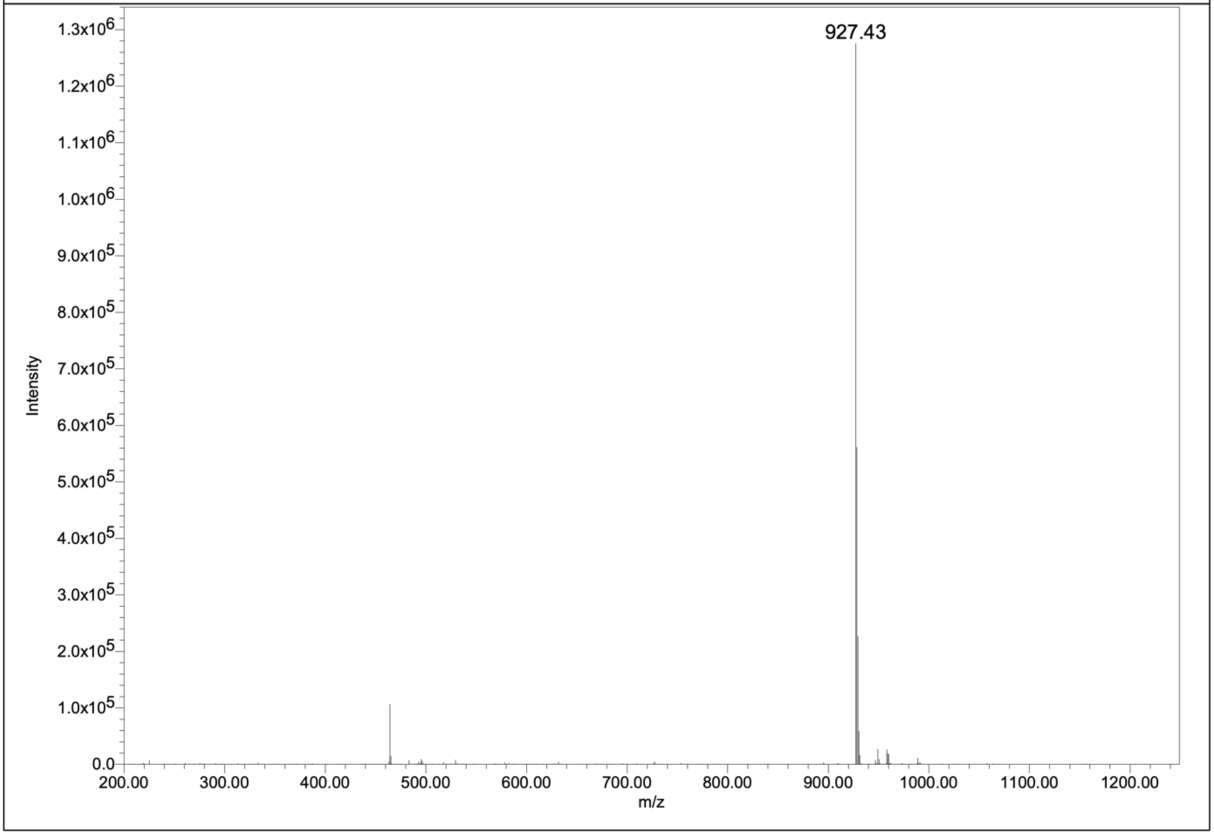

927.43
Intensity
m/z

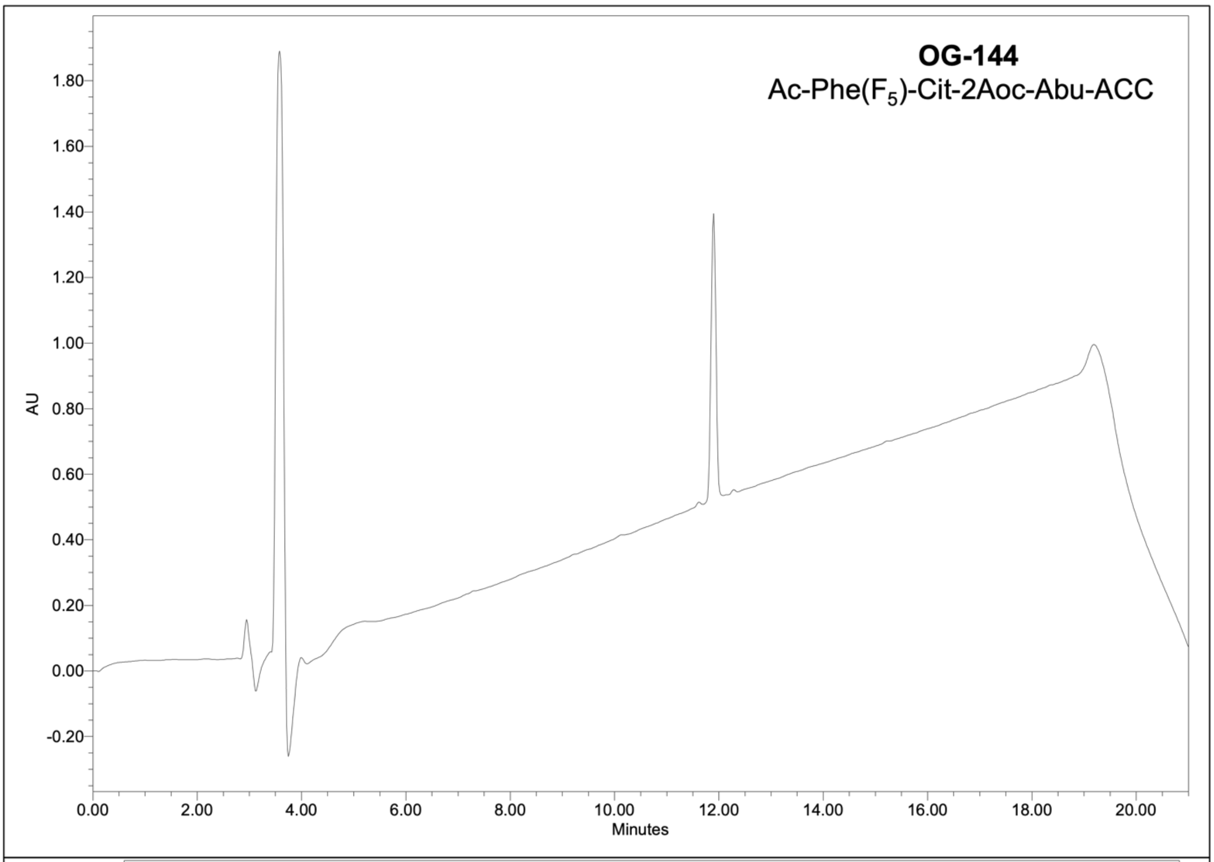
OG-144
Ac-Phe(F5)-Cit-2Aoc-Abu-ACC
AU
Minutes

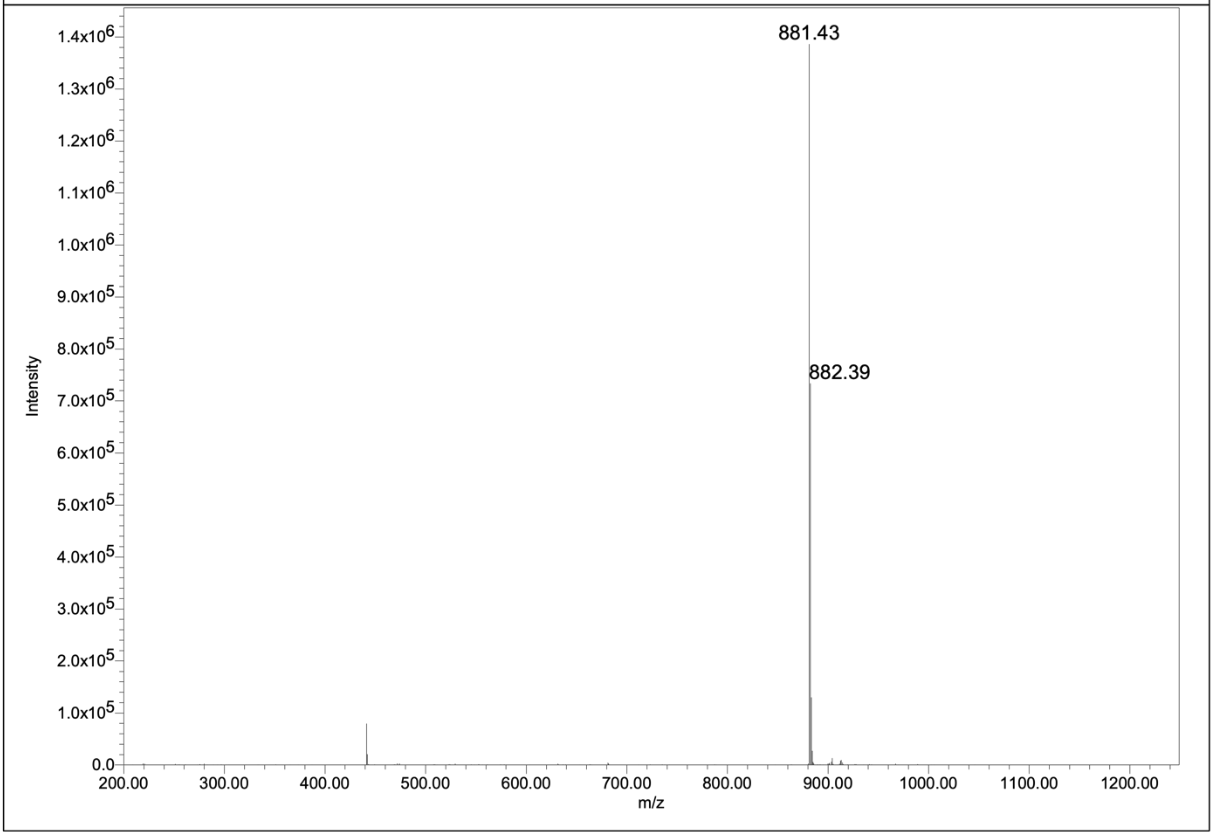
881.43
882.39
Intensity
m/z

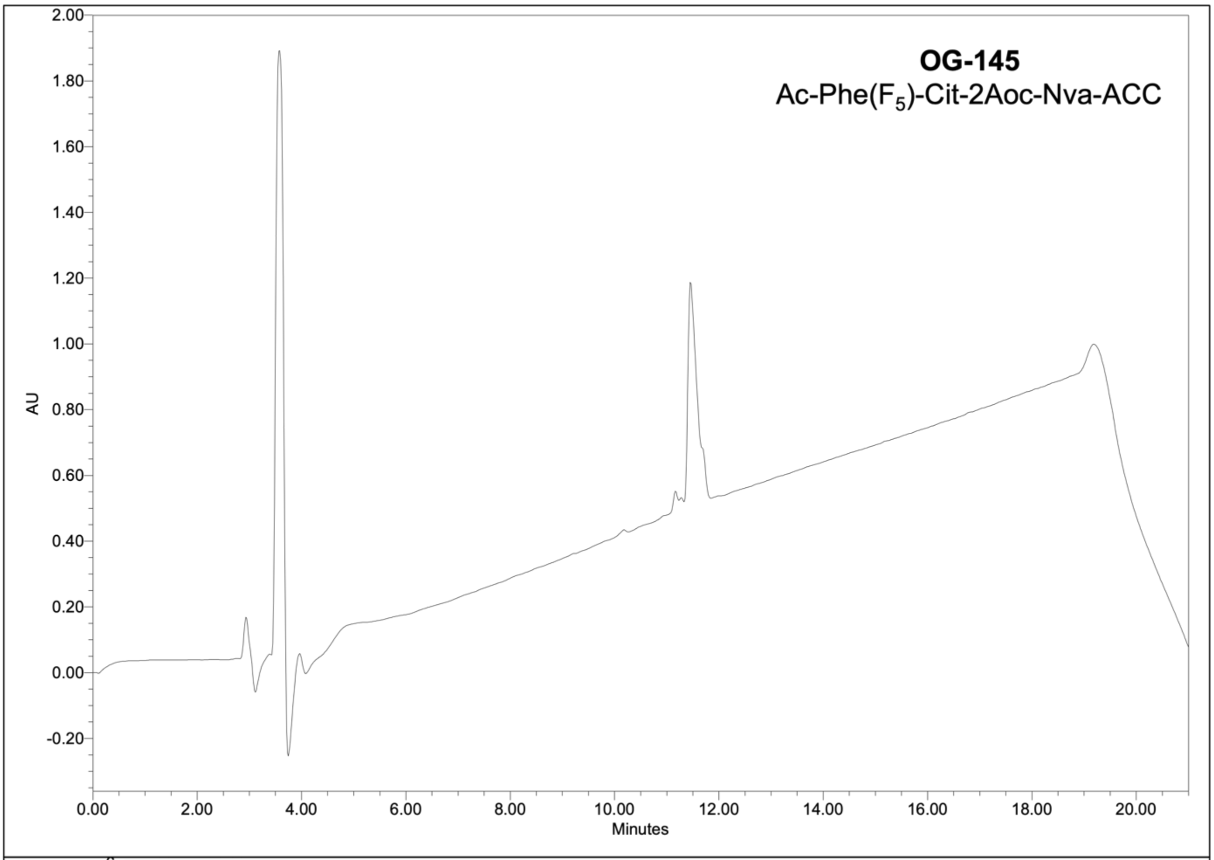

OG-145
Ac-Phe(F5)-Cit-2Aoc-Nva-ACC
AU
Minutes

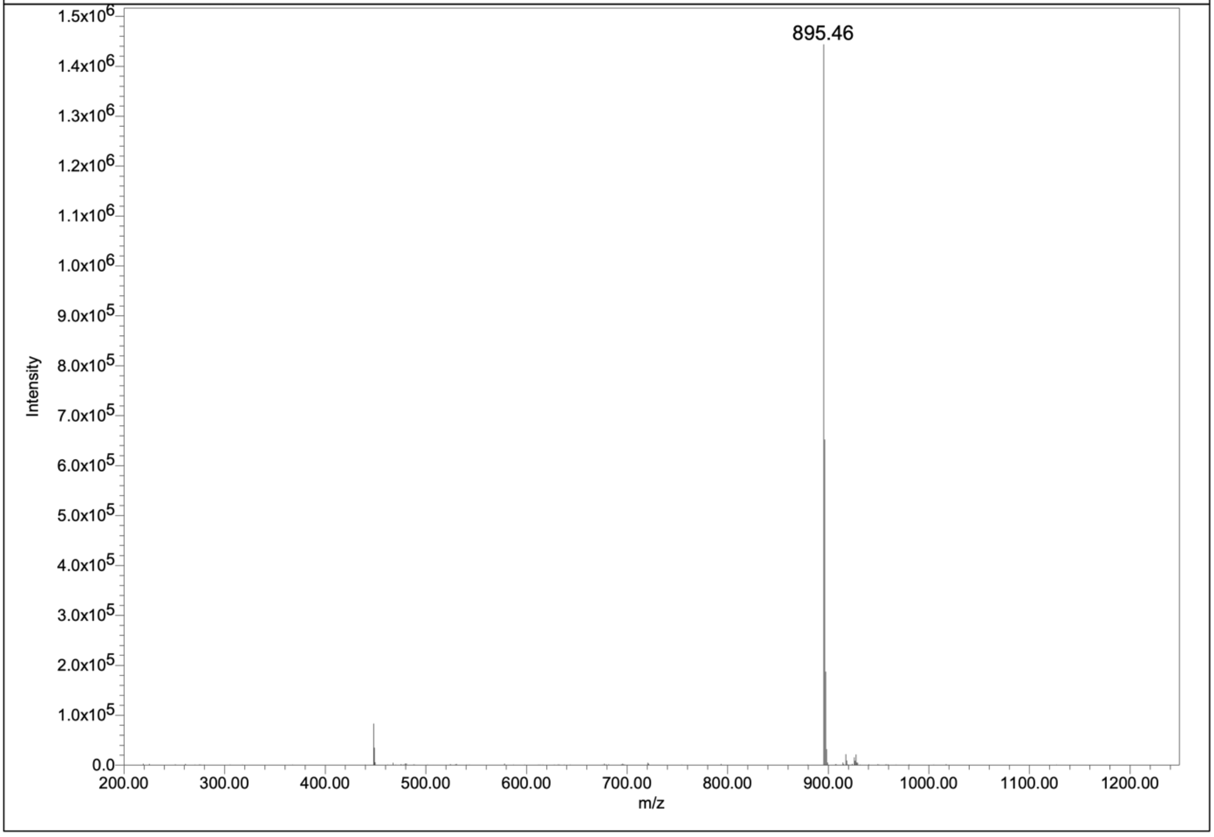

895.46
Intensity
m/z

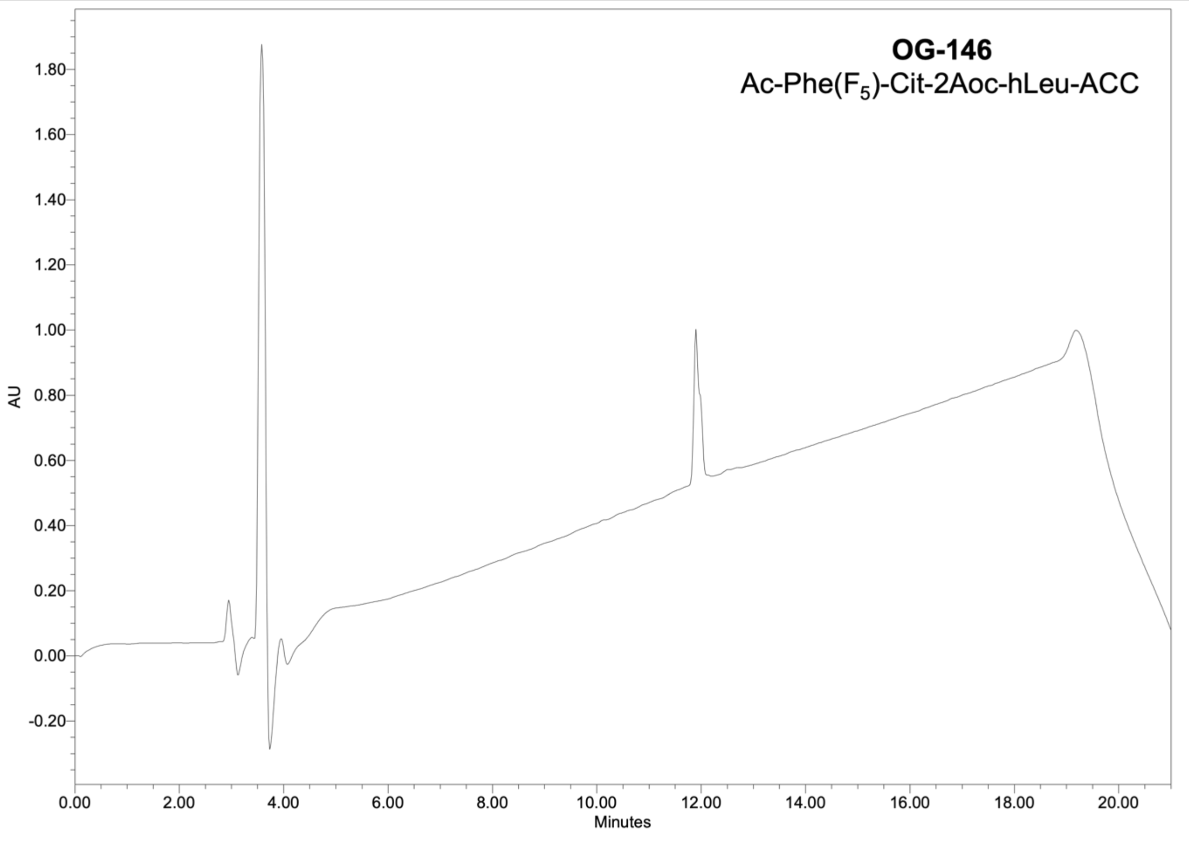
OG-146
Ac-Phe(F5)-Cit-2Aoc-hLeu-ACC
AU
Minutes

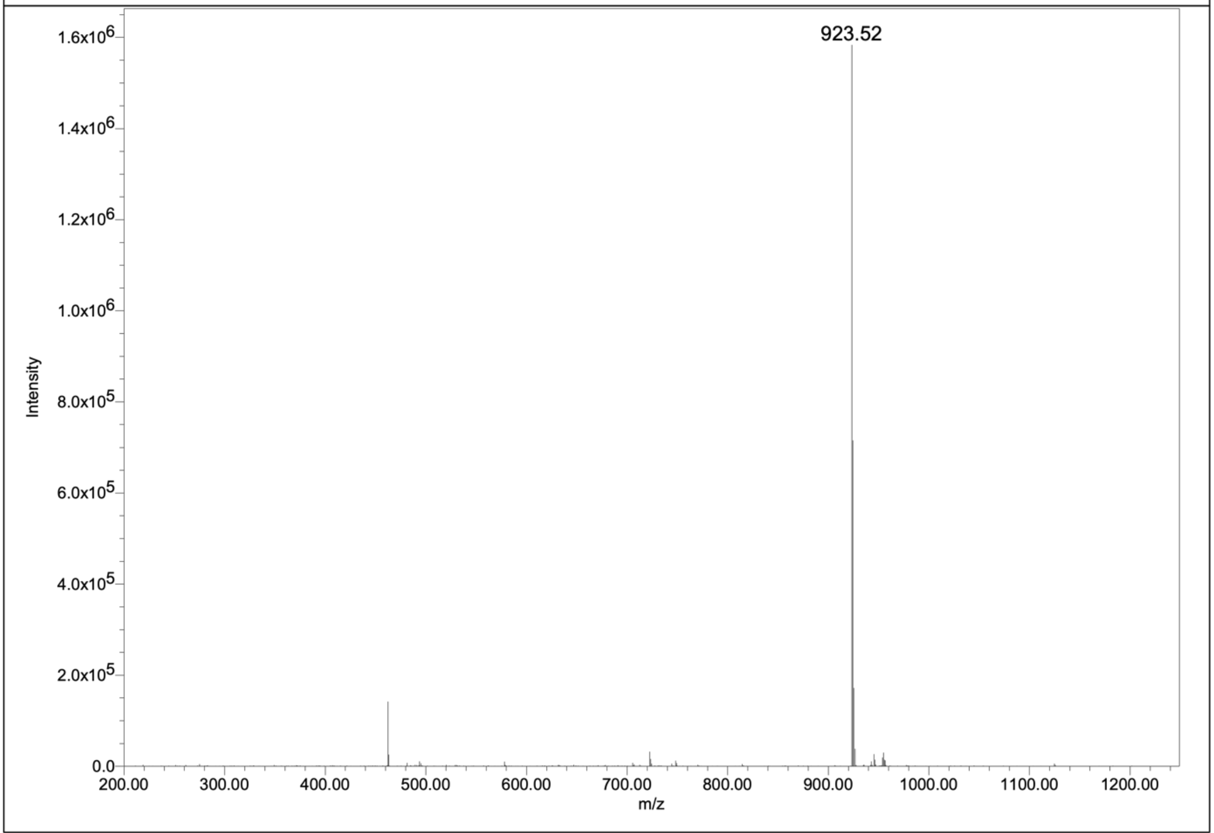
923.52
Intensity
m/z

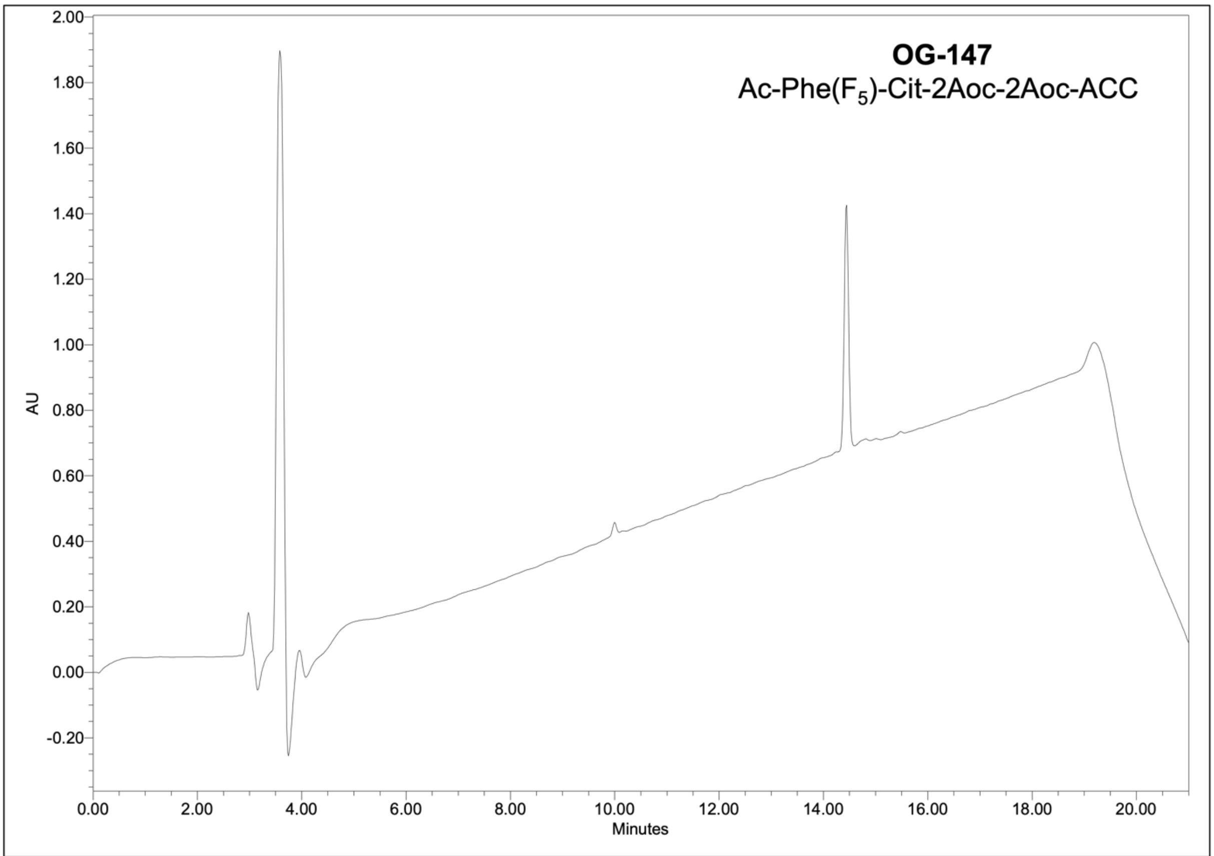
OG-147
Ac-Phe(F5)-Cit-2Aoc-2Aoc-ACC
AU
Minutes
2.00
1.80
1.60
1.40
1.20
1.00
0.80
0.60
0.40
0.20
0.00
-0.20
0.00 2.00 4.00 6.00 8.00 10.00 12.00 14.00 16.00 18.00 20.00

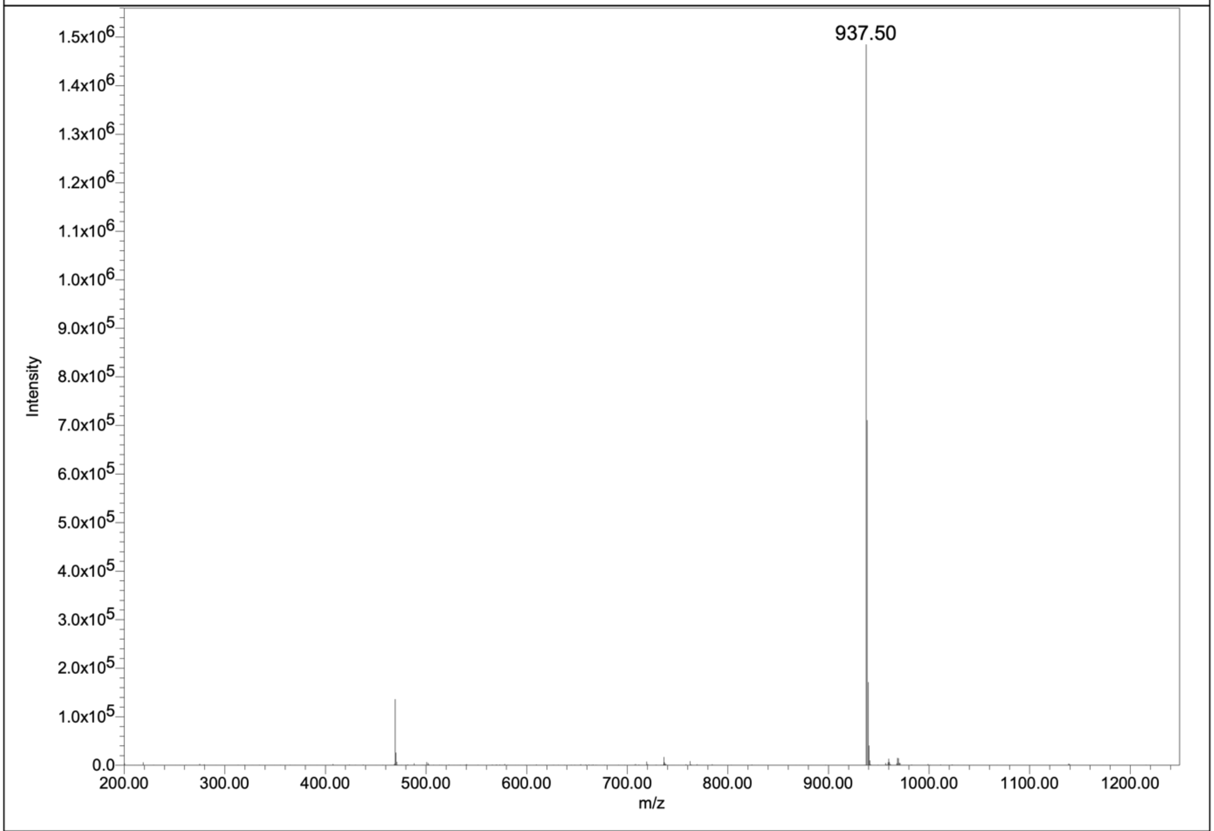
937.50
Intensity
m/z
200.00 300.00 400.00 500.00 600.00 700.00 800.00 900.00 1000.00 1100.00 1200.00

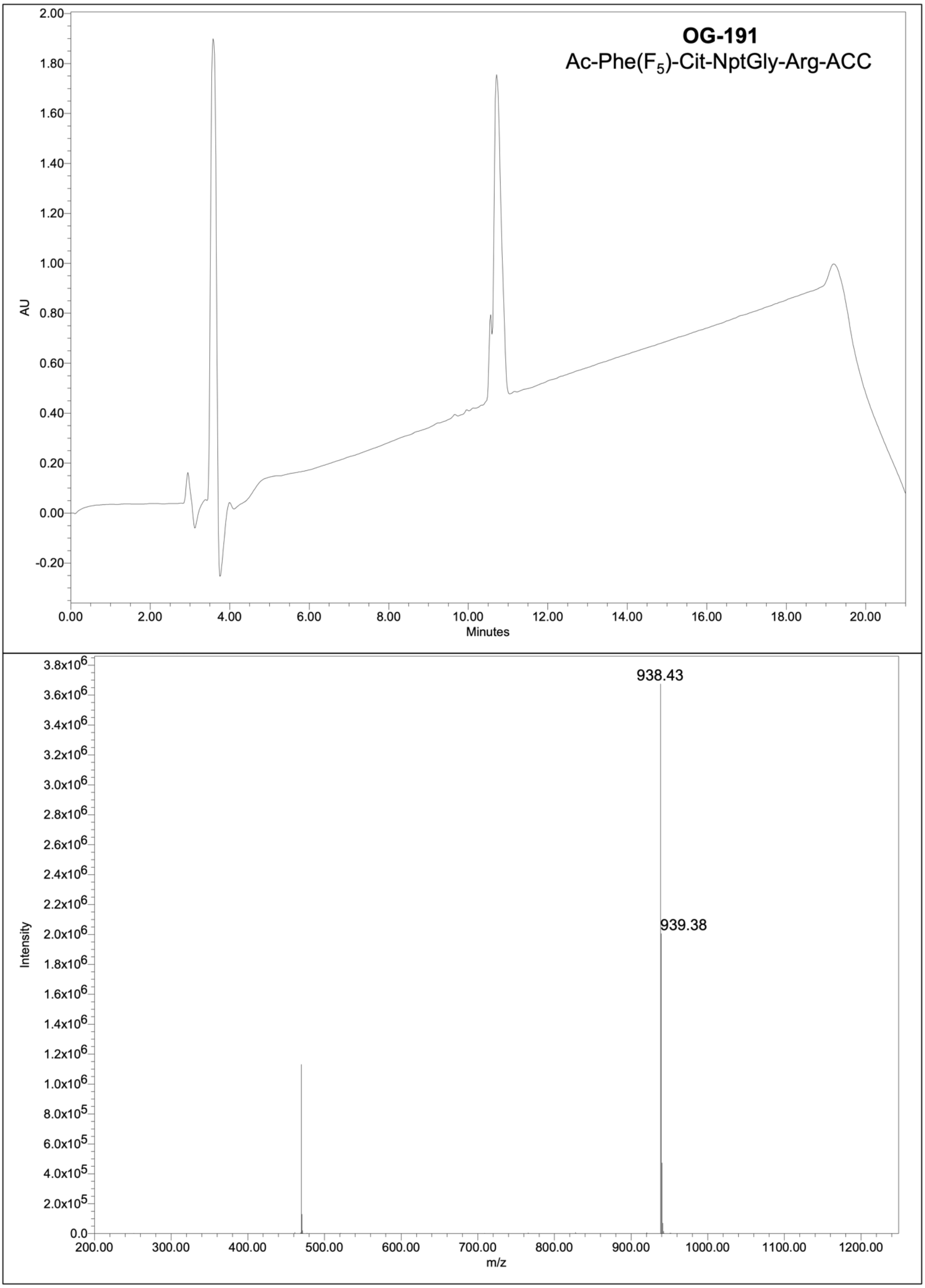
OG-191
Ac-Phe(F5)-Cit-NptGly-Arg-ACC
AU
Minutes
938.43
939.38
Intensity
m/z

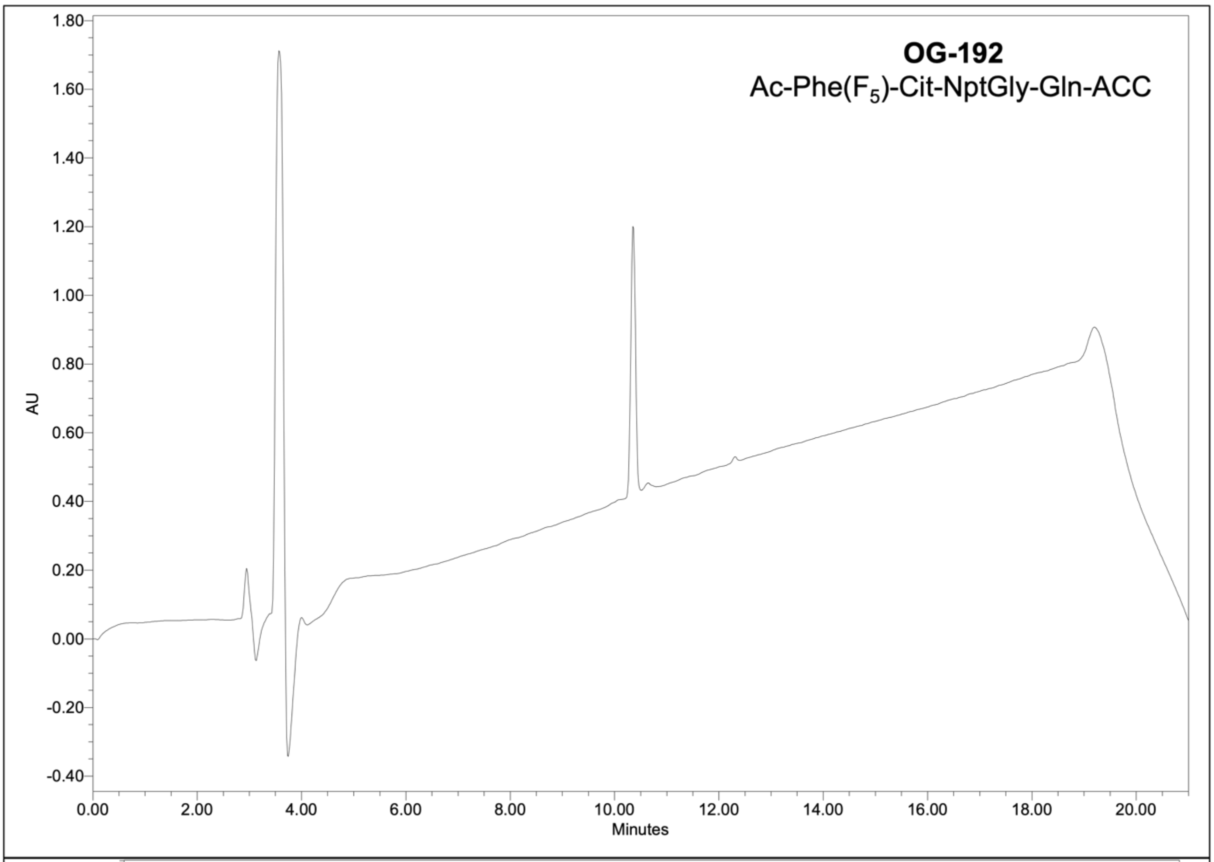
OG-192
Ac-Phe(F$_5$)-Cit-NptGly-Gln-ACC
AU
Minutes

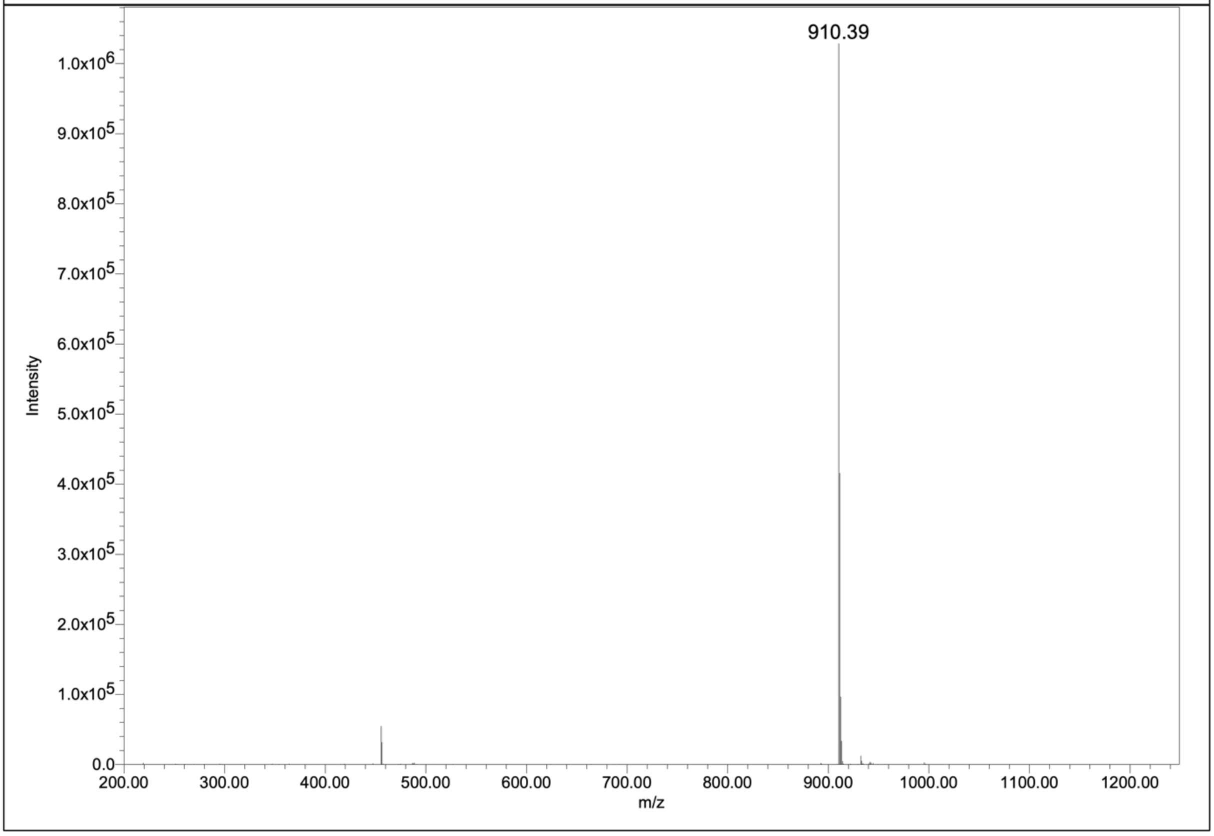
910.39
Intensity
m/z

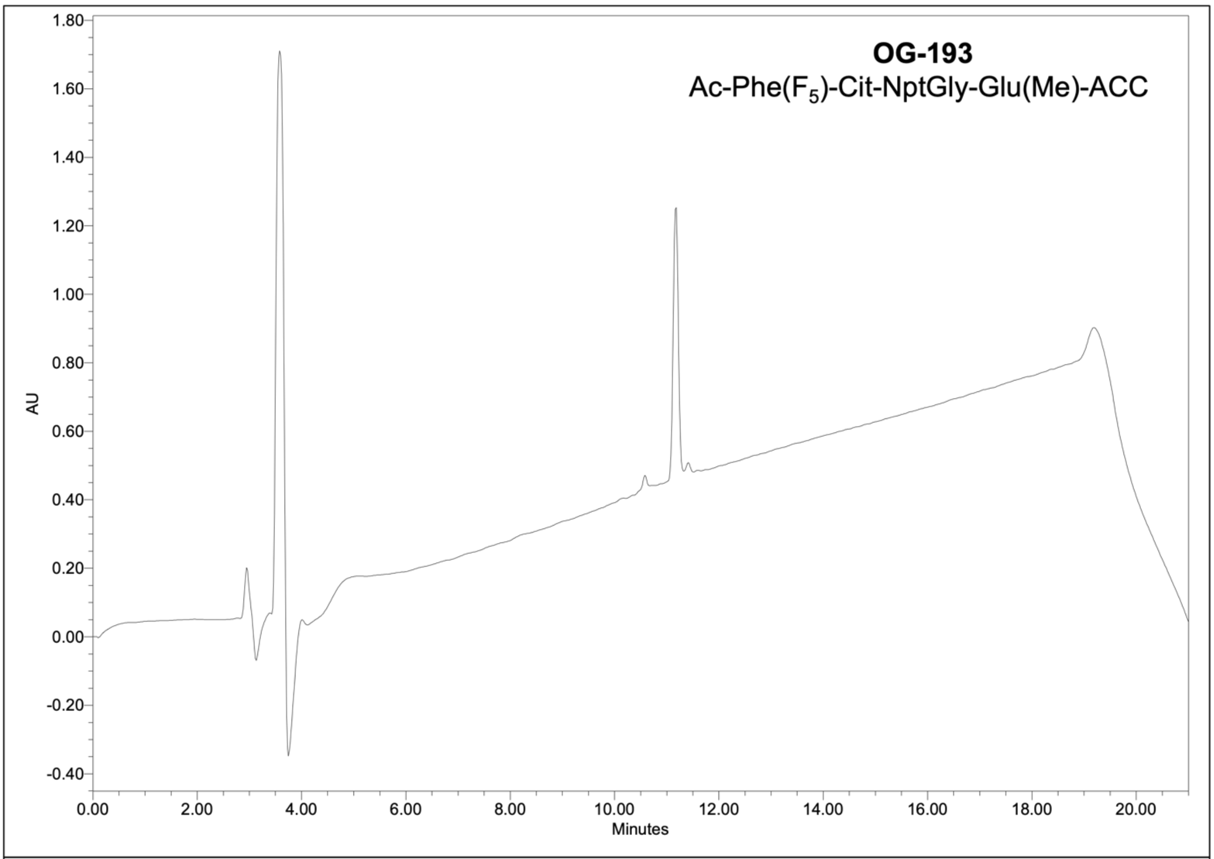
OG-193
Ac-Phe(F5)-Cit-NptGly-Glu(Me)-ACC
AU
Minutes

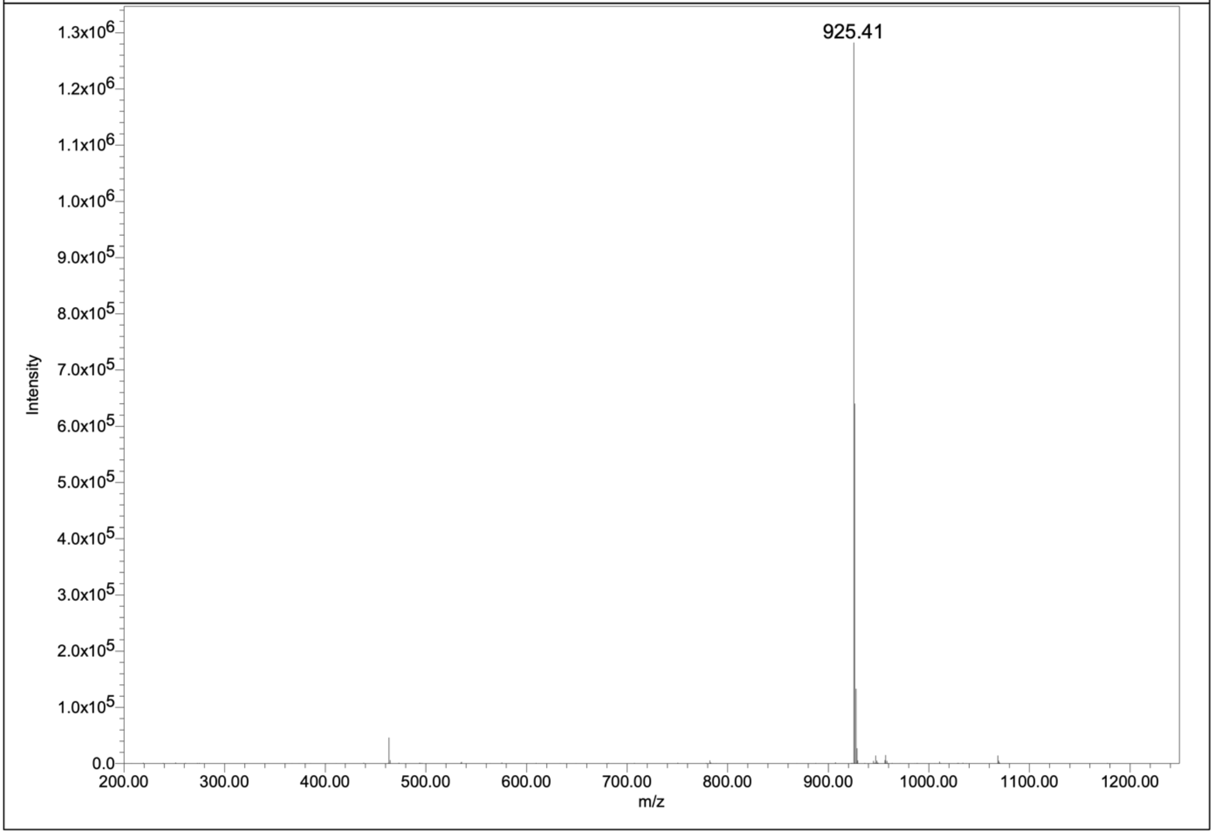
925.41
Intensity
m/z

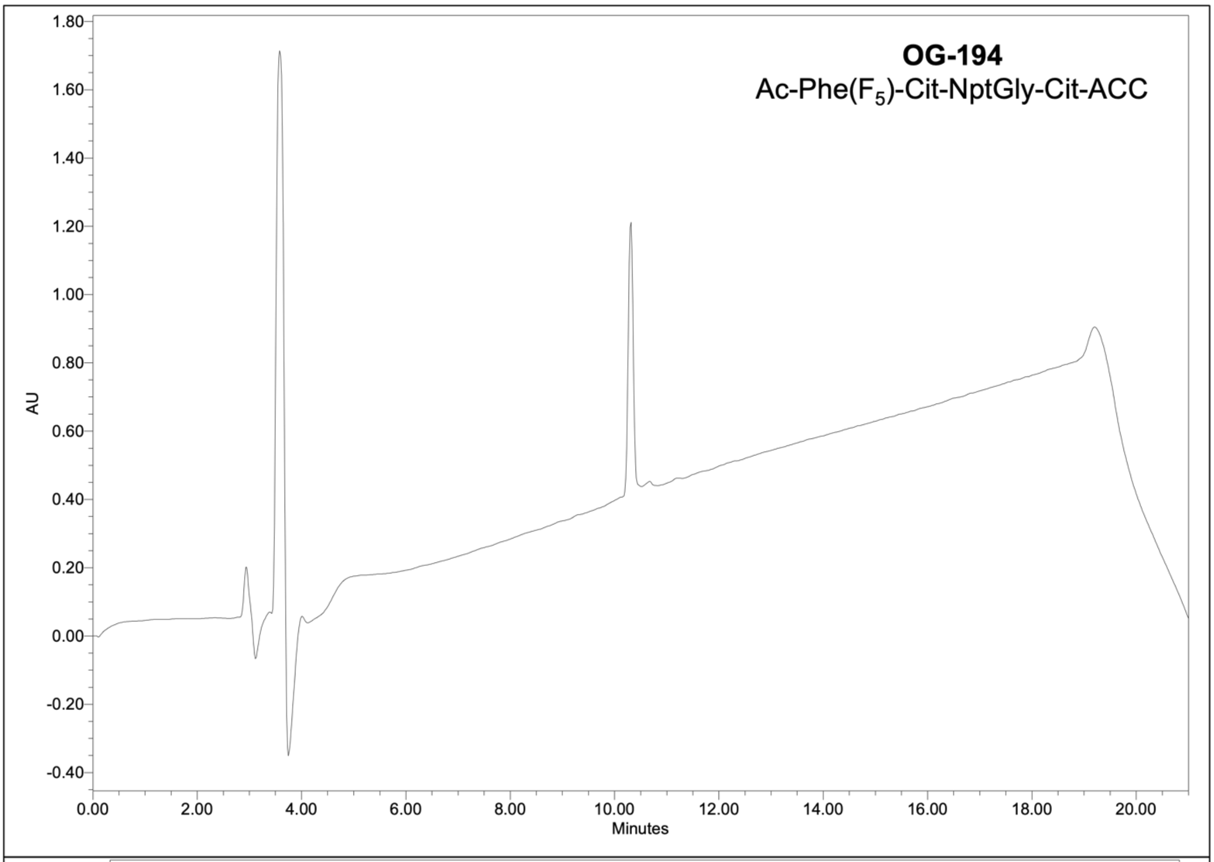
OG-194
Ac-Phe(F5)-Cit-NptGly-Cit-ACC
AU
Minutes

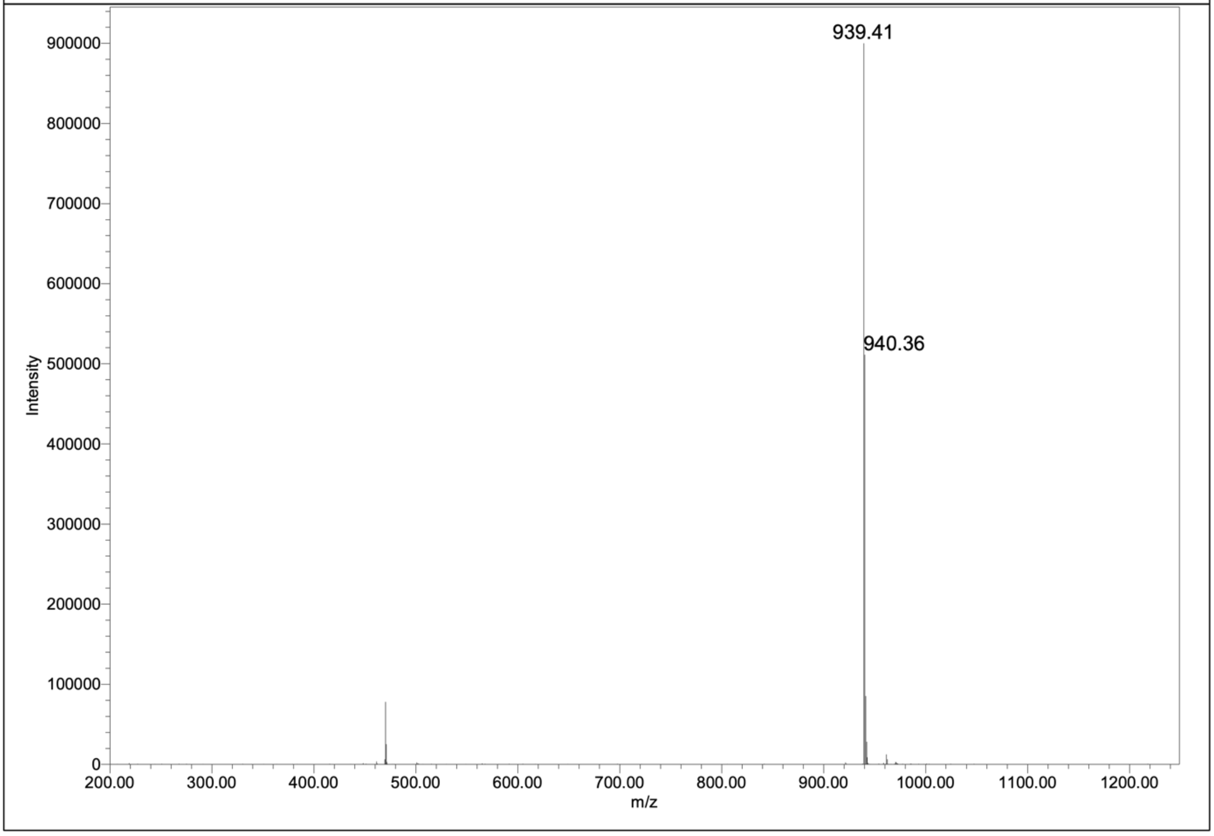
939.41
940.36
Intensity
m/z

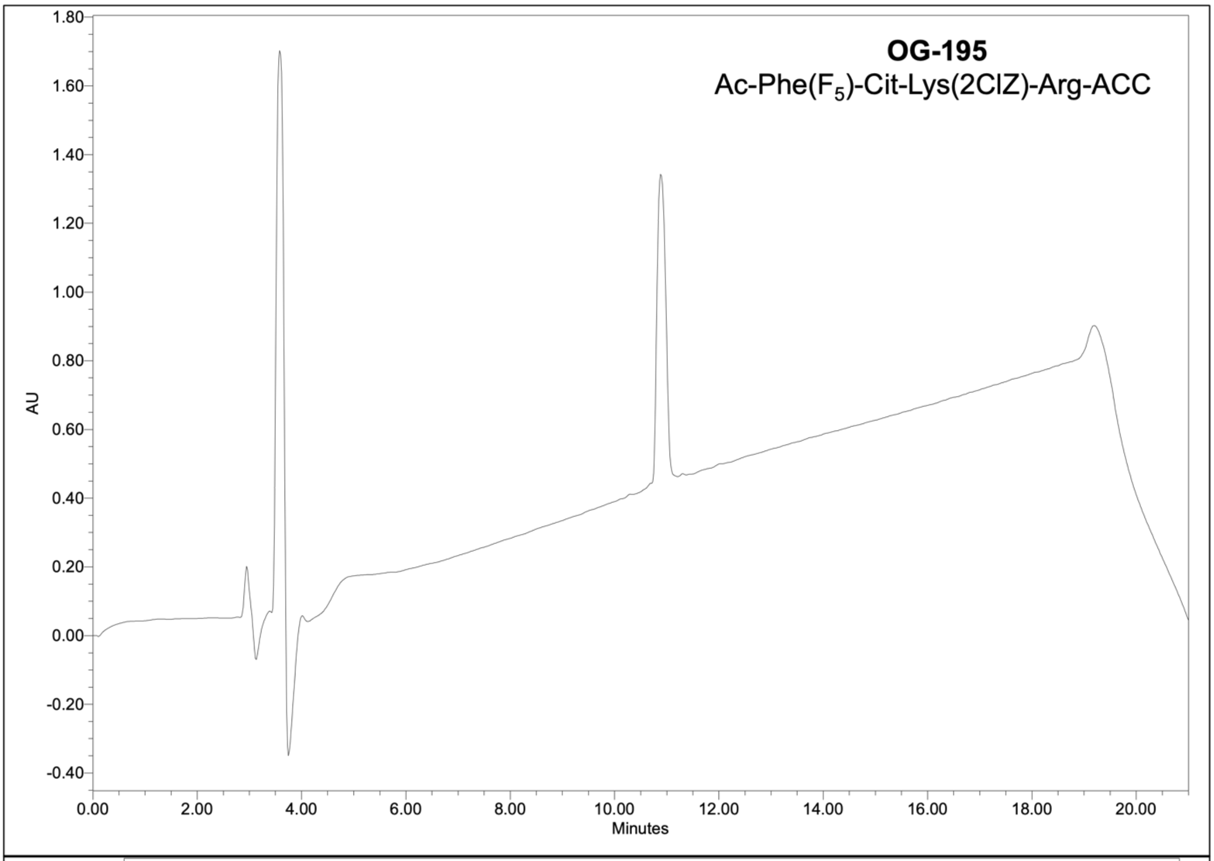
OG-195
Ac-Phe(F5)-Cit-Lys(2ClZ)-Arg-ACC
AU
Minutes

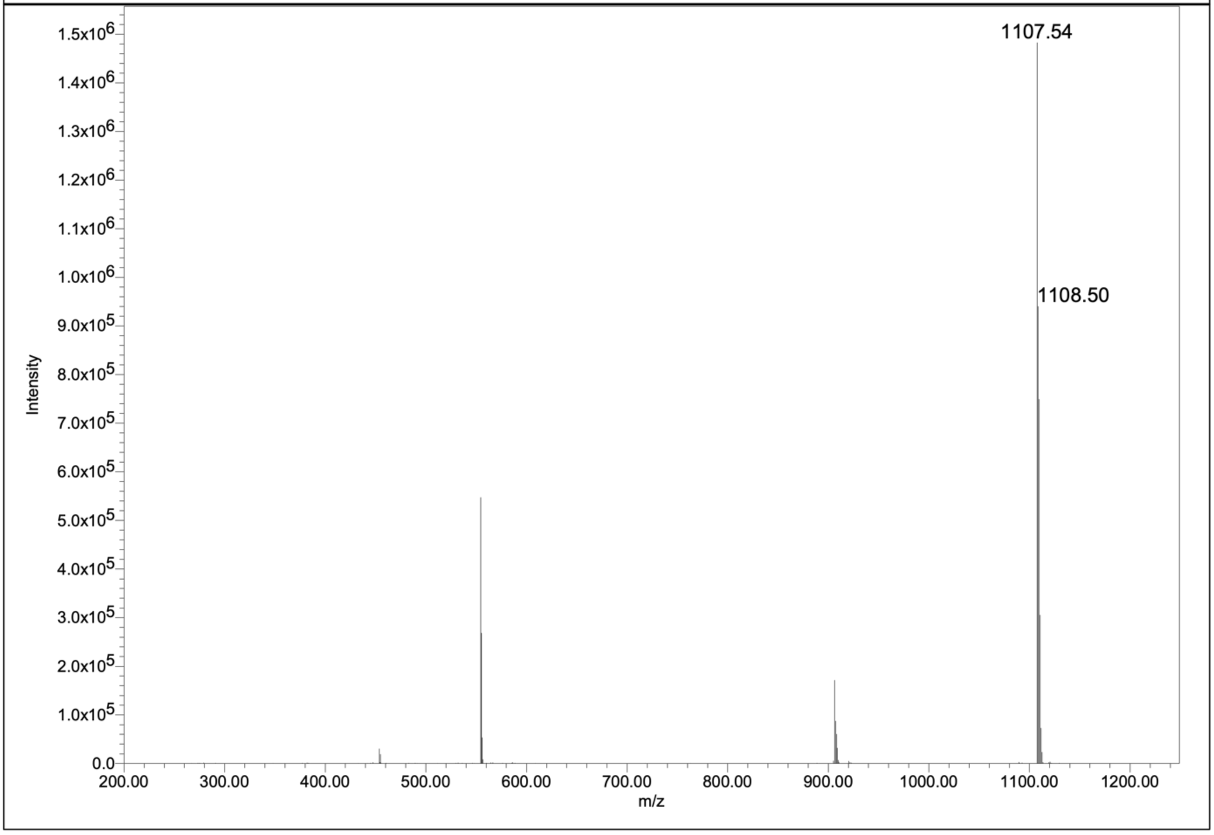
1107.54
1108.50
Intensity
m/z

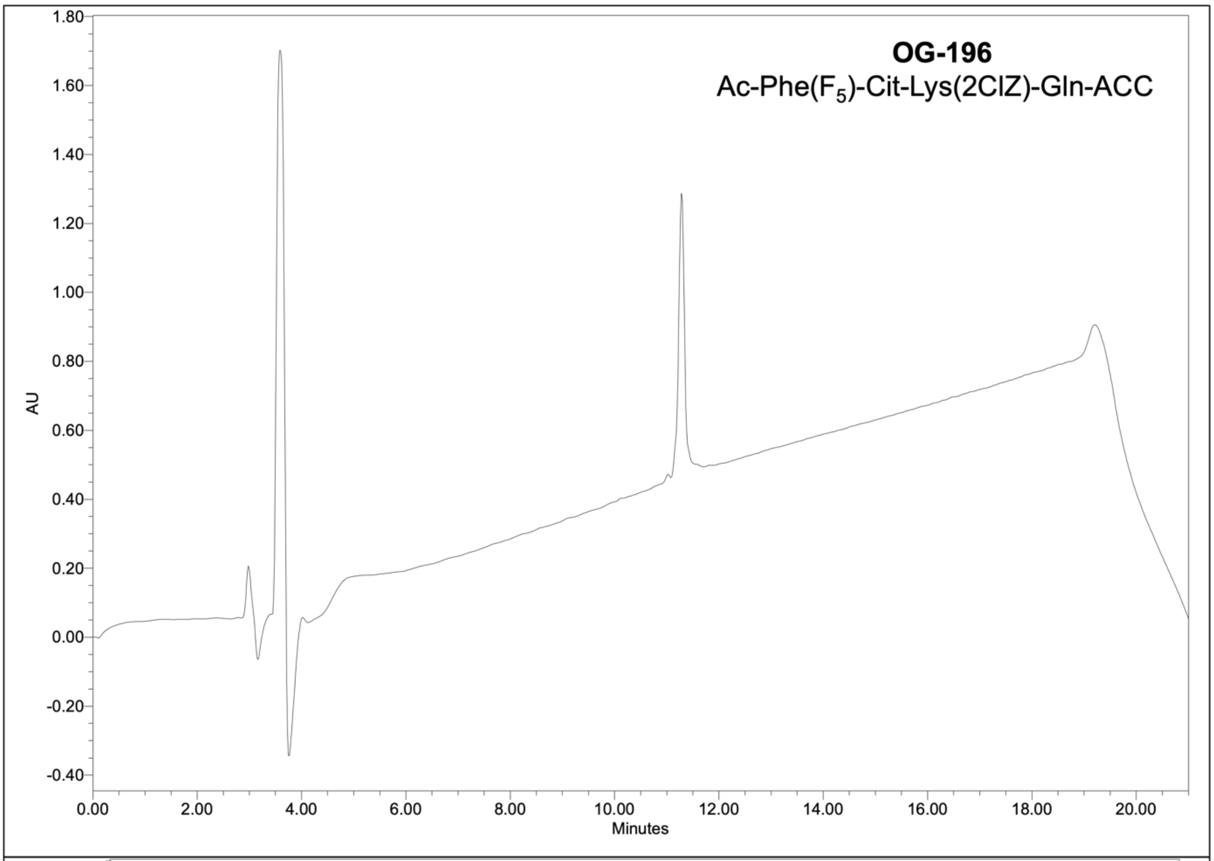
OG-196
Ac-Phe(F5)-Cit-Lys(2ClZ)-Gln-ACC
AU
Minutes

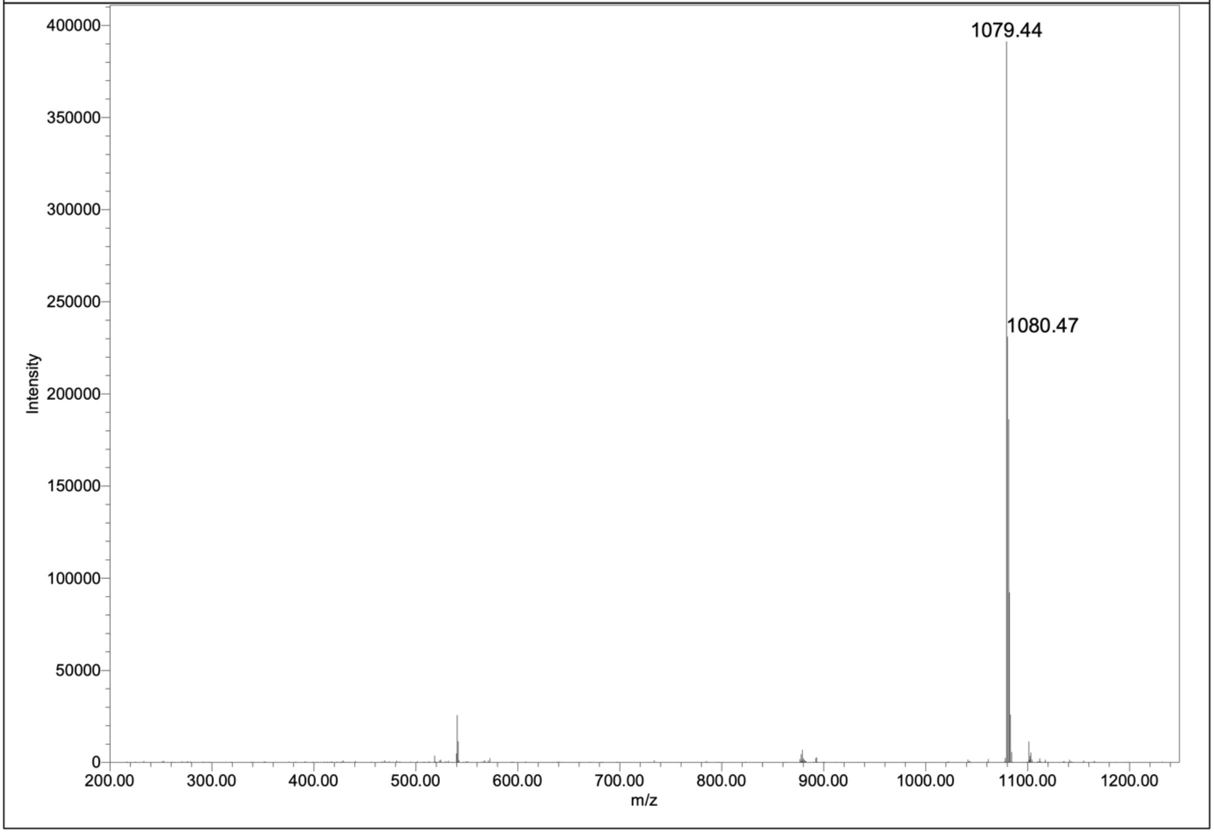
1079.44
1080.47
Intensity
m/z

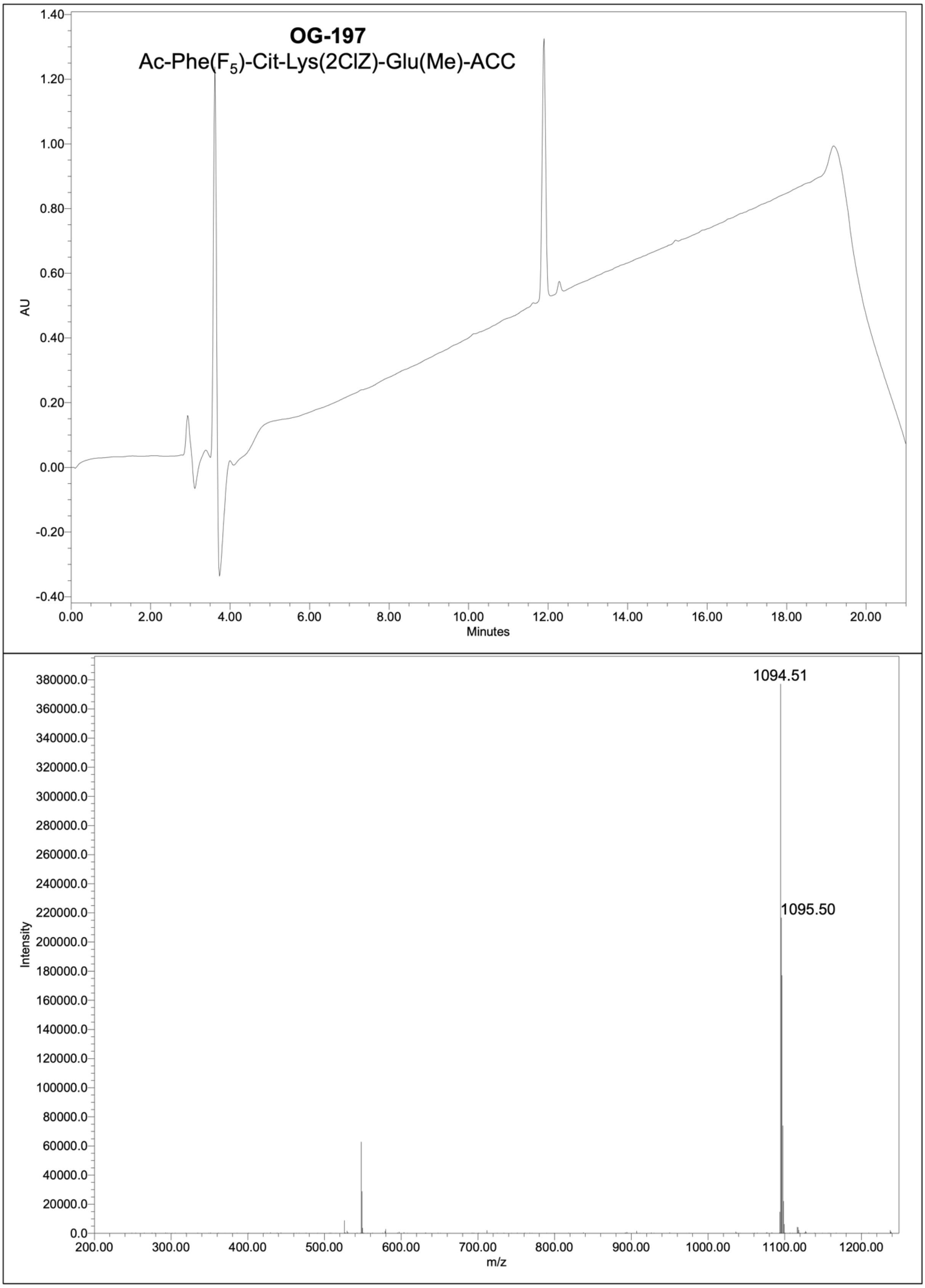
OG-197
Ac-Phe(F5)-Cit-Lys(2ClZ)-Glu(Me)-ACC
AU
Minutes
1094.51
1095.50
Intensity
m/z

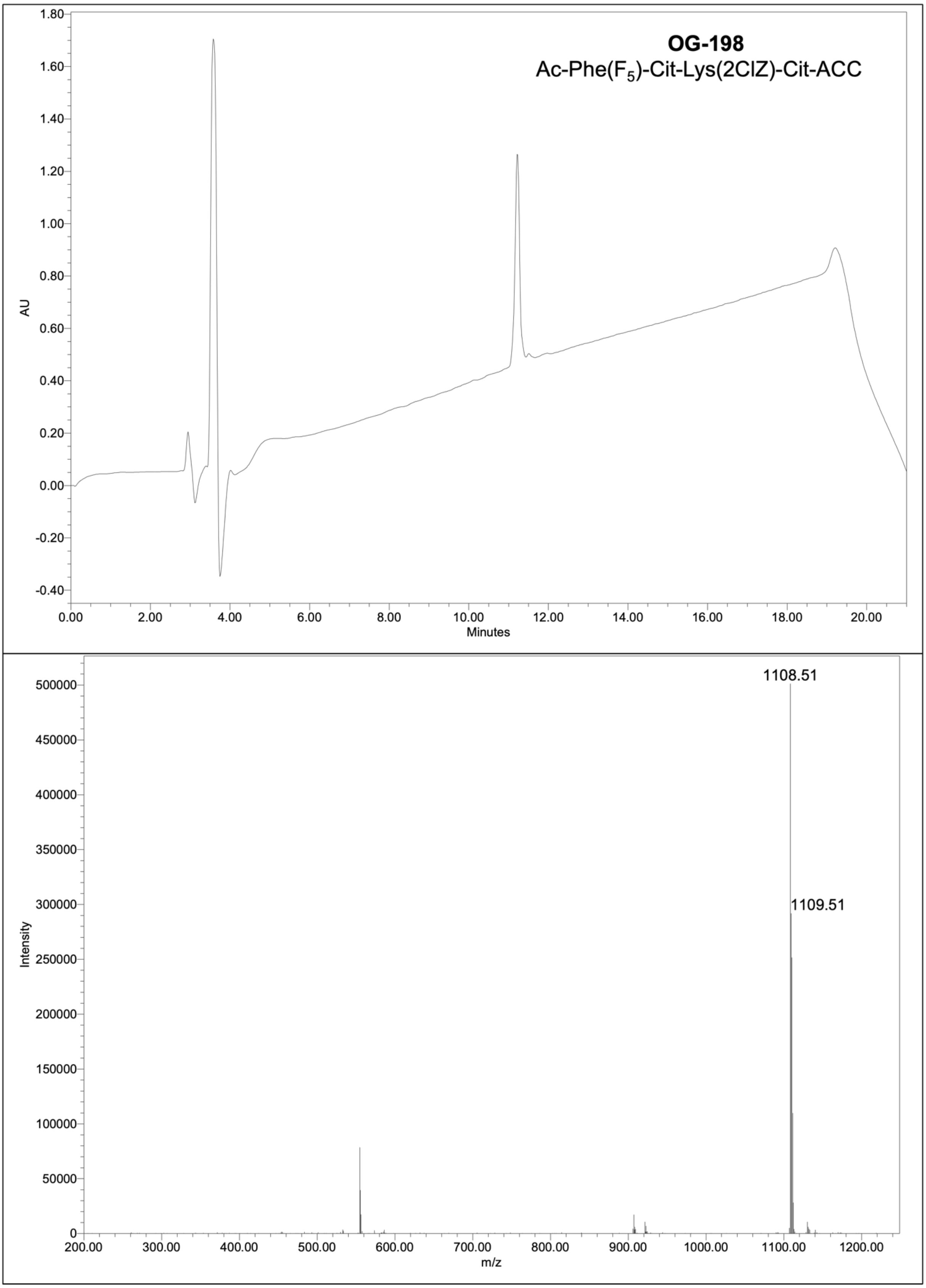
OG-198
Ac-Phe(F5)-Cit-Lys(2ClZ)-Cit-ACC
AU
Minutes
1108.51
1109.51
Intensity
m/z

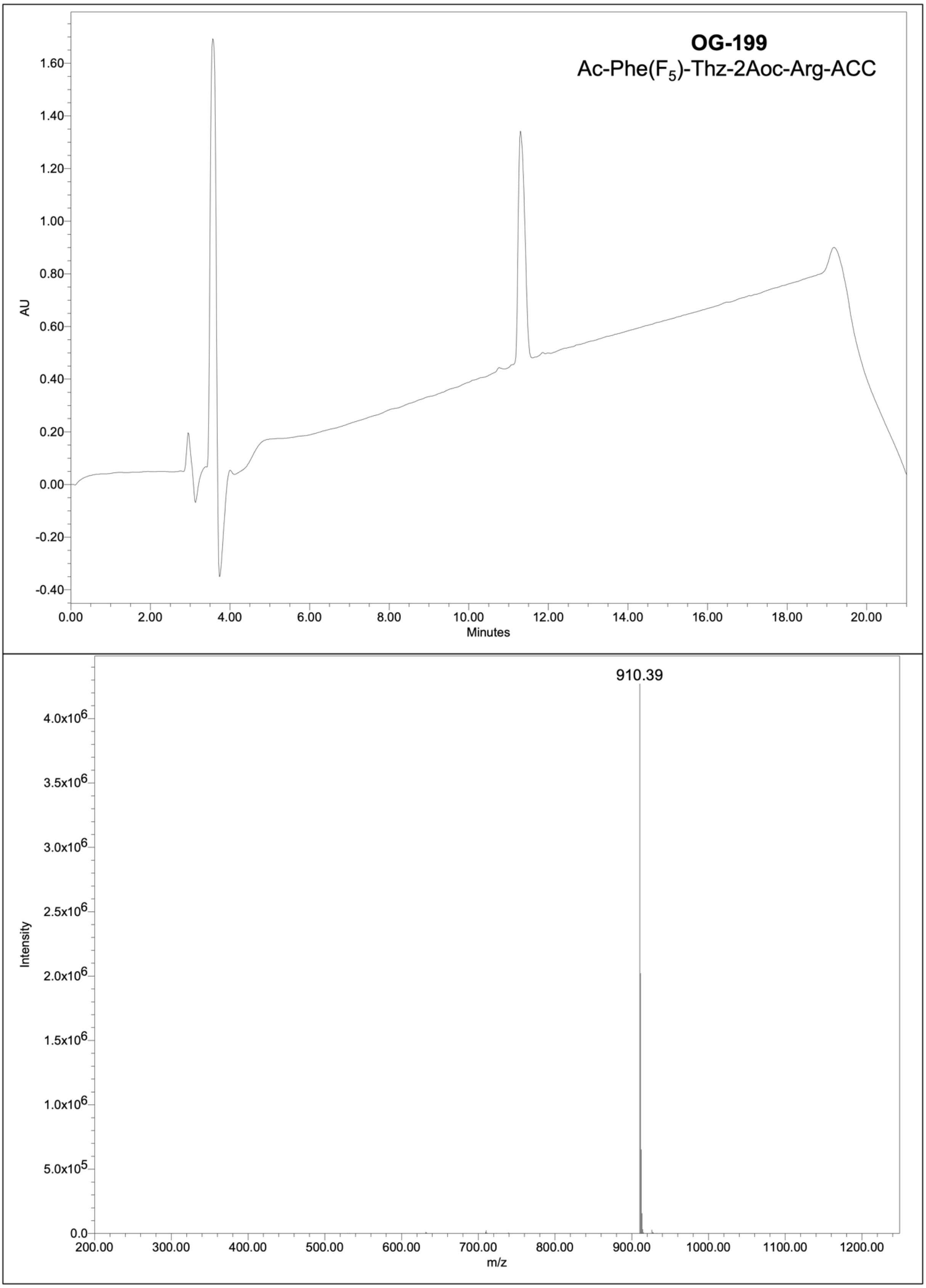

OG-199
Ac-Phe(F5)-Thz-2Aoc-Arg-ACC
AU
Minutes
910.39
Intensity
m/z

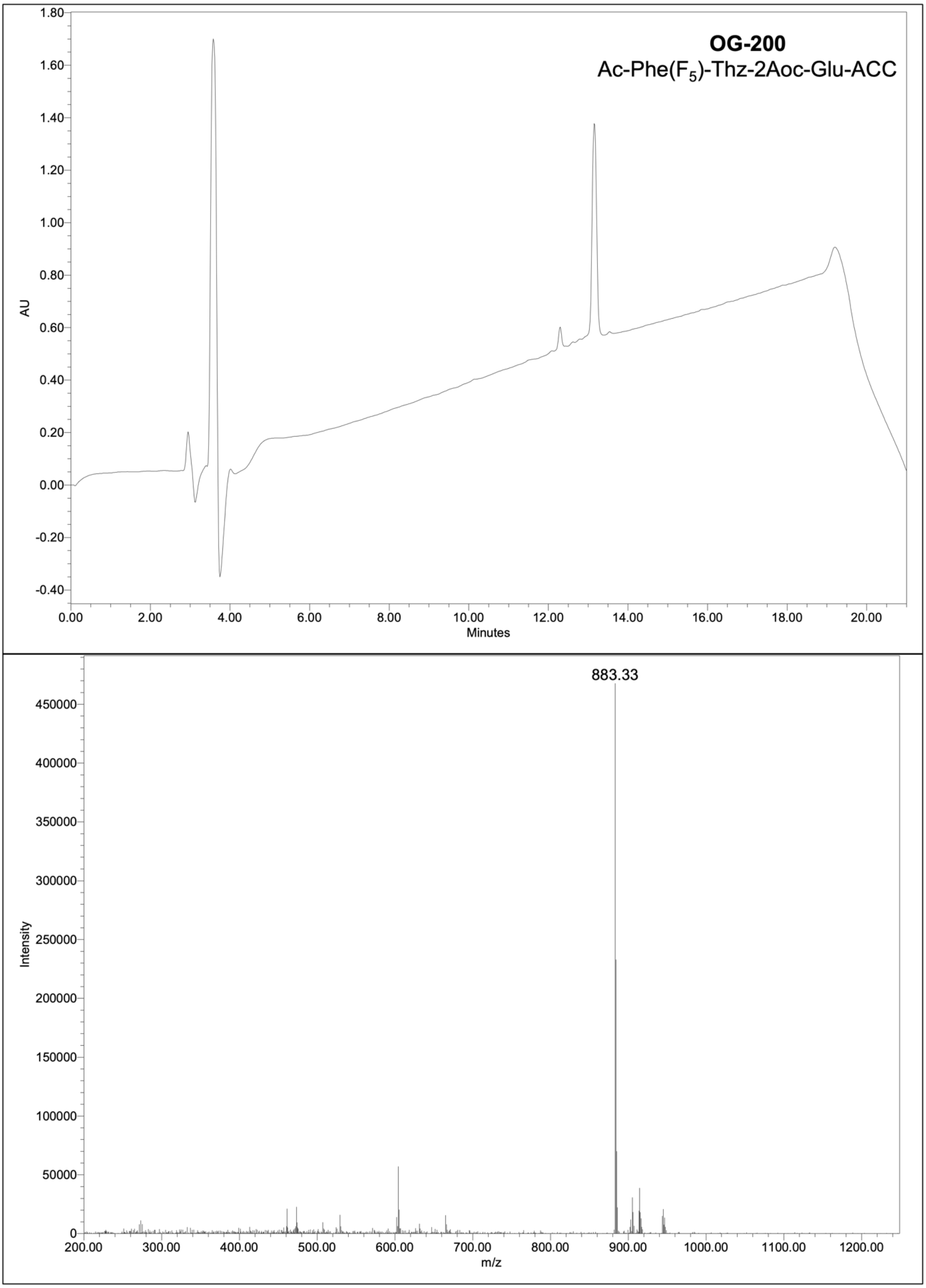
OG-200
Ac-Phe(F5)-Thz-2Aoc-Glu-ACC
AU
Minutes
883.33
Intensity
m/z

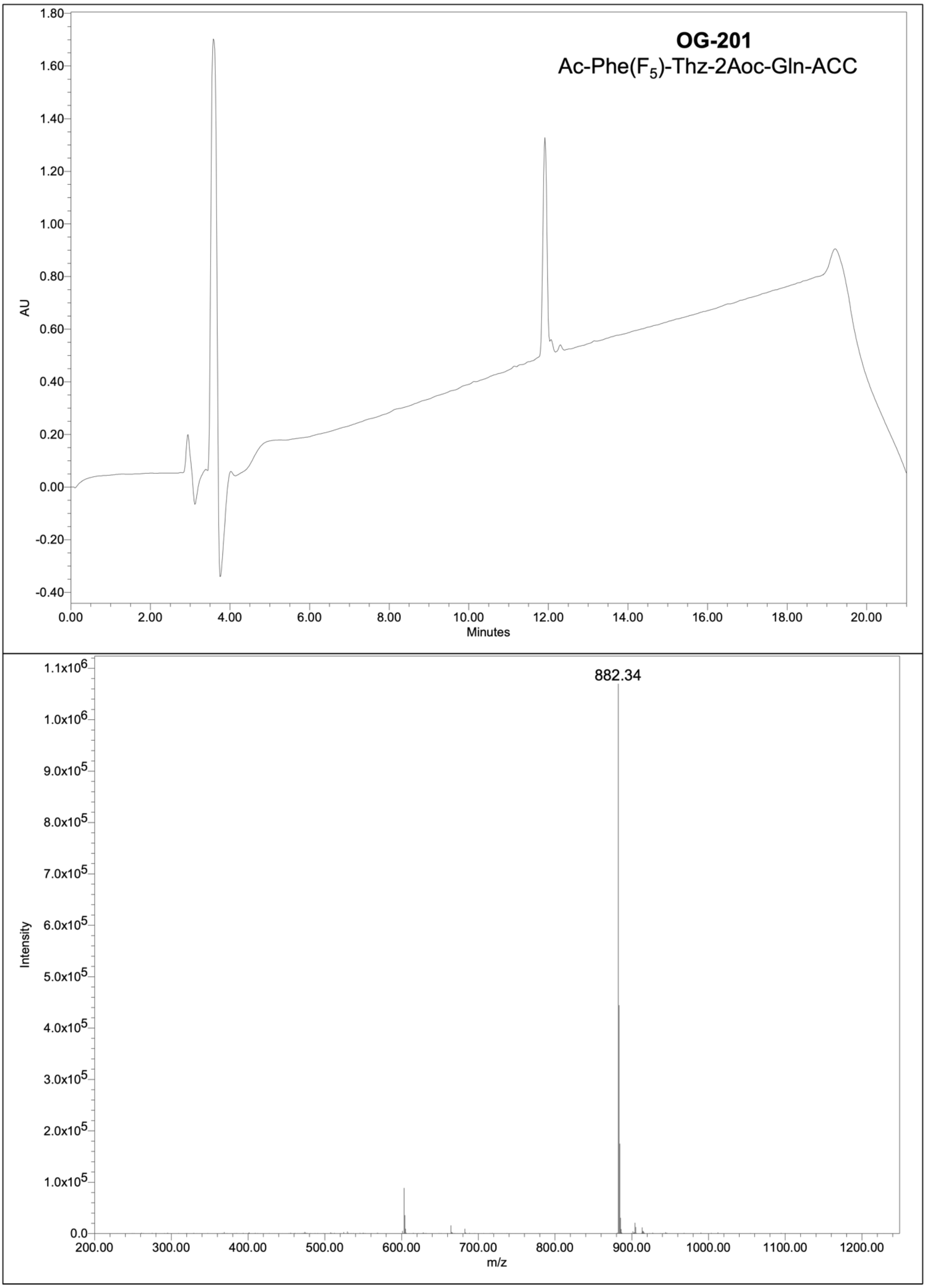
OG-201
Ac-Phe(F$_5$)-Thz-2Aoc-Gln-ACC
AU
Minutes
882.34
Intensity
m/z

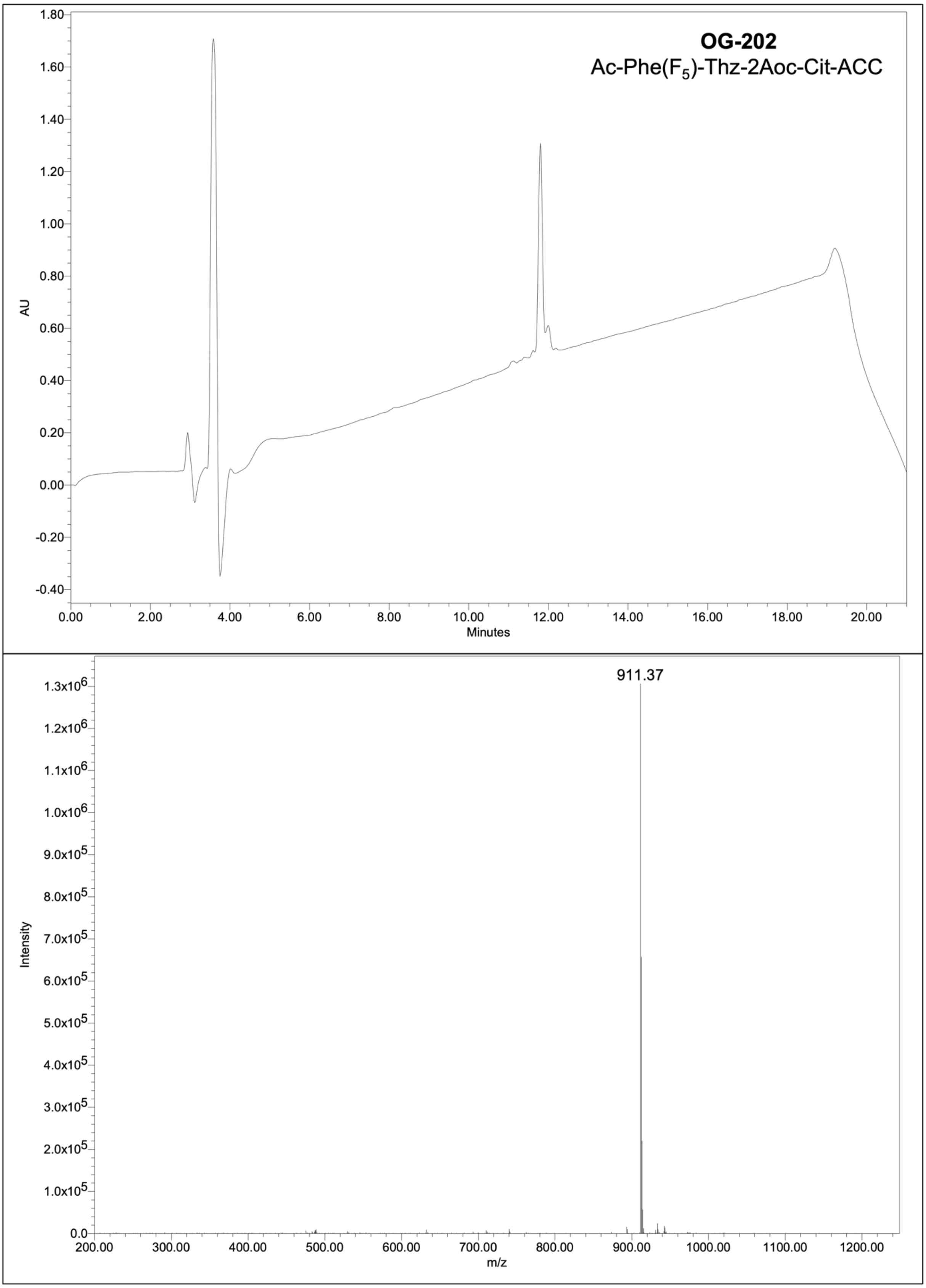
OG-202
Ac-Phe(F5)-Thz-2Aoc-Cit-ACC
AU
Minutes
911.37
Intensity
m/z

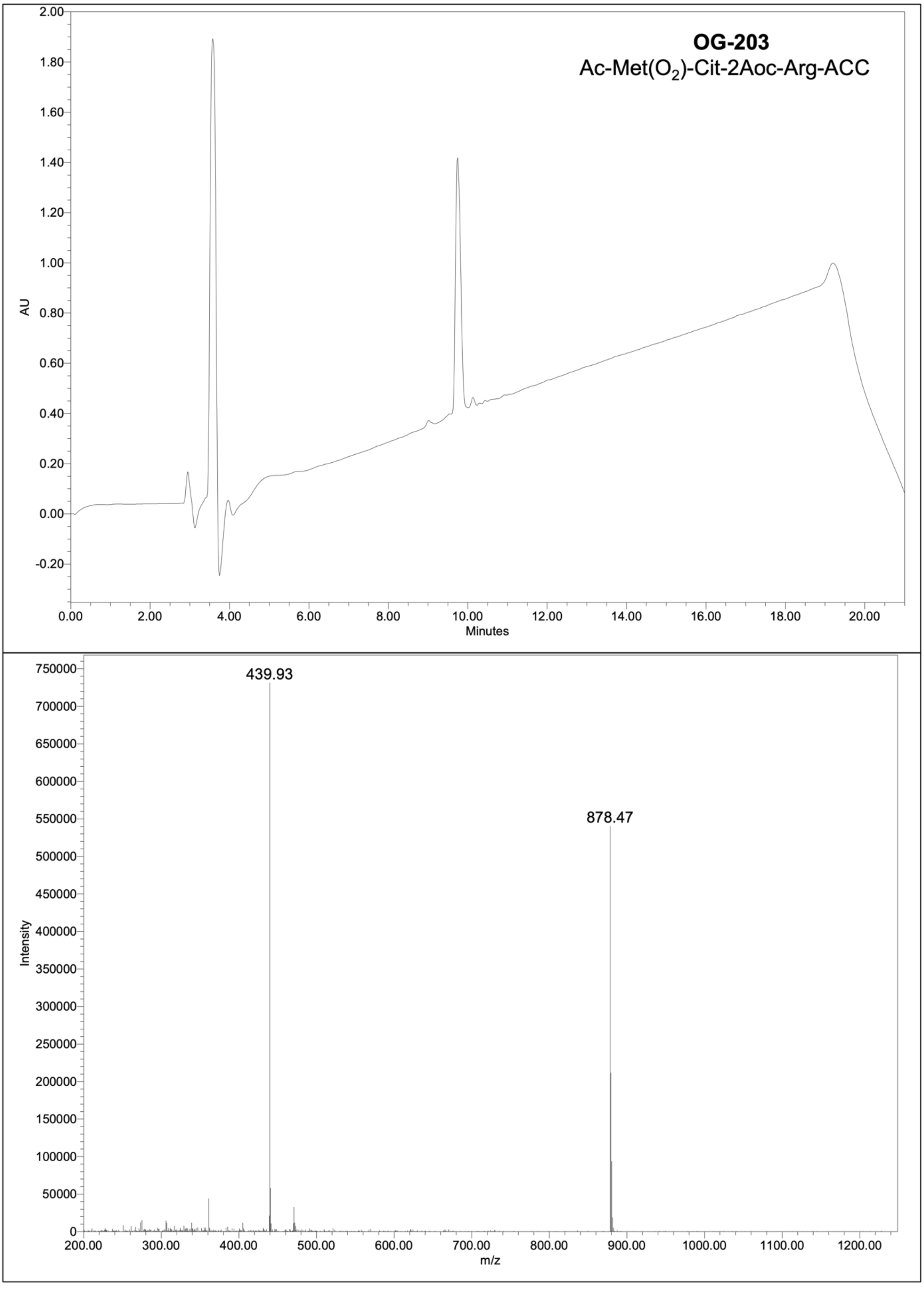
OG-203
Ac-Met(O2)-Cit-2Aoc-Arg-ACC
AU
Minutes
439.93
878.47
Intensity
m/z

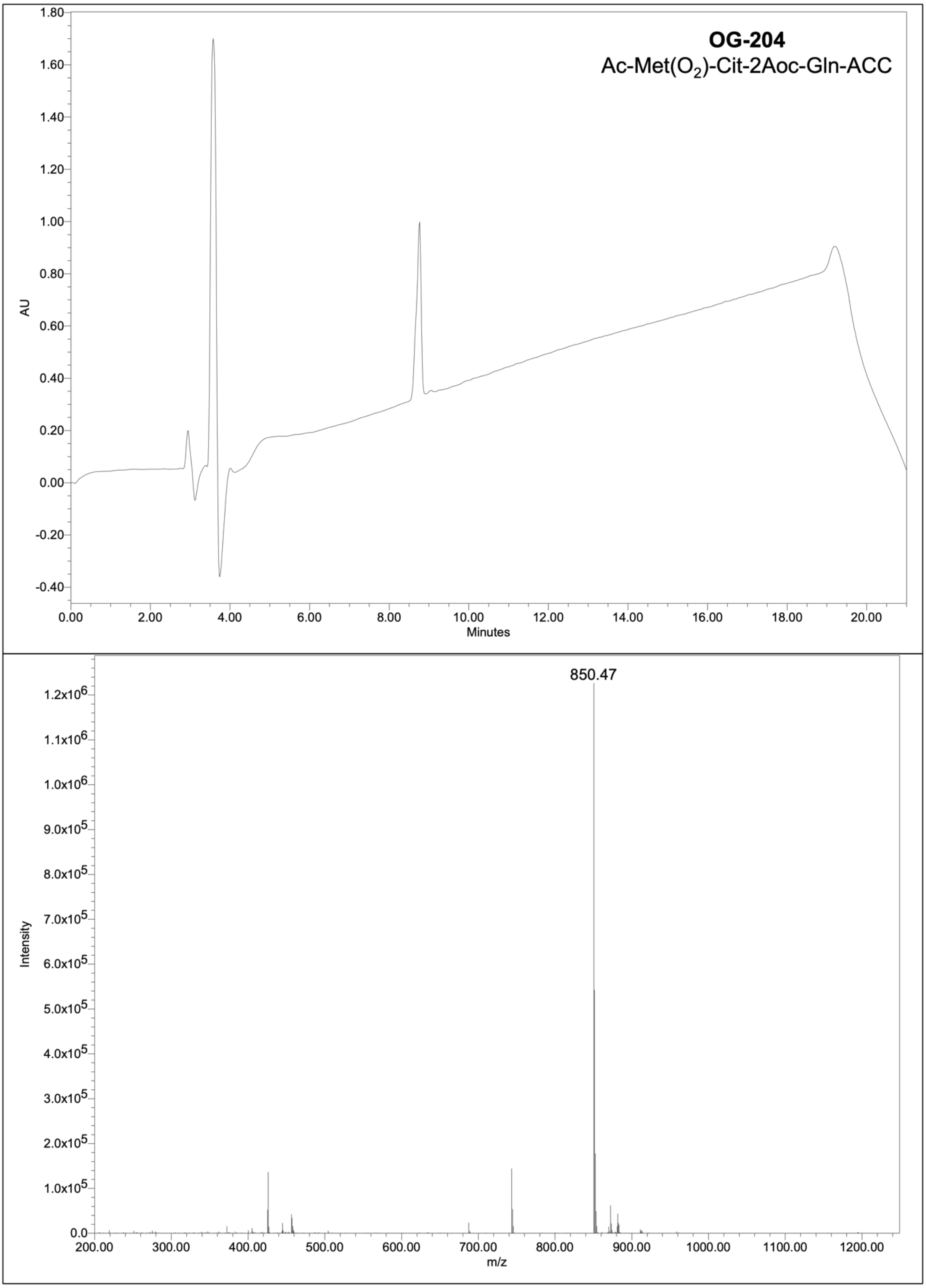
OG-204
Ac-Met(O_2)-Cit-2Aoc-Gln-ACC
AU
Minutes
850.47
Intensity
m/z

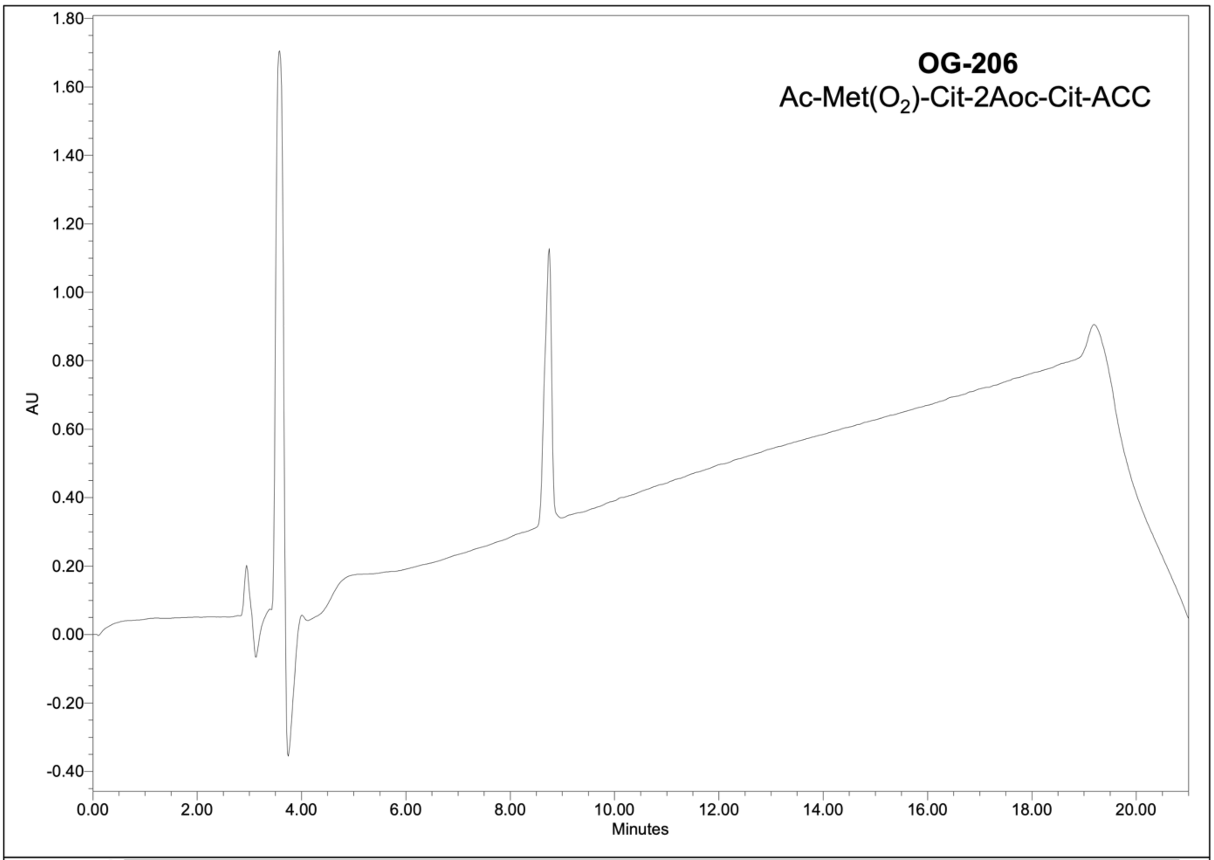
OG-206
Ac-Met(O_2)-Cit-2Aoc-Cit-ACC
AU
Minutes
1.80
1.60
1.40
1.20
1.00
0.80
0.60
0.40
0.20
0.00
-0.20
-0.40
0.00
2.00
4.00
6.00
8.00
10.00
12.00
14.00
16.00
18.00
20.00

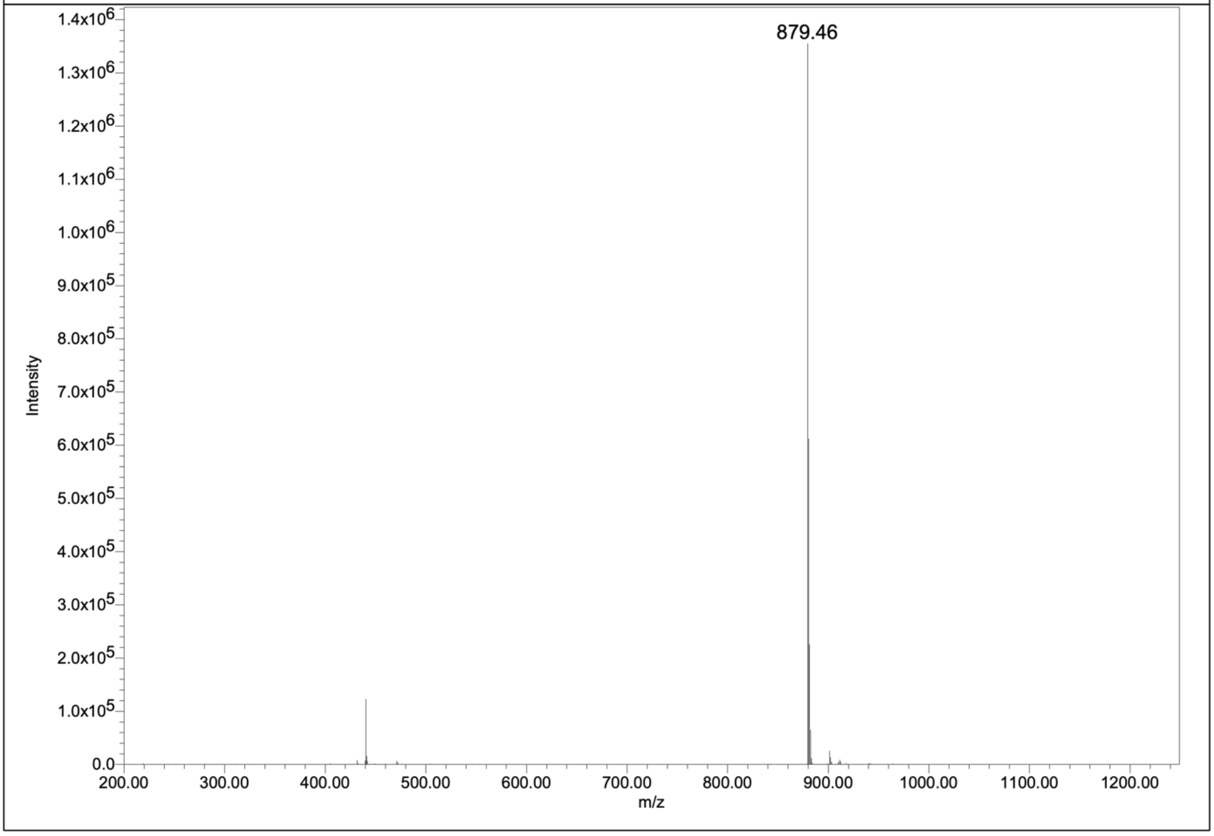
879.46
Intensity
m/z
1.4x10^6
1.3x10^6
1.2x10^6
1.1x10^6
1.0x10^6
9.0x10^5
8.0x10^5
7.0x10^5
6.0x10^5
5.0x10^5
4.0x10^5
3.0x10^5
2.0x10^5
1.0x10^5
0.0
200.00
300.00
400.00
500.00
600.00
700.00
800.00
900.00
1000.00
1100.00
1200.00

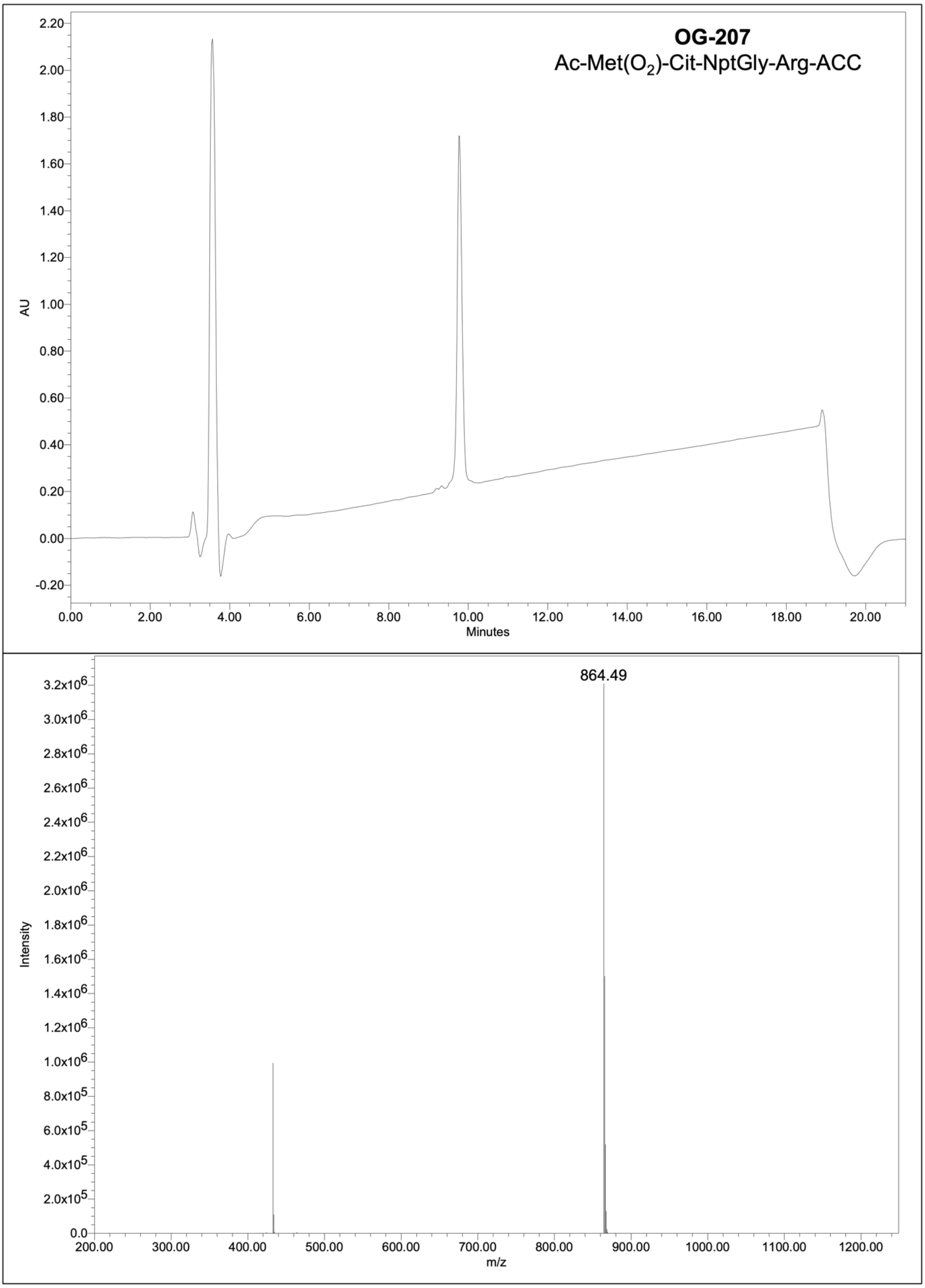

OG-207
Ac-Met(O2)-Cit-NptGly-Arg-ACC
AU
Minutes
864.49
Intensity
m/z

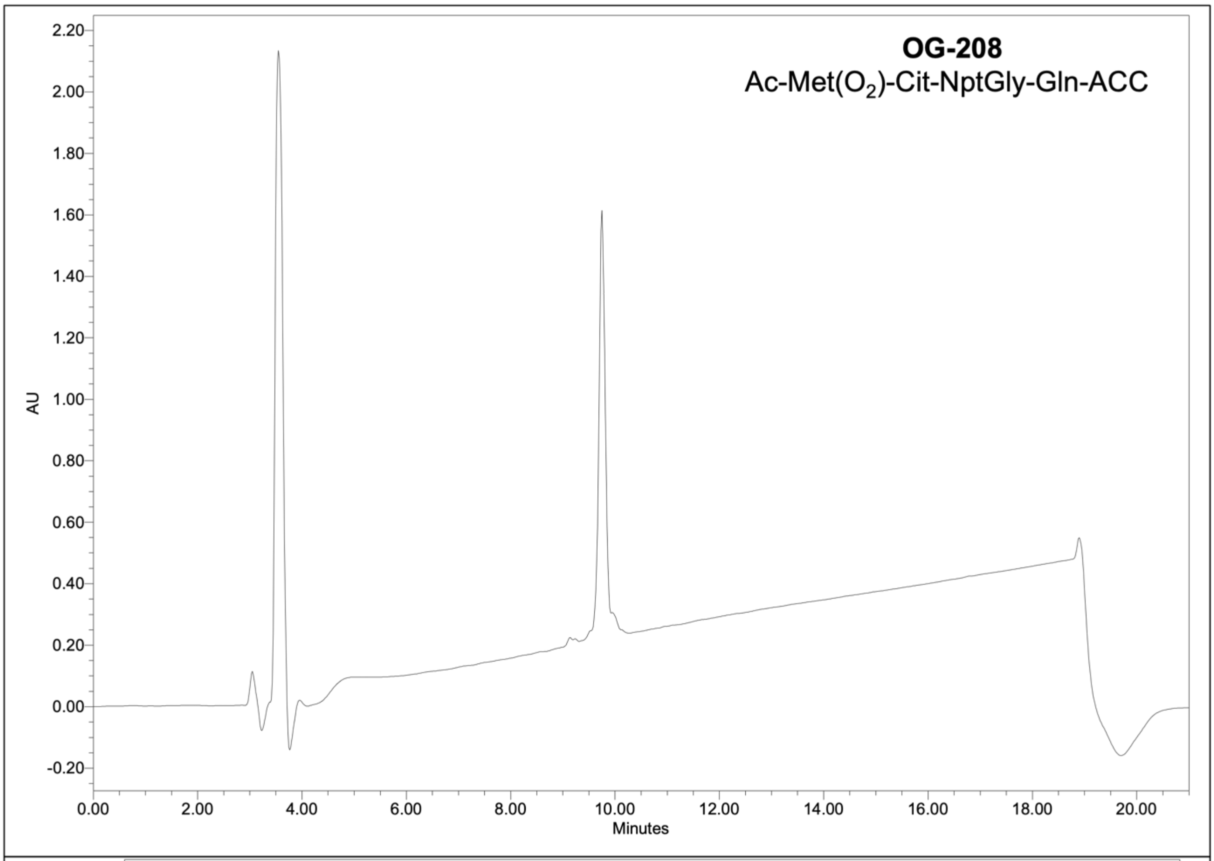
OG-208
Ac-Met(O2)-Cit-NptGly-Gln-ACC
AU
Minutes
2.20
2.00
1.80
1.60
1.40
1.20
1.00
0.80
0.60
0.40
0.20
0.00
-0.20
0.00
2.00
4.00
6.00
8.00
10.00
12.00
14.00
16.00
18.00
20.00

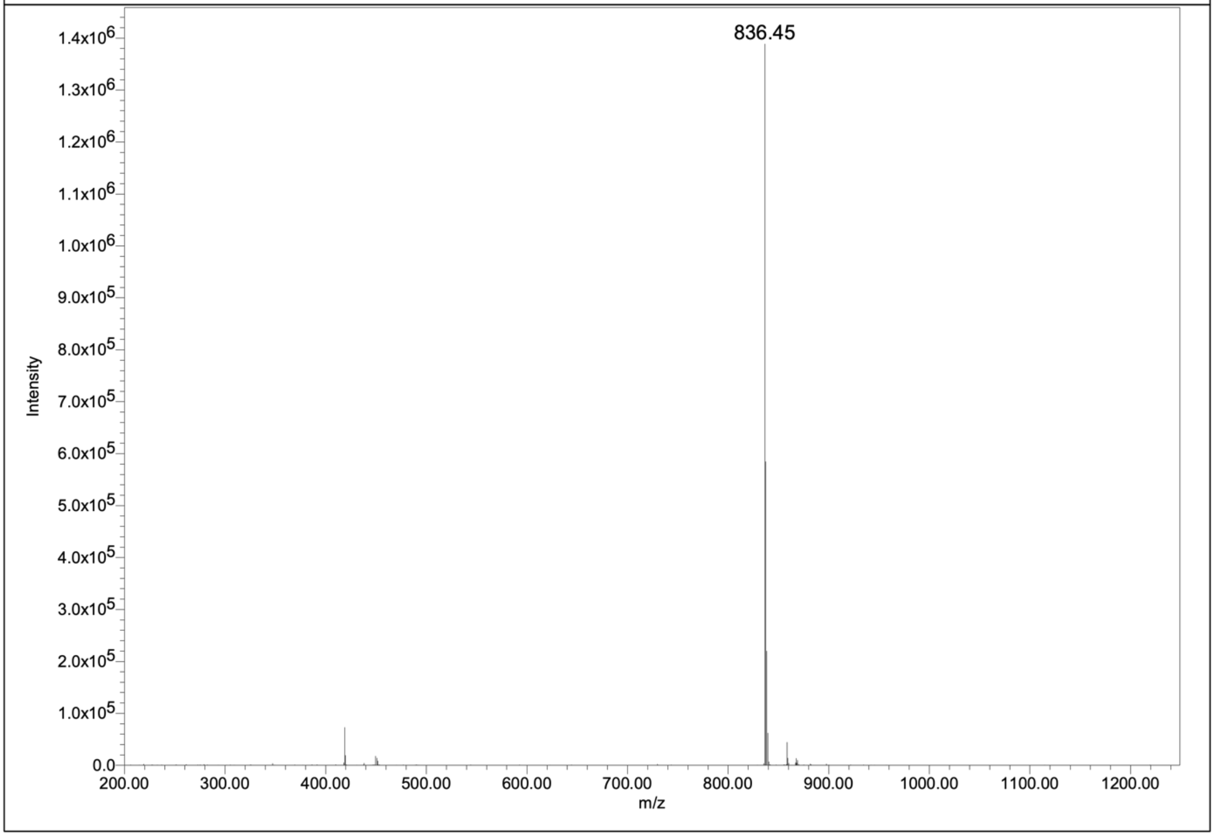
836.45
Intensity
m/z
1.4x10^6
1.3x10^6
1.2x10^6
1.1x10^6
1.0x10^6
9.0x10^5
8.0x10^5
7.0x10^5
6.0x10^5
5.0x10^5
4.0x10^5
3.0x10^5
2.0x10^5
1.0x10^5
0.0
200.00
300.00
400.00
500.00
600.00
700.00
800.00
900.00
1000.00
1100.00
1200.00

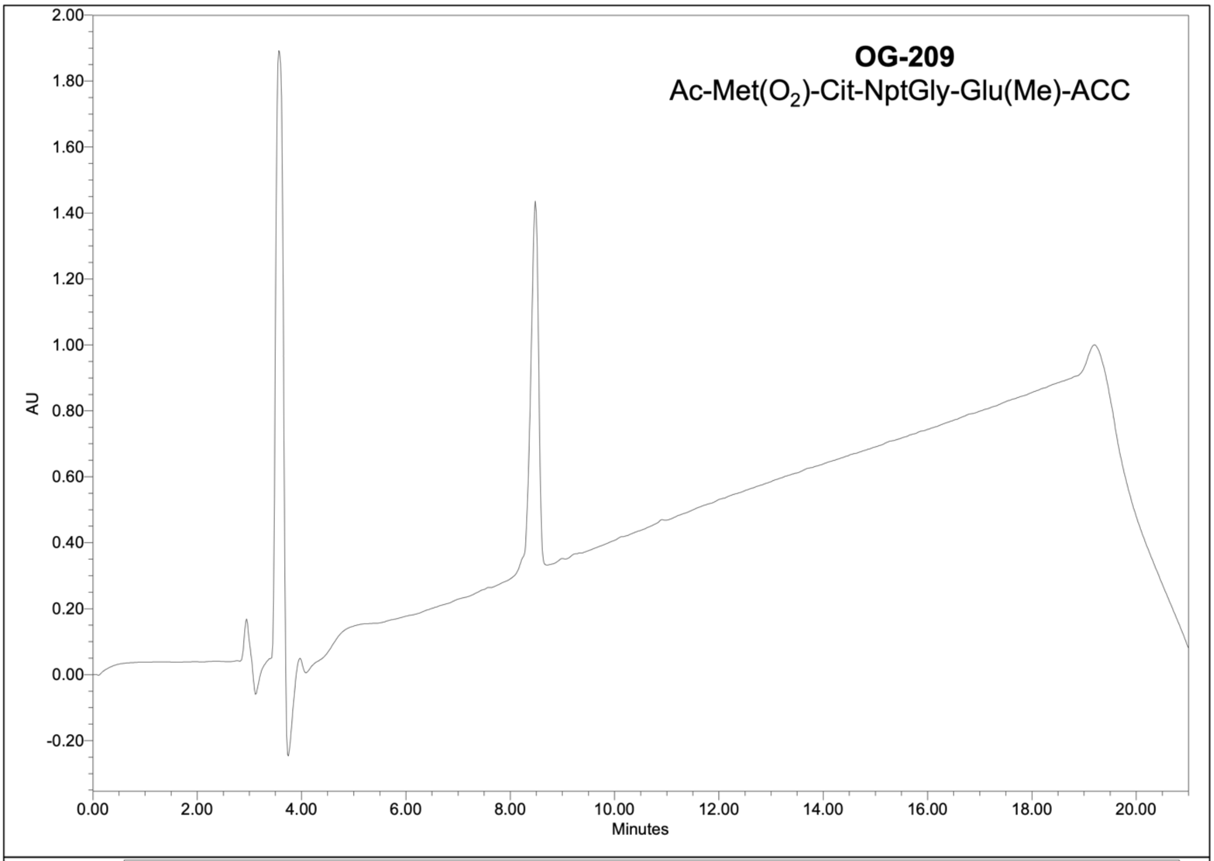
OG-209
Ac-Met(O_2)-Cit-NptGly-Glu(Me)-ACC
AU
Minutes

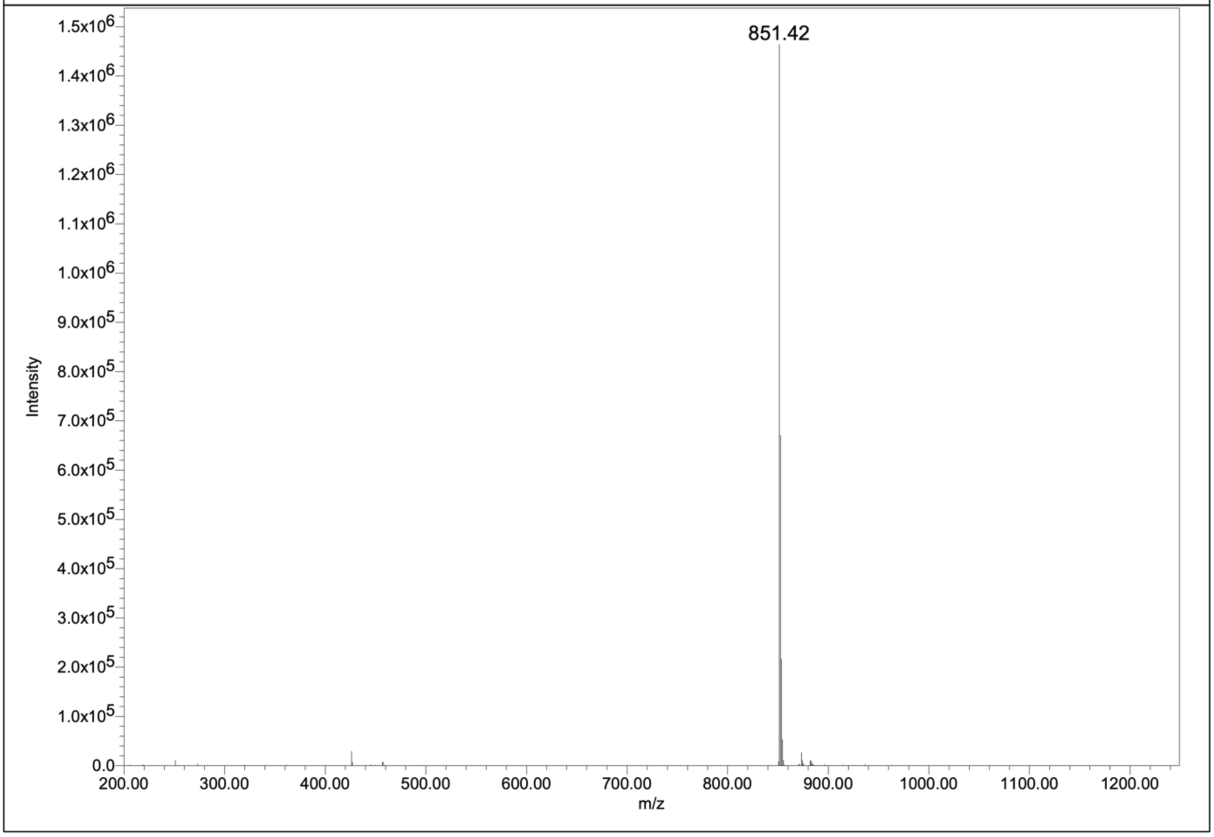
851.42
Intensity
m/z

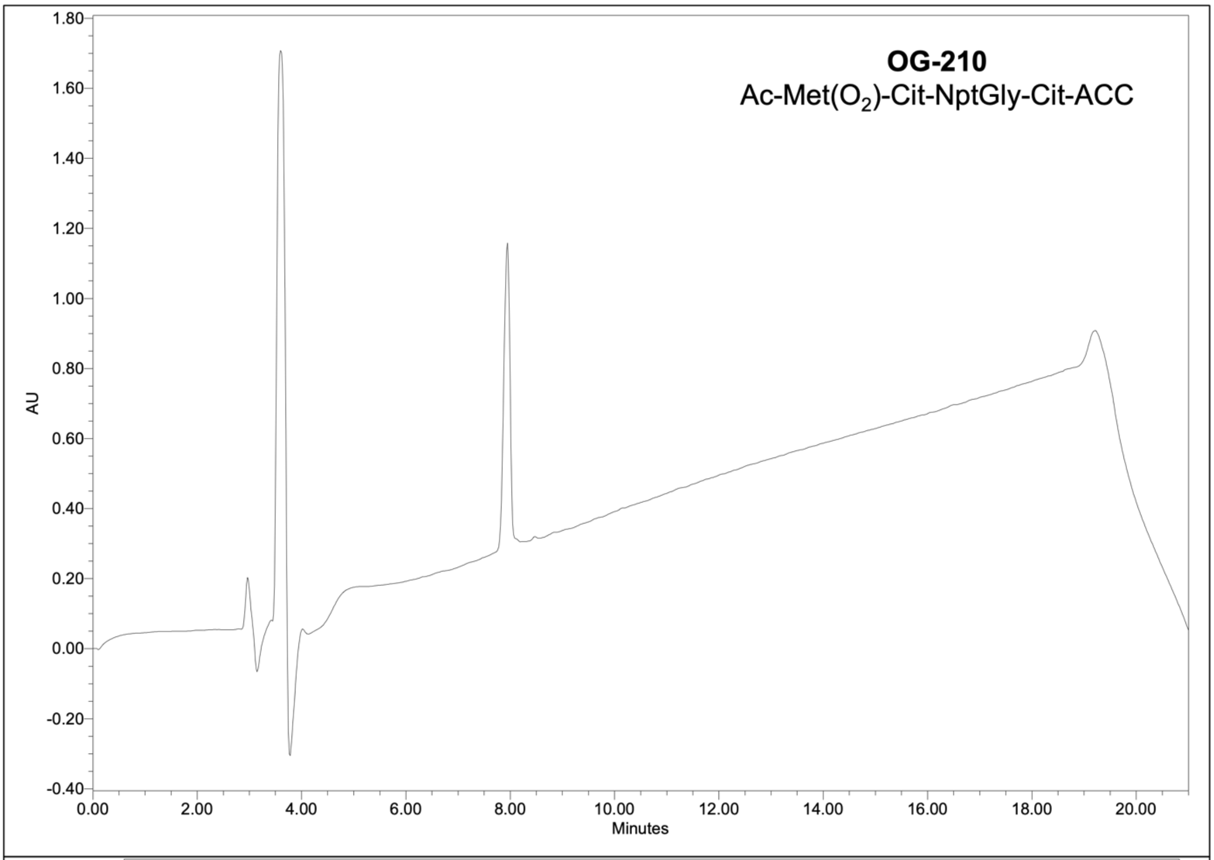
OG-210
Ac-Met(O2)-Cit-NptGly-Cit-ACC
AU
Minutes
1.80
1.60
1.40
1.20
1.00
0.80
0.60
0.40
0.20
0.00
-0.20
-0.40
0.00
2.00
4.00
6.00
8.00
10.00
12.00
14.00
16.00
18.00
20.00

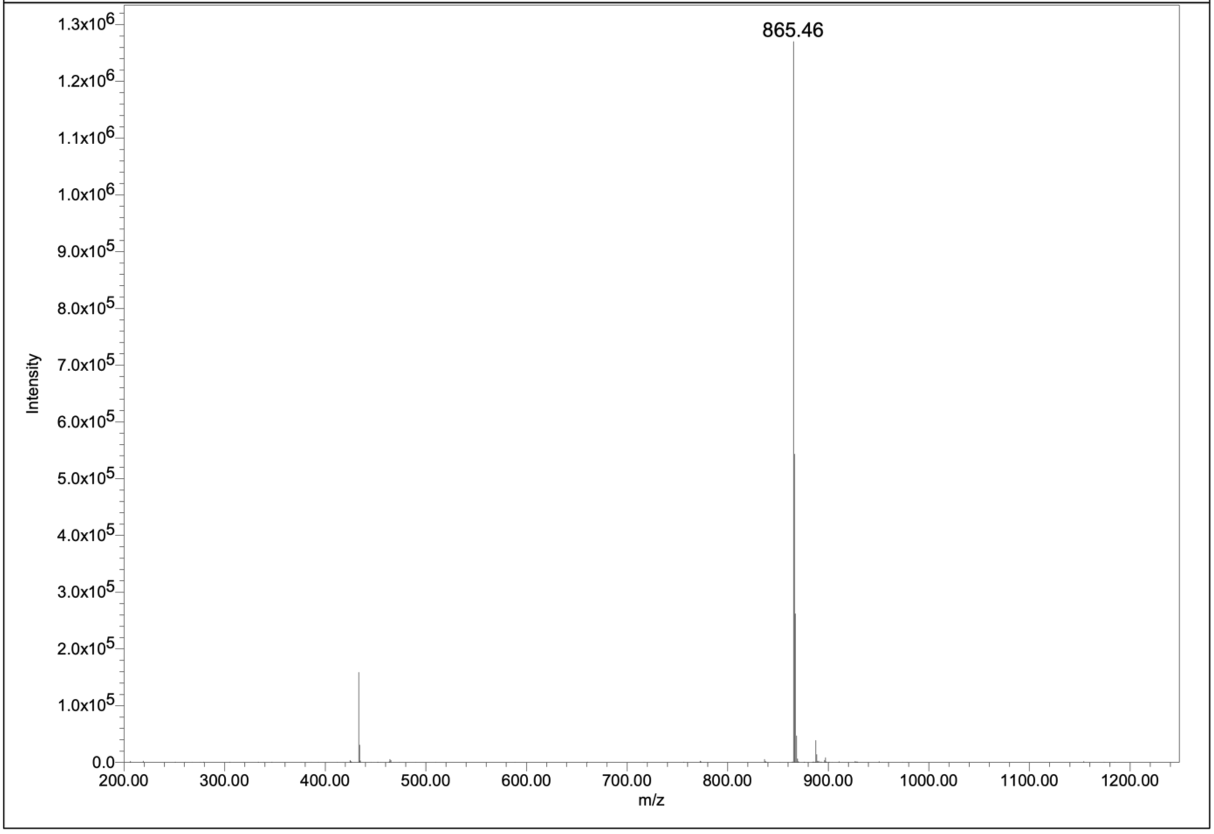
865.46
Intensity
m/z
1.3x10^6
1.2x10^6
1.1x10^6
1.0x10^6
9.0x10^5
8.0x10^5
7.0x10^5
6.0x10^5
5.0x10^5
4.0x10^5
3.0x10^5
2.0x10^5
1.0x10^5
0.0
200.00
300.00
400.00
500.00
600.00
700.00
800.00
900.00
1000.00
1100.00
1200.00

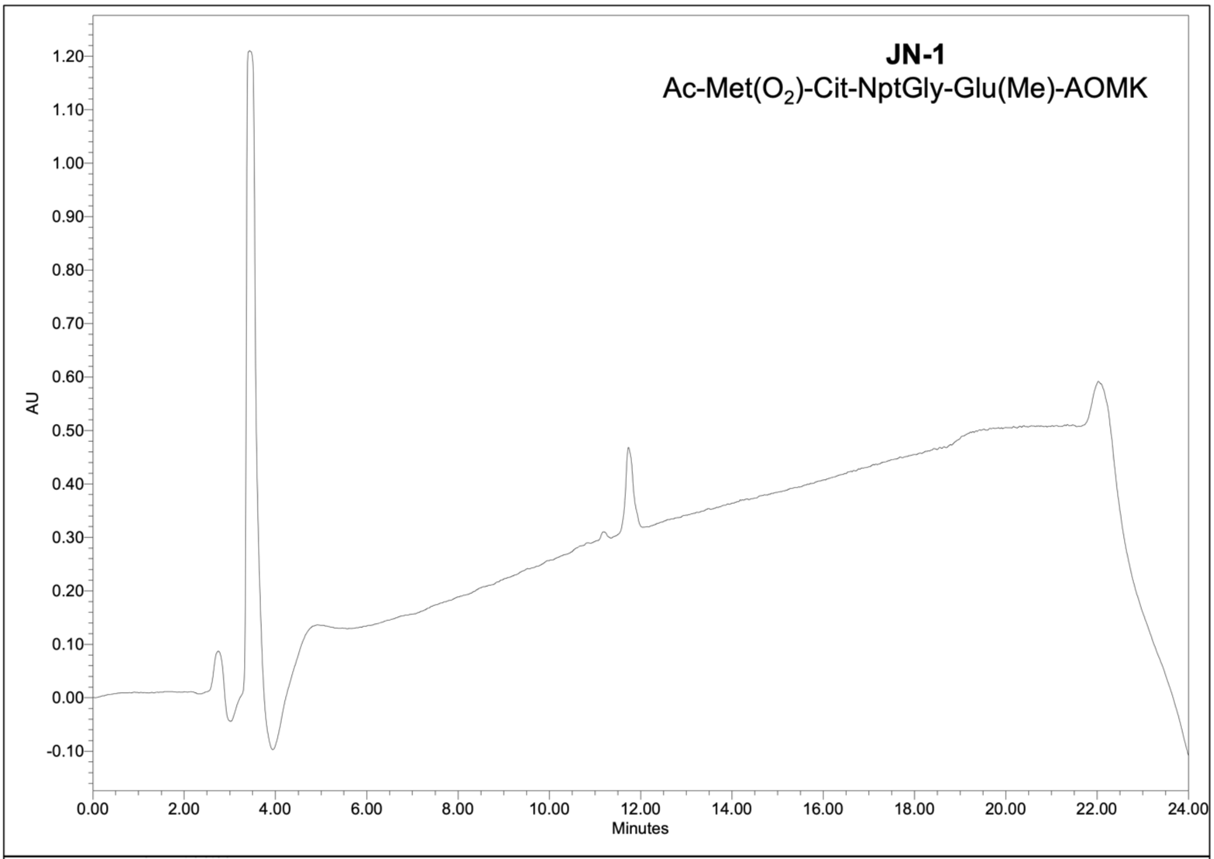
JN-1
Ac-Met(O2)-Cit-NptGly-Glu(Me)-AOMK
AU
Minutes
1.20
1.10
1.00
0.90
0.80
0.70
0.60
0.50
0.40
0.30
0.20
0.10
0.00
-0.10
0.00
2.00
4.00
6.00
8.00
10.00
12.00
14.00
16.00
18.00
20.00
22.00
24.00

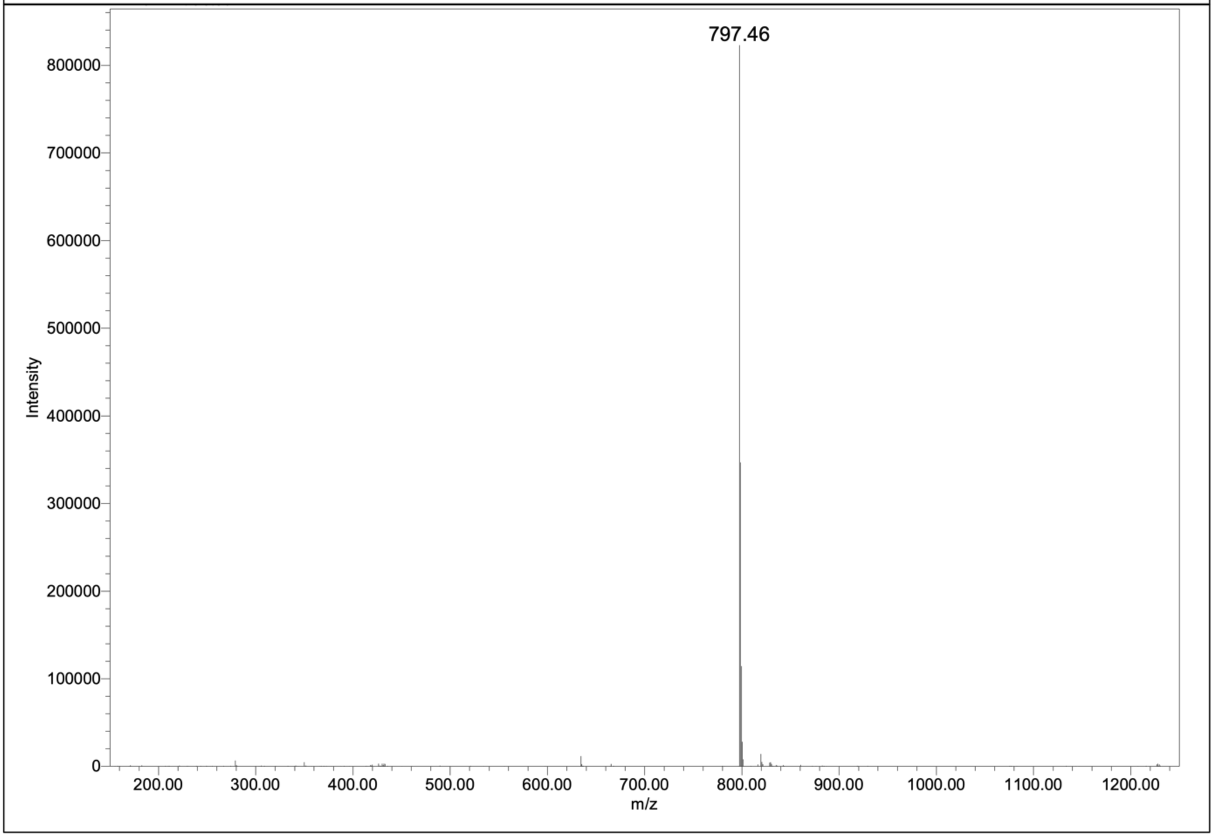
797.46
Intensity
m/z
800000
700000
600000
500000
400000
300000
200000
100000
0
200.00
300.00
400.00
500.00
600.00
700.00
800.00
900.00
1000.00
1100.00
1200.00

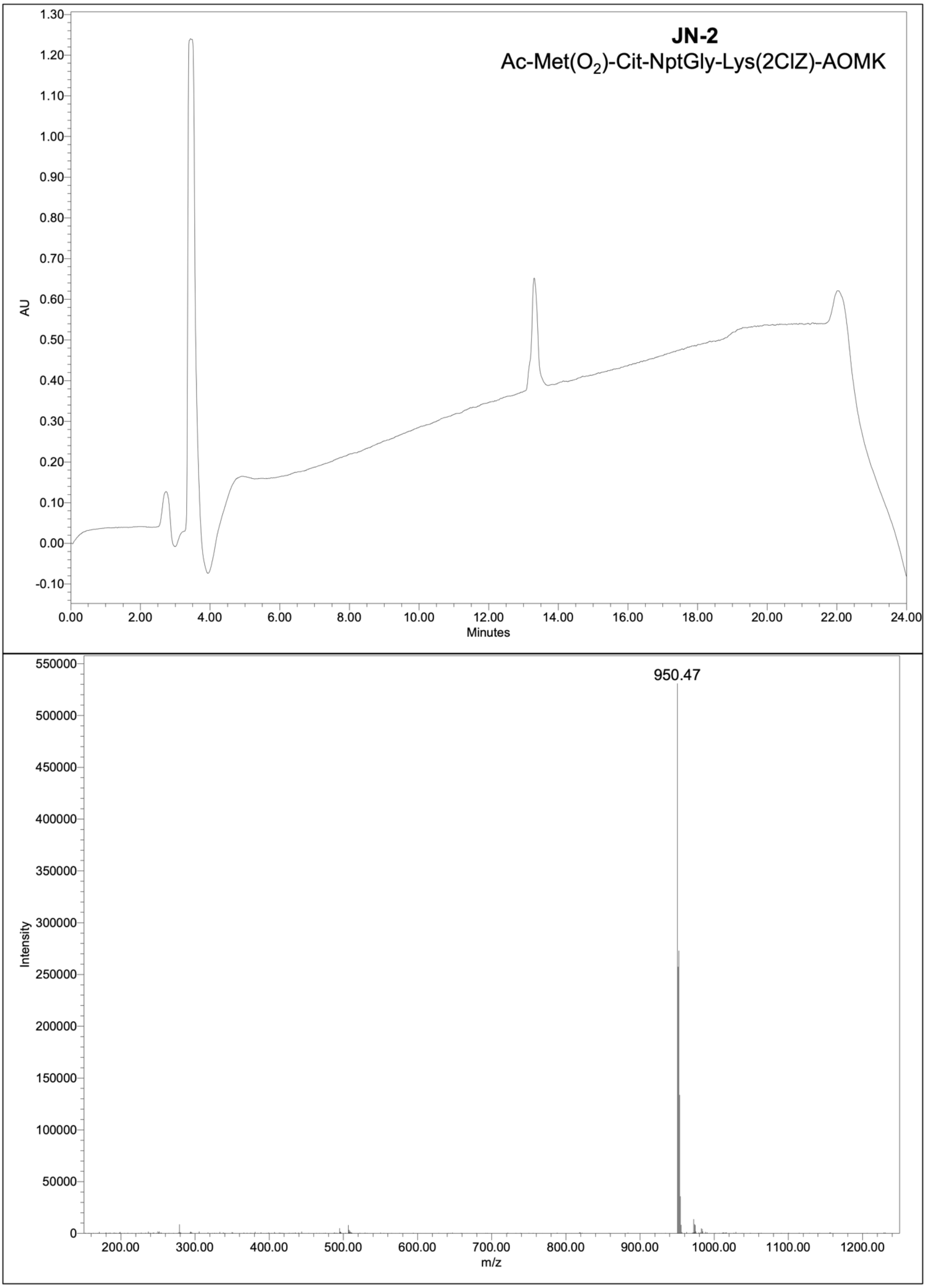
JN-2
Ac-Met(O_2)-Cit-NptGly-Lys(2ClZ)-AOMK
AU
Minutes
950.47
Intensity
m/z

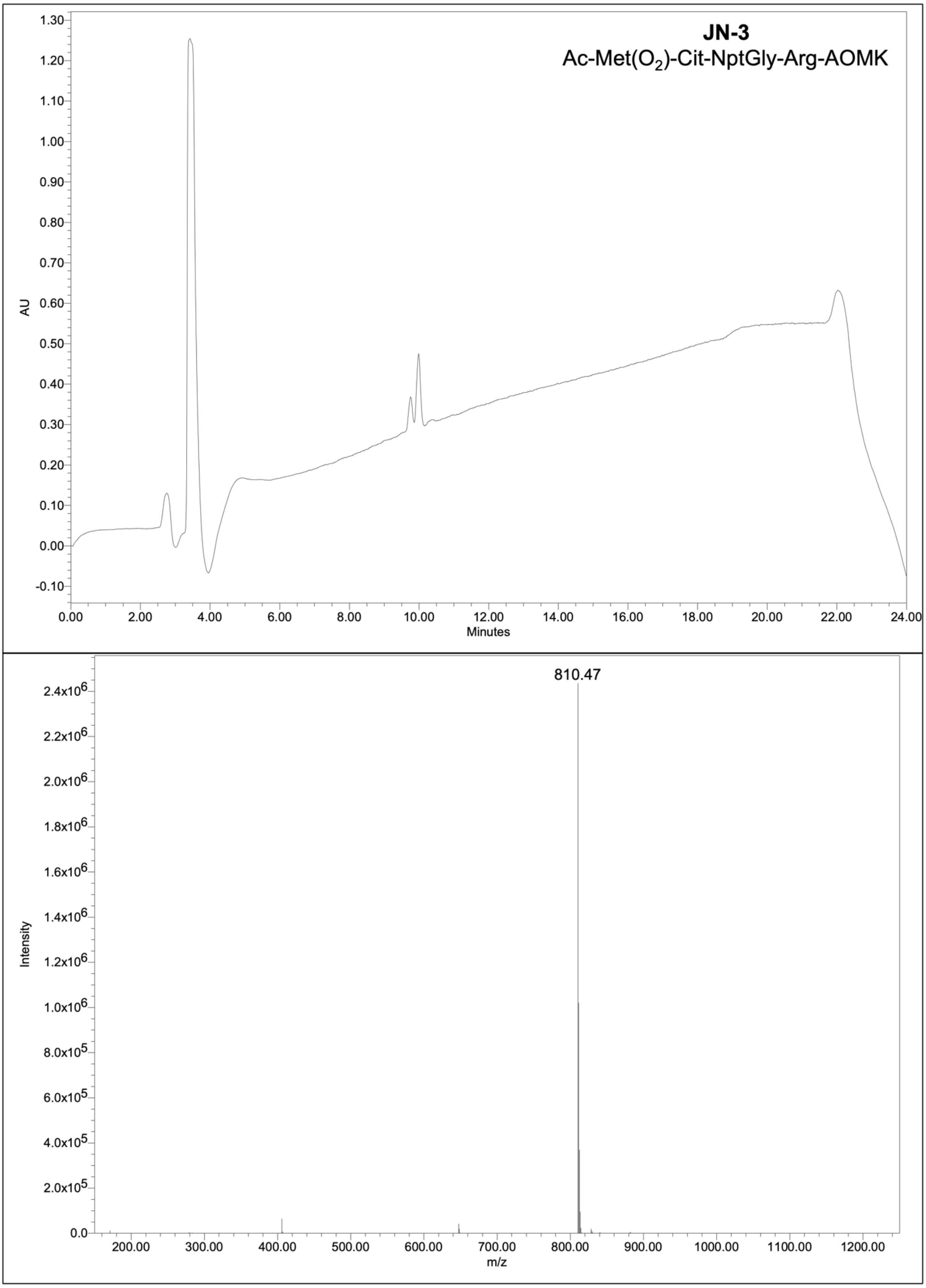
JN-3
Ac-Met(O2)-Cit-NptGly-Arg-AOMK
AU
Minutes
810.47
Intensity
m/z

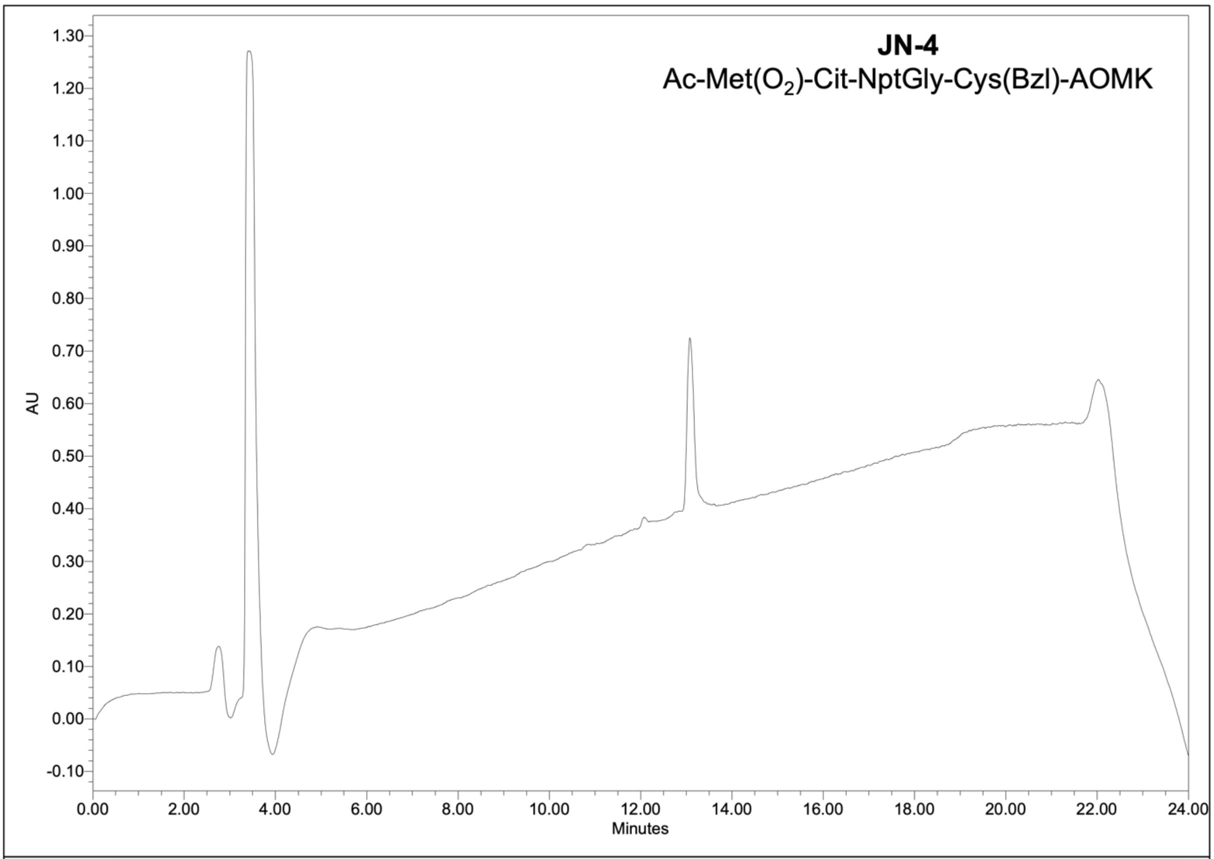
JN-4
Ac-Met(O2)-Cit-NptGly-Cys(Bzl)-AOMK
AU
Minutes
1.30
1.20
1.10
1.00
0.90
0.80
0.70
0.60
0.50
0.40
0.30
0.20
0.10
0.00
-0.10
0.00
2.00
4.00
6.00
8.00
10.00
12.00
14.00
16.00
18.00
20.00
22.00
24.00

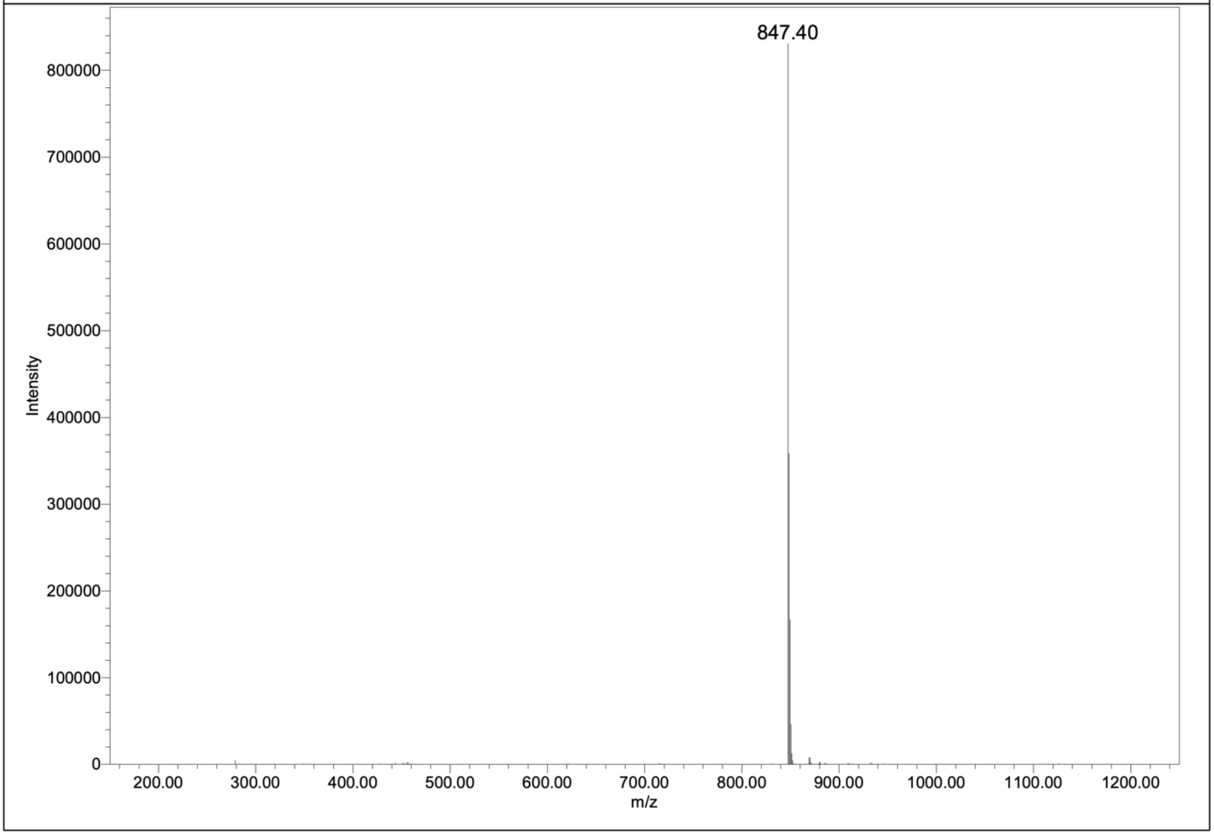
847.40
Intensity
m/z
800000
700000
600000
500000
400000
300000
200000
100000
0
200.00
300.00
400.00
500.00
600.00
700.00
800.00
900.00
1000.00
1100.00
1200.00

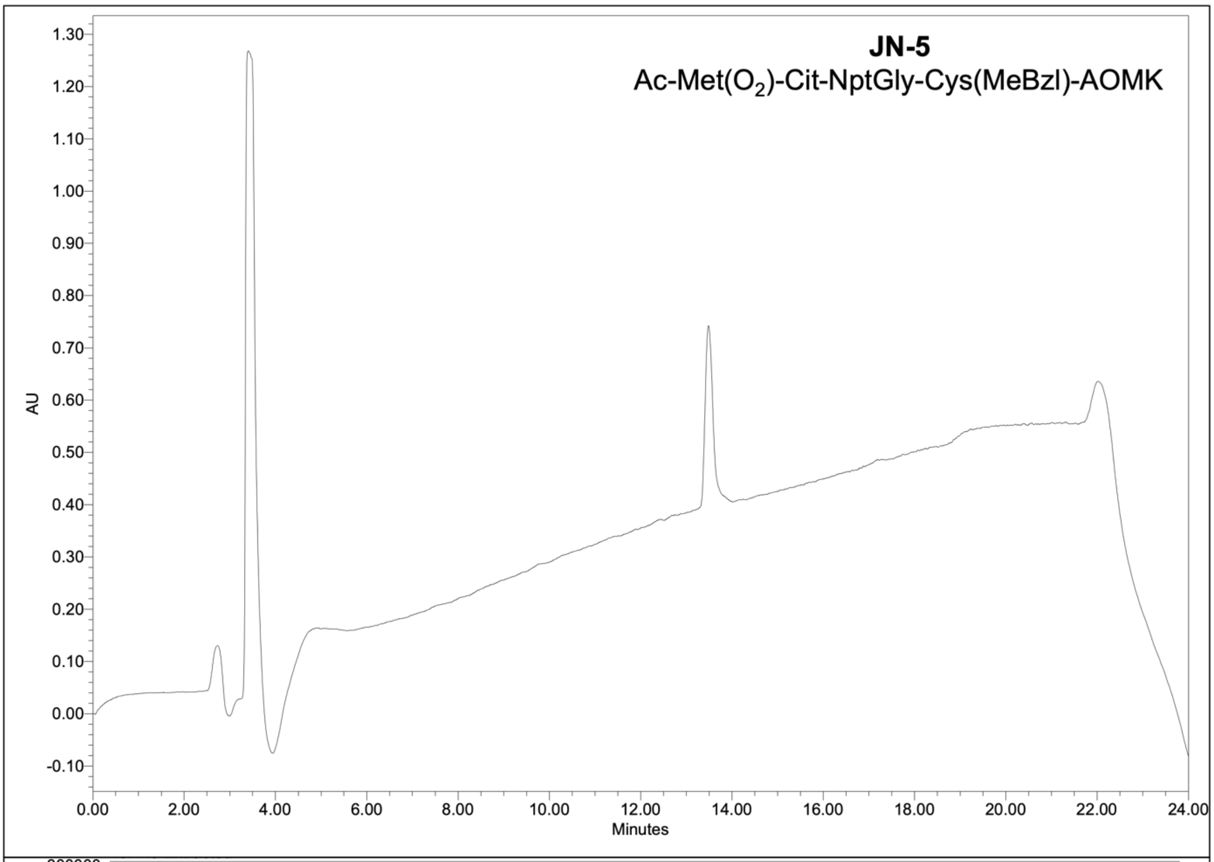
JN-5
Ac-Met(O2)-Cit-NptGly-Cys(MeBzl)-AOMK
AU
Minutes

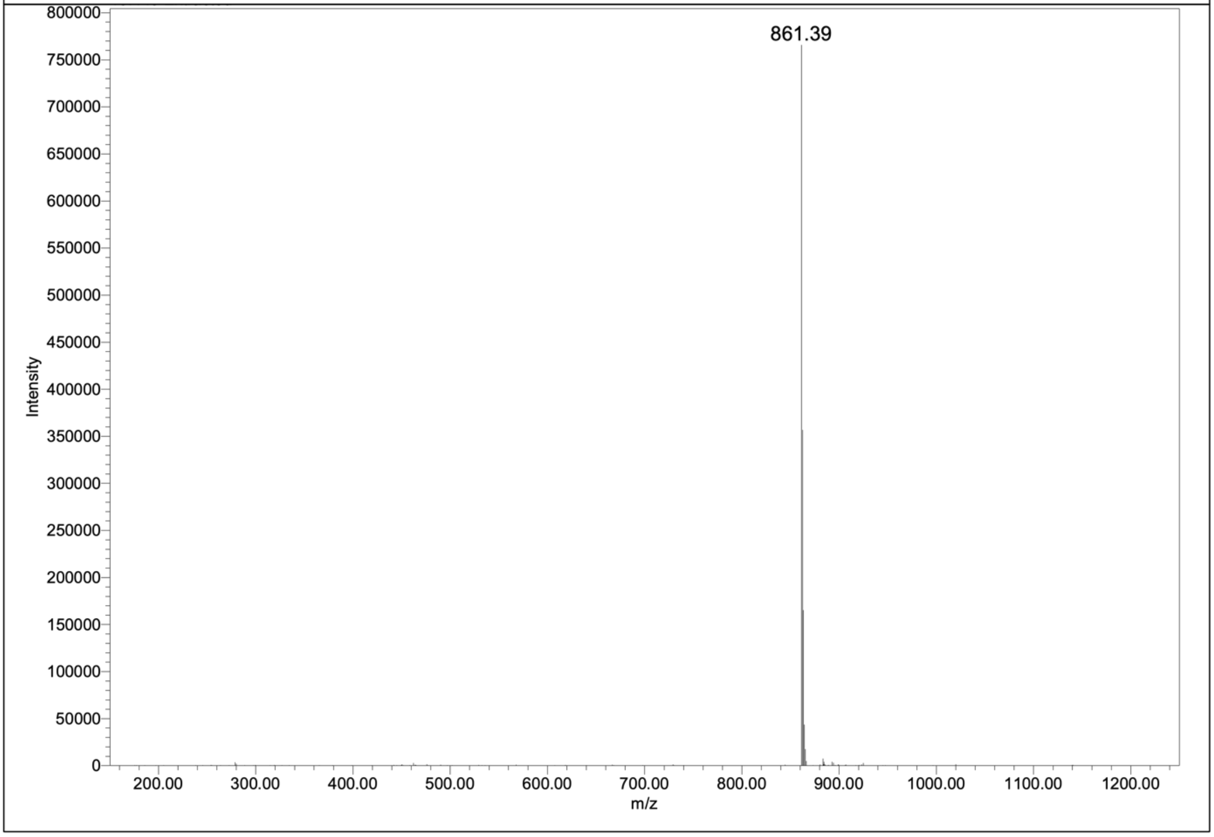
861.39
Intensity
m/z

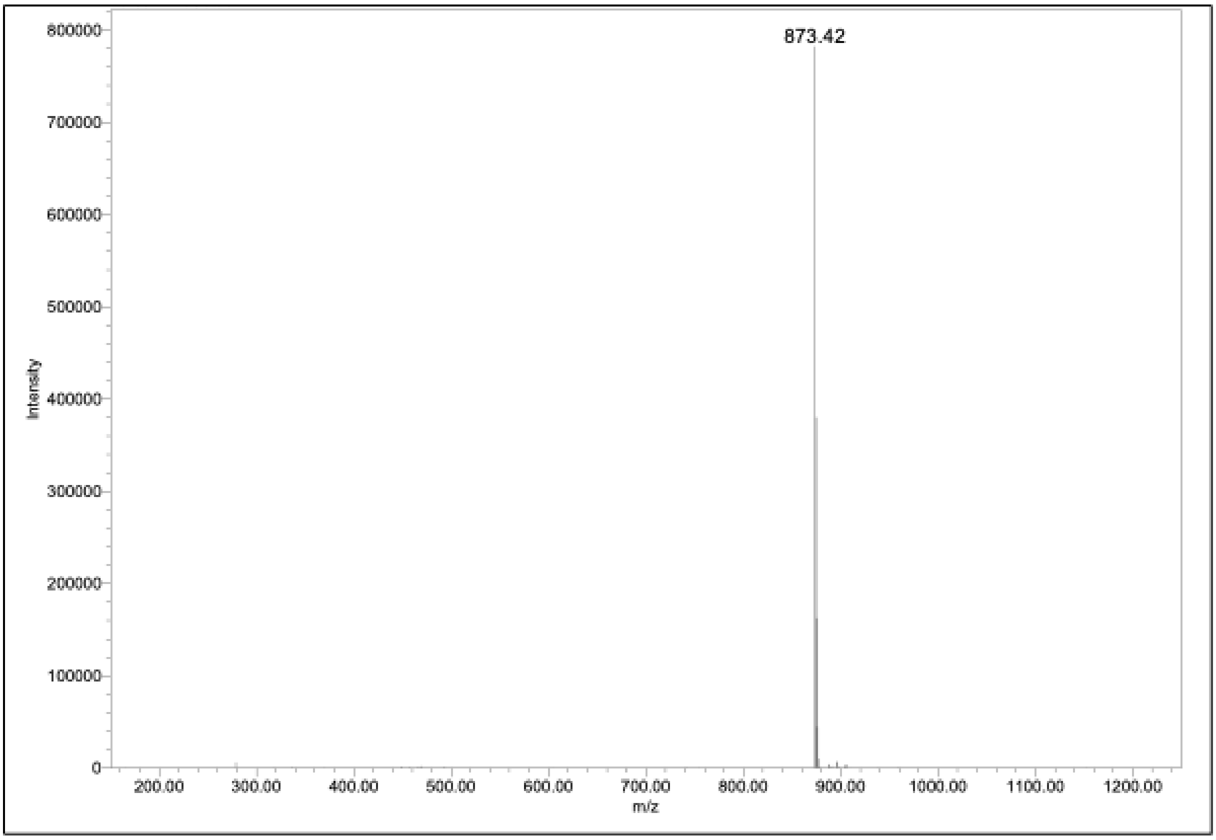
873.42
Intensity
800000
700000
600000
500000
400000
300000
200000
100000
0
200.00 300.00 400.00 500.00 600.00 700.00 800.00 900.00 1000.00 1100.00 1200.00
m/z

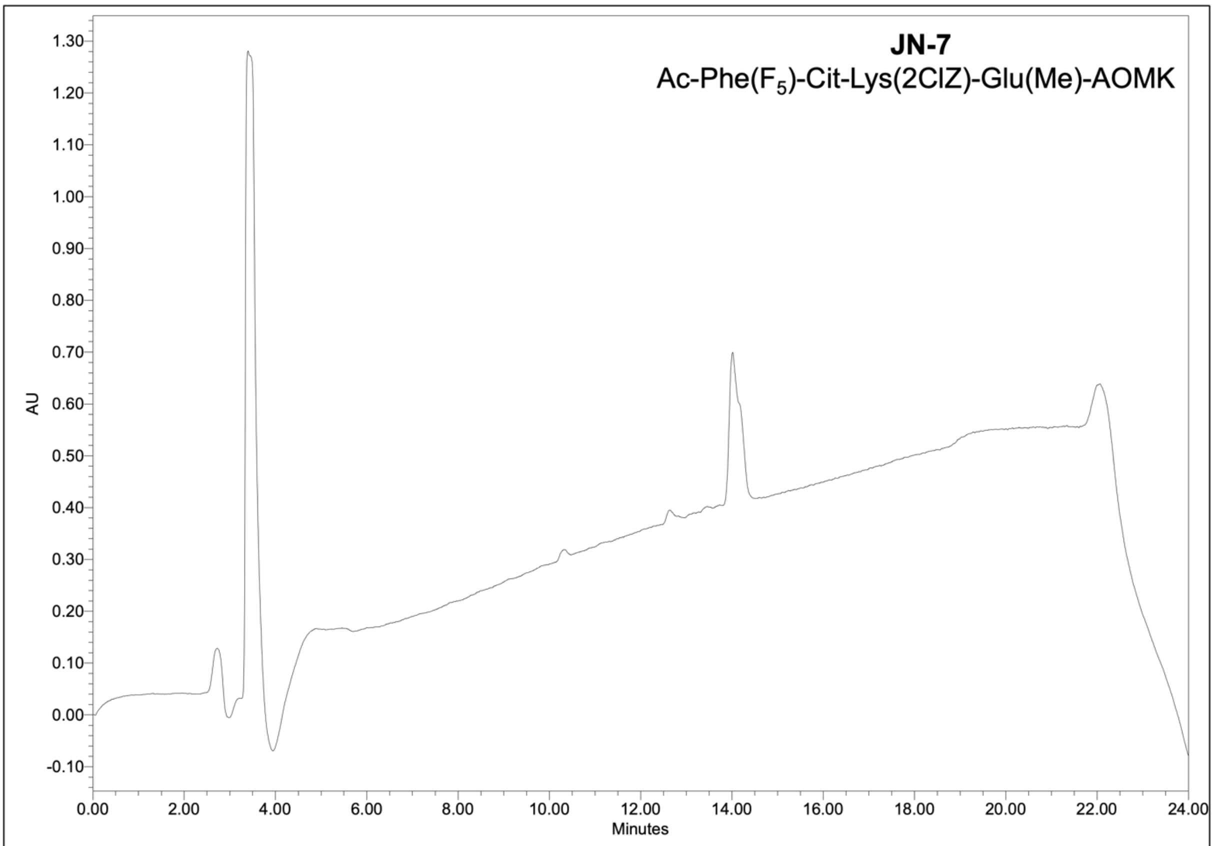
JN-7
Ac-Phe(F5)-Cit-Lys(2ClZ)-Glu(Me)-AOMK
AU
Minutes
1.30
1.20
1.10
1.00
0.90
0.80
0.70
0.60
0.50
0.40
0.30
0.20
0.10
0.00
-0.10
0.00
2.00
4.00
6.00
8.00
10.00
12.00
14.00
16.00
18.00
20.00
22.00
24.00

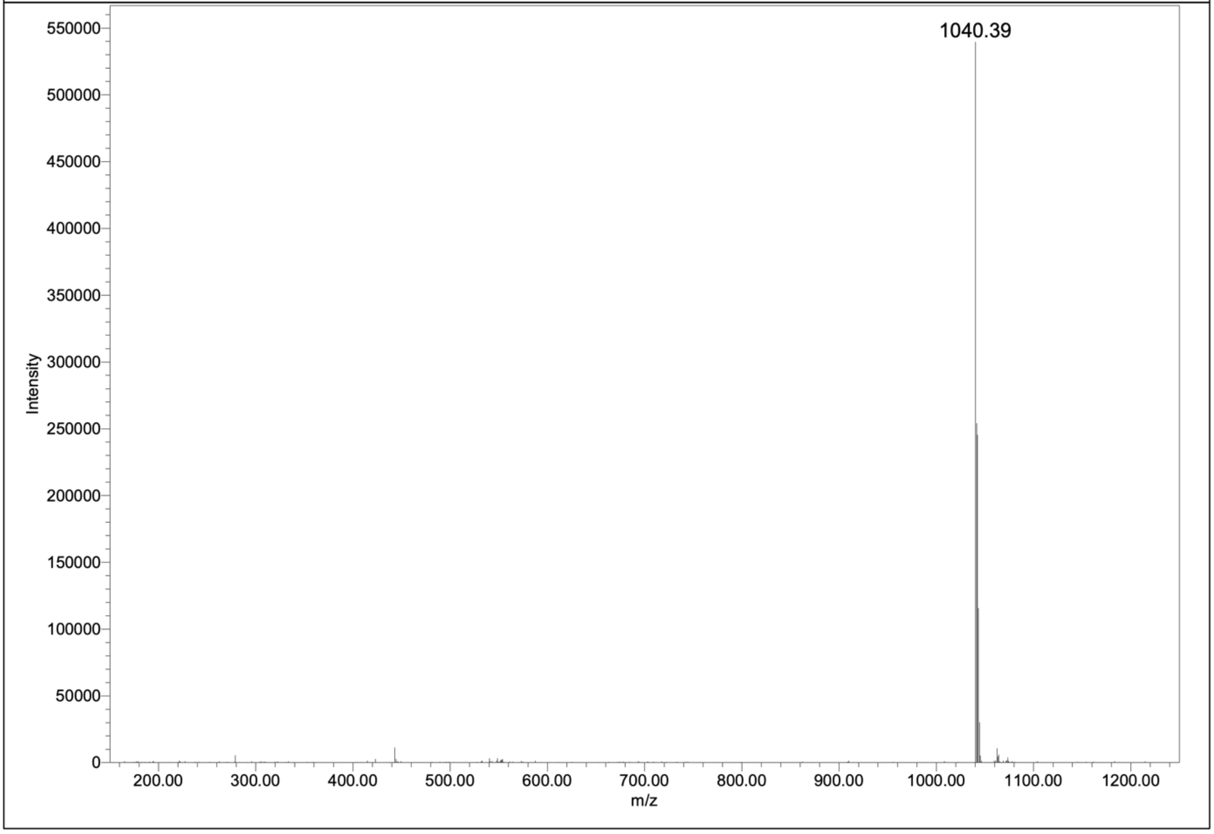
1040.39
Intensity
m/z
550000
500000
450000
400000
350000
300000
250000
200000
150000
100000
50000
0
200.00
300.00
400.00
500.00
600.00
700.00
800.00
900.00
1000.00
1100.00
1200.00

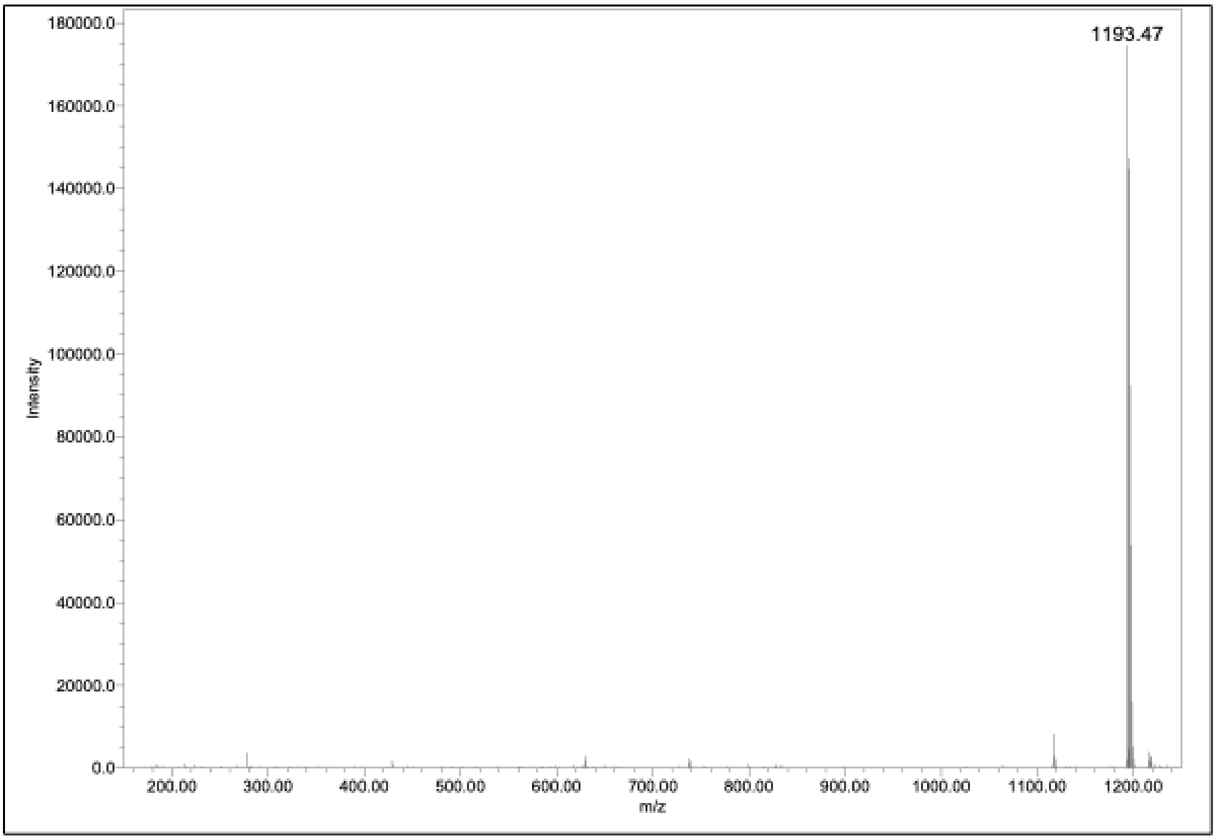

1193.47
180000.0
160000.0
140000.0
120000.0
100000.0
80000.0
60000.0
40000.0
20000.0
0.0
Intensity
200.00
300.00
400.00
500.00
600.00
700.00
800.00
900.00
1000.00
1100.00
1200.00
m/z

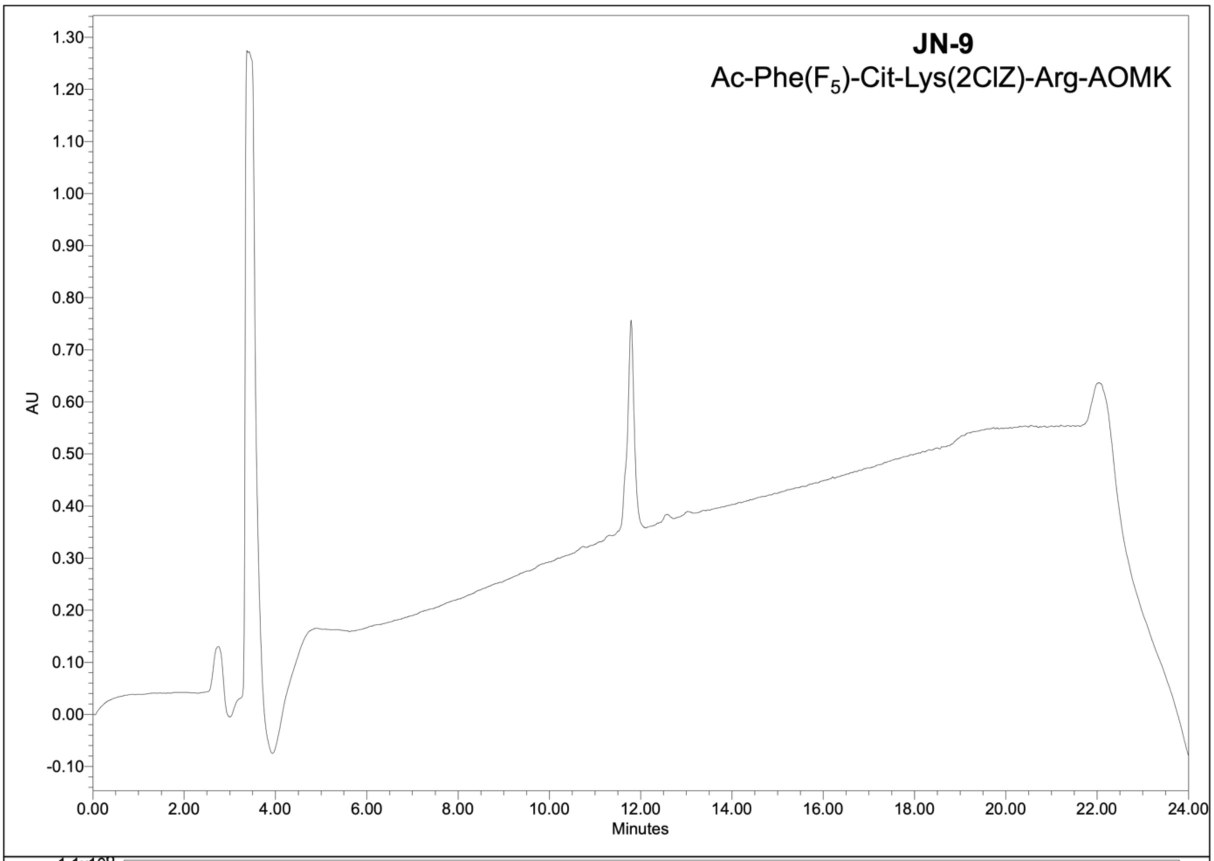
JN-9
Ac-Phe(F5)-Cit-Lys(2ClZ)-Arg-AOMK
AU
Minutes
1.30
1.20
1.10
1.00
0.90
0.80
0.70
0.60
0.50
0.40
0.30
0.20
0.10
0.00
-0.10
0.00
2.00
4.00
6.00
8.00
10.00
12.00
14.00
16.00
18.00
20.00
22.00
24.00

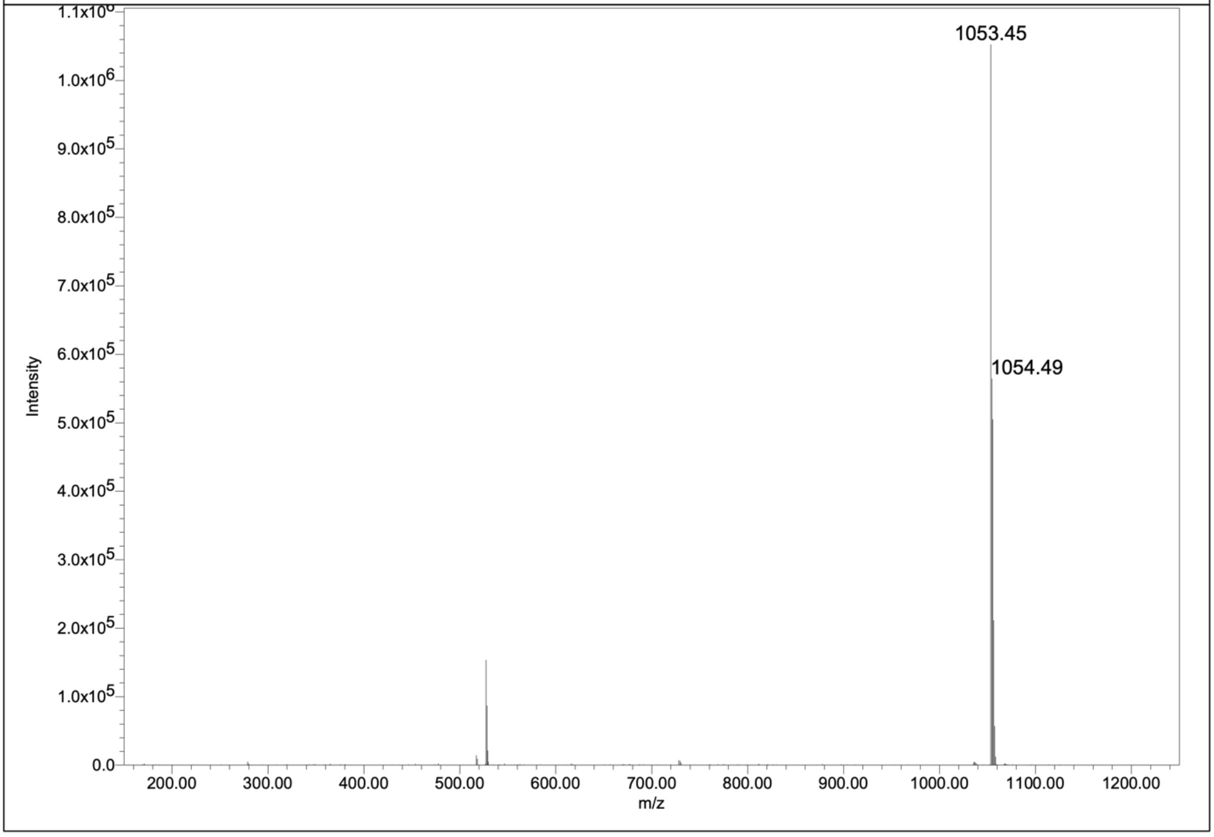
1053.45
1054.49
Intensity
m/z
1.0x10^6
9.0x10^5
8.0x10^5
7.0x10^5
6.0x10^5
5.0x10^5
4.0x10^5
3.0x10^5
2.0x10^5
1.0x10^5
0.0
200.00
300.00
400.00
500.00
600.00
700.00
800.00
900.00
1000.00
1100.00
1200.00

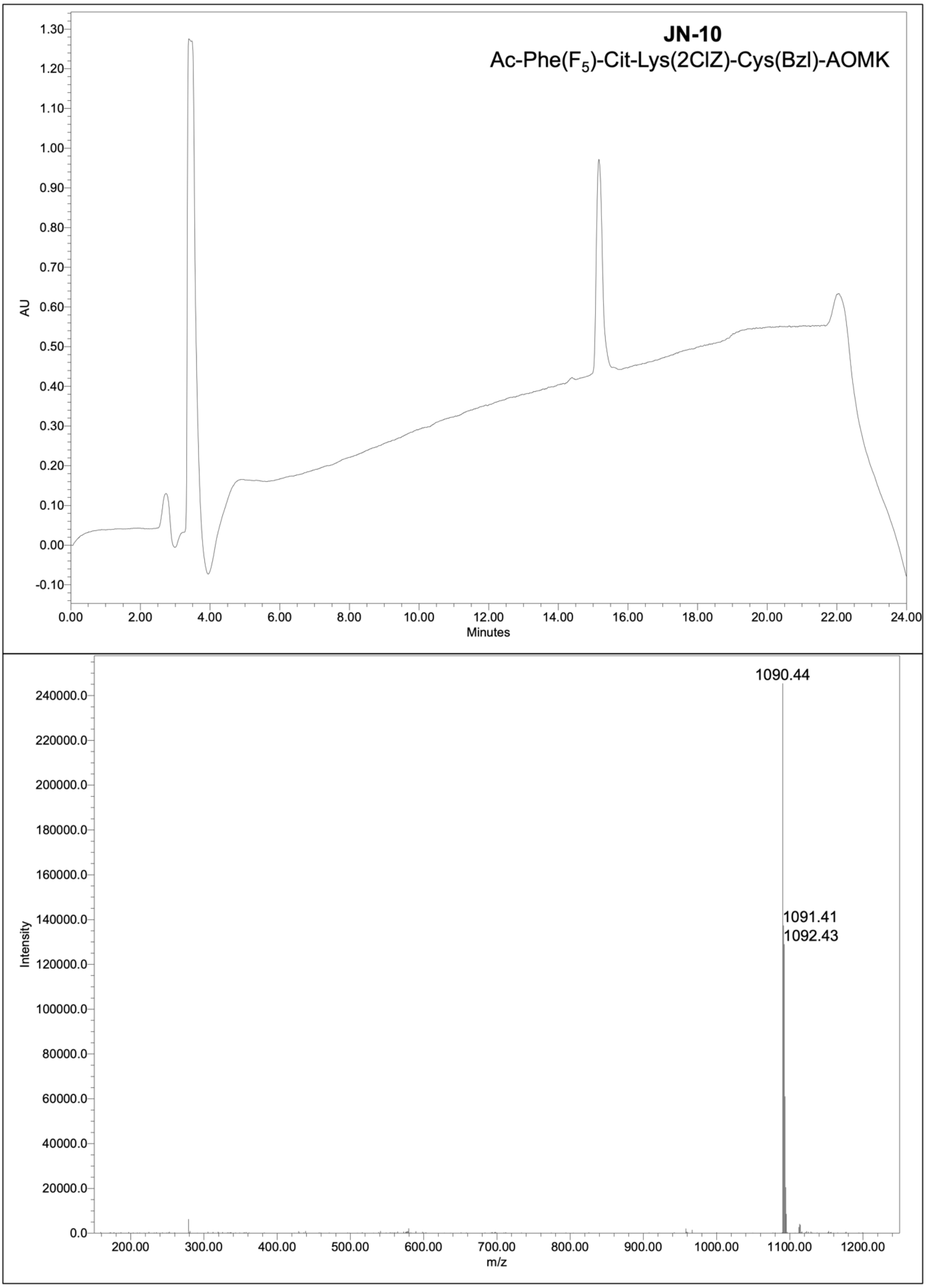
JN-10
Ac-Phe(F5)-Cit-Lys(2ClZ)-Cys(Bzl)-AOMK
AU
Minutes
1090.44
1091.41
1092.43
Intensity
m/z

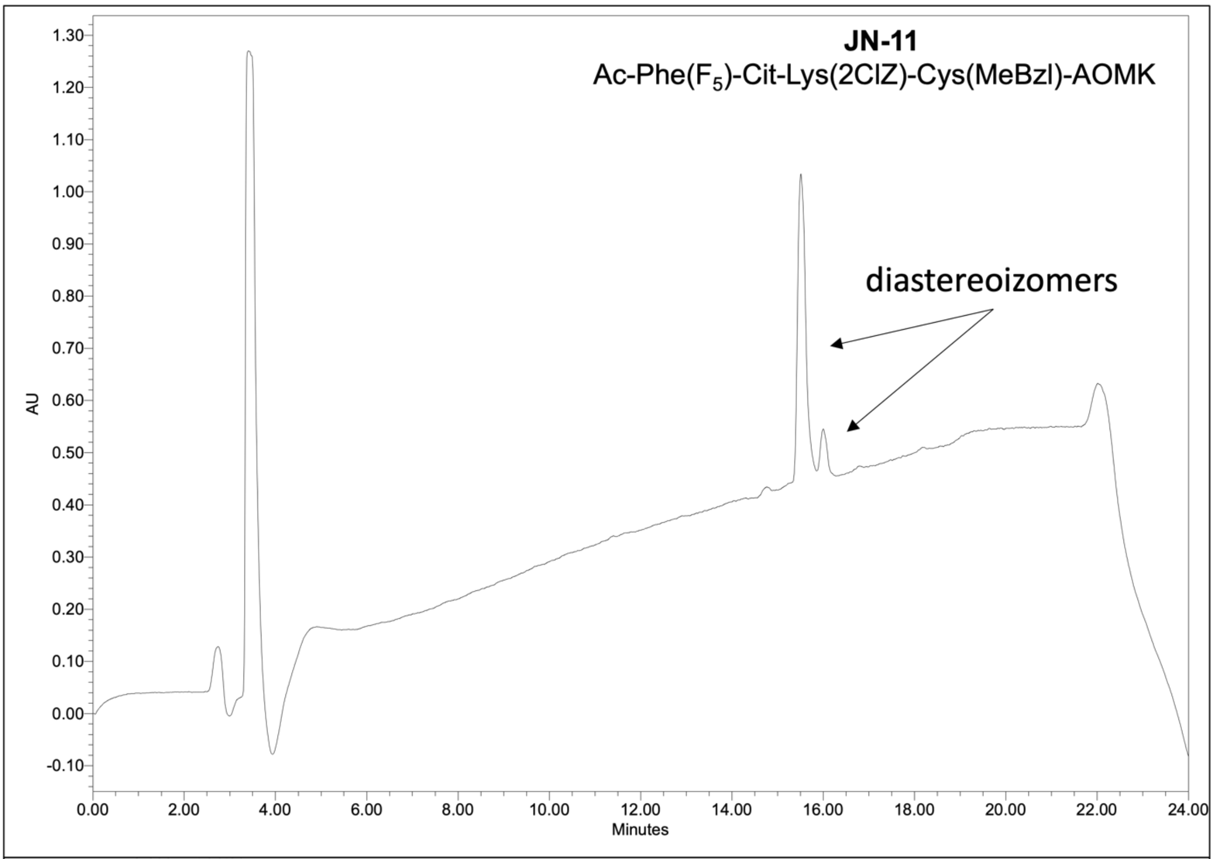
JN-11
Ac-Phe(F5)-Cit-Lys(2ClZ)-Cys(MeBzl)-AOMK
diastereoizomers
AU
Minutes

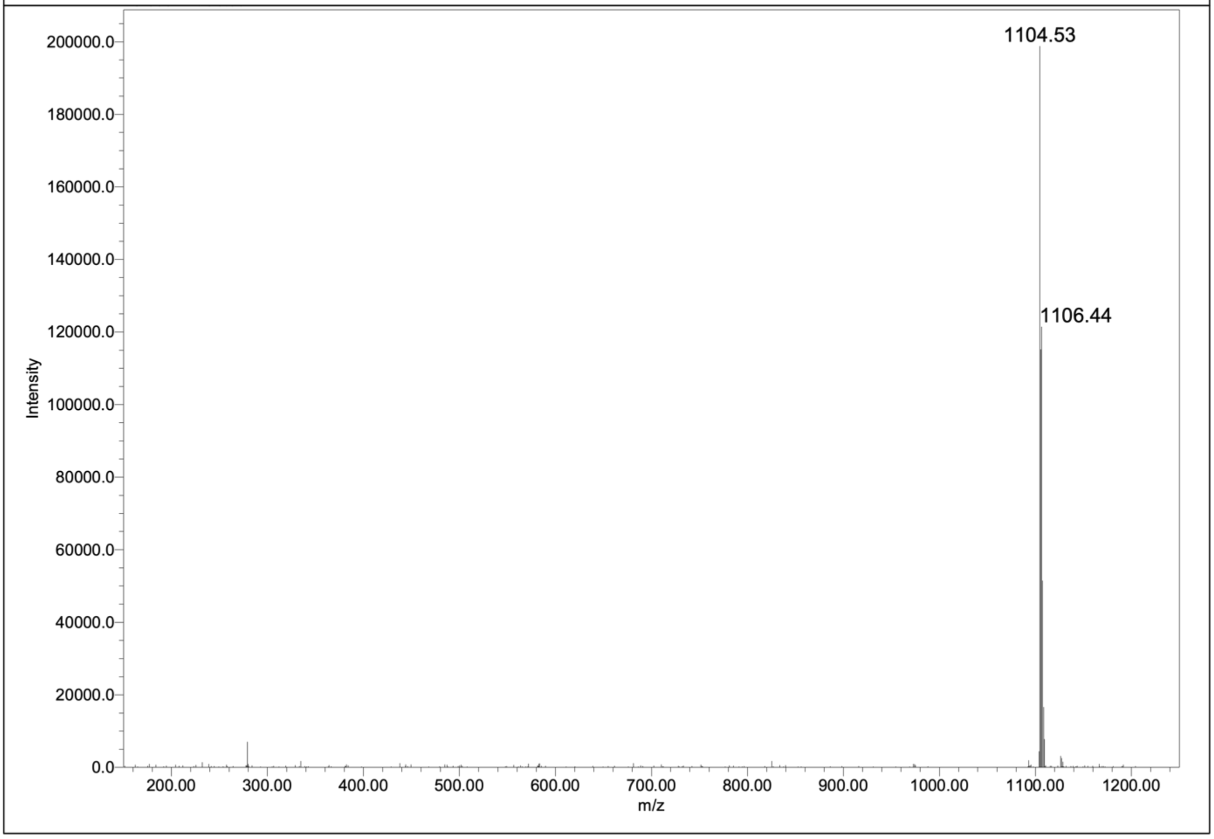
1104.53
1106.44
Intensity
m/z

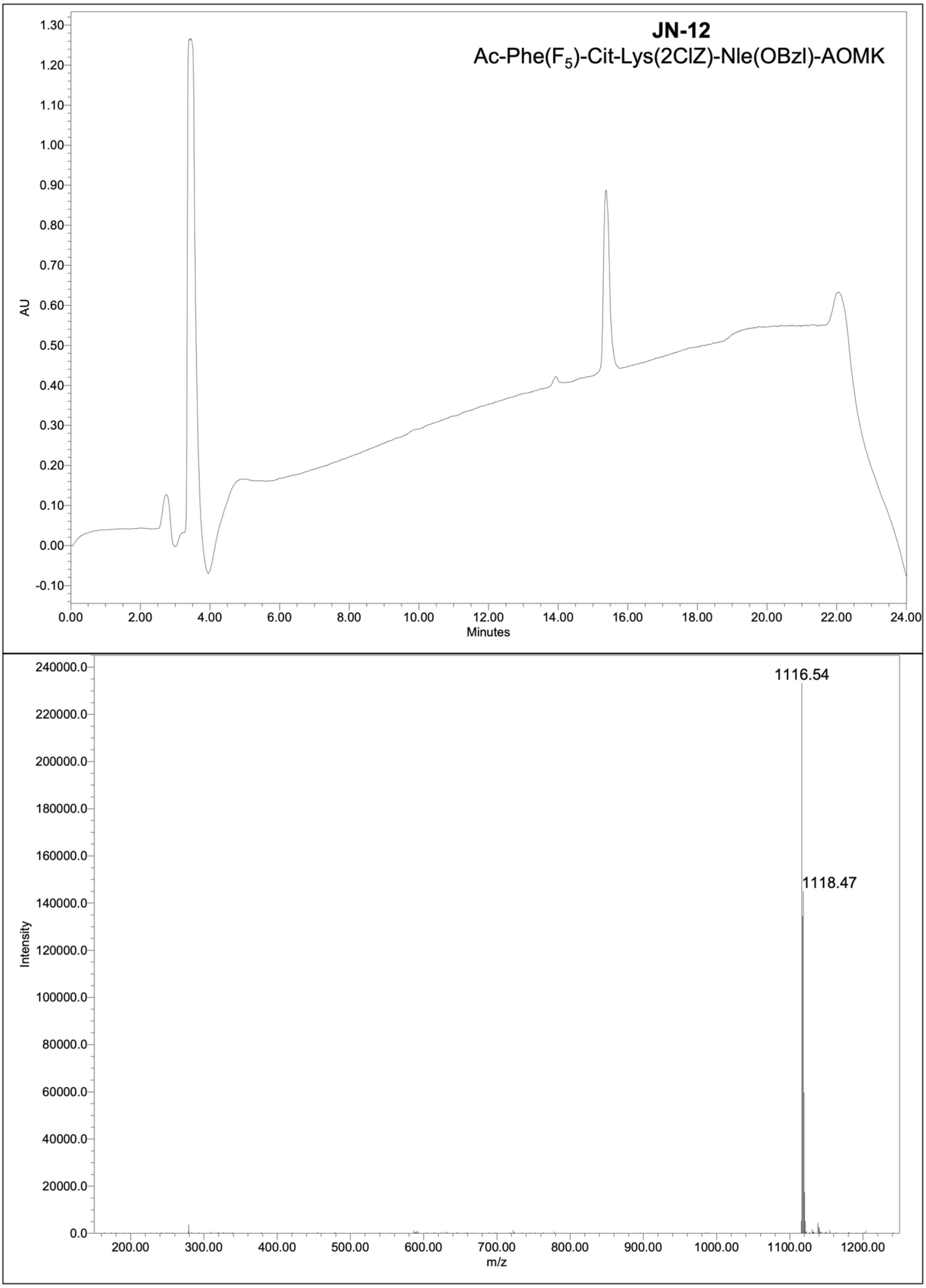
JN-12
Ac-Phe(F5)-Cit-Lys(2ClZ)-Nle(OBzl)-AOMK
AU
Minutes
1116.54
1118.47
Intensity
m/z

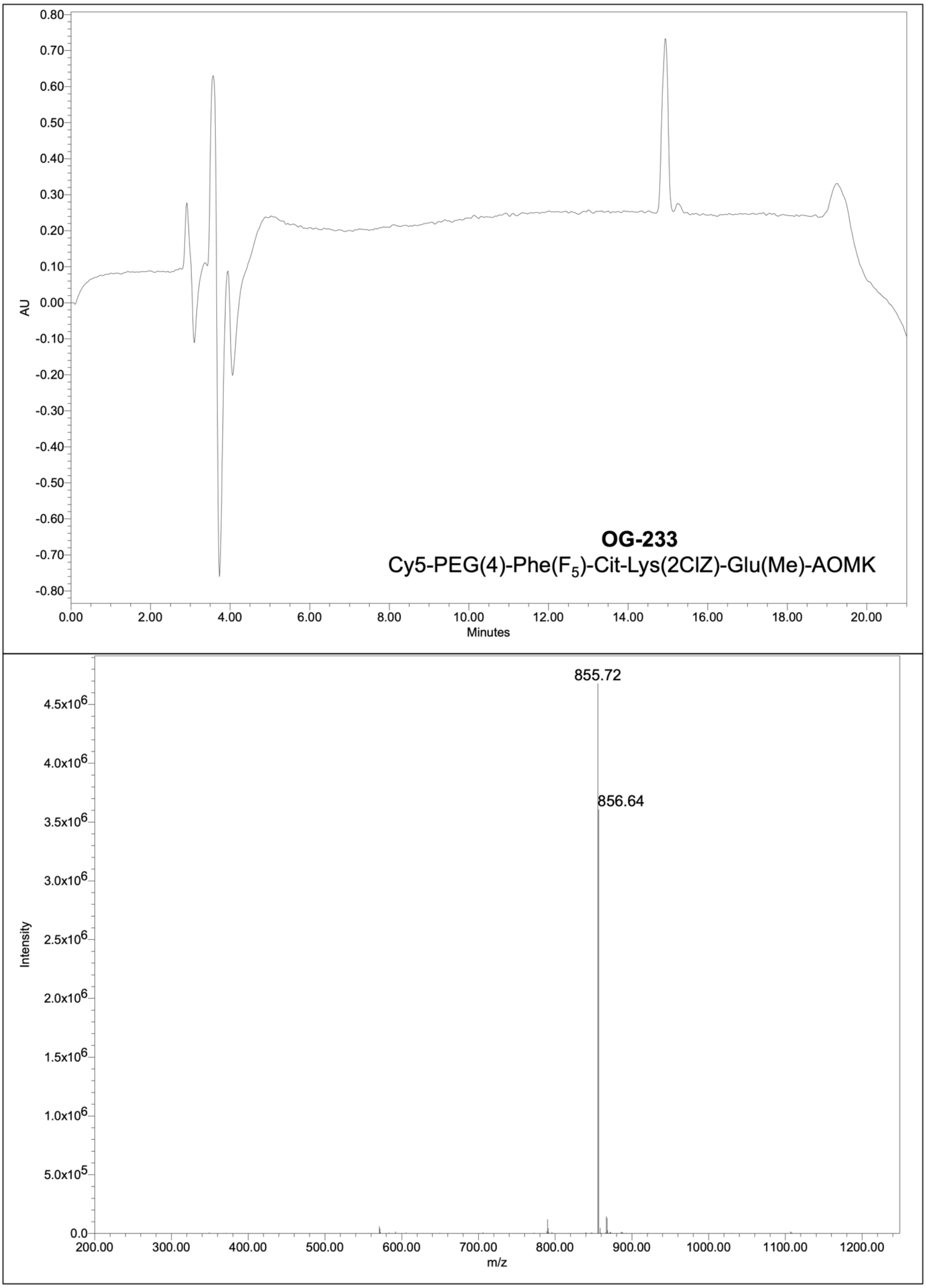

OG-233
Cy5-PEG(4)-Phe(F5)-Cit-Lys(2ClZ)-Glu(Me)-AOMK
AU
Minutes
855.72
856.64
Intensity
m/z

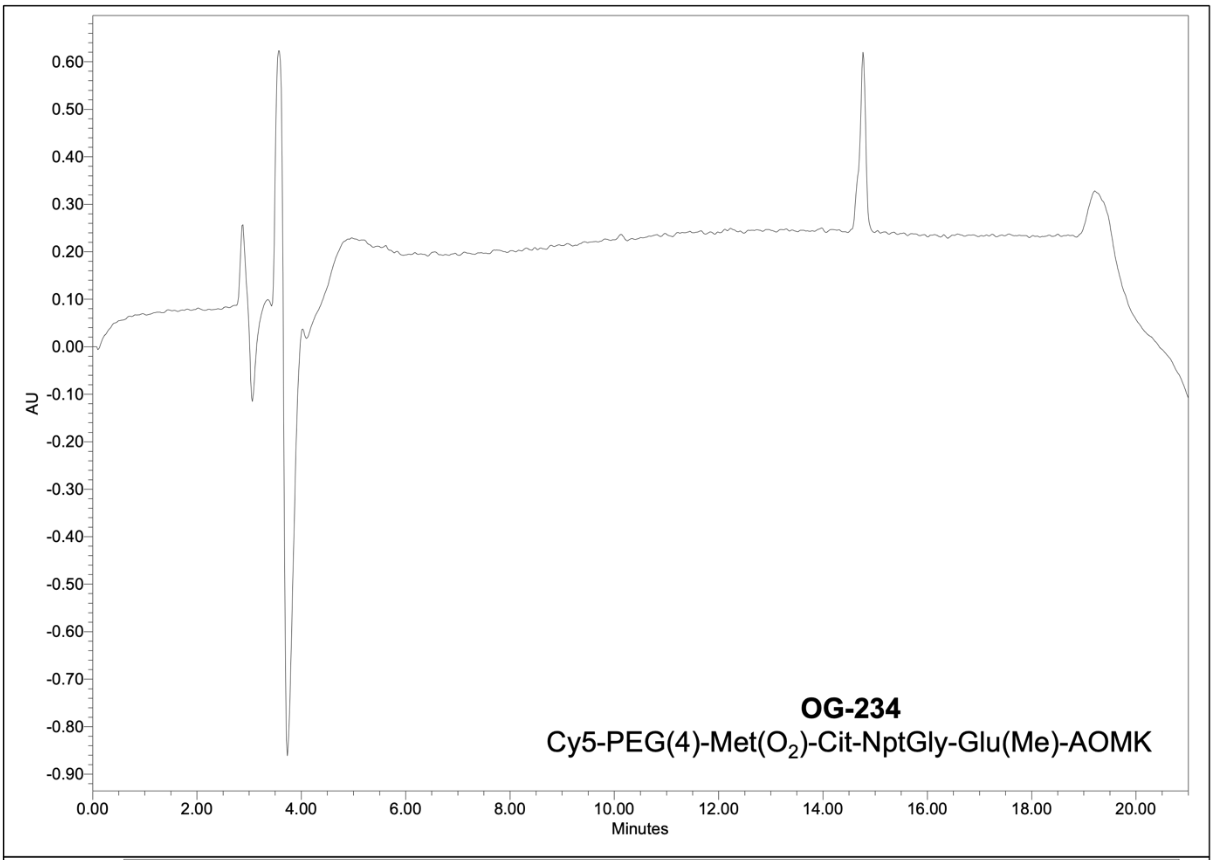

0.60
0.50
0.40
0.30
0.20
0.10
0.00
-0.10
-0.20
-0.30
-0.40
-0.50
-0.60
-0.70
-0.80
-0.90
AU
0.00
2.00
4.00
6.00
8.00
10.00
12.00
14.00
16.00
18.00
20.00
Minutes
OG-234
Cy5-PEG(4)-Met(O2)-Cit-NptGly-Glu(Me)-AOMK

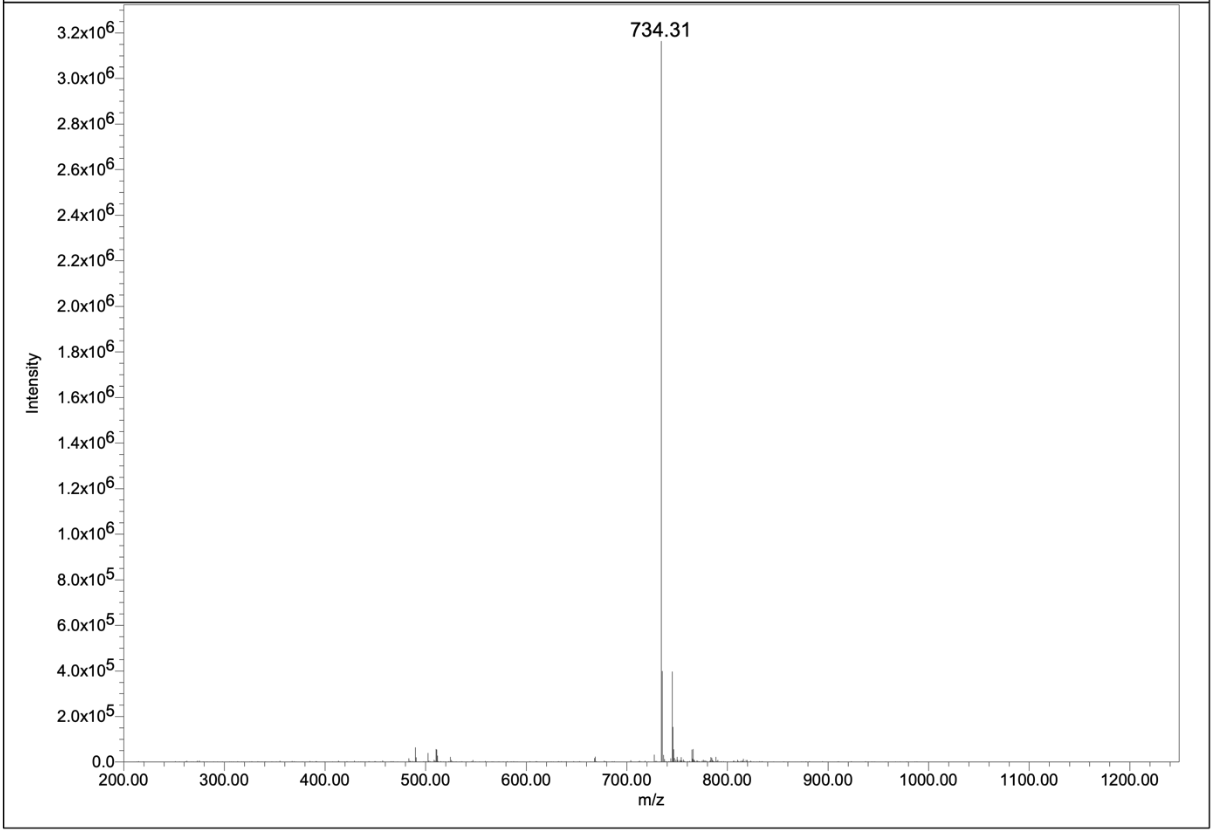

734.31
3.2x10^6
3.0x10^6
2.8x10^6
2.6x10^6
2.4x10^6
2.2x10^6
2.0x10^6
1.8x10^6
1.6x10^6
1.4x10^6
1.2x10^6
1.0x10^6
8.0x10^5
6.0x10^5
4.0x10^5
2.0x10^5
0.0
Intensity
200.00
300.00
400.00
500.00
600.00
700.00
800.00
900.00
1000.00
1100.00
1200.00
m/z

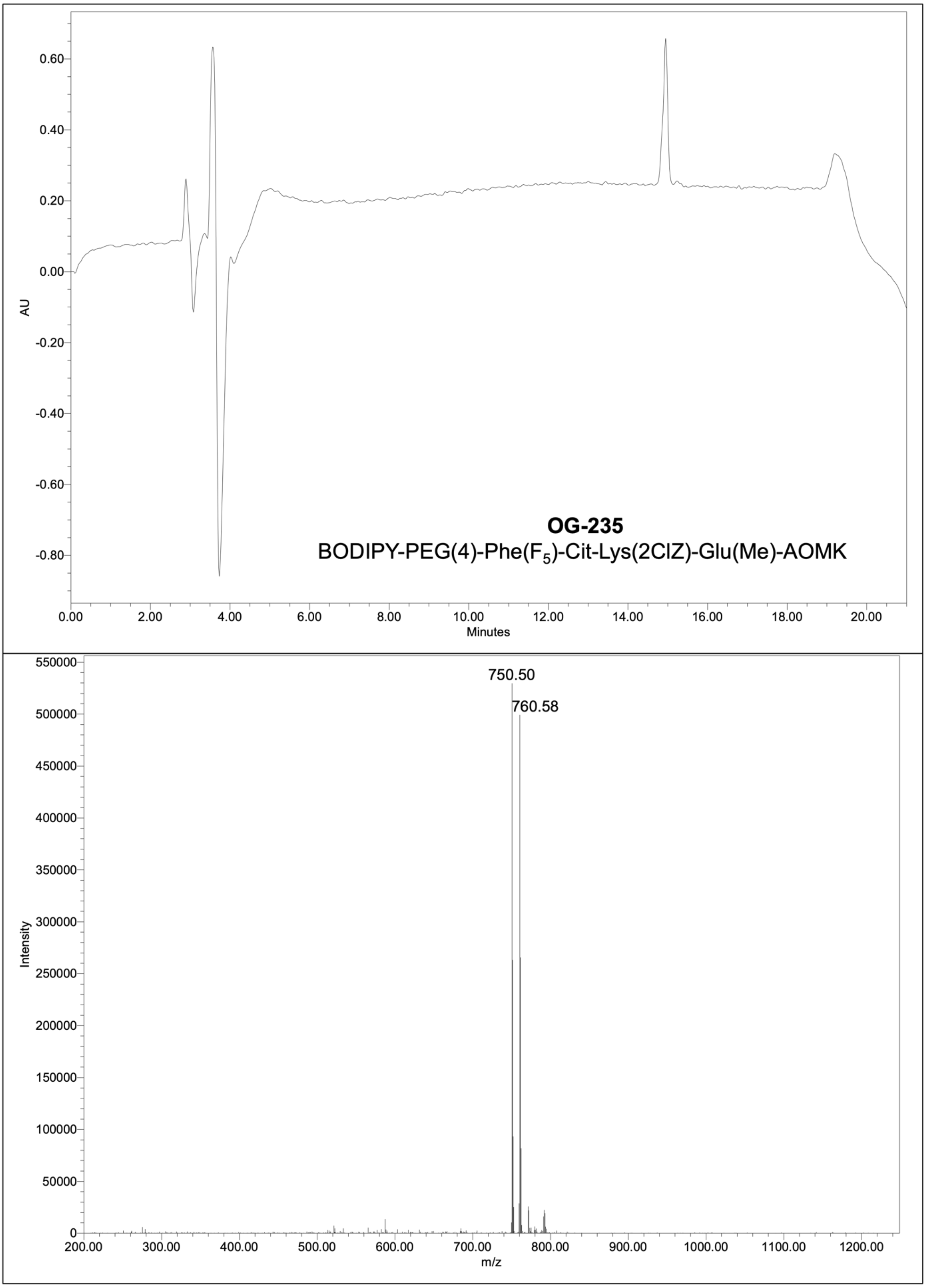

OG-235
BODIPY-PEG(4)-Phe(F5)-Cit-Lys(2ClZ)-Glu(Me)-AOMK
AU
Minutes
750.50
760.58
Intensity
m/z

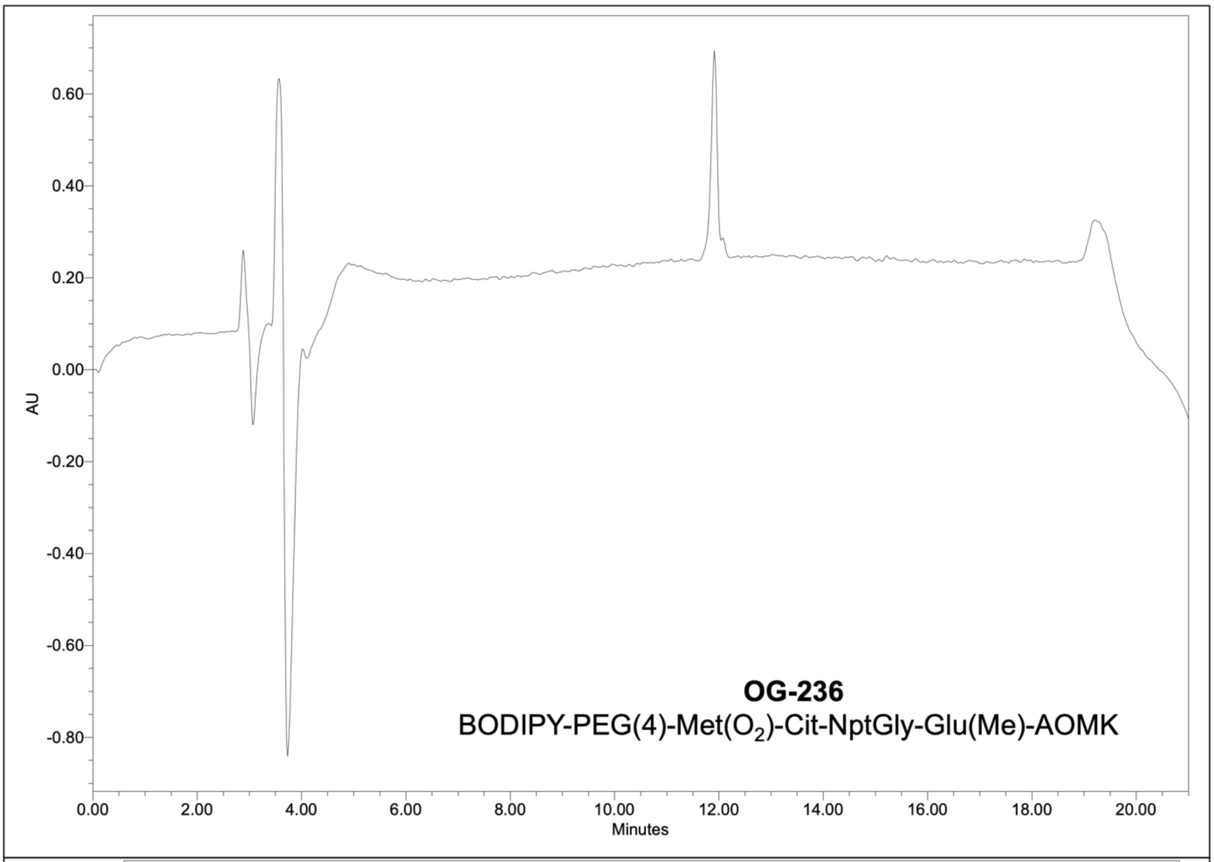

0.60
0.40
0.20
0.00
-0.20
-0.40
-0.60
-0.80
AU
OG-236
BODIPY-PEG(4)-Met(O2)-Cit-NptGly-Glu(Me)-AOMK
0.00
2.00
4.00
6.00
8.00
10.00
12.00
14.00
16.00
18.00
20.00
Minutes

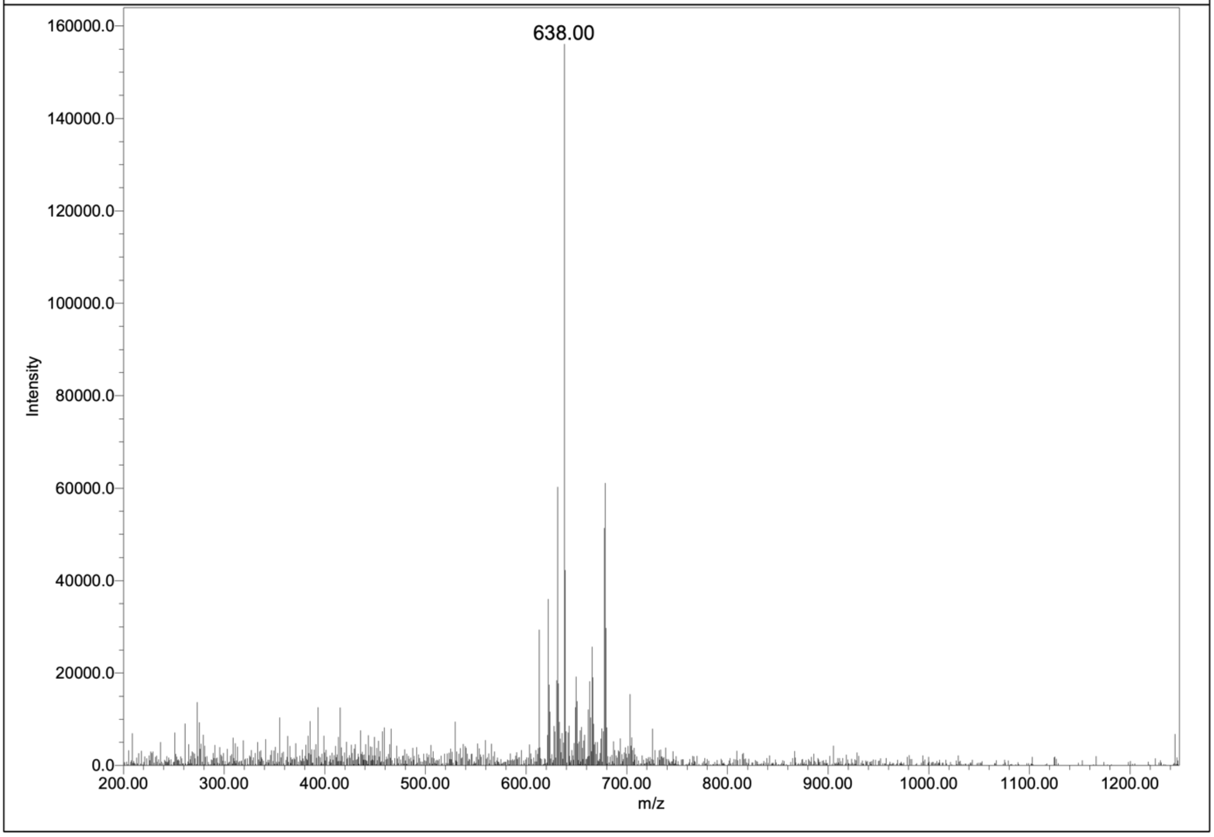

160000.0
140000.0
120000.0
100000.0
80000.0
60000.0
40000.0
20000.0
0.0
Intensity
638.00
200.00
300.00
400.00
500.00
600.00
700.00
800.00
900.00
1000.00
1100.00
1200.00
m/z

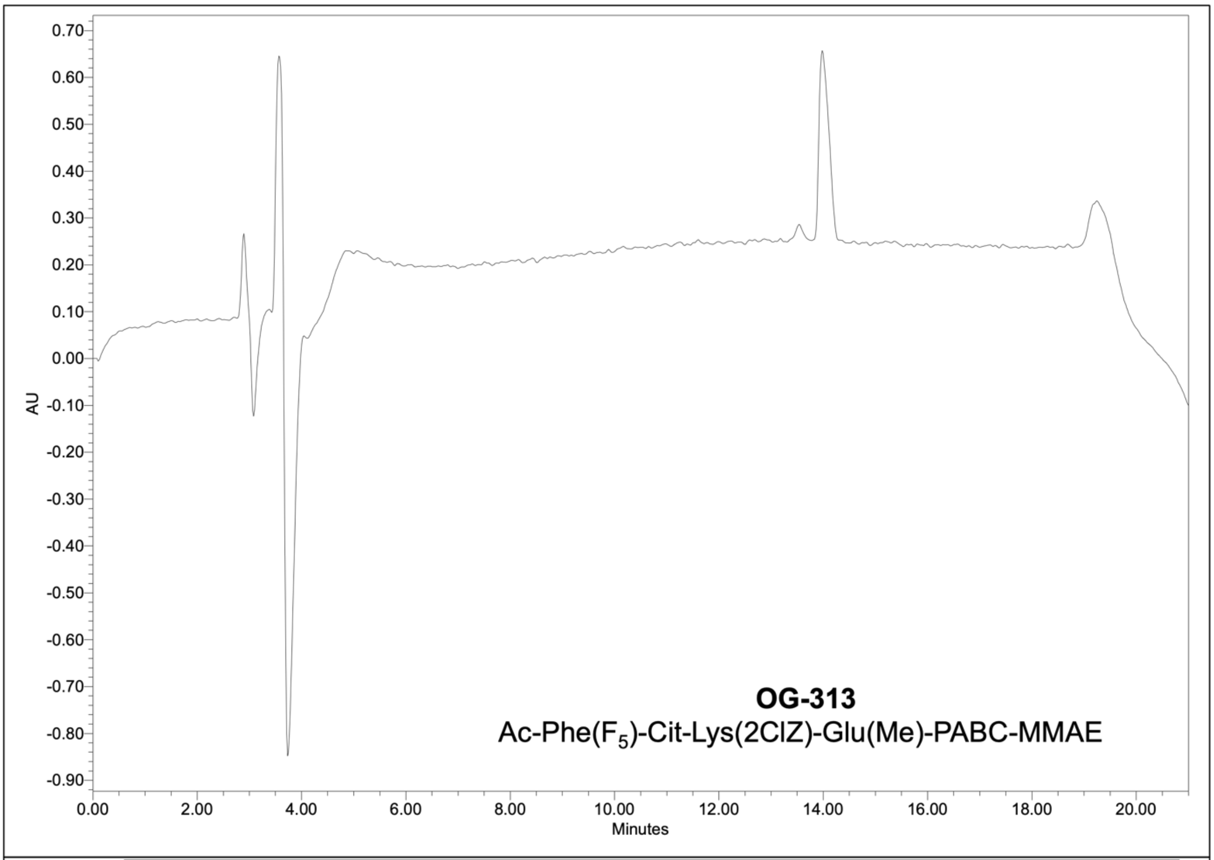
AU
Minutes
OG-313
Ac-Phe(F5)-Cit-Lys(2ClZ)-Glu(Me)-PABC-MMAE

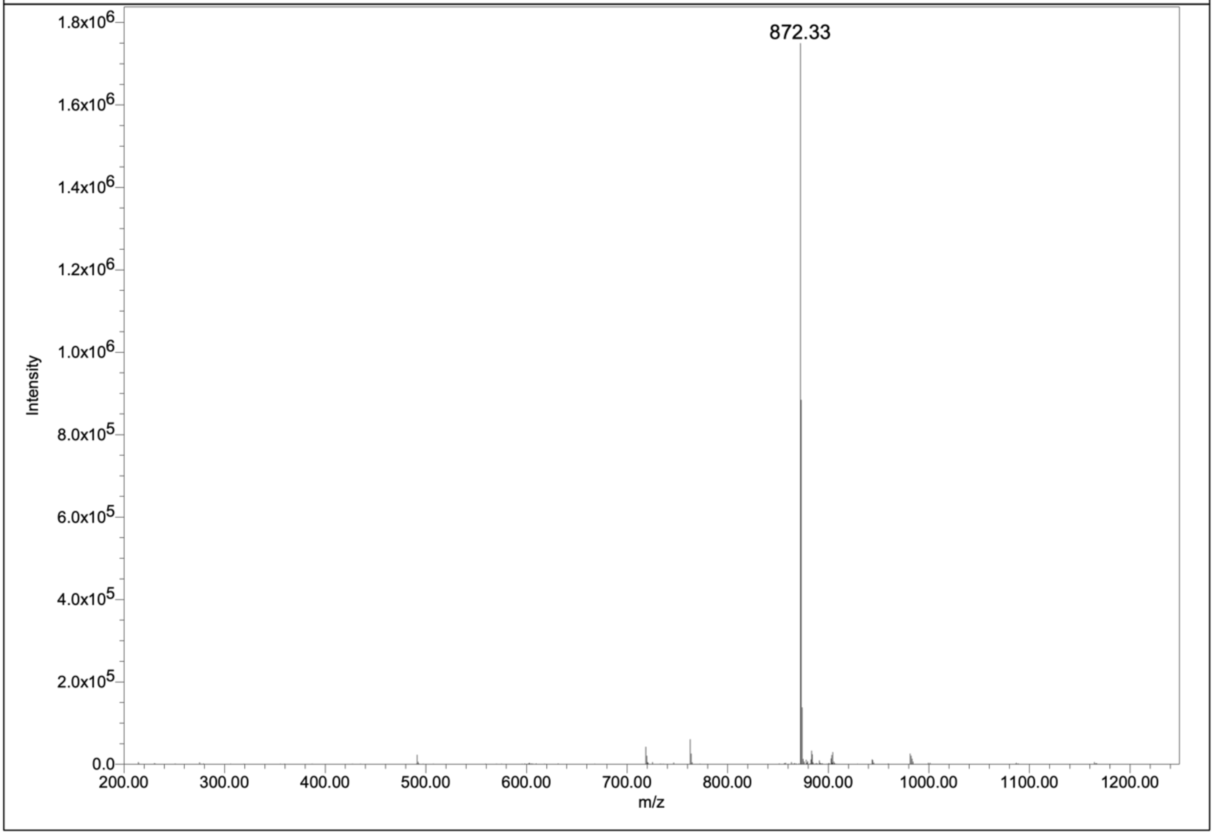
872.33
Intensity
m/z

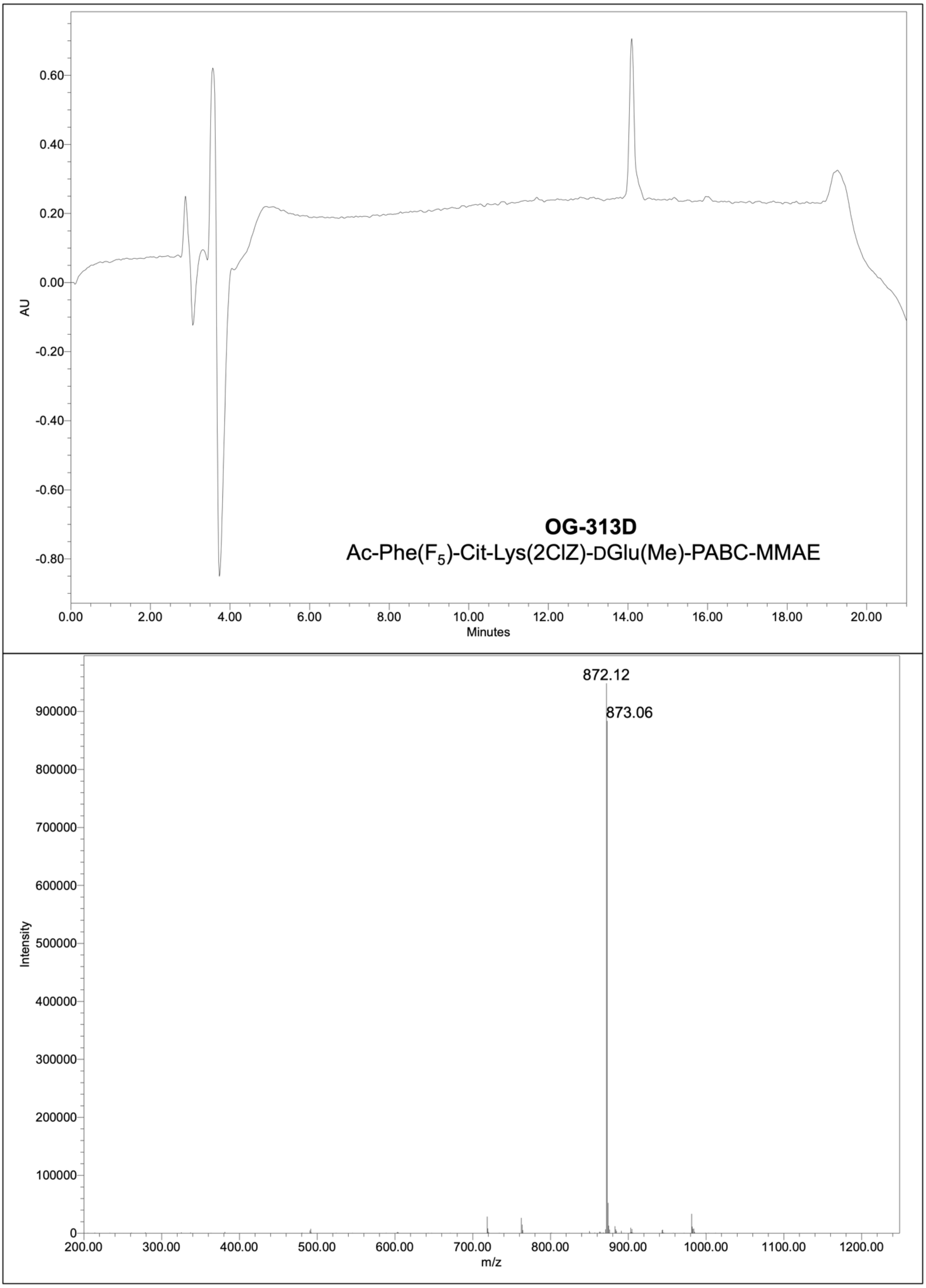
OG-313D
Ac-Phe(F5)-Cit-Lys(2ClZ)-DGlu(Me)-PABC-MMAE
AU
Minutes
0.60
0.40
0.20
0.00
-0.20
-0.40
-0.60
-0.80
0.00
2.00
4.00
6.00
8.00
10.00
12.00
14.00
16.00
18.00
20.00
872.12
873.06
Intensity
m/z
900000
800000
700000
600000
500000
400000
300000
200000
100000
0
200.00
300.00
400.00
500.00
600.00
700.00
800.00
900.00
1000.00
1100.00
1200.00

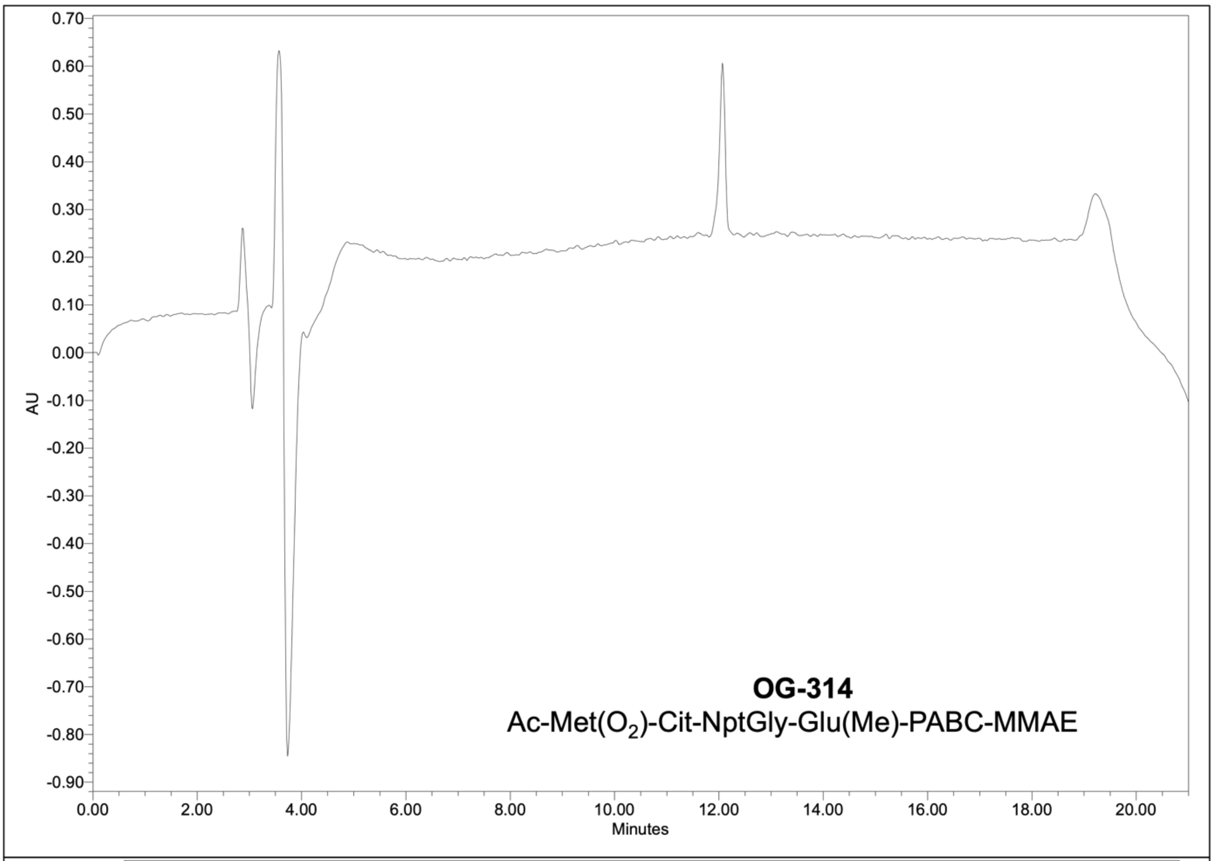
OG-314
Ac-Met(O2)-Cit-NptGly-Glu(Me)-PABC-MMAE
AU
Minutes
0.70
0.60
0.50
0.40
0.30
0.20
0.10
0.00
-0.10
-0.20
-0.30
-0.40
-0.50
-0.60
-0.70
-0.80
-0.90
0.00
2.00
4.00
6.00
8.00
10.00
12.00
14.00
16.00
18.00
20.00

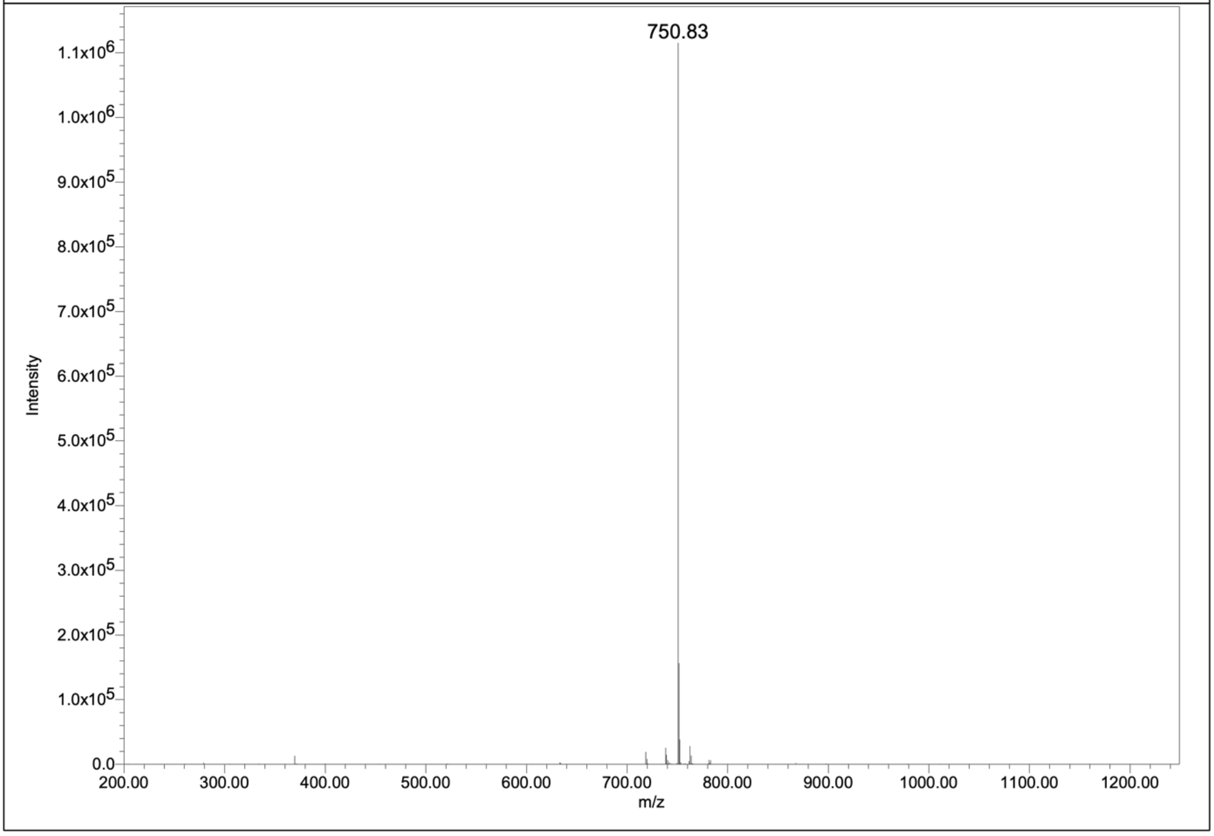
750.83
Intensity
m/z
1.1x10^6
1.0x10^6
9.0x10^5
8.0x10^5
7.0x10^5
6.0x10^5
5.0x10^5
4.0x10^5
3.0x10^5
2.0x10^5
1.0x10^5
0.0
200.00
300.00
400.00
500.00
600.00
700.00
800.00
900.00
1000.00
1100.00
1200.00

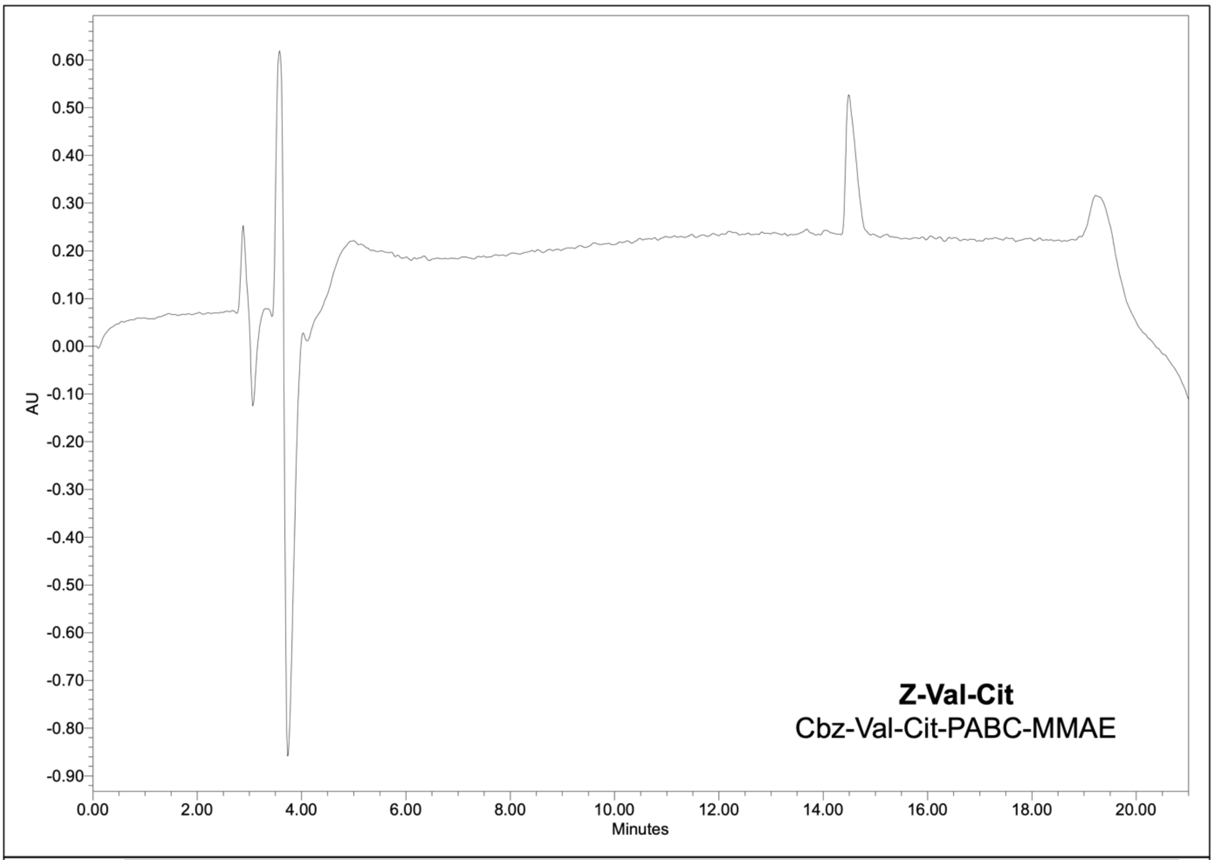

Z-Val-Cit
Cbz-Val-Cit-PABC-MMAE
AU
Minutes
0.60
0.50
0.40
0.30
0.20
0.10
0.00
-0.10
-0.20
-0.30
-0.40
-0.50
-0.60
-0.70
-0.80
-0.90
0.00
2.00
4.00
6.00
8.00
10.00
12.00
14.00
16.00
18.00
20.00

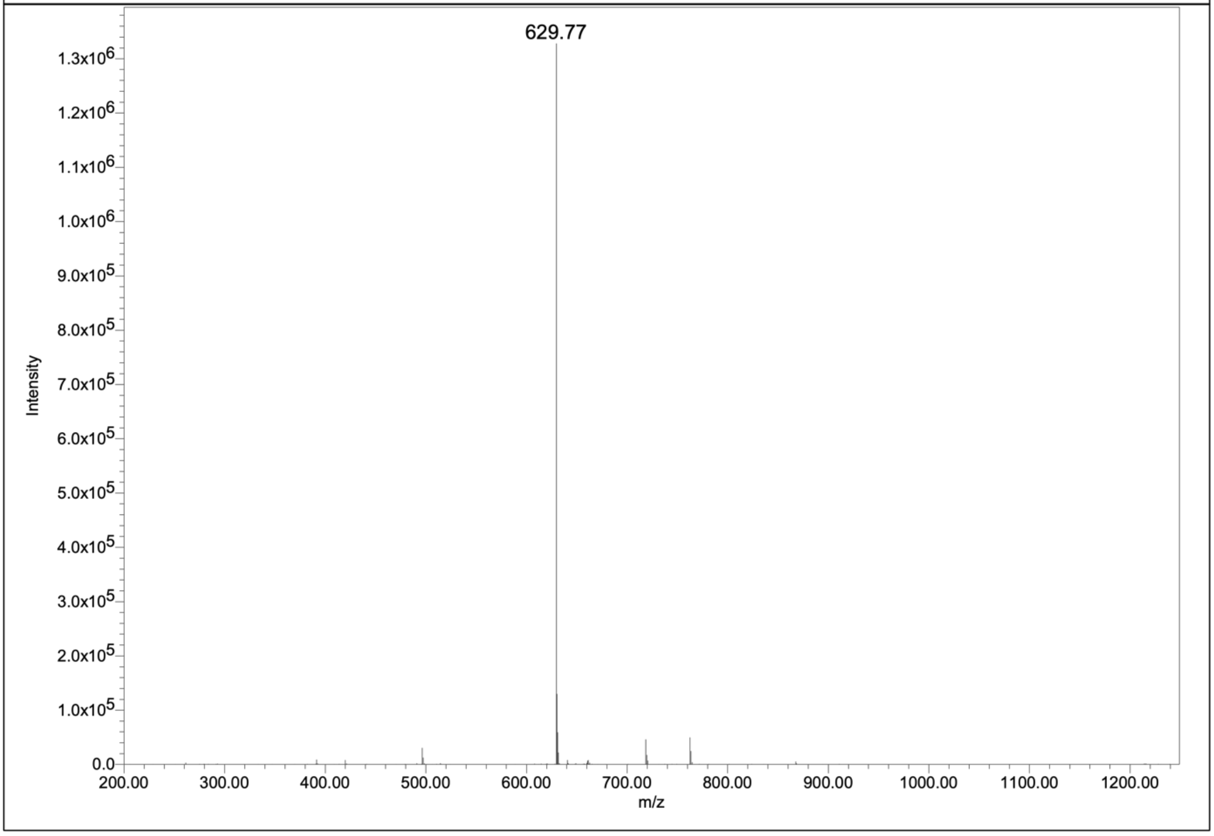

629.77
Intensity
m/z
1.3x10^6
1.2x10^6
1.1x10^6
1.0x10^6
9.0x10^5
8.0x10^5
7.0x10^5
6.0x10^5
5.0x10^5
4.0x10^5
3.0x10^5
2.0x10^5
1.0x10^5
0.0
200.00
300.00
400.00
500.00
600.00
700.00
800.00
900.00
1000.00
1100.00
1200.00
